# Supplementary material for: The draft genomes of five agriculturally important African orphan crops
Source: Gigascience. 2018 Dec 7;8(3):giy152. doi: 10.1093/gigascience/giy152 (PMC6405277; doi:10.1093/gigascience/giy152)
Supplement: GIGA-D-18-00275_Revision_1.pdf [file gigascience_8_3_giy152_s7.pdf]

GigaScience

The draft genomes of five agriculturally important African orphan crops

--Manuscript Draft--

**Manuscript Number:**	GIGA-D-18-00275R1
**Full Title:**	The draft genomes of five agriculturally important African orphan crops
**Article Type:**	Data Note
**Funding Information:**	Shenzhen Municipal Government of China (JCYJ20150831201643396) — Dr. Yue Chang<br>Shenzhen Municipal Government of China (JCYJ20150529150409546) — Dr. Shifeng Cheng<br>State Key Laboratory of Agricultural Genomics (2011DQ782025) — Mr. Huan Liu<br>Guangdong Provincial Key Laboratory of Genome Read and Write (2017B030301011) — Mr. Haorong Lu
**Abstract:**	Background: Continuous growth in the world population is expected to double the worldwide demand for food by 2050. Moreover, 88% of countries are currently facing a serious burden of malnutrition, especially in Africa and Southern & South-Eastern Asia. About 95% of the present food energy needs of humans are fulfilled by 30 species, within which wheat, maize and rice provide the majority of calories. Therefore, to diversify and stabilize global food supply, enhance agricultural productivity and tackle malnutrition in these countries, a greater utilization of neglected or underutilized local plants (generally so-called orphan crops, but also a few plants with special contribution to agriculture, such agroforestry and nutrient) could be a partial solution.<br><br>Findings: Here we present draft genome information from five agriculturally, biologically, medicinally and economically important underutilized plants in Africa, namely; Vigna subterranea, Lablab purpureus, Faidherbia albida, Sclerocarya birrea, and Moringa oleifera. The assembled genomes range in size from 217 to 654 Mb. In addition, we have predicted 31707, 20946, 28979, 18937, 18451 protein-coding genes in V. subterranea, L. purpureus, F. albida, S. birrea and M. oleifera respectively. We have further analyzed the expansion and contraction of selected gene families, and characterized root-nodule-symbiosis genes, transcription factors and starch biosynthesis related genes in these genomes.<br><br>Conclusions: This genome data will be useful to identify and characterize agronomically important genes and understand their mode of actions, enabling genomics-based, evolutionary studies, and breeding strategies for designing faster, focused and predictable crop improvement programs.
**Corresponding Author:**	Xin Liu, Ph.D.<br>BGI<br>CHINA
**Corresponding Author Secondary Information:**	
**Corresponding Author's Institution:**	BGI
**Corresponding Author's Secondary Institution:**	
**First Author:**	Yue Chang
**First Author Secondary Information:**	
**Order of Authors:**	Yue Chang<br>Huan Liu<br>Min Liu

Powered by Editorial Manager® and ProduXion Manager® from Aries Systems Corporation

	Xuezhu Liao
	Sunil Kumar Sahu
	Yuan Fu
	Bo Song
	Shifeng Cheng
	Robert Kariba
	Samuel Muthemba
	Prasad S. Hendre
	Sean Mayes
	Wai Kuan Ho
	Presidor Kendabie
	Sibo Wang
	Linzhou Li
	Alice Muchugi
	Ramni Jamnadass
	Haorong Lu
	Shufeng Peng
	Allen Van Deynze
	Anthony Simons
	Howard Yana-Shapiro
	Xun Xu
	Huanming Yang
	Jian Wang
	Xin Liu, Ph.D.
**Order of Authors Secondary Information:**	
**Response to Reviewers:**	Responses to comments of Reviewer #1<br><br>The topic of nitrogen fixation is complex and well studied. The brief section in this paper begins to ask some good question (about presence of genes that play important roles in nodulation) - but the presentation is insufficient to conclude "The reason why F. albida showed a relatively lower ability to fix nitrogen [77] could be explained by the loss of IPD3, NFP, and some proteins with lower efficiency which would have taken its place in F. albida." See the recent papers by Greismann et al., 10.1126/science.aat1743 and van Velzen et al., https://doi.org/10.1073/pnas.1721395115, for state-of-the-art work in this area.<br><br>Response: Thank you for the suggestion. The suggested reference manuscript on the “Phylogenomics studies of nitrogen-fixing root nodule symbiosis” which is recently published in Science (Greismann et al.) is the outcome of our BGI-Research team along with our collaborators. We do referred the suggested papers, and removed the confused conclusion, and revised the description, as follows:<br>“The difference in the components within RNS pathway (Table 8) together with the relatively weak nitrogen-fixing ability [80] of F. albida thus make itself a good reference in the research of RNS diversification”.<br><br>1. Abstract: In the first sentence, the initial article, "A", is unnecessary ("A continued growth ...").

Powered by Editorial Manager® and ProduXion Manager® from Aries Systems Corporation

Response: According to your suggestion, we have revised the sentence, as follows:
“Continuous growth in the world population is expected to double the worldwide demand for food by 2050.”

2. Abstract, third sentence: typically, a sentence isn't started with a number ("30 species").

Response: According to your suggestion, we have revised the sentence, as follows:
“About 95% of the present food energy needs of humans are fulfilled by 30 species, within which wheat, maize and rice provide the majority of calories.”

3. Introduction: a minor point, but I am skeptical that the "World Population Prospects" from the U.N. (reference 1) is suitably paraphrased this way: "ensuring a sustainable food supply to meet the energy and nutritional needs of the expanding population is the greatest global challenge ahead of us." That is: scanning the report, I don't see that the report makes a claim about the "greatest global challenge" in an absolute sense (putting this need among others such as climate change, international conflict, etc.).

Response: Thank you for raising this question. According to your suggestion, we have revised the sentence, as follows:
“The world’s population is expected to reach 9.8 billion by 2050, thus ensuring a sustainable food supply to meet the energy and nutritional needs of the expanding population is one of the greatest global challenge.”

4. Introduction: "the utilization of crops plants appear to be the best choice" -- There is no other choice, right? We predominantly use crop plants (the only others being wild-harvested, non-crop foods).

Response: Thank you for the suggestion. According to your suggestion, we have revised the sentence, as follows:
“the utilization of potential crops (both model and non-model) plants appears to be a better choice.”

5. "which originated in West Africa, and cultivated in Sub-Saharan" --> "which originated in West Africa, and IS cultivated in Sub-Saharan" (for parallel construction)

Response: According to your suggestion, we have revised the sentence, as follows:
“which originated in West Africa, and is cultivated in Sub-Saharan areas, particularly Nigeria.”

6. "thereby highly making bambara groundnut a complete food" -- nonstandard word usage (omit "highly" to make it standard).

Response: According to your suggestion, we have omited “highly”, as follows:
“thereby making bambara groundnut a complete food.”

7. Section on Lablab: "South West" should be one word, and should probably lower-case unless it names a particular place, e.g. "the Southwest": "In southwestern parts of Bangladesh ..."

Response: According to your suggestion, we have revised the sentence, as follows:
“In southwestern parts of Bangladesh, lablab is reported to have a total production area of approximately 48000 ha.”

8. Extra period: "Kenya, approx.. 10,000"

Response: The suggested correction was implemented as follows:
“Kenya, approx. 10,000 ha”

9. Section on phylogenetic analysis: "divergence time between M. truncatula and legumes" -- what other legumes? (since Medicago is itself a legume)

Response: The suggested correction was implemented as follows:

“39-59 Mya between M. truncatula and the main branch of legumes, 15-30 Mya between G. max and P. vulgaris, and 83-90 Mya between T. cacao and A. thaliana.”

10. "In the present study, the divergence time between F. albida and Papilionoideae was predicted to be 79.1" - This is way outside the expected ranges, because the legume family itself is estimated to have originated around 60-64 Mya. Also, the value would depend on the particular species selected within the Papilionoideae - because rates are species-specific. See rates in Lavin et al. (2005), DOI: 10.1080/10635150590947131

Response: Thank you for raising this important point. We have removed the confused description, as follows:
“Based on the tree constructed by single-copy-family genes, the divergence time between F. albida and Papilionoideae was predicted to be 79.1 (70.0-87.0) Mya, which is a little different from the previous predicted origin of legumes based on two gene markers (matk and rbcL) (Lavin et al., 2005).”

11. Section "Identification of protein, starch, and fatty acid biosynthesis related genes" "Based on these observations we inferred that the ability to synthesize lecithin in V. subterranea is higher than that of soybeans" -- biosynthetic ability can't be inferred solely by the presence of gene sequences. All that can be said is that a necessary factor is present.

Response: Thank you for the suggestion. We do agree with your point, and removed the hypothetical description, and revised the sentence as follows:
“Based on these observations we inferred that the all the necessary factor to synthesize lecithin are present in V. subterranea.”

12. "... and in comparison with other orphan crops it has higher potential to be a new food crop." -- on what basis? Certainly not on the basis of gene composition, or on the ability to synthesize lecithin (which is itself of questionable nutritional value).

Response: Thank you for the suggestion. We do agree with your point, and removed the hypothetical description.

13. Sentence beginning "Therefore, this fine reference genomes together" needs to be rewritten. I don't think that "fine" is the intended word.

Response: Thank you for the suggestion. We have deleted this sentence.

14. Section "Identification of root nodule symbiosis pathway": "it has a major impact" --> "they have a major impact"

Response: According to your suggestion, we have revised the sentence, as follows:
“They have a major impact on global nitrogen cycle.”

15. Data availability: I see that PRJNA453822 points to Faidherbia (good), but I don't find PRJNA474418 in GenBank. Should the bioproject IDs be given for the other species in the study?

Response: Thank you for pointing this out. Actually, we have now released the data (PRJNA474418) in NCBI.

16. Data availability: "The assembly and annotation of the B. ceiba genome and other supporting data, including BUSCO results, are available in the GigaScience database" -- is this an error? I assume this refers to Bombax ceiba - which is not described in the paper.

Response: Thank you for pointing out the typing error. According to your suggestion, we have
revised the sentence, as follows:
“The assembly and annotation of the five genomes and other supporting data, including BUSCO results, are available in the GigaScience GigaDB repository.”

Powered by Editorial Manager® and ProduXion Manager® from Aries Systems Corporation

Responses to comments of Reviewer #2

1. The premises of the study talks about orphan crops which are important for Africa: to qualify this statement, the crops chosen should be either consumed or grown by Africans in large quantity: Based on the introduction and the statistics given therein M. oleifera and L. purpureus do not qualify.

Response: The improper description is replaced with "underutilized local plants". For example, in the abstract" ..enhance agricultural productivity and tackle malnutrition in these countries, a greater utilization of neglected or underutilized local plants (generally so-called orphan crops, but also a few plants with special contribution to agriculture, such agroforestry and nutrient) could be a partial solution".

2. M. oleifera genome is already sequenced and published (Tian et al., 2015; Sci China Life Sci. 2015 Jul; 58(7):627-38. doi: 10.1007/s11427-015-4872-x.). The manuscript neither mentions this fact nor compares their results with this.

Response: Thank you for the suggestion. We add the description in Page 5, L16-18, as follows:
"Prior to this study, a draft genome of Moringa oleifera from Yunnan (China) was also reported with similar genome assembly size and gene numbers compared to our version".

3. The results of RNA-seq have been used only for checking the genome completion suggesting gross underutilization of data. The materials and methods says just different parts of the plant has been subjected to RNA-seq. RNA-seq data of S. birrea is completely missing and there is no explanation of the same in the manuscript. The information provided in the supplementary file shows that there is no common denominator followed for the choice of tissue for RNA-seq. Further from table 5, it could be seen that only one among these various tissues have been used for checking the completeness of the WGS assembly. Overall, this gives a very hazy picture though a lot of work has been done and huge data-sets have been generated. I would recommend culling the data which is in no way utilized for obtaining the results provided in this manuscript.

Response: Thank you for raising this important point. We have actually compiled all the transcriptome data from different tissues, and used the combined version to check the completeness of the WGS assembly again. The results are shown in the Table 3 (not Table 5).

4. Genome and RNA-seq statistics are given only in Gb and Mb. This should be accompanied by number of reads and nucleotides.

Response: Thank you for the suggestion. According to your suggestion, we have revised the additional file 1: tableS1 and tableS2, and we used "bp" instead of "Gb", and also added "Reads number (bp)" data.

5. The difference between raw data and clean data seem to be too high ((30 to 43 %) except for S. birrea with respect to WGS data. Any specific reasons? This is even after keeping the cut off for quality score pretty low (< 16). Even for Sanger this kept as 20 while for NGS, this score is 30 to have high quality data.

Response: Thank you for pointing this out. Actually, the difference between raw and clean data is caused due to the filtering of the duplicated reads from the mate-pair libraries. However, for the pair-end data, the clean rate percentage were more than 80%. Therefore, we strongly believe that the cut off (<16) is suitable and reliable for our data. Kindly refer the below table for your kind perusal.

6. The comparison of orthologs within the five species does not seem to have a common ground as they belong to different species with not much evolutionary relationships to call for orthologous comparison. It would have been worthwhile to have the orthologous comparison with the related species. The choice of species in Table 5 needs to be explained.

Powered by Editorial Manager® and ProduXion Manager® from Aries Systems Corporation

Response: Thank you for the nice suggestion. We made the changes according to your suggestion. The orthologs of all the 14 species were identified just to get the single-copy-family genes for the construction of the tree. The comparison was made within fabids (for F. albida, L. purpureus and V. subterranea) and malvids (for M. oleifera and S. birrea) respectively. The species details in the Table 5 is now updated according to Figure 2.

7. In continuation of the previous point, the Vigna mungo genome and V. anguicularis genome should have been used along with other more complete legume genome (species) and mentioned in the manuscript while discussing the V subterranea.

Response: Thank you for the suggestion. We have now added the description in Page 5 L3-L4, as follows:
"The genomes of mung bean and adzuki bean have been published [9, 10], which also belongs to the Vigna genus"

8. The introduction does not talk about the previous genomic resources available in these five crops.
Response: Thank you for the suggestion. We admit our negligence. We have now added the relevant description regarding the previous genomic resources in the introduction section as well as in the data description, wherever necessary.

9. Table 4 formatting is confusing. Is it really required?

Response: Yes, the information on different classes of repeats (%) in five species is important. According to your suggestion, we have revised the table 4 for more better understanding. We have now classified the Repeat Type in a more detailed manner (Table 4)

10. A lot of analysis has been mentioned in Supplementary data - however there is no major point emerging out of it - such data may be removed from the manuscript altogether. It just increases the bulk of the paper without really contributing anything.

Response: Thank you for the suggestion. We have removed the previous
table S13. Comparative analysis of the protein biosynthesis related genes in each species., table S14. Comparative analysis of the starch biosynthesis related genes in each species.
table S15. Comparative analysis of the fatty acid-plastids biosynthesis related genes in each species.
table S16. Comparative analysis of the fatty acid synthesis and storage related genes in each species.,
table S17. Comparative analysis of the fatty acid degradation related genes in each species. in additional file 2.
And add new table in additional file 1, as follows:
Table S6. Enriched pathways of unique paralogs genes in families.
Table S7. Enriched GO terms (level 3) of unique paralogs genes in families.
What's more ,we renumber the table.
11. Overall, results and discussion section shows hardly any discussion and incomplete results

Response: As our manuscript is a "data note" we focused mainly on data and its analysis part. The detailed findings and discussion will be presented in our subsequent manuscript covering the genomic data of several orphan crop species. The overall goal of the African Orphan Crops Consortium (AOCC) and BGI is to sequence, assemble and annotate the genomes of 101 plants contributed to traditional African food supplies by 2020 (www.africanorphancrops.org).

Minor shortcomings
1. Please read the manuscript carefully and check punctuation. Examples: Page 20: Line No: In other cereals .... in barley.
Page 22: LN: 48-50. Fragment owing to wrong punctuation.
2. The accession numbers of these data-sets are indicated as SSR in the respective supplementary tables.

Powered by Editorial Manager® and ProduXion Manager® from Aries Systems Corporation

Response: We have now rectified the above mentioned errors.

Responses to comments of Reviewer #3

1. The plants sequenced in this project have smaller genome size compared to many other sequenced crops, and repeat elements are also comparatively low. However none of the assemblies are complete and couldn't assemble into the chromosome level. If the authors have used long insert libraries also, it would have been better

Response: Thank you for the suggestion, we do agree with your comments. The incomplete assembly could be due to large fragments of repetitive sequences. This is one of the reasons, why we have submitted the manuscript as "data note" rather than "full length article". The experience gained from the sequencing of five orphan species, we plan to apply more sequencing strategies for the future African orphan project, like techniques generating longer reads.

2. "Various gene structure parameters were compared to the related species of each sequenced genome as summarized in table 5"- The number of protein coding genes in these sequenced genomes seems to be less compared to the related species. Can the authors provide an explanation for this?

Response: Thank you for the suggestion. The number of protein coding genes in V. subterranean and F. albida is similar to other legumes, except G. max and M. truncatula. These exceptionally large number is caused by their lineage-specific duplication. The lower numbers in other three species may be related to their smaller genome size. But, our BUSCO results showed a relative high completeness of core genes, compared to those of other published plant genomes, and the size of the assemblies is closer to the estimated sizes. For instance, the previously reported gene number in M. oleifera (Tian et al., 2015; Sci China Life Sci. 2015) is extremely close to our number. Therefore, the possibility of mis-annotation of genes is pretty low.

3. Figure S5 is not provided

Response: Thank you for the suggestion. It is provided but our previous layout was confusing. Thank you for reminding, and we have modified it in this version.

4. 633, 372, 861, 364 and 216 genes are unannotated in V. subterranea L. purpureus F. albida S. birrea and M. oleifera respectively. Are these genes specific to the respective genomes?

Response: We found that there are 400, 305, 1514, 293, 172 unannotated genes which does not cluster with other species in gene family of V. subterranea L. purpureus F. albida S. birrea and M. oleifera respectively. Hence, we speculated that these genes are specific to the respective genomes. Kindly refer the specific results in the below table.

5. "Furthermore, the 10,103 gene families of V. subterranea, L. purpureus, F. albida, M. truncatula and G. max were clustered (Figure 2A). There were 1,105 orthologous families shared by the four Papilionoideae species, while 808 gene families containing 1,966 genes were specific to F. albida, 281 gene families containing 538 genes were specific to L. purpureus, 789 gene families containing 3,118 genes were specific to V. subterranea.
Moreover, 8,184 gene families of S. birrea, M. oleifera, C. papaya, C. sinensis and T. cacao were clustered (Figure 2B), of which 365 gene families containing 798 genes were specific to M. oleifera, 362 gene families containing 796 genes were specific to S. birrea, respectively". -To which class the specific genes mostly belong in the functional annotation?

Response: Thank you for raising the question. We additionally analyzed our data and updated the description as follows:
"The enrichment analysis on KEGG pathway of the paralogs genes were also

	calculated (Additional file1: Table S6, S7). The functional annotation revealed that they mainly correspond to the carbon fixation, zeatin biosynthesis, glyoxylate and dicarboxylate metabolism in V. subterranea. However, for L. purpureus, the fatty acid elongation pathway was enriched. While in F. albida, the pathways corresponding to the plant-pathogen interaction and cyanoamino acid metabolism were enriched. In S. birrea, the pathways of plant-pathogen interaction, starch and sucrose metabolism, fatty acid biosynthesis were enriched. In M. oleifera, the pathways related to fatty acid and diterpenoid biosynthesis, cyanoamino acid metabolism were enriched. The enrichment analysis on GO of paralogs genes were ion binding, metabolic process, disease resistance, cell component, biological process in V. subterranea, L. purpureus, F. albida, M. oleifera, and S. birrea respectively."<br><br>6. In the phylogenetic analysis with 141 single-copy genes from 14 species, Populus trichocarpa clusters with other members in Fabids. But in some other phylogenetic analysis constructed using the same criteria, the group malpigiales, which includes Populus trichocarpa clusters with malvids or as a separate group. How do the authors explain this?<br><br>Response: Thank you for the nice suggestion. The figure 1 in the earlier version of manuscript was only a hand-drawn tree, and was used to display the taxonomy of our sequenced species. The taxonomic position of Populus trichocarpa was according to the NCBI taxonomy. The actual phylogenetic tree based on 141-gene was constructed without Populus trichocarpa (Figure 3 & 4). Therefore, to avoid the confusion between different phylogenetic trees in the manuscript, we have merged the previous figure 1 and 3, and moved figure 4 to the additional file1.<br><br>Chang et al 2018. Supporting data for "The draft genomes of five agriculturally important African orphan crops". GigaScience Database 2018. http://dx.doi.org/10.5524/100504.
**Additional Information:**	
**Question**	**Response**
Are you submitting this manuscript to a special series or article collection?	No
**Experimental design and statistics**<br><br>Full details of the experimental design and statistical methods used should be given in the Methods section, as detailed in our Minimum Standards Reporting Checklist. Information essential to interpreting the data presented should be made available in the figure legends.<br><br>Have you included all the information requested in your manuscript?	Yes
**Resources**<br><br>A description of all resources used, including antibodies, cell lines, animals and software tools, with enough information to allow them to be uniquely	Yes

identified, should be included in the Methods section. Authors are strongly encouraged to cite Research Resource Identifiers (RRIDs) for antibodies, model organisms and tools, where possible.<br><br>Have you included the information requested as detailed in our Minimum Standards Reporting Checklist?	
**Availability of data and materials**<br><br>All datasets and code on which the conclusions of the paper rely must be either included in your submission or deposited in publicly available repositories (where available and ethically appropriate), referencing such data using a unique identifier in the references and in the “Availability of Data and Materials” section of your manuscript.<br><br>Have you have met the above requirement as detailed in our Minimum Standards Reporting Checklist?	Yes

Manuscript clean
Click here to access/download;Manuscript;GIGA-D-18-00275_clean.docx
Click here to view linked References

The draft genomes of five agriculturally important African orphan crops

Yue Chang[1,2*], Huan Liu[1,2*], Min Liu[1,2*], Xuezhu Liao[1,2], Sunil Kumar Sahu[1,2], Yuan Fu[1,2], Bo Song[1,2], Shifeng Cheng[1,2], Robert Kariba[3], Samuel Muthemba[3], Prasad S. Hendre[3], Sean Mayes[5,6,7], Wai Kuan Ho[6,7], Presidor Kendabie[5], Sibo Wang[1,2], Linzhou Li[1,2], Alice Muchugi[3], Ramni Jamnadass[3], Haorong Lu[1,2], Shufeng Peng[1,2], Allen Van Deynze[3,4], Anthony Simons[3], Howard Yana-Shapiro[3,4], Xun Xu[1,2], Huanming Yang[1,2], Jian Wang[1,2], Xin Liu[1,2,8#.]

1. BGI-Shenzhen, Shenzhen 518083, China
2. China National GeneBank, BGI-Shenzhen, Shenzhen 518120, China
3. African Orphan Crops Consortium, World Agroforestry Centre (ICRAF), Nairobi, Kenya
4. University of California, 1 Shields Ave, Davis, USA, 95616
5. Plant and Crop Sciences, Biosciences, University of Nottingham, Sutton Bonington Campus, Loughborough, Leicestershire, LE12 5RD
6. Biosciences, University of Nottingham Malaysia Campus, Jalan Broga 43500 Semenyih, Selangor, Malaysia
7. Crops For the Future, Jalan Broga, 43500 Semenyih, Selangor, Malaysia
8. BGI-Fuyang, BGI-Shenzhen, Fuyang 236009, China

Correspondence address: Xin Liu (liuxin@genomics.cn)

* Equal contribution

ABSTRACT

Background: Continuous growth in the world population is expected to double the worldwide demand for food by 2050. Moreover, 88% of countries are currently facing a serious burden of malnutrition, especially in Africa and Southern & South-Eastern Asia. About 95% of the present food energy needs of humans are fulfilled by 30 species, within which wheat, maize and rice provide the majority of calories. Therefore, to diversify and stabilize global food supply, enhance agricultural productivity and tackle malnutrition in these countries, a greater utilization of neglected or underutilized local plants (generally so-called orphan crops, but also a few plants with special contribution to agriculture, such agroforestry and nutrient) could be a partial solution.

Findings: Here we present draft genome information from five agriculturally, biologically, medicinally and economically important underutilized plants in Africa, namely; *Vigna subterranea*, *Lablab purpureus*, *Faidherbia albida*, *Sclerocarya birrea*, and *Moringa oleifera*. The assembled genomes range in size from 217 to 654 Mb. In addition, we have predicted 31707, 20946, 28979, 18937, 18451 protein-coding genes in *V. subterranea*, *L. purpureus*, *F. albida*, *S. birrea* and *M. oleifera* respectively. We have further analyzed the expansion and contraction of selected gene families, and characterized root-nodule-symbiosis genes, transcription factors and starch biosynthesis related genes in these genomes.

Conclusions: This genome data will be useful to identify and characterize agronomically important genes and understand their mode of actions, enabling genomics-based, evolutionary studies, and breeding strategies for designing faster, focused and predictable crop improvement programs.

Keywords: Orphan crops; food security; whole-genome sequencing; transcriptome; root nodule symbiosis; transcription factors

BACKGROUND INFORMATION

The world's population is expected to reach 9.8 billion by 2050, thus ensuring a sustainable food supply to meet the energy and nutritional needs of the expanding population is one of the greatest global challenge [1]. Moreover, about 88% of the countries are currently facing a serious burden of malnutrition [2]. To overcome this burgeoning food and nutritional challenge, the utilization of potential crops (both model and non-model) plants appears to be a better choice. Throughout history, human beings have relied on astonishing varieties of plants for energy and nutrition: From 390,000 known plant species, it is estimated that around 5,000-7,000 plant species have been cultivated or collected for food [1, 2]. But, in the present century, less than 150 species are commercially cultivated for food purposes, and surprisingly 30 species alone provide 95% of the food energy needs of humans. More than half of the protein and calories which we obtain from plants are acquired from just three 'megacrops' – rice, wheat and maize [3]. This narrow range of dietary diversity is partly a result of decades of intensive research, focused on just a few species, which has successfully led to the production of high-yielding varieties of these major crops, usually cultivated under high input agricultural systems. However, we are now witnessing a drastic decrease in their yields in some regions and it has been questioned whether rice and wheat (in particular) are currently making enough breeding progress to meet the challenge. All three megacrops are high energy carbohydrate sources, but are limited in protein content. Even if these crops can meet the energy requirement of the increasing world population, they cannot meet the nutritional requirement for active health by themselves [2].

To diversify the global food supply, enhance the agricultural productivity and tackle malnutrition, it is necessary to diversify and focus more on crop plants that are utilized in rural societies as a local source of nutrition and sustenance, but have received little attention for crop improvement. These landraces tend to be locally adapted and can often provide a rich source of nutrition yet they largely been kept out of modern interventions. The goal of the African Orphan Crops Consortium (AOCC), an international public-private partnership is to sequence, assemble and annotate the genomes of 101 plants contributed to traditional African food supplies by 2020 (www.africanorphancrops.org). These neglected or orphan plants have been little studied by science, but are of major importance in many African countries. They are usually grown by smallholder farmers, either for consumption or local sale, and are a major food source for 600 million rural Africans [4, 5]. In this study, we sequenced and assembled draft genomes of five African orphan plant species (Figure 1), which are highly important to augment food and nutritional security in Africa.

Vigna subterranea (Bambara groundnut; NCBI taxon ID 115715) belonging to Fabacaeae family is a leguminoceous plant species which originated in West Africa, and is cultivated in Sub-Saharan areas, particularly Nigeria [6,7]. With good nitrogen-fixing ability, drought tolerance, on average the seeds contain 63% carbohydrate, 19% protein and 6.5% oil, thereby making bambara groundnut a complete food. The annual production of this species is about 165,000 tons in Africa, and yields are low because efforts to improve bambara has been negligible for many years [8]. The genomes of mung bean and adzuki bean have been published [9,10], which also belongs to *Vigna*

genus.

Moringa oleifera (Moringa; NCBI taxon ID 3735) is a highly nutritious, fast growing and drought tolerant tree, and is indigenous to Northern India, Pakistan and Nepal [11]. Presently, this species is ubiquitously distributed throughout tropical and subtropical countries, and in particular covers the major agro-ecological region in Nigeria. The leaves are rich in protein, minerals, beta-carotene and antioxidant compounds which are generally used as nutrition supplements and in traditional medicine. The seeds are used to extract oil and seed powder can be used for water purification [12, 13]. Various sources have had varying reports of Moringa production, India is the largest producer of Moringa with an annual production of 1.1–1.3 million tonnes of tender fruits from an area of 38,000 ha. In Limpompo province relatively small holder areas (0.25- 1ha) are under Moringa cultivation with seed yields of 50-100 kgs/ha^{-1} [14]. Prior to this study, a draft genome of *Moringa oleifera* from Yunnan (China) was also reported [15] with similar genome assembly size and gene numbers compared to our version.

Lablab purpureus (Dolichos bean or hyacinth bean; NCBI taxon ID 35936), a member of Fabaceae family is one of the most ancient (>3500 years) domesticated and multipurpose legume species used as an intercrop in livestock systems. Although it displays a large agro-morphological diversity in South Asia, its origin appears to be African [16]. It is rich in protein, has good nitrogen-fixing ability and displays high adaptability to a diverse range of environmental conditions [17]. There is limited production data available suggesting that yields are low. In southwestern parts of

Bangladesh, lablab is reported to have a total production area of approximately 48000 ha [16]. In other areas, Dolichos is reported to have a similarly relatively low production area, for example, Kenya, approx. 10,000 ha [18] and Karnataka India, 79000 ha [19].

Faidherbia albida (apple-ring acacia; NCBI taxon ID 138055) is the only tree species in genus *Faidherbia* (Fabaceae). Due to its distinctive key features like reverse phenology (leaves grow in the long dry season and shed during the rainy season) and nitrogen-fixing ability, *F. albida* has been planted as a key agroforestry species in traditional African farming systems for hundreds of years [20]. It originated in the Sahara or Eastern and Southern Africa, then spread over semi-arid tropical Africa, later spreading to the Middle East and Arabia. It is estimated that tree was cultivated over an area of 300,000 hectares during the last decade [21] The average pod production ranges from 6-135 kgs per tree in a year in the Sudanian zone. In Zimbabwe (Manapools) two trees averaged 161 kgs per tree in a year [22]. This yield per unit area is about 2000 to 3000kg/ha on assumption of about 20 mature trees per hectare [23].

Sclerocarya birrea (Marula; NCBI taxon ID 289766) belongs to the Anacardiaceae family, and is a traditional fruit tree found in southern Africa, mostly south of the Zambesi river [24]. The fruits are eaten fresh or used to produce juices and wine which has substantial socioeconomic and commercialization importance. The seed of the fruits are rich in nutrition and oil content (56%) and are often consumed raw. It is estimated that the total value of the commercial marula trade to the rural communities is worth USD $160,000 a year [25] with values per tree ranging from 315 kg (17,500 fruits) to 1643 kg (91,300 fruits) [25, 26]. A survey in Northcentral Namibia showed

that on an average there are 5.33 farm/household with a total number of 13,278 fruiting trees.

Considering the limited systematic efforts to improve the breeding of these crops, the availability of genomic data of these understudied tropical plants will give much needed impetus to conduct basic as well as applied translational research to improve and develop them as important food crops adapted for sustainable cultivation. These efforts are a vital instrument for direct or indirect nutrition of an increasing urban population in the regions these crops are grown.

DATA DESCRIPTION

Sample collection, library construction, and sequencing

The genomic DNA was extracted either from a tree (*Faidheriba albida*, *Moringa oleifera*) or from nursery plantlets (*Vigna subtarranea, Lablab purpureus, Sclerocarya birrea*) grown at the World AgroForestry Center (ICRAF) campus in Kenya using a modified CTAB method [27].

The extracted DNA was used to construct paired-end libraries (insert size from 170 to 800 bp) and mate-pair libraries (insert size larger than 2 kb) following the protocols from Illumina (San Diego, USA). Subsequently, the sequencing was performed on a HiSeq 2000 platform (Illumina, San Diego, CA, USA) with a strategy of shotgun sequencing to generate more than 100 Gb raw data for each species (Additional file1: Table S1). The data were filtered using SOAPfilter (v2.2) [28] as follows: (1) small insert size reads were discarded; (2) PCR duplicates and adapter contamination were

discarded; (3) reads with ≥30% low quality bases (quality score ≤ 15) were removed; (4) bases with low quality were trimmed from both sides of the reads; (5) reads with ≥ 10% uncalled ("N") bases were removed. Finally, more than 100× of high-quality reads were obtained for each species according to their estimated genome size (Additional file1: Table S1).

RNA for transcriptome sequencing was extracted from different tissues of *Vigna subterranea, Lablab purpureus, Faidherbia albida, Moringa oleifera*. The RNA was extracted using the PureLink RNA Mini Kit (Thermo Fisher Scientific, Carlsbad, CA, USA) according to the manufacturer's instructions. Libraries for the RNA samples were constructed following the manual of TruSeq RNA Sample Preparation Kit (Illumina, San Diego, CA, USA), and then sequenced on the Illumina HiSeq 2500 platform (paired-end, 100 base pair reads) and generated about 36 Gb of sequence data for each species. The data was then filtered with a strategy similar to DNA filtration, except a slight modification: (1) reads with ≥10% low quality bases (quality score ≤ 15) were removed; (2) reads with ≥ 5% uncalled ("N") bases were removed (Additional file 1: Table S2). We compiled all the transcriptome data from different tissues, and used the combined version to check the completeness of the WGS assembly.

Evaluation of genome size

Clean reads of the paired-end libraries were used to estimate genome sizes. (insert size 250 bp and 500 bp). The k-mer frequency distribution analysis was performed using the following formula: $Gen = Num*(Len - 17 + 1) / K\_Dep$, where *Num* represents the

read number of used reads, *Len* represents the length of read, *K* represents the length of k-mer and *K_Dep* refers to where the main peak is located in the distribution curve [29]. In this analysis, K-mer distributions of *F. albida, S. birrea,* and *M. oleifera* showed two distinct peaks (Additional file1: Figure S1), where the second peak was confirmed as the main one for each of the species. The genome size of *V. subterranea*, *L. purpureus*, *F. albida*, *S. birrea* and *M. oleifera* was predicted as 550, 423, 661, 356 and 278 Mb, respectively (Additional file1: Table S3).

***De novo* assembling of genomes**

For *de novo* genome assembly, SOAPdenovo2 (SOAPdenovo2, RRID:SCR_014986) [28] was used for constructing contigs, followed by scaffolding, and finally gap filling. To build a contig, libraries ranging from 170 to 800 bp were used to construct de Bruijn graphs with the parameters "pregraph -d 2 -K 55, and contigs were subsequently formed with the parameters "contig -g -D 1" to delete links with low coverage. In the scaffolding step, paired-end and mate-pair information were used to order the contigs with parameters "scaff -g -F" and "map -g -k 55". Finally, to fill the gaps within scaffolds, GapCloser version 1.12 (GapCloser, RRID:SCR_015026) [28] was used with the parameters "-l 150 -t 32" using the pair-end libraries. Finally, a total assembled length of 535.05, 395.47, 653.73, 330.98, and 216.76 Mb was obtained for *V. subterranea*, *L. purpureus, F. albida, S.birrea* and *M. oleifera* genomes, respectively (Table 1). This accounted for approximately 97.3%, 93.5%, 98.9%, 92.9% and 77.9% of their estimated genome size, respectively.

Genome evaluation

The completeness of the genome assemblies was assessed with BUSCO version 3.0.1 (Benchmarking Universal Single-Copy Orthologues), (BUSCO, RRID:SCR_015008) [30]. From the 1,440 core embryophyta genes, 1,326 (92.1%), 1,341 (93.2%), 1,315 (91.3%), 1,384 (96.1%) and 1,297 (90.1%) were identified in the *V. subterranea*, *L. purpureus*, *F. albida*, *S. birrea* and *M. oleifera* assemblies, with 1,244 (86.4%), 1,258 (87.4%), 1,231 (85.5%), 1,352 (93.9%) and 1.278 (88.8%) genes being complete (Table 2), respectively.

To evaluate the completeness of genes in the assemblies, unigenes were generated from the transcript data of each species using Bridger software with the parameters "-kmer_length 25 -min_kmer_coverage 2" [31], and then aligned to the corresponding assembly using BLAT (BLAT, RRID:SCR_011919) [32]. The results indicated that each of the assemblies covered about 90% of the expressed unigenes, suggesting that the assembled genomes contained a high percentage of expressed genes (Table 3).

In order to confirm the accuracy of the assemblies, some of the paired-end libraries were mapped to the genome assemblies and the sequencing coverage was calculated using SOAPaligner, version 2.21 (SOAPaligner/soap2 , RRID:SCR_005503) [33]. The sequencing coverage showed that > 99% of the bases had a sequencing depth of more than 10 x and confirmed the accuracy at the base level (Additional file1: Figure S2). The GC content and average depth were also calculated with 10 kb non-overlapping windows, the distribution of GC content indicated a relatively pure single genome without contamination or GC bias (Additional file1: Figure S3). Moreover, the GC

content of each sequenced genome was also compared to that of their related species. As expected, the close peak positions showed the related species were similar in GC content (Additional file1: Figure S4).

Repeat annotation

Repetitive sequences were identified using RepeatMasker (version 4-0-5) [34], with a combined Repbase and a custom library obtained through careful self-training. The custom library composed of three parts: the MITE (miniature inverted repeat transposable elements), LTR (long terminal repeat) and an extensive library which was constructed as follows. First, the annotated MITE library was created using MITE-hunter [35] with default parameters. Then, the LTR elements with a length of 1.5 kb to 25 kb, and two terminal repeats ranging from 100 bp to 6000 bp with >= 85% similarity was constructed using LTRharvest [36] integrated in Genometools (version 1.5.8) [37] with parameters "-minlenltr 100 -maxlenltr 6000 -mindistltr 1500 -maxdistltr 25000 -mintsd 5 -maxtsd 5 -similar 90 -vic 10". Subsequently, we used several strategies to filter the candidates, e.g. *i.* presence of intact PPT (poly purine tract) or PBS (primer binding site) sites [38] using the eukaryotic tRNA library (http://gtrnadb.ucsc.edu/), *ii.* removal of contamination from local gene clusters and tandem local repeats by inspecting 50 bases of the upstream and downstream LTR flanks using MUSCLE (MUSCLE, RRID:SCR_011812) [39] for a minimum of 60% identity *iii.* removal of nested LTR candidates with other types of the elements. Exemplars for the LTR library were extracted from the filtered candidates using a cutoff of 80% identity in 90% of the

sequence. Furthermore, the regions annotated as LTRs and MITEs in the genome were masked, and then put into RepeatModeler version 1-0-8 (RepeatModeler, RRID:SCR_015027) to predict other repetitive sequences for the extensive library. Finally, the MITE, LTR and extensive libraries were integrated into the custom library, which was combined with the Repbase library and taken as an input for RepeatMasker to identify and classify genome-wide repetitive elements. The pipeline identified 205,189,285 (38.35% of the genome length), 147,050,327 (37.18%), 358,653,534 (54.86%), 149,551,125 (45.18%), and 87,944,150 (40.57%) bases of non-redundant repetitive sequences in *V. subterranea*, *L. purpureus*, *F. albida*, *S. birrea* and *M. oleifera* respectively. LTR elements were predominant, taking up to 19.8%, 23.8%, 44.6%, 38.8%, 22.7% of each genome, respectively (Table 4).

Gene prediction

Repetitive regions of the genome were masked before gene prediction. The structures of protein-coding genes were predicted using the MAKER-P pipeline (version 2.31) [40] based on RNA, homologous and *de novo* prediction evidence. For RNA evidence, the clean transcriptome reads were assembled into inchworms using Trinity version 2.0.6 [41], and then provided to MAKER-P as EST evidence. For homologous comparison, the protein sequences from the model plant *Arabidopsis thaliana* and related species of each sequenced species were downloaded and provided as protein evidence. The related species we used for homologous evidence are listed below: *V. subterranea*: (*Arachis duranensis*, *Arachis ipaensis*, *Glycine max*, *Lotus japonicus*,

Medicago truncatula, *Vigna angularis*); *L. purpureus*: (*A. duranensis*, *Cajanus cajan*, *G. max*, *M. truncatula*, *Phaseolus vulgaris*, *Vigna angularis*); *F. albida*: (*Cajanus cajan*, *V. angularis*, *L. japonicus*, *P. vulgaris*, *M. truncatula*, *G. max*); *S. birrea*: (*Actinidia chinensis*, *Musa acuminata*); *M. oleifera*: (*G. max*, *Oryza sativa*, *Populus trichocarpa*, *Sorghum bicolor*).

For evidence from *de novo* prediction, a series of training sets were made to optimize different *ab initio* gene predictors. Initially, a set of transcripts were generated by a genome-guided approach using Trinity with parameters "--full_cleanup --jaccard_clip --genome_guided_max_intron 10000 --min_contig_length 200". The transcripts were then mapped back to the genome using PASA (version 2.0.2) [42] and a set of gene models with real gene characteristics (e.g. size and number of exons/introns per gene, features of splicing sites) were generated. The complete gene models were picked for training Augustus [43]. Genemark-ES (version 4.21) [44] was self-trained with default parameters. The first round of MAKER-P was run based on the evidence as above with default parameters except with "est2genome" and "protein2genome" were set to "1", yielding only RNA and protein-supported gene models. SNAP [45] was then trained with these gene models. Default parameters were used to run the second and final round of MAKER-P, producing the final gene models.

Finally, 31,707, 20,946, 28,979, 18,937 and 18,451 protein-coding genes were identified in *V. subterranea*, *L. purpureus*, *F. albida*, *S. birrea* and *M. oleifera*. Compared to the other sequenced species in the same genus [9, 10], the gene number of *V. subterranea* is more than that of mung bean (22,427) but less than that of adzuki

bean (34,183). Various gene structure parameters were compared to the related species of each sequenced genome as summarized in table 5 and additional file1: Figure S5. BUSCO evaluation showed that at least 85% of 1,440 core genes could be identified across all the species, suggesting an acceptable quality of gene annotation for the five sequenced genomes (Additional file1: Table S4).

Furthermore, non-coding RNA genes in the sequenced genomes were also annotated. The ribosomal RNA (rRNA) genes were searched using BLAST against the *A. thaliana* rRNA database, or by searching for microRNAs (miRNA) and small nuclear RNA (snRNA) against the Rfam database (Rfam, RRID:SCR_004276) (release 12.0) [46]. Further, tRNAscan-SE (tRNAscan-SE, RRID:SCR_010835) was used to scan for transfer RNAs (tRNA) [47]. The result is summarized in Table 6.

Functional annotation of protein-coding genes

The functional annotation of protein-coding genes was based on sequence similarity and domains conservation by aligning predicted amino acid sequences to public databases. The protein-coding genes were first searched against protein sequence databases for best matches, such as KEGG (KEGG, RRID:SCR_012773) [48], NR database (NCBI), COG [49], SwissProt and TrEMBL [50] using BLASTP with an E-value cut-off of 1e-5. Then, InterProScan 55.0 (InterProScan, RRID:SCR_005829) [51] was used as an engine to identify domains and motifs based on Pfam (Pfam, RRID:SCR_004726) [52], SMART (SMART, RRID:SCR_005026) [53], PANTHER (PANTHER, RRID:SCR_004869) [54] , PRINTS (PRINTS, RRID:SCR_003412) [55]

and ProDom (ProDom, RRID:SCR_006969) [56]. In total, 98.0%, 98.2%, 93.6%, 98.1% and 98.8% of genes in *V. subterranea*, *L. purpureus*, *F. albida*, *S.birrea* and *M. oleifera* were functionally annotated. Among the unannotated genes, there are 400, 305, 1514, 293 and 172 genes specific in *V. subterranea*, *L. purpureus*, *F. albida*, *S. birrea* and *M. oleifera* respectively (Table 7).

Gene family construction

Protein and nucleotide sequences from the five sequenced species and 9 other species (*A. thaliana*, *Carica papaya*, *Citrus sinensis*, *G. max*, *M. truncatula*, *O. sativa*, *P. vulgaris*, *S. bicolor*, *Theobroma cacao*) were retrieved to construct gene families using OrthoMCL software [57] based on an all-versus-all BLASTP alignments with an E-value cutoff of 1e-5. A total of 609, 104, 499, 205 and 150 gene families were found specific to *V. subterranea*, *L. purpureus*, *F. albida*, *S. birrea* and *M. oleifera*, respectively (Additional file1: Table S5).

Furthermore, the 10,103 gene families of *V. subterranea*, *L. purpureus*, *F. albida*, *M. truncatula* and *G. max* were clustered (Figure 2A). There were 1,105 orthologous families shared by the four Papilionoideae species, while 808 gene families containing 1,966 genes were specific to *F. albida*, 281 gene families containing 538 genes were specific to *L. purpureus*, 789 gene families containing 3,118 genes were specific to *V. subterranea.*

Moreover, 8,184 gene families of *S. birrea*, *M. oleifera*, *C. papaya*, *C. sinensis* and *T. cacao* were clustered (Figure 2B), of which 365 gene families containing 798 genes

were specific to *M. oleifera*, 362 gene families containing 796 genes were specific to *S. birrea*, respectively. The enrichment analysis on KEGG pathway of the paralogs genes were also calculated (Additional file1: Table S6, S7). The functional annotation revealed that they mainly correspond to the carbon fixation, zeatin biosynthesis, glyoxylate and dicarboxylate metabolism in *V. subterranea*. However, for *L. purpureus*, the fatty acid elongation pathway was enriched. While in *F. albida*, the pathways corresponding to the plant-pathogen interaction and cyanoamino acid metabolism were enriched. In *S. birrea*, the pathways of plant-pathogen interaction, starch and sucrose metabolism, fatty acid biosynthesis were enriched. In *M. oleifera*, the pathways related to fatty acid and diterpenoid biosynthesis, cyanoamino acid metabolism were enriched. The enrichment analysis on GO of paralogs genes were ion binding, metabolic process, disease resistance, cell component, biological process in *V. subterranea*, *L. purpureus*, *F. albida*, *M. oleifera*, and *S. birrea* respectively.

Phylogenetic analysis and divergence time estimation

We identified 141 single-copy genes in the 14 species used for the above analysis, and subsequently used them to build a phylogenetic tree. Coding DNA sequence (CDS) alignments of each single-copy family were generated following the protein sequence alignment with MUSCLE (MUSCLE, RRID:SCR_011812) [39]. The aligned CDS sequences of each species were then concatenated to a supergene sequence. The phylogenetic tree was constructed with PhyML-3.0 (PhyML, RRID:SCR_014629) [58] with the HKY85+gamma substitution model on extracted four-fold degenerate sites.

Divergence time was calculated using the Bayesian relaxed molecular clock method with MCMCTREE in PAML (PAML, RRID:SCR_014932) [59], based on the published calibration times (39-59 Mya between *M. truncatula* and the main branch of legumes, 15-30 Mya between *G. max* and *P. vulgaris*, and 83-90 Mya between *T. cacao* and *A. thaliana*) [10, 60]. Based on the tree constructed by single-copy-family genes, the divergence time between *F. albida* and Papilionoideae was predicted to be 79.1 (70.0-87.0) Mya, which is a little different from the previous predicted origin of legumes based on two gene markers (matk and rbcL) [61]. Whereas, the divergence time between *M. oleifera* and *C. papaya* was predicted to be 65.4 (59.2-71.1) Mya, and 67.9 (53.6-77.3) Mya between *S. birrea* and *C. sinensis* (Figure 1). Subsequently, to evaluate the gene gain and loss, CAFE (CAFE, RRID:SCR_005983) [62] was employed to estimate the universal gene birth and death rate λ (lambda) under a random birth and death model with the maximum likelihood method. The results for each branch of the phylogenetic tree were estimated and represented in Figure 1. Enrichment analysis on GO and pathway of genes in expanded families in the lineage of each sequenced species were also calculated (Additional file1: Table S8, S9). Terms related to energy and nutrient metabolism were commonly distributed in the enrichment output of *V. subterranean, L. purpureus, M. oleifera and S. birrea*, such as proton-transporting two-sector ATPase complex, cyclase activity, nutrient reservoir activity and carbohydrate derivative binding. While in *F. albida*, expansion of gene families were related to signal transfer or regulation, such as signaling receptor activity, phosphatase regulator activity regulation of response to stimulus and so on. Furthermore, regulatory

factors (*GLABRA3*, *ENHANCER OF GLABRA 3*, *AUX1*, *LAX2*, and *LAX3*) [63-65] related to the formation of root hair and lateral root were identified in these families. As a traditional agroforestry tree in Africa, *F. albida* was previously reported to have a root system architecture (RSA) displaying severe variations to different environmental factors (soil depth, nutrient amount, or water reservoirs) [66], suggesting its adaptability to the complex environment, which requires signal transferring and regulation. The result of the GO enrichment analysis was consistent with the biological characteristic of *F. albida*.

Mining of transcription factors

The transcription factors (TFs) in the sequenced species, were identified using protein sequences of plant TFs from the plant transcription factor database (http://planttfdb.cbi.pku.edu.cn/index.php) by BLASTP search with an e-value cutoff of 10E−10, a minimum identity of 40% and a minimum query coverage of 50%. About 59 TF families were (Additional file 2: Table S14) were revealed across the genes in *M. truncatula, G. max, P. vulgaris, C. papaya, C. sinensis,* and the five sequenced species. Among these TFs, bHLH, NAC, ERF, MYB related, C2H2, MYB, WRKY, bZIP, FAR1, C3H, B3, G2-like, Trihelix, LBD, GRAS, M-type MADS, HD-ZIP, MIKC_MADS, HSF, GATA were found in major abundance (Figure 4).

Identification of protein, starch, and fatty acid biosynthesis related genes

Using the amino acid, starch and fatty acid synthesis genes in soybean [10, 67] as bait, we performed an ortholog search in *V. subterranea*, *L. purpureus*, *F. albida*, *S. birrea*,

M. oleifera, *G. max*, *T. aestivum*, *Z. mays* and *O. sativa* (Additional file 1: Table S10,Table S11, Table S12, Table S13). *V. subterranea* is a good source of resistance starch (RS) [68], which has the potential to protect against diabetes and reduce the incidence of diarrhea and other inflammatory bowel disease [69]. It is known that high amylose can contribute to RS, and previously studies have shown that deficiency in *SSIIIa* (soluble starch synthase gene) will decrease amylopectin biosynthesis and increase the amylose biosynthesis by GBSSI encoded by the *Wx* gene in *indica* [70]. Down-regulation of soluble starch synthase (SS) *SSIIa* and of *SBE* will lead to higher RS amount in barley [71]. Interestingly, two out of four granule-bound starch synthase GBSS in *V. subterranea* underwent expansion, suggesting its vital role in controlling starch synthesis (Figure 5) at the transcriptional and post-transcriptional level. Moreover, no expansion in GBSS was observed among *L. purpureus*, *F. albida*, *S. birrea* and *M. oleifera* genomes. Meanwhile the soluble starch synthase SS in *V. subterranea* were not expanded. Therefore, we speculate that the expansion of GBSS might be the reason why *V. subterranea* is rich in resistance starch. Similarly, difference in the copy numbers of choline kinase, which is a key factor in fatty acid synthesis and storage (7) was found to be different from the other three legumes including *G. max* [*F. albida* (4), *L. purpureus* (2), *G. max* (5) and two orphan species (*S. birrea* (1), *M. oleifera* (3)]. The choline kinase is the first enzyme in the cytidine diphosphate-choline pathway which is involved in lecithin biosynthesis [72, 73]. Based on these observations we inferred that the all the necessary factor to synthesize lecithin are present in V. *subterranea*. However, we still lack the gene expression data about the

GBSS and choline kinase genes in these the five species. More transcriptomic analysis and chemical test are still required to dig into their nutrition metabolism in future.

Identification of root nodule symbiosis pathway

Legumes (Fabaceae) are well known for their ability to fix nitrogen, which is an important trait to replenish nitrogen supply in soil and agricultural systems. Furthermore, being a part of human food production chain, They have a major impact on global nitrogen cycle. Nitrogen-fixing plants can do this through root nodule symbiosis (RNS) using symbiotic nitrogen-fixing bacteria. In a previous report, RNS was revealed to be restricted to Fabales, Fagales, Cucurbitales, and Rosales that together form the monophyletic nitrogen-fixing clade, thus suggesting a predisposition event in their common ancestor, which enabled the subsequent evolution [74]. Despite this genetic predisposition, many members of the nitrogen-fixing clade are non-fixer, within the legumes [75]. This has led to the question whether the nodulation trait evolved independently in a convergent manner, or originated from a single evolutionary event followed by multiple losses. However, the answers to the above questions cannot be explained with the help of current genomic approaches, as the genomic information of nodulating species at present is limited to a single subfamily (Papilionoideae) in Fabaceae. Although the Mimosoideae subfamily under Fabaceae also contains nitrogen-fixing species, none of its members have been genome-sequenced. In this analysis, we identified 16 root nodulation symbiosis signal (Sym) pathway genes in three legumes (*V. subterranea*, *L. purpureus*, and *F. albida*) and two non-legumes (*S.*

birrea and *M. oleifera*). First, we collected the protein sequences of previously reported genes in the Sym pathway of *L. japonicus* and *M. truncatula* [76] (Figure 3). Using these sequences as bait, the Sym genes in *V. subterranea*, *L. purpureus*, *F. albida*, *S. birrea*, and *M. oleifera* were predicted through reciprocal best hits generated by BLASTP search with an E-value of 1e-5 (Table 8). To verify the prediction with syntenic analysis, the 'all vs all' BLASTP results were subjected to MCSCANX [77] with default parameters to generate the syntenic blocks. The result showed that most of the components in the pathway are conserved in the three legumes, except *MtNFP*/*LjNFR5*, *LjCASTOR*, *CCaMK*, *MtCRE1*/*LjLHK1*, and *NF-YA2*. While many components were missing in the non-legumes. Among the three legumes, the orthologous genes of *MtNFP/LjNFR5*, *LjCASTOR* and *MtIPD3/LjCYCLOPS* were absent in *F. albida*. As previously reported, the expression of *NIN* is lower in the *ipd3*-mutant line [78], and the analysis of the *M. truncatula* mutant C31 showed that the Nod Factor Perception (NFP) gene plays an essential role in Nod factor perception at early stages of the symbiotic interaction [79]. Meanwhile, the function of *IPD3* was proved to be partly redundant, which means other proteins phosphorylated by CCaMK probably could partly do the job when *IPD3* is absent [78]. The difference in the components within RNS pathway (Table 8) together with the relatively weak nitrogen-fixing ability [80] of *F. albida* thus make itself a good reference in the research of RNS diversification.

Conclusion

This comprehensive study reports the sequencing, assembly, and annotation of five genomes of underutilized plants in Africa along with details of their key evolutionary features. The draft genomes of these species will serve as an important complementary resource for the non-model food crops especially the leguminous plants, and will be valuable for both agroforestry and evolutionary research. Improvement in these former underutilized plants using genomics-assisted tools and methods could bring food security for millions of people.

Availability of supporting data

The raw data from our genome project was deposited in the SRA (Sequence Read Archive) database of National Center for Biotechnology Information with Bioproject ID PRJNA453822 and PRJNA474418. The assembly and annotation of the five genomes and other supporting data, including BUSCO results, are available in the *GigaScience* GigaDB repository [81].

Abbreviations

AOCC: African Orphan Crops Consortium; BLAST: Basic Local Alignment Search Tool; BUSCO: Benchmarking Universal Single-Copy Orthologues; CDS: Coding DNA sequence; CFU: The Conservation Farming Unit; LTR: long terminal repeat; TF: transcription factors; MITE: miniature inverted repeat transposable elements; NCBI: National Center for Biotechnology Information; PBS: primer binding site; PPT: poly

purine tract.

Author contributions

XL, XX, HY, JW, PSH, RJ, AV and YC conceived the project. They supervised the respective components: AOCC-ICRAF: DNA extraction, sample logistics and collection; BGI: data generation and analyses of the study. YC supervised the analyses. RK and SM collected and extracted the DNA and RNA. SB and FY performed the genome assembly. ML, XZL, SBW and LZL performed the genome annotation, gene family analysis and identification of genes related to root growth and root nodule symbiosis. YC, ML, XZL performed the phylogenetic analysis. YC, HL, SKS, PSH and AV wrote the manuscript. HRL and SFP sequenced the samples. SM, WKH, AM, PSH, JW, HMY revised the manuscript. All authors read, edited and approved the final manuscript.

Acknowledgments

This work was supported by the Shenzhen Municipal Government of China, (No. JCYJ20150831201643396 and No. JCYJ20150529150409546), as well as the funding from the State Key Laboratory of Agricultural Genomics (No. 2011DQ782025), and Guangdong Provincial Key Laboratory of Genome Read and Write (No. 2017B030301011). This work is part of 10KP project led by BGI-Shenzhen and China National GeneBank.

Table 1: Statistics of the final *de novo* genome assembly in *V. subterranea*, *L. purpureus*, *F. albida*, *S. birrea* and *M. oleifera*.

		V. subterranea		***L. purpureus***		***F. albida***		***S. birrea***		***M. oleifera***	
		**Contig**	**Scaffold**	**Contig**	**Scaffold**	**Contig**	**Scaffold**	**Contig**	**Scaffold**	**Contig**	**Scaffold**
**Length (bp)**	N90	3,804	75,271	785	860	8,254	95,167	3,661	21,833	6,676	57,837
	N80	7,872	197,296	8,009	61,348	16,321	251,730	7,649	82,385	16,503	241,828
	N70	11,464	325,826	16,144	205,392	24,165	380,587	11,885	155,416	25,754	441,152
	N60	15,122	474,616	24,010	359,168	32,440	534,880	16,393	243,236	35,081	644,014
	N50	19,154	640,666	32,223	621,373	42,029	692,039	21,349	335,449	45,268	957,246
	N40	23,828	865,081	42,690	950,808	53,479	881,230	26,914	485,585	58,406	1,446,587
	N30	29,382	1,133,817	54,401	1,489,002	69,167	1,197,388	33,914	705,409	74,710	1,878,891
	N20	36,928	1,503,436	70,790	1,971,744	92,147	1,501,241	43,984	1,098,843	96,626	2,565,629
	N10	49,695	2,049,645	95,643	2,606,483	139,388	1,925,526	62,875	2,089,533	136,952	3,296,678
**Number**	N90	29,245	1,087	26,272	9,409	16,834	1,132	17,585	1,537	5,524	366
	N80	20,188	664	9,869	715	11,420	727	11,678	787	3,574	191
	N70	14,829	453	6,576	366	8,198	514	8,313	499	2,542	125
	N60	10,943	315	4,630	222	5,898	370	6,001	332	1,833	84
	N50	7,932	220	3,244	138	4,151	263	4,277	214	1,295	56
	N40	5,532	147	2,204	86	2,791	179	2,929	131	876	37
	N30	3,590	93	1,403	52	1,728	114	1,857	74	553	24
	N20	2,024	52	776	29	912	64	1,012	36	300	13
	N10	806	22	306	12	326	26	387	12	112	6
Maximum length		148,612	3,684,321	240,194	5,699,750	529,842	4,746,824	227,874	5,850,796	449,426	4,637,711
Total length		512,516,846	535,052,523	385,303,786	395,472,305	644,456,383	653,726,905	322,977,033	330,983,508	213,739,255	216,759,177
Total number>=100bp		104,575	65,586	135,039	118,976	75,572	51,470	64,158	40,280	29,972	22,329
Total number>=2000bp		35,465	2,920	15,984	4,265	26,459	5,758	22,172	4,852	8,300	2,166

Percentage of N content (%)	4.21	2.57	1.42	2.42	1.39

Table 2: Completeness evaluation of genome assembly using BUSCO database in five species.

BUSCOs	*V. subterranea*		*L. purpureus*		*F. albida*		*S. birrea*		*M. oleifera*	
	**NO.**	**P,%**	**NO.**	**P,%**	**NO.**	**P,%**	**NO.**	**P,%**	**NO.**	**P,%**
Complete single copy	1,244	86.39	1,258	87.40	1,231	85.50	1352	93.90	1,278	88.80
Complete duplicated	82	5.69	83	5.80	84	5.80	32	2.20	19	1.30
Fragmented	28	1.94	20	1.40	34	2.40	21	1.50	23	1.60
Missing	86	5.97	79	5.40	91	6.30	35	2.40	120	8.30
Total	1440	/	1440	/	1440	/	1440	/	1440	/

Table 3: The gene coverage of the candidate species based on transcriptome data

Species	Dataset	Number	Total Length (bp)	Base by (%)	Coverage Assembly	Sequence coverage by assembly (%)
V. subterranea	All	**116,223**	161,077,155		89.61	98.21
	>200bp	116,223	161,077,155		89.61	98.21
	>500bp	72,139	147,068,299		89.03	98.00
	>1000bp	47,952	129,884,929		88.33	97.52
L. purpureus	All	**86,867**	80,837,182		93.59	99.25
	>200bp	86,867	80,837,182		93.59	99.25
	>500bp	41,252	66,764,786		92.94	99.18
	>1000bp	24,627	55,074,989		92.32	99.02
F. albida	All	**50,294**	46,650,067		93.62	98.85
	>200bp	50,294	46,650,067		93.62	98.85
	>500bp	26,352	39,282,694		93.32	99.05
	>1000bp	15,569	31,560,858		92.78	98.95
M. oleifera	All	**60964**	57114636		88.98	92.16
	>200bp	60964	57114636		88.98	92.16
	>500bp	29581	47523018		88.85	92.69
	>1000bp	18322	39528310		88.70	92.99

Table 4: The proportion of different classes of repeats (%) in five species.

Repeat Type	*V. subterranea*		*L. purpureus*		*F. albida*		*S. birrea*		*M. oleifera*	
	**% in genome**	**Length (bp)**	**% in genome**	**Length(bp)**	**% in genome**	**Length (bp)**	**% in genome**	**Length (bp)**	**%in genome**	**Length (bp)**
SINE	0	313	0.005	19,444	< 0.01	1,966	0.02	69,836	0.11	248,569
LINE	0.25	1,387,567	0.45	1,784,785	0.91	6,003,271	0.19	647,579	1.83	3,970,802
LTR	19.77	105,828,735	23.78	94,062,428	44.65	291,901,514	38.78	128,362,381	22.69	49,200,625
DNA	7.15	38,294,871	4.76	18,851,402	4	26,164,519	1.76	5,829,982	5.81	12,599,607
Satellite	0.01	71,679	0.02	107,451	0.01	110,749	0	18,597	0.74	1,623,399
Simple repeat	0.35	1,922,719	0.2	821,773	0.04	308,481	0.04	153,135	0.29	630,662
Others	11.94	63,926,350	8.95	35,400,400	6.48	42,426,306	5.11	16,918,179	10.35	22,439,026
Total	**38.35**	**205,189,285**	**37.18**	**147,050,327**	**54.86**	**358,653,534**	**45.18**	**149,551,125**	**40.57**	**87,944,150**

Table 5. Various gene structure parameters of *V. subterranea*, *L. purpureus*, *F. albida*, *M. oleifera* and *S. birrea*.

	V. subterranea	*L. purpureus*	*F. albida*	*M. truncatula*	*G. max*
Protein-coding gene number	31,707	20,946	28,979	50,358	55,137
Mean gene length (bp)	3,287	3,696	3,396	2,334	3,144
Mean cds length (bp)	1,163	1,276	1,207	986	1,169
Mean exons per gene	5	5	5	4	5
Mean exon length (bp)	222	239	226	243	232
Mean intron length (bp)	501	557	504	440	488

	S. birrea	*M. oleifera*	*C. papaya*	*T. cacao*	*C. sinensis*
Protein-coding gene number	18,937	18,451	24,107	41,951	35,182
Mean gene length (bp)	3,561	3,308	2,531	3,684	3,797
Mean cds length (bp)	1,343	1,238	962	1,323	1,424
Mean exons per gene	6	5	4	6	6
Mean exon length (bp)	239	232	223	223	237
Mean intron length (bp)	479	478	473	479	475

Table 6. Annotation of non-coding RNA genes in *V. subterranea*, *L. purpureus*, *F. albida*, *S. birrea* and *M. oleifera* genome.

		miRNA	tRNA	rRNA					snRNA				Total
				**Total rRNA**	**18S**	**28S**	**5.8S**	**5S**	**Total snRNA**	**CD-box**	**HACA-box**	**splicing**	
V. subterranea	**Copy (w)**	102	756	1,080	55	62	17	946	523	327	47	149	2,461
	**Average length (bp)**	122	75	124	560	126	124	99	117	100	133	149	110
	**Total length (bp)**	12,466	56,639	134,185	30,798	7,793	2,110	93,484	61,006	32,643	6,236	22,127	264,296
	**% of genome**	0.0023%	0.0106%	0.0251%	0.0058%	0.0015%	0.0004%	0.0175%	0.0114%	0.0061%	0.0012%	0.0041%	0.0494%
L. purpureus	**Copy (w)**	109	611	633	213	283	53	84	457	278	48	131	1,810
	**Average length (bp)**	123	75	227	446	121	135	84	118	97	133	158	136
	**Total length (bp)**	13,398	45,748	143,466	95,074	34,186	7,177	7,029	54,029	26,915	6,371	20,743	256,641
	% of genome	0.0034%	0.0116%	0.0363%	0.0240%	0.0086%	0.0018%	0.0018%	0.0137%	0.0068%	0.0016%	0.0052%	0.0649%
F. albida	**Copy(w)**	126	458	1,008	25	26	6	951	1,996	1,836	42	118	3,588
	**Average length (bp)**	122	75	107	321	118	118	101	108	106	132	138	103
	**Total length (bp)**	15,364	34,388	107,518	8,034	3,063	710	95,711	216,482	194,676	5,548	16,258	373,752
	**% of genome**	0.0024%	0.0053%	0.0164%	0.0012%	0.0005%	0.0001%	0.0146%	0.0331%	0.0298%	0.0008%	0.0025%	0.0572%
S. birrea	**Copy (w)**	106	564	313	80	57	16	160	841	638	34	169	1,824
	**Average length (bp)**	122	75	142	240	113	103	106	115	105	124	148	113
	**Total length (bp)**	12,899	42,181	44,378	19,239	6,460	1,644	17,035	96,517	67,216	4,217	25,084	195,975
	**% of genome**	0.0039%	0.0127%	0.0134%	0.0058%	0.0020%	0.0005%	0.0051%	0.0292%	0.0203%	0.0013%	0.0076%	0.0592%
M. oleifera	**Copy (w)**	111	1,241	8,406	3,256	3,808	1,182	160	229	119	38	72	9,987

**Average length (bp)**	119	75	309	608	113	150	69	119	97	132	147	622
**Total length (bp)**	13,161	93,620	2,598,079	1,979,080	430,280	177,612	11,107	27,158	11,578	4,999	10,581	2,732,018
**% of genome**	0.0061%	0.0432%	1.1986%	0.9130%	0.1985%	0.0819%	0.0051%	0.0125%	0.0053%	0.0023%	0.0049%	1.2604%

Table 7. Statistics of functional annotation of protein-coding genes in the *V. subterranea*, *L. purpureus*, *F. albida*, *S. birrea* and *M. oleifera* genome.

	V. subterranea		***L. purpureus***		***F. albida***		***S. birrea***		***M. oleifera***	
	**Number of genes**	**Percentage (%)**	**Number of genes**	**Percentage (%)**	**Number of genes**	**Percentage (%)**	**Number of genes**	**Percentage (%)**	**Number of genes**	**Percentage (%)**
Nr-Annotated	31,013	97.81	20,540	98.06	27,021	93.24	18,547	97.94	18,203	98.65
Swissprot-Annotated	22,496	70.95	15,905	75.93	21,247	73.32	15,513	81.92	15,109	81.88
KEGG-Annotated	22,141	69.83	14,699	70.18	20,184	69.65	14,623	77.22	14,044	76.11
COG-Annotated	10,814	34.11	7,854	37.50	10,526	36.32	7,715	40.74	7,662	41.52
TrEMBL-Annotated	30,964	97.66	20,489	97.82	26,828	92.58	18,477	97.57	18,193	98.60
Interpro-Annotated	22,744	71.73	18,911	90.28	25,401	87.65	15,537	82.05	15,134	82.02
GO-Annotated	18,894	59.59	13,811	65.94	15,182	52.39	11,505	60.75	11,877	64.37
Overall	31,074	98.00	20,574	98.22	27,118	93.58	18,573	98.08	18,236	98.83
Unannotated	633	2.00	372	1.78	1,861	6.86	364	1.92	216	1.17

Table 8: The nitrogen fixation orthologous in *V. subterranea, L. purpureus, F. albida, M. oleifera and S. birrea*.

Gene	*V. subterranea*	*L. purpureus*	*F. albida*	*M. oleifera*	*S. birrea*
MtLYK3/LjNFR1	Vigsu176S22567_VIGSU	Labpu216S12485_LABPU	Faial2789S13350_FAIAL	——	——
MtNFP/LjNFR5	Vigsu1898S04417_VIGSU	Labpu54S03611_LABPU	——	——	Sclbi409S02347_SCLBI
MtDMI2/LjSYMRK	Vigsu107959S16599_VIGSU	Labpu4785S15752_LABPU	Faial1833S08172_FAIAL	Morol36160S02362_MOROL	Sclbi59955S15146_SCLBI
LjCASTOR	Vigsu108012S17109_VIGSU	Labpu27S13484_LABPU	——	——	———
MtHMGR1	——	——	——	——	———
MtDMI1/LjPOLLUX	Vigsu108496S19983_VIGSU	Labpu4332S15101_LABPU	Faial363S16033_FAIAL	Morol36085S07630_MOROL	———
NSP1	Vigsu2922S08781_VIGSU	Labpu723S04373_LABPU	Faial1104S01086_FAIAL	Morol36102S01150_MOROL	Sclbi5005S02593_SCLBI
NSP2	Vigsu107793S01507_VIGSU	Labpu887S08157_LABPU	Faial757S23006_FAIAL	Morol36224S03158_MOROL	Sclbi2944S01716_SCLBI
CCaMK	Vigsu91S05737_VIGSU	——	Faial752S22546_FAIAL	——	———
MtIPD3/LjCYCLOPS	Vigsu104856S09608_VIGSU	Labpu701S17462_LABPU	——	——	Sclbi2578S10386_SCLBI
NIN	Vigsu273S23676_VIGSU	Labpu165S10337_LABPU	Faial788S23538_FAIAL	Morol36195S02810_MOROL	Sclbi2838S04948_SCLBI
MtCRE1/LjLHK1	——	Labpu2293S02028_LABPU	Faial1226S02883_FAIAL	——	——
NF-YA1	Vigsu107799S13964_VIGSU	Labpu193775S11413_LABPU	Faial246S12019_FAIAL	Morol36154S02289_MOROL	Sclbi406S12278_SCLBI
NF-YA2	——	——	Faial858S26716_FAIAL	——	———
MtERN1	Vigsu107612S00570_VIGSU	Labpu210S01798_LABPU	Faial719S21851_FAIAL	Morol36040S00658_MOROL	Sclbi1920S01196_SCLBI
MtERN2	Vigsu108137S07511_VIGSU	Labpu448S03276_LABPU	Faial4604S17896_FAIAL	——	———

Additional files

Figure S1: K-mer (K=17) analysis of five genomes.

Figure S2: Distribution of sequencing depth of the assembly data.

Figure S3: The GC content.

Figure S4: Comparison of GC content across closely related species.

Figure S5: Statistics of gene models in *V. subterranea, L. purpureus, F. albida, M. oleifera, S.birrea*.

Figure S6: Expansion and contraction of gene families.

Table S1. Statistics of the raw and clean data of DNA sequencing.

Table S2. Summary statistics of the transcriptome data in four species.

Table S3. Estimation of genome size based on K-mer statistics in five species.

Table S4. BUSCO evaluation of the annotated protein-coding genes in five species.

Table S5. Analysis of gene families of different species.

Table S6. Enriched pathways of unique paralogs genes in families.

Table S7. Enriched GO terms (level 3) of unique paralogs genes in families.

Table S8. Enriched GO terms (level 3) of genes in families with expansion.

Table S9. Enriched pathways of genes in families with expansion.

Table S10. The copy numbers of protein biosynthesis related genes in each species.

Table S11. The copy numbers of starch biosynthesis genes in each species.

Table S12. The copy numbers of fatty acid synthesis and storage related genes in each species.

Table S13. The copy number of fatty acid degradation related genes in each species.

Table S14. The numbers of Transcription factor in the studied species.

References

1. United Nations, Department of Economic and Social Affairs, Population Division. World population prospects: the 2017 revision, Key Findings and Advance Tables. 2017. Working Paper No. ESA/P/WP/248.

2. Development Initiatives. Global nutrition report 2017: nourishing the SDGs. Bristol, UK: Development Initiatives. 2017.

3. Mouillé, B., Charrondière, U. R., & Burlingame. The contribution of plant genetic resources to health and dietary diversity. Thematic Background Study. 2010.

4. Varshney RK, Chen W, Li Y, Bharti AK, Saxena RK, Schlueter JA, et al. Draft genome sequence of pigeonpea (*Cajanus cajan*), an orphan legume crop of resource-poor farmers. Nat Biotechnol. 2011;30:83-89. doi:10.1038/nbt.2022.

5. Foyer CH, Lam H-M, Nguyen HT, Siddique KHM, Varshney RK, Colmer TD, et al. Neglecting legumes has compromised human health and sustainable food production. Nat Plants. 2016;2:16112. doi:10.1038/nplants.2016.112.

6. Borget M. Food legumes. In: The Tropical Agriculturalist, CTA Macmillan. 1992.

7. Linnemann A.R, Azam–Ali S.N. Bambara groundnut (*Vigna subterranea*) literature review: A revised and updated bibliography. Tropical Crops Communication No. 7. 1993.

8. Gbaguidi AA, Dansi A, Dossou-Aminon I, Gbemavo DSJC, Orobiyi A, Sanoussi F, et al. Agromorphological diversity of local Bambara groundnut (*Vigna subterranea* (L.) Verdc.) collected in Benin. Genet Resour Crop Evol. 2018;65(4):1159-1171. doi:10.1007/s10722-017-0603-4.

9. Kang YJ, Kim SK, Kim MY, Lestari P, Kim KH, Ha B-K, et al. Genome

sequence of mungbean and insights into evolution within Vigna species. Nat Commun. 2014;5:5443. doi:10.1038/ncomms6443.

10. Yang K, Tian Z, Chen C, Luo L, Zhao B, Wang Z, et al. Genome sequencing of adzuki bean (*Vigna angularis*) provides insight into high starch and low fat accumulation and domestication. Proc Natl Acad Sci U S A. 2015;112(43):13213-13218. doi:10.1073/pnas.1420949112.

11. Jung IL. Soluble extract from *Moringa oleifera* leaves with a new anticancer activity. PLoS One. 2014;9(4):e95492. doi:10.1371/journal.pone.0095492.

12. Leone A, Spada A, Battezzati A, Schiraldi A, Aristil J and Bertoli S. Cultivation, genetic, ethnopharmacology, phytochemistry and pharmacology of *Moringa oleifera* Leaves: An Overview. Int J Mol Sci. 2015;16(6):12791-12835. doi:10.3390/ijms160612791.

13. Lea M. Bioremediation of turbid surface water using seed extract from *Moringa oleifera* Lam. (drumstick) tree. Curr Protoc Microbiol. 2014;33:1G.2.1-G.2.8. doi:10.1002/9780471729259.mc01g02s16.

14. Mabapa MP, Ayisi KK, Mariga IK, Mohlabi RC and Chuene RS. Production and utilization of moringa by farmers in Limpopo Province, South Africa. International Journal of Agricultural Research. 1962;12(4):160-171. doi:10.3923/ijar.2017.160.171.

15. Tian Y, Zeng Y, Zhang J, Yang CG, Yan L, Wang XJ, et al. High quality reference genome of drumstick tree (*Moringa oleifera* Lam.), a potential perennial crop. Science China Life Sciences. 2015;58(7):627-638. doi:10.1007/s11427-015-4872-x.

16. Maass BL, Knox MR, Venkatesha SC, Angessa TT, Ramme S and Pengelly BC. *Lablab purpureus*-a crop lost for Africa? Trop Plant Biol. 2010;3(3):123-135.

doi:10.1007/s12042-010-9046-1.

17. Robotham O and Chapman M. Population genetic analysis of hyacinth bean (*Lablab purpureus* (L.) Sweet, Leguminosae) indicates an East African origin and variation in drought tolerance. Genet Resour Crop Evol. 2017;64(1):139-148. doi:10.1007/s10722-015-0339-y.

18. Kamotho GN. Evaluation of adaptability potential and genetic diversity of Kenyan Dolichos bean germplasm. PhD thesis. 2015.

19. Vankatesha S.C. Molecular characterization and development of mapping populatuions for construction of genetic map in dolichos bean. PhD thesis. 2012.

20. Mokgolodi NC, Setshogo MP, Shi L-l, Liu Y-j and Ma C. Achieving food and nutritional security through agroforestry: a case of *Faidherbia albida* in sub-Saharan Africa. For. Stud. China. 2011;13(2):123-131. doi:10.1007/s11632-011-0202-y.

21. Garrity DP, Akinnifesi FK, Ajayi OC, Weldesemayat SG, Mowo JG, Kalinganire A, et al. Evergreen agriculture: a robust approach to sustainable food security in Africa. Food Sec. 2010;2(3):197-214. doi:10.1007/s12571-010-0070-7.

22. DUNHAM KM. Biomass dynamics of herbaceous vegetation in Zambezi riverine woodlands. African Journal of Ecology. 1990;28(3):200-212. doi:10.1111/j.1365-2028.1990.tb01153.x.

23. Barnes RD and Fagg CW. *Faidherbia albida* monograph and annotated bibliography. Oxford Forestry Inst. 2003;41-267

24. Nerd A, Mizrahi Y, Janick J and Simon JE. Domestication and introduction of marula (*Sclerocarya birrea* subsp. *caffra*) as a new crop for the Negev Desert of Israel. New crops. 1993;496-499.

25. Mng'Omba SA, Sileshi GW, Jamnadass R, Akinnifesi FK and Mhango J. Scion and stock diameter size effect on growth and fruit production of *Sclerocarya birrea* (Marula) trees. J Hortic For. 2012;4(9):153-60.

26. Gouwakinnou GN, Lykke AM, Assogbadjo AE and Sinsin B. Local knowledge, pattern and diversity of use of *Sclerocarya birrea*. J Ethnobiol Ethnomed. 2011;7 (1):1-9. doi:10.1186/1746-4269-7-8.

27. Yang T and Wu C. DNA Extraction for plant samples by CTAB. protocols.io. 2018; dx.doi.org/10.17504/protocols.io.pzqdp5w

28. Luo R, Liu B, Xie Y, Li Z, Huang W, Yuan J, et al. SOAPdenovo2: an empirically improved memory-efficient short-read de novo assembler. GigaScience. 2012;1(1):1-6. doi:10.1186/2047-217X-1-18.

29. Teh BT, Lim K, Yong CH, Ng CCY, Rao SR, Rajasegaran V, et al. The draft genome of tropical fruit durian (*Durio zibethinus*). Nat Genet. 2017;49:1633-1641. doi:10.1038/ng.3972.

30. Simao FA, Waterhouse RM, Ioannidis P, Kriventseva EV and Zdobnov EM. BUSCO: assessing genome assembly and annotation completeness with single-copy orthologs. Bioinformatics. 2015;31(19):3210-3212. doi:10.1093/bioinformatics/btv351.

31. Chang Z, Li G, Liu J, Zhang Y, Ashby C, Liu D, et al. Bridger: a new framework for de novo transcriptome assembly using RNA-seq data. Genome Biol. 2015;16:30. doi:10.1186/s13059-015-0596-2.

32. Kent WJ. BLAT--the BLAST-like alignment tool. Genome Res. 2002;12(4):656-664. doi:10.1101/gr.229202.

33. Li R, Yu C, Li Y, Lam TW, Yiu SM, Kristiansen K, et al. SOAP2: an improved ultrafast tool for short read alignment. Bioinformatics. 2009;25(15):1966-1967.

doi:10.1093/bioinformatics/btp336.

34. Tarailo-Graovac M and Chen N. Using RepeatMasker to identify repetitive elements in genomic sequences. Curr Protoc Bioinformatics. 2009;25(1) 4.10.1-4.10.14. doi:10.1002/0471250953.bi0410s25.

35. Han Y and Wessler SR. MITE-Hunter: a program for discovering miniature inverted-repeat transposable elements from genomic sequences. Nucleic Acids Res. 2010;38(22):e199-e199. doi:10.1093/nar/gkq862.

36. Ellinghaus D, Kurtz S and Willhoeft U. LTRharvest, an efficient and flexible software for de novo detection of LTR retrotransposons. BMC Bioinformatics. 2008;9:18. doi:10.1186/1471-2105-9-18.

37. Gremme G, Steinbiss S and Kurtz S. GenomeTools: a comprehensive software library for efficient processing of structured genome annotations. IEEE/ACM Trans Comput Biol Bioinform. 2013;10(3):645-656. doi:10.1109/tcbb.2013.68.

38. Steinbiss S, Willhoeft U, Gremme G and Kurtz S. Fine-grained annotation and classification of de novo predicted LTR retrotransposons. Nucleic Acids Res. 2009;37(21):7002-7013. doi:10.1093/nar/gkp759.

39. Edgar RC. MUSCLE: multiple sequence alignment with high accuracy and high throughput. Nucleic Acids Res. 2004;32(5):1792-1797. doi:10.1093/nar/gkh340.

40. Campbell MS, Holt C, Moore B and Yandell M. Genome annotation and curation using MAKER and MAKER-P. Curr Protoc Bioinformatics. 2014;48(1): 4.11.1-4.11.39. doi:10.1002/0471250953.bi0411s48.

41. Haas BJ, Papanicolaou A, Yassour M, Grabherr M, Blood PD, Bowden J, et al. De novo transcript sequence reconstruction from RNA-seq using the Trinity platform for reference generation and analysis. Nat Protoc. 2013;8:1494–1512. doi:10.1038/nprot.2013.084.

42. Haas BJ, Salzberg SL, Zhu W, Pertea M, Allen JE, Orvis J, et al. Automated eukaryotic gene structure annotation using EVidenceModeler and the program to assemble spliced alignments. Genome Biol. 2008;9(1):R7. doi:10.1186/gb-2008-9-1-r7.

43. Stanke M, Schoffmann O, Morgenstern B and Waack S. Gene prediction in eukaryotes with a generalized hidden Markov model that uses hints from external sources. BMC Bioinformatics. 2006;7:62. doi:10.1186/1471-2105-7-62.

44. Lomsadze A, Ter-Hovhannisyan V, Chernoff YO and Borodovsky M. Gene identification in novel eukaryotic genomes by self-training algorithm. Nucleic Acids Res. 2005;33(20):6494-6506. doi:10.1093/nar/gki937.

45. Korf I. Gene finding in novel genomes. BMC Bioinformatics. 2004;5:59. doi:10.1186/1471-2105-5-59.

46. Nawrocki EP, Burge SW, Bateman A, Daub J, Eberhardt RY, Eddy SR, et al. Rfam 12.0: updates to the RNA families database. Nucleic Acids Res. 2015;43(D1):D130-D137. doi:10.1093/nar/gku1063.

47. Lowe TM and Chan PP. tRNAscan-SE On-line: integrating search and context for analysis of transfer RNA genes. Nucleic Acids Res. 2016;44(W1):W54-W57. doi:10.1093/nar/gkw413.

48. Tanabe M and Kanehisa M. Using the KEGG database resource. Curr Protoc Bioinformatics. 2012; 38(1):1.12.1-1.12.43. doi:10.1002/0471250953.bi0112s38.

49. Tatusov RL, Koonin EV and Lipman DJ. A genomic perspective on protein families. Science. 1997;278(5338):631-637.

50. Boeckmann B, Bairoch A, Apweiler R, Blatter MC, Estreicher A, Gasteiger E,

et al. The SWISS-PROT protein knowledgebase and its supplement TrEMBL in 2003. Nucleic Acids Res. 2003;31(1):365-370.

51. Jones P, Binns D, Chang HY, Fraser M, Li W, McAnulla C, et al. InterProScan 5: genome-scale protein function classification. Bioinformatics. 2014;30(9):1236-1240. doi:10.1093/bioinformatics/btu031.

52. Finn RD, Mistry J, Tate J, Coggill P, Heger A, Pollington JE, et al. The Pfam protein families database. Nucleic Acids Res. 2010;38 suppl 1:D211-D222. doi:10.1093/nar/gkp985.

53. Letunic I, Doerks T and Bork P. SMART 6: recent updates and new developments. Nucleic Acids Res. 2009;37 suppl 1:D229-D232. doi:10.1093/nar/gkn808.

54. Mi H, Muruganujan A, Casagrande JT and Thomas PD. Large-scale gene function analysis with the PANTHER classification system. Nat Protoc. 2013;8:1551-1566. doi:10.1038/nprot.2013.092

55. Attwood TK, Bradley P, Flower DR, Gaulton A, Maudling N, Mitchell AL, et al. PRINTS and its automatic supplement, prePRINTS. Nucleic Acids Res. 2003;31(1):400-402.

56. Corpet F, Servant F, Gouzy J and Kahn D. ProDom and ProDom-CG: tools for protein domain analysis and whole genome comparisons. Nucleic Acids Res. 2000;28(1):267-269.

57. Stichting C, Centrum M and Dongen SV. A Cluster Algorithm for Graphs. Information Systems [INS]. 2000:1-40.

58. Guindon S, Dufayard JF, Lefort V, Anisimova M, Hordijk W and Gascuel O. New algorithms and methods to estimate maximum-likelihood phylogenies: assessing the performance of PhyML 3.0. Syst Biol. 2010;59(3):307-321.

doi:10.1093/sysbio/syq010.

59. Yang Z. PAML 4: Phylogenetic analysis by maximum likelihood. Mol Biol Evol. 2007;24(8):1586-1591. doi:10.1093/molbev/msm088.

60. He N, Zhang C, Qi X, Zhao S, Tao Y, Yang G, et al. Draft genome sequence of the mulberry tree *Morus notabilis*. Nat Commun. 2013;4:2445. doi:10.1038/ncomms3445.

61. Lavin M, Herendeen PS, Wojciechowski MF and Linder P. Evolutionary rates analysis of leguminosae implicates a rapid diversification of lineages during the Tertiary. Syst Biol. 2005;54(4):575-594. doi:10.1080/10635150590947131.

62. De Bie T, Cristianini N, Demuth JP and Hahn MW. CAFE: a computational tool for the study of gene family evolution. Bioinformatics. 2006;22(10):1269-1271. doi:10.1093/bioinformatics/btl097.

63. Bernhardt C, Lee MM, Gonzalez A, Zhang F, Lloyd A and Schiefelbein J. The bHLH genes GLABRA3 (GL3) and ENHANCER OF GLABRA3 (EGL3) specify epidermal cell fate in the Arabidopsis root. Development. 2003;130(26):6431-6439. doi:10.1242/dev.00880.

64. Paponov IA, Paponov M, Teale W, Menges M, Chakrabortee S, Murray JA, et al. Comprehensive transcriptome analysis of auxin responses in Arabidopsis. Mol Plant. 2008;1(2):321-337. doi:10.1093/mp/ssm021.

65. Vanneste S, Rybel BD, Beemster GTS, Ljung K, Smet ID, Isterdael GV, et al. Cell cycle progression in the pericycle is not sufficient for SOLITARY ROOT/IAA14-mediated lateral root initiation in *Arabidopsis thaliana*. Plant Cell. 2005;17(11):3035-3050. doi:10.1105/tpc.105.035493.

66. Vandenbeldt RJ. Faidherbia albida in the West African semi-arid tropics. ICRISAT. 1992. p. 107-110.

67. Jang YE, Kim MY, Shim S, Lee J and Lee S-H. Gene expression profiling for seed protein and oil synthesis during early seed development in soybean. Genes Genom. 2015;37(4):409-418. doi:10.1007/s13258-015-0269-2.

68. Bamshaiye OM, Adegbola JA and Bamishaiye EI. Bambara groundnut : an under-utilized nut in Africa. Adv Agric Biotechnol. 2011;1:60-72.

69. Raigond P, Ezekiel R and Raigond B. Resistant starch in food: a review. J Sci Food Agric. 2015;95(10):1968-1978.

70. Zhou H, Wang L, Liu G, Meng X, Jing Y, Shu X, et al. Critical roles of soluble starch synthase SSIIIa and granule-bound starch synthase Waxy in synthesizing resistant starch in rice. Proc Natl Acad Sci U S A. 2016;113(45):12844-12849. doi:10.1073/pnas.1615104113.

71. Bird AR, Flory C, Davies DA, Usher S and Topping DL. A novel barley cultivar (*Himalaya 292*) with a specific gene mutation in starch synthase IIa raises large bowel starch and short-chain fatty acids in rats. J Nutr. 2004;134(4):831-835. doi:10.1093/jn/134.4.831.

72. Morre DJ, Nyquist S and Rivera E. Lecithin biosynthetic enzymes of onion stem and the distribution of phosphorylcholine-cytidyl transferase among cell fractions. Plant Physiol. 1970;45(6):800-804.

73. Johnson KD and Kende H. Hormonal control of lecithin synthesis in barley aleurone cells: regulation of the CDP-choline pathway by gibberellin. Proc Natl Acad Sci U S A. 1971;68(11):2674-2677.

74. Soltis DE, Soltis PS, Morgan DR, Swensen SM, Mullin BC, Dowd JM, et al. Chloroplast gene sequence data suggest a single origin of the predisposition for symbiotic nitrogen fixation in angiosperms. Proc Natl Acad Sci U S A. 1995;92(7):2647-2651.

75. Doyle JJ. Phylogenetic perspectives on the origins of nodulation. Mol Plant Microbe Interact. 2011;24(11):1289-1295. doi:10.1094/MPMI-05-11-0114.

76. Geurts R, Xiao TT and Reinhold-Hurek B. What does it take to evolve a nitrogen-fixing endosymbiosis? Trends Plant Sci. 2016;21 (3):199–208. doi:10.1016/j.tplants.2016.01.012.

77. Wang Y, Tang H, Debarry JD, Tan X, Li J, Wang X, et al. MCScanX: A toolkit for detection and evolutionary analysis of gene synteny and collinearity. Nucleic Acids Res. 2012;40(7):e49. doi:10.1093/nar/gkr1293.

78. Horváth B, Li HY, Domonkos Á, Halász G, Gobbato E, Ayaydin F, et al. *Medicago truncatula* IPD3 is a member of the common symbiotic signaling pathway required for rhizobial and mycorrhizal symbioses. Mol Plant Microbe Interact. 2011;24(11):1345-1358. doi:10.1094/MPMI-01-11-0015.

79. Amor BB, Shaw SL, Oldroyd GED, Maillet F, Penmetsa RV, Cook D, et al. The NFP locus of *Medicago truncatula* controls an early step of Nod factor signal transduction upstream of a rapid calcium flux and root hair deformation. Plant J. 2003;34(4):495-506.

80. Ndoye I, Gueye M, Danso SKA and Dreyfus B. Nitrogen fixation in *Faidherbia albida*, *Acacia raddiana*, *Acacia senegal* and *Acacia seyal* estimated using the ^{15}N isotope dilution technique. Plant Soil. 1995;172(2):175-180. doi:10.1007/BF00011319.

81. Chang Y, Liu H, Liu M, Liao X, Sahu SK, Fu Y, Song B; Cheng S, Kariba R, Muthemba S, Hendre PS, Mayes S, Ho WK, Kendabie P, Wang S, Li L, Muchugi A, Jamnadass R, Lu H, Peng S, Deynze AV, Simons A, Yana-Shapiro H, Xu X, Yang H, Wang J, Liu X. Supporting data for "The draft genomes of five agriculturally important African orphan crops". GigaScience Database

2018. http://dx.doi.org/10.5524/100504

Figure 1. Phylogenetic and evolutionary analysis. The scale bar indicates 10 million years. The values at the branch points indicate the estimates of divergence time (mya), while the blue numbers show the divergence time (million years ago, Mya), and the red nodes indicate the previously published calibration times. *V.sub* showed the seeds of *Vigna subterranea*, *L.pur* showed the flowers of *Lablab purpureus*, *F.alb* showed the seed pods of *Faidherbia albida*, *S.bir* showed the fruit of *Sclerocarya birrea*, *M.ole* showed the flowers of *Moringa oleifera*.

Figure 2. (A) The groups of orthologues shared among the *Lablab purpureus* (LABPU), *Faidherbia albida* (FAIAL), *Glycine max* (GLYMA), *Medicago truncatula* (MEDTR), *Vigna subterranea* (VIGSU). (B) The groups of orthologues shared among the *Sclerocarya birrea* (SCLBI), *Moringa oleifera* (MOROL), *Carica papaya* (CARPA), *Citrus sinensis* (CITSI), *Theobroma cacao* (THECA). Venn diagram generated by http://bioinformatics.psb.ugent.be/webtools/Venn/.

Figure 3. The common symbiosis signaling pathway. A total of 16 root nodulation symbiosis signal (Sym) pathway genes were identified in three legumes (*V. subterranea*, *L. purpureus*, and *F. albida*) and two non-legumes (*S. birrea* and *M. oleifera*). Lj : *L. japonicas*; Mt: *Medicago truncatula*, and LCOs: Lipochitooligosaccharides.

Figure 4. The percentage of transcription factors in five orphan species. Blastp tools was utilized to search against 58 plant transcription factor families obtained from PlantTFDB (http://planttfdb.cbi.pku.edu.cn/) (Additional file 2: Table S14). In this figure, MADS include M-type_MADS and MIKC_MADS. MYB include MYB and MYB_related. NF-YA/B/C include NF-YA, NF-YB and NT-YC. “Others” comprises

31 types of transcription factors (E2F/DP, Nin-like, TALE, YABBY, GeBP, BES1, DBB, CO-like, CPP, SBP, STAT, WOX, BBR-BPC, CAMTA, AP2, ZF-HD, S1Fa-like, ARR-B, SRS, GRF, LSD, NF-X1, EIL, RAV, HRT-like, HB-PHD, VOZ, Whirly, SAP, LFY, NZZ/SPL) whose percentage was less than 1%.

Figure 5: The identification of the genes involved in the starch biosynthesis pathway. The identified genes involving in starch synthesis are shown in red. The number of homolog genes are presented in the additional file 2 Table S11. (AGP: ADP-glucose pyrophosphorylase; AGPL: AGP large subunit; AGPS: AGP small subunit; PHOH: Starch phosphorylase H (Cytosolic type); GBSS: granule-bound starch synthase; SS: soluble starch synthase; BE: starch branching enzyme; ISA: isoamylase DPE: starch debranching enzyme).

Manuscript with track changes
Click here to view linked References
Click here to access/download;Manuscript;GIGA-D-18-00275.docx

The draft genomes of five agriculturally important African orphan crops

Yue Chang[1,2*], Huan Liu[1,2*], Min Liu[1,2*], Xuezhu Liao[1,2], Sunil Kumar Sahu[1,2], Yuan Fu[1,2], Bo Song[1,2], Shifeng Cheng[1,2], Robert Kariba[3], Samuel Muthemba[3], Prasad S. Hendre[3], Sean Mayes[5,6,7], Wai Kuan Ho[6,7], Presidor Kendabie[5], Sibo Wang[1,2], Linzhou Li[1,2], Alice Muchugi[3], Ramni Jamnadass[3], Haorong Lu[1,2], Shufeng Peng[1,2], Allen Van Deynze[3,4], Anthony Simons[3], Howard Yana-Shapiro[3,4], Xun Xu[1,2], Huanming Yang[1,2], Jian Wang[1,2], Xin Liu[1,2,8#].

1. BGI-Shenzhen, Shenzhen 518083, China
2. China National GeneBank, BGI-Shenzhen, Shenzhen 518120, China
3. African Orphan Crops Consortium, World Agroforestry Centre (ICRAF), Nairobi, Kenya
4. University of California, 1 Shields Ave, Davis, USA, 95616
5. Plant and Crop Sciences, Biosciences, University of Nottingham, Sutton Bonington Campus, Loughborough, Leicestershire, LE12 5RD
6. Biosciences, University of Nottingham Malaysia Campus, Jalan Broga 43500 Semenyih, Selangor, Malaysia
7. Crops For the Future, Jalan Broga, 43500 Semenyih, Selangor, Malaysia
8. BGI-Fuyang, BGI-Shenzhen, Fuyang 236009, China

Correspondence address: Xin Liu (liuxin@genomics.cn)

* Equal contribution

ABSTRACT

Background: A continued Continuous growth in the world population is expected to double the worldwide demand for food by 2050. Moreover, 88% of countries are currently facing a serious burden of malnutrition, especially in Africa and Southern & South-Eastern Asia. 30 species alone contribute Presently, aAbout 95% of the present food energy needs of humans are contributedfulfilled by 30 species, within which wheat, maize and rice providing provide the majority of calories. Therefore, to diversify and stabilize global food supply, enhance agricultural productivity and tackle malnutrition in these countries, a greater utilization of neglected or underutilized underused local plants (generally so-called orphan crops, but also a few plants with special contribution to agriculture, such agroforestry and nutrientcrops (orphan crops) could be a partial solution.

Findings: Here we present draft genome information from five five agriculturally, biologically, medicinally and economically important underutilized plants in African orphan crops, namely; *Vigna subterranea*, *Lablab purpureus*, *Faidherbia albida*, *Sclerocarya birrea*, and *Moringa oleifera*. The assembled genomes range in size from 217 to 654 Mb. In addition, we have predicted 31707, 20946, 28979, 18937, 18451 protein-coding genes in *V. subterranea*, *L. purpureus*, *F. albida*, *S. birrea* and *M. oleifera* respectively. We have further analyzed the expansion and contraction of selected gene families, and characterized root-nodule-symbiosis genes, transcription factors and starch biosynthesis related genes in these genomes.

Conclusions: This genome data will be useful to identify and characterize agronomically important genes and understand their mode of actions, enabling genomics-based, evolutionary studies, and breeding strategies for designing faster, focused and predictable crop improvement programs.

Keywords: Orphan crops; food security; whole-genome sequencing; transcriptome; root nodule symbiosis; transcription factors

BACKGROUND INFORMATION

The world's population is expected to reach 9.8 billion by 2050, thus ~~and~~ ensuring a sustainable food supply to meet the energy and nutritional needs of the expanding population is one of the greatest global challenge ~~ahead of us~~ [1]. Moreover, about 88% of the countries are currently facing a serious burden of malnutrition [2]. To overcome this burden food and nutritional challenge, the utilization of potential crops (both model and non-model) ~~–~~plants ~~is almost the only choice~~appears to be a better choice~~appear to be the best choice~~. Throughout history, human beings have relied on astonishing varieties of plants for energy and nutrition: From 390,000 known plant species, it is estimated that around 5,000-7,000 plant species have been cultivated or collected for food [1, 2]. But, in the present century, less than 150 species are commercially cultivated for food purposes, and surprisingly 30 species alone provide 95% of the food energy needs of humans. More than half of the protein and calories which we obtain from plants are acquired from just three 'megacrops' – rice, wheat and maize [3]. This narrow range of dietary diversity is partly a result of decades of intensive research, focused on just a few species, which has successfully led to the production of high-yielding varieties of these major crops, usually cultivated under high input agricultural systems. However, we are now witnessing a drastic decrease in their yields in some regions and it has been questioned whether rice and wheat (in particular) are currently making enough breeding progress to meet the challenge. All three

Formatted: Not Highlight

Formatted: Not Highlight

Formatted: Not Highlight

Formatted: Not Highlight

Formatted: Not Highlight

megacrops are high energy carbohydrate sources, but are limited in protein content. Even if these crops can meet the energy requirement of the increasing world population, they cannot meet the nutritional requirement for active health by themselves [2].

To diversify the global food supply, enhance the agricultural productivity and tackle malnutrition, it is necessary to diversify and focus more on crop plants that are utilized in rural societies as a local source of nutrition and sustenance, but have received little attention for crop improvement. These landraces tend to be locally adapted and can often provide a rich source of nutrition yet they largely been kept out of modern interventions. The goal of the African Orphan Crops Consortium (AOCC), an international public-private partnership is to sequence, assemble and annotate the genomes of 101 plants contributed to traditional African food ~~crops~~ supplies by 2020 (www.africanorphancrops.org). These neglected or orphan ~~crops~~ plants have been little studied by science, but are of major importance in many African countries. They are usually grown by smallholder farmers, either for consumption or local sale, and are a major food source for 600 million rural Africans [4, 5]. In this study, we sequenced and assembled draft genomes of five African orphan plant species (Figure 1)~~-~~, which are highly important to augment food and nutritional security in Africa.

Vigna subterranea (Bambara groundnut; NCBI taxon ID 115715) belonging to Fabacaeae family is a leguminoceous plant species which originated in West Africa, and is cultivated in Sub-Saharan areas, particularly Nigeria [6,7]. With good nitrogen-fixing ability, drought tolerance, on average the seeds contain 63% carbohydrate, 19% protein and 6.5% oil, thereby ~~highly~~ making bambara groundnut a complete food. ~~-~~The

Formatted: Not Highlight

Formatted: Not Highlight

annual production of this species is about 165,000 tons in Africa, and yields are low because efforts to improve bambara has been negligible for many years [8]. The genomes of mung bean and adzuki bean have been published [9,10], which also belongs to *Vigna* genus~~which are in the same genus of *Vigna subterranea*~~.

Moringa oleifera (Moringa; NCBI taxon ID 3735) is a highly nutritious, fast growing and drought tolerant tree, and is indigenous to Northern India, Pakistan and Nepal [11~~9~~]. Presently, this species is ubiquitously distributed throughout tropical and subtropical countries, and in particular covers the major agro-ecological region in Nigeria. The leaves are rich in protein, minerals, beta-carotene and antioxidant compounds which are generally used as nutrition supplements and in traditional medicine. The seeds are used to extract oil and seed powder can be used for water purification [12~~0~~, 13~~11~~]. Various sources have had varying reports of Moringa production, India is the largest producer of Moringa with an annual production of 1.1–1.3 million tonnes of tender fruits from an area of 38,000 ha. In Limpopo province relatively small holder areas (0.25- 1ha) are under Moringa cultivation with seed yields of 50-100 kgs/ha^{-1} [14~~2~~]. ~~Before~~Prior to this study, a draft genome of *Moringa oleifera* from Yunnan (China) was ~~published~~also reported [15]~~in 2015~~ with similar~~ity~~ ~~in~~genome assembly size and gene numbers compared to our version.

Lablab purpureus (Dolichos bean or hyacinth bean; NCBI taxon ID 35936), a member of Fabaceae family is one of the most ancient (>3500 years) domesticated and multipurpose legume species used as an intercrop in livestock systems. Although it displays a large agro-morphological diversity in South Asia, its origin appears to be

Commented [A1]: Cite:
1. Genome sequence of mungbean and insights into evolution within *Vigna*s pecies
2. [57] Yang K, Tian Z, Chen C, Luo L, Zhao B, Wang Z, et al. Genome sequencing of adzuki bean (*Vigna angularis*) provides insight into high starch and low fat accumulation and domestication. Proc Natl Acad Sci U S A. 2015;112(43):13213-13218. doi: 10.1073/pnas.1420949112.

Formatted: Font: Italic

Formatted: Font: Italic

African [16~~13~~]. It is rich in protein, has good nitrogen-fixing ability and displays high adaptability to a diverse range of environmental conditions [17~~14~~]. There is limited production data available suggesting that yields are low. In ~~S~~south~~ W~~western parts of Bangladesh, lablab is reported to have a total production area of approximately 48000 ha [16~~13~~]. In other areas Dolichos is reported to have a similarly relatively low production area for example Kenya, ~~appx~~approx. 10,000 ha [18~~15~~] and Karnataka India, 79000 ha [19~~16~~].

Faidherbia albida (apple-ring acacia; NCBI taxon ID 138055) is the only tree species in genus *Faidherbia* (Fabaceae). Due to its distinctive key features like reverse phenology (leaves grow in the long dry season and shed during the rainy season) and nitrogen-fixing ability, *F. albida* has been planted as a key agroforestry species in traditional African farming systems for hundreds of years [20~~17~~]. It originated in the Sahara or Eastern and Southern Africa, then spread over semi-arid tropical Africa, later spreading to the Middle East and Arabia. It is estimated that tree was cultivated over an area of 300,000 hectares during the last decade [21~~18~~] The average pod production ranges from 6-135 kgs per tree in a year in the Sudanian zone. In Zimbabwe (Manapools) two trees averaged 161 kgs per tree in a year [22~~19~~]. This yield per unit area is about 2000 to 3000kg/ha on assumption of about 20 mature trees per hectare [23~~20~~].

Sclerocarya birrea (Marula; NCBI taxon ID 289766) belongs to the Anacardiaceae family, and is a traditional fruit tree found in southern Africa, mostly south of the Zambesi river [24~~21~~]. The fruits are eaten fresh or used to produce juices and wine which has substantial socioeconomic and commercialization importance. The seed of

Formatted: Not Highlight

Formatted: Not Highlight

Formatted: Not Highlight

Formatted: Not Highlight

Formatted: Not Highlight

the fruits are rich in nutrition and oil content (56%) and are often consumed raw. It is estimated that the total value of the commercial marula trade to the rural communities is worth USD $160,000 a year [25~~22~~] with values per tree ranging from 315 kg (17,500 fruits) to 1643 kg (91,300 fruits) [25~~22~~, 26~~23~~]. A survey in Northcentral Namibia showed that on an average there are 5.33 farm/household with a total number of 13,278 fruiting trees.

~~Taking into account~~Considering the limited systematic efforts to improve the breeding of these crops, the availability of genomic data of these understudied tropical plants will give much needed impetus to conduct basic as well as applied translational research to improve and develop them as important food crops adapted for sustainable cultivation. These efforts are a vital instrument for direct or indirect nutrition of an increasing urban population in the regions these crops are grown.

DATA DESCRIPTION

Sample collection, library construction, and sequencing

The genomic DNA was extracted either from a tree (*Faidheriba albida*, *Moringa oleifera*) or from nursery plantlets (*Vigna subtarranea*, *Lablab purpureus*, *Sclerocarya birrea*) grown at the World AgroForestry Center (ICRAF) campus in Kenya using a modified CTAB method [27~~24~~].

The extracted DNA was used to construct paired-end libraries (insert size from 170 to 800 bp) and mate-pair libraries (insert size larger than 2 kb) following the protocols from Illumina (San Diego, USA). Subsequently, the sequencing was performed on a

HiSeq 2000 platform (Illumina, San Diego, CA, USA) with a strategy of shotgun sequencing to generate more than 100 Gb raw data for each species (Additional file1: Table S1). The data were filtered using SOAPfilter (v2.2) [2825] as follows: (1) small insert size reads were discarded; (2) PCR duplicates and adapter contamination were discarded; (3) reads with ≥30% low quality bases (quality score ≤ 15) were removed; (4) bases with low quality were trimmed from both sides of the reads; (5) reads with ≥ 10% uncalled ("N") bases were removed. Finally, more than 100× of high-quality reads were obtained for each species according to their estimated genome size (Additional file1: Table S1).

RNA for transcriptome sequencing was extracted from different tissues of *Vigna subterranea, Lablab purpureus, Faidherbia albida, Moringa oleifera*. The RNA was extracted using the PureLink RNA Mini Kit (Thermo Fisher Scientific, Carlsbad, CA, USA) according to the manufacturer's instructions. Libraries for the RNA samples were constructed following the manual of TruSeq RNA Sample Preparation Kit (Illumina, San Diego, CA, USA), and then sequenced on the Illumina HiSeq 2500 platform (paired-end, 100 base pair reads) and generated about 36 Gb of sequence data for each species. The data was then filtered with a strategy similar to DNA filtration, except a slight modification: (1) reads with ≥10% low quality bases (quality score ≤ 15) were removed; (2) reads with ≥ 5% uncalled ("N") bases were removed (Additional file 1: Table S2). We compiled all the transcriptome data from different tissues, and used the combined version to check the completeness of the WGS assembly.

Evaluation of genome size

Clean reads of the paired-end libraries were used to estimate genome sizes. (insert size 250 bp and 500 bp). The k-mer frequency distribution analysis was performed using the following formula: $Gen = Num*(Len - 17 + 1) / K\_Dep$, where *Num* represents the read number of used reads, *Len* represents the length of read, *K* represents the length of k-mer and *K_Dep* refers to where the main peak is located in the distribution curve [29~~26~~]. In this analysis, K-mer distributions of *F. albida*, *S. birrea*, and *M. oleifera* showed two distinct peaks (Additional file1: Figure S1), where the second peak was confirmed as the main one for each of the species. The genome size of *V. subterranea*, *L. purpureus*, *F. albida*, *S. birrea* and *M. oleifera* was predicted as 550, 423, 661, 356 and 278 Mb, respectively (Additional file1: Table S3).

***De novo* assembling of genomes**

For *de novo* genome assembly, SOAPdenovo2 (SOAPdenovo2, RRID:SCR_014986) [28~~25~~] was used for constructing contigs, followed by scaffolding, and finally gap filling. To build a contig, libraries ranging from 170 to 800 bp were used to construct de Bruijn graphs with the parameters "pregraph -d 2 -K 55, and contigs were subsequently formed with the parameters "contig -g -D 1" to delete links with low coverage. In the scaffolding step, paired-end and mate-pair information was used to order the contigs with parameters "scaff -g -F" and "map -g -k 55". Finally, to fill the gaps within scaffolds, GapCloser version 1.12 (GapCloser, RRID:SCR_015026) [28~~25~~] was used with the parameters "-l 150 -t 32" using the pair-end libraries. Finally, a total

assembled length of 535.05, 395.47, 653.73, 330.98, and 216.76 Mb was obtained for *V. subterranea*, *L. purpureus*, *F. albida*, *S.birrea* and *M. oleifera* genomes, respectively (Table 1). This accounted for approximately 97.3%, 93.5%, 98.9%, 92.9% and 77.9% of their estimated genome size, respectively.

Genome evaluation

The completeness of the genome assemblies was assessed with BUSCO version 3.0.1 (Benchmarking Universal Single-Copy Orthologues), (BUSCO, RRID:SCR_015008) [3027]. From the 1,440 core embryophyta genes, 1,326 (92.1%), 1,341 (93.2%), 1,315 (91.3%), 1,384 (96.1%) and 1,297 (90.1%) were identified in the *V. subterranea*, *L. purpureus*, *F. albida*, *S. birrea* and *M. oleifera* assemblies, with 1,244 (86.4%), 1,258 (87.4%), 1,231 (85.5%), 1,352 (93.9%) and 1.278 (88.8%) genes being complete (Table 2), respectively.

To evaluate the completeness of genes in the assemblies, unigenes were generated from the transcript data of each species using Bridger software with the parameters "-kmer_length 25 -min_kmer_coverage 2" [3128], and then aligned to the corresponding assembly using BLAT (BLAT, RRID:SCR_011919) [3229]. The results indicated that each of the assemblies covered about 90% of the expressed unigenes, suggesting that the assembled genomes contained a high percentage of expressed genes (Table 3).

In order to confirm the accuracy of the assemblies, some of the paired-end libraries were mapped to the genome assemblies and the sequencing coverage was calculated using SOAPaligner, version 2.21 (SOAPaligner/soap2 , RRID:SCR_005503) [3330].

The sequencing coverage showed that > 99% of the bases had a sequencing depth of more than 10 x and confirmed the accuracy at the base level (Additional file1: Figure S2). The GC content and average depth were also calculated with 10 kb non-overlapping windows, the distribution of GC content indicated a relatively pure single genome without contamination or GC bias (Additional file1: Figure S3). Moreover, the GC content of each sequenced genome was also compared to that of their related species. As expected, the close peak positions showed the related species were similar in GC content (Additional file1: Figure S4).

Repeat annotation

Repetitive sequences were identified using RepeatMasker (version 4-0-5) [34~~31~~], with a combined Repbase and a custom library obtained through careful self-training. The custom library composed of three parts: the MITE (miniature inverted repeat transposable elements), LTR (long terminal repeat) and an extensive library which was constructed as follows. First, the annotated MITE library was created using MITE-hunter [35~~32~~] with default parameters. Then, the LTR elements with a length of 1.5 kb to 25 kb, and two terminal repeats ranging from 100 bp to 6000 bp with >= 85% similarity was constructed using LTRharvest [36~~33~~] integrated in Genometools (version 1.5.8) [37~~34~~] with parameters "-minlenltr 100 -maxlenltr 6000 -mindistltr 1500 -maxdistltr 25000 -mintsd 5 -maxtsd 5 -similar 90 -vic 10". Subsequently, we used several strategies to filter the candidates, e.g. *i.* presence of intact PPT (poly purine tract) or PBS (primer binding site) sites [38~~35~~] using the eukaryotic tRNA library

(http://gtrnadb.ucsc.edu/), *ii.* removal of contamination from local gene clusters and tandem local repeats by inspecting 50 bases of the upstream and downstream LTR flanks using MUSCLE (MUSCLE, RRID:SCR_011812) [39~~36~~] for a minimum of 60% identity *iii.* removal of nested LTR candidates with other types of the elements. Exemplars for the LTR library were extracted from the filtered candidates using a cutoff of 80% identity in 90% of the sequence. Furthermore, the regions annotated as LTRs and MITEs in the genome were masked, and then put into RepeatModeler version 1-0-8 (RepeatModeler, RRID:SCR_015027) to predict other repetitive sequences for the extensive library. Finally, the MITE, LTR and extensive libraries were integrated into the custom library, which was combined with the Repbase library and taken as an input for RepeatMasker to identify and classify genome-wide repetitive elements. The pipeline identified 205,189,285 (38.35% of the genome length), 147,050,327 (37.18%), 358,653,534 (54.86%), 149,551,125 (45.18%), and 87,944,150 (40.57%) bases of non-redundant repetitive sequences in *V. subterranea*, *L. purpureus*, *F. albida*, *S. birrea* and *M. oleifera* respectively. LTR elements were predominant, taking up to 19.8%, 23.8%, 44.6%, 38.8%, 22.7% of each genome, respectively (Table 4).

Gene prediction

Repetitive regions of the genome were masked before gene prediction. The structures of protein-coding genes were predicted using the MAKER-P pipeline (version 2.31) [40~~37~~] based on RNA, homologous and *de novo* prediction evidence. For RNA evidence, the clean transcriptome reads were assembled into inchworms using Trinity

version 2.0.6 [41~~38~~], and then provided to MAKER-P as EST evidence. For homologous comparison, the protein sequences from the model plant *Arabidopsis thaliana* and related species of each sequenced species were downloaded and provided as protein evidence. The related species we used for homologous evidence are listed below: *V. subterranea*: (*Arachis duranensis*, *Arachis ipaensis*, *Glycine max*, *Lotus japonicus*, *Medicago truncatula*, *Vigna angularis*); *L. purpureus*: (*A. duranensis*, *Cajanus cajan*, *G. max*, *M. truncatula*, *Phaseolus vulgaris*, *Vigna angularis*); *F. albida*: (*Cajanus cajan*, *V. angularis*, *L. japonicus*, *P. vulgaris*, *M. truncatula*, *G. max*); *S. birrea*: (*Actinidia chinensis*, *Musa acuminata*); *M. oleifera*: (*G. max*, *Oryza sativa*, *Populus trichocarpa*, *Sorghum bicolor*).

For evidence from *de novo* prediction, a series of training sets were made to optimize different *ab initio* gene predictors. Initially, a set of transcripts were generated by a genome-guided approach using Trinity with parameters "--full_cleanup --jaccard_clip --genome_guided_max_intron 10000 --min_contig_length 200". The transcripts were then mapped back to the genome using PASA (version 2.0.2) [42~~39~~] and a set of gene models with real gene characteristics (e.g. size and number of exons/introns per gene, features of splicing sites) were generated. The complete gene models were picked for training Augustus [43~~40~~]. Genemark-ES (version 4.21) [44~~41~~] was self-trained with default parameters. The first round of MAKER-P was run based on the evidence as above with default parameters except with "est2genome" and "protein2genome" were set to "1", yielding only RNA and protein-supported gene models. SNAP [45~~42~~] was then trained with these gene models. Default parameters were used to run the second

and final round of MAKER-P, producing the final gene models.

Finally, 31,707, 20,946, 28,979, 18,937 and 18,451 protein-coding genes were identified in *V. subterranea*, *L. purpureus*, *F. albida*, *S. birrea* and *M. oleifera*. Compared to the other sequenced species in the same genus [9, 10], the gene number of *V. subterranea* is more than that of mung bean (22,427) but less than that of adzuki bean (34,183). Various gene structure parameters were compared to the related species of each sequenced genome as summarized in table 5 and additional file1: Figure S5. BUSCO evaluation showed that at least 85% of 1,440 core genes could be identified across all the species, suggesting an acceptable quality of gene annotation for the five sequenced genomes (Additional file1: Table S4).

Furthermore, non-coding RNA genes in the sequenced genomes were also annotated. The ribosomal RNA (rRNA) genes were searched using BLAST against the *A. thaliana* rRNA database, or by searching for microRNAs (miRNA) and small nuclear RNA (snRNA) against the Rfam database (Rfam, RRID:SCR_004276) (release 12.0) [4643]. Further, tRNAscan-SE (tRNAscan-SE, RRID:SCR_010835) was used to scan for transfer RNAs (tRNA) [4744]. The result is summarized in Table 6.

Functional annotation of protein-coding genes

The functional annotation of protein-coding genes was based on sequence similarity and domains conservation by aligning predicted amino acid sequences to public databases. The protein-coding genes were first searched against protein sequence databases for best matches, such as KEGG (KEGG, RRID:SCR_012773) [4845], NR

Commented [A2]: Cite:

1. Genome sequence of mungbean and insights into evolution within *Vigna*s pecies

2. [57] Yang K, Tian Z, Chen C, Luo L, Zhao B, Wang Z, et al. Genome sequencing of adzuki bean (*Vigna angularis*) provides insight into high starch and low fat accumulation and domestication. Proc Natl Acad Sci U S A. 2015;112(43):13213-13218. doi: 10.1073/pnas.1420949112.

Formatted: Font: Italic

database (NCBI), COG [4946], SwissProt and TrEMBL [5047] using BLASTP with an E-value cut-off of 1e-5. Then, InterProScan 55.0 (InterProScan, RRID:SCR_005829) [5148] was used as an engine to identify domains and motifs based on Pfam (Pfam, RRID:SCR_004726) [5249], SMART (SMART, RRID:SCR_005026) [5350], PANTHER (PANTHER, RRID:SCR_004869) [5451] , PRINTS (PRINTS, RRID:SCR_003412) [5552] and ProDom (ProDom, RRID:SCR_006969) [5653]. In total, 98.0%, 98.2%, 93.6%, 98.1% and 98.8% of genes in *V. subterranea*, *L. purpureus*, *F. albida*, *S.birrea* and *M. oleifera* were functionally annotated. Among the unannotated genes, there are 400, 305, 1514, 293 and 172 genes specific in *V. subterranea*, *L. purpureus*, *F. albida*, *S. birrea* and *M. oleifera* respectively (Table 7).

Formatted: Not Highlight

Gene family construction

Protein and nucleotide sequences from the five sequenced species and 9 other species (*A. thaliana*, *Carica papaya*, *Citrus sinensis*, *G. max*, *M. truncatula*, *O. sativa*, *P. vulgaris*, *S. bicolor*, *Theobroma cacao*) were retrieved to construct gene families using OrthoMCL software [5754] based on an all-versus-all BLASTP alignments with an E-value cutoff of 1e-5. A total of 609, 104, 499, 205 and 150 gene families were found specific to *V. subterranea*, *L. purpureus*, *F. albida*, *S. birrea* and *M. oleifera*, respectively (Additional file1: Table S5).

Furthermore, the 10,103 gene families of *V. subterranea*, *L. purpureus*, *F. albida*, *M. truncatula* and *G. max* were clustered (Figure 2A). There were 1,105 orthologous families shared by the four Papilionoideae species, while 808 gene families containing

1,966 genes were specific to *F. albida*, 281 gene families containing 538 genes were specific to *L. purpureus*, 789 gene families containing 3,118 genes were specific to *V. subterranea*.

Moreover, 8,184 gene families of *S. birrea*, *M. oleifera*, *C. papaya*, *C. sinensis* and *T. cacao* were clustered (Figure 2B), of which 365 gene families containing 798 genes were specific to *M. oleifera*, 362 gene families containing 796 genes were specific to *S. birrea*, respectively. The enrichment analysis on KEGG pathway of the paralogs genes were also calculated (Additional file1: Table S6, S7). The functional annotation revealed that they mainly correspond to the carbon fixation, zeatin biosynthesis, glyoxylate and dicarboxylate metabolism in *V. subterranea*. However, for *L. purpureus*, the fatty acid elongation pathway was enriched. While in *F. albida*, the pathways corresponding to the plant-pathogen interaction and cyanoamino acid metabolism were enriched. In *S. birrea*, the pathways of plant-pathogen interaction, starch and sucrose metabolism, fatty acid biosynthesis were enriched. In *M. oleifera*, the pathways related to fatty acid and diterpenoid biosynthesis, cyanoamino acid metabolism were enriched. The enrichment analysis on GO of paralogs genes were ion binding, metabolic process, disease resistance, cell component, biological process in *V. subterranea*, *L. purpureus*, *F. albida*, *M. oleifera*, and *S. birrea* respectively.

Phylogenetic analysis and divergence time estimation

We identified 141 single-copy genes in the 14 species used for the above analysis, and subsequently used them to build a phylogenetic tree. Coding DNA sequence (CDS)

Formatted: Indent: Left: 0", Hanging: 0.29"

alignments of each single-copy family were generated following the protein sequence alignment with MUSCLE (MUSCLE, RRID:SCR_011812) [3936]. The aligned CDS sequences of each species were then concatenated to a supergene sequence. The phylogenetic tree was constructed with PhyML-3.0 (PhyML, RRID:SCR_014629) [5855] with the HKY85+gamma substitution model on extracted four-fold degenerate sites. Divergence time was calculated using the Bayesian relaxed molecular clock method with MCMCTREE in PAML (PAML, RRID:SCR_014932) [5956], based on the published calibration times (39-59 Mya between divergence time between *M. truncatula* and the main branch of legumes is 39-59 Mya, 15-30 Mya between *G. max* and *P. vulgaris*, , and 83-90 Mya between *T. cacao* and *A. thaliana*) [1057, 6058]. Based on the tree constructed by single-copy-family genesIn the present study, the divergence time between *F. albida* and Papilionoideae was predicted to be 79.1 (70.0-87.0) Mya, which is a little different from the previous predicted origin of legumes based on two gene markers (matk and rbcL) [61]. Wwhereas, the divergence time between *M. oleifera* and *C. papaya* was predicted to be 65.4 (59.2-71.1) Mya, and 67.9 (53.6-77.3) Mya between *S. birrea* and *C. sinensis* (Figure 13). Subsequently, to evaluate the gene gain and loss, CAFECAFE (-CAFE, RRID:SCR_005983) [6259] was employed to estimate the universal gene birth and death rate λ (lambda) under a random birth and death model with the maximum likelihood method. The results for each branch of the phylogenetic tree were estimated and represented in Figure 14. Enrichment analysis on GO and pathway of genes in expanded families in the lineage of each

Formatted: Font: Not Italic

Commented [A3]: Cite: Lavin et al. (2005), DOI: 10.1080/10635150590947131.

sequenced species were also calculated (Additional file1: Table S86, S97). Terms related to energy and nutrient metabolism were commonly distributed in the enrichment output of *V. subterranean, L. purpureus, M. oleifera and S. birrea*, such as proton-transporting two-sector ATPase complex, cyclase activity, nutrient reservoir activity and carbohydrate derivative binding. While in *F. albida*, expansion of gene families were related to signal transfer or regulation, such as signaling receptor activity, phosphatase regulator activity regulation of response to stimulus and so on. Furthermore, regulatory factors (*GLABRA3*, *ENHANCER OF GLABRA 3*, *AUX1*, *LAX2*, and *LAX3*) [630-6562] related to the formation of root hair and lateral root were identified in these families. As a traditional agroforestry tree in Africa, *F. albida* was previously reported to have a root system architecture (RSA) displaying severe variations to different environmental factors (soil depth, nutrient amount, or water reservoirs) [6663], suggesting its adaptability to the complex environment, which requires signal transferring and regulation. The result of the GO enrichment analysis was consistent with the biological characteristic of *F. albida*.

Mining of transcription factors

The transcription factors (TFs) in the sequenced species, were identified using protein sequences of plant TFs from the plant transcription factor database (http://planttfdb.cbi.pku.edu.cn/index.php) by BLASTP search with an e-value cutoff of 10E−10, a minimum identity of 40% and a minimum query coverage of 50%. About

59 TF families were (Additional file 2: Table S14~~2~~) were revealed across the genes in *M. truncatula*, *G. max*, *P. vulgaris*, *C. papaya*, *C. sinensis*, and the five sequenced species. Among these TFs, bHLH, NAC, ERF, MYB related, C2H2, MYB, WRKY, bZIP, FAR1, C3H, B3, G2-like, Trihelix, LBD, GRAS, M-type MADS, HD-ZIP, MIKC_MADS, HSF, GATA were found in major abundance (Figure 4~~6~~).

Formatted: Line spacing: Double

Identification of protein, starch, and fatty acid biosynthesis related genes

~~Identification of protein, starch, and fatty acid biosynthesis related genes~~

Formatted: Heading 2, Line spacing: single, Tab stops: Not at 0.77"

Using the amino acid, starch and fatty acid synthesis genes in soybean [~~57~~10, 67~~64~~] as bait, we performed an ortholog search in *V. subterranea*, *L. purpureus*, *F. albida*, *S. birrea*, *M. oleifera*, *G. max*, *T. aestivum*, *Z. mays* and *O. sativa* (Additional file 1: Table S10~~8~~,Table S11~~9~~, Table S12~~0~~, Table S13~~1~~). *V. subterranea* is a good source of resistance starch (RS) [68~~65~~], which has the potential to protect against diabetes and reduce the incidence of diarrhea and other inflammatory bowel disease [69~~66~~]. It is known that high amylose can contribute to RS, and previously studies have shown that deficiency in *SSIIIa* (soluble starch synthase gene) will decrease amylopectin biosynthesis and increase the amylose biosynthesis by GBSSI encoded by the *Wx* gene in *indica* [70~~67~~]. ~~In other cereals, d~~Down-regulation of soluble starch synthase (SS) *SSIIa* and of *SBE* will lead to~~results in~~ ~~greater~~ higher RS amount in barley [71~~68~~]. Interestingly, two out of four granule-bound starch synthase GBSS in *V. subterranea*

Formatted: Not Highlight

underwent expansion, suggesting its vital role in controlling starch synthesis (Figure 5) at the transcriptional and post-transcriptional level. Moreover, no expansion in GBSS was observed among *L. purpureus*, *F. albida*, *S. birrea* and *M. oleifera* genomes. Meanwhile the soluble starch synthase SS in *V. subterranea* were not expanded. Therefore, we speculate that the expansion of GBSS might be the reason why *V. subterranea* is rich in resistance starch.

Similarly, difference in the copy numbers of ~~the copy numbers of~~ choline kinase, which is a key factor ~~encodes~~ in fatty acid synthesis and storage ~~genes in *V. subterranea*~~ (7) was found to be different from the other three legumes including *G. max* [*F. albida* (4), *L. purpureus* (2), *G. max* (5) and two orphan species (*S. birrea* (1), *M. oleifera* (3)]. The choline kinase is the first enzyme in the cytidine diphosphate-choline pathway which is involved in lecithin biosynthesis [72~~69~~, 73~~70~~]. Based on these observations we inferred that the all the necessary factor to synthesize lecithin are present in V. *subterranea.* ~~Based on these observations we inferred that the ability to synthesize lecithin in *V. subterranea* is higher than that of soybeans, and in comparison with other orphan crops it has higher potential to be a new food crop.~~ However, we still lack the gene expression data about the GBSS and choline kinase genes in these the five ~~orphan~~ species. More ~~Therefore, this fine reference genomes together with the~~ transcriptomeic analysis and chemical test are still required to dig into their nutrition metabolism ~~data can be utilized and explored for detailed analyses~~ in future.

Formatted: Line spacing: Double, Tab stops: 0.77", Left

Formatted: Font: Italic

Formatted: Font: Italic

Formatted: Not Highlight

Formatted: Not Highlight

Identification of root nodule symbiosis pathway

Legumes (Fabaceae) are well known for their ability to fix nitrogen, which is an important trait to replenish nitrogen supply in soil and agricultural systems. Furthermore, being a part of human food production chain, ~~it has~~They have a major impact on global nitrogen cycle. Nitrogen-fixing plants can do this through root nodule symbiosis (RNS) using symbiotic nitrogen-fixing bacteria. In a previous report, RNS was revealed to be restricted to Fabales, Fagales, Cucurbitales, and Rosales that together form the monophyletic nitrogen-fixing clade, thus suggesting a predisposition event in their common ancestor, which enabled the subsequent evolution [74~~71~~]. Despite this genetic predisposition, many members of the nitrogen-fixing clade are non-fixer, within the legumes [75~~72~~]. This has led to the question whether the nodulation trait evolved independently in a convergent manner, or originated from a single evolutionary event followed by multiple losses. However, the answers to the above questions cannot be explained with the help of current genomic approaches, as the genomic information of nodulating species at present is limited to a single subfamily (Papilionoideae) in Fabaceae. Although the Mimosoideae subfamily under Fabaceae also contains nitrogen-fixing species, none of its members have been genome-sequenced. In this analysis, we identified 16 root nodulation symbiosis signal (Sym) pathway genes in three legumes (*V. subterranea*, *L. purpureus*, and *F. albida*) and two non-legumes (*S. birrea* and *M. oleifera*). First, we collected the protein sequences of previously reported genes in the Sym pathway of *L. japonicus* and *M. truncatula* [76~~73~~] (Figure 3). Using these sequences as bait, the Sym genes in *V. subterranea*, *L. purpureus*,

Formatted: Not Highlight

F. albida, *S. birrea*, and *M. oleifera* were predicted through reciprocal best hits generated by BLASTP search with an E-value of 1e-5 (Table 8). To verify the prediction with syntenic analysis, the 'all vs all' BLASTP results were subjected to MCSCANX [77~~74~~] with default parameters to generate the syntenic blocks. The result showed that most of the components in the pathway are conserved in the three legumes, except *MtNFP*/*LjNFR5*, *LjCASTOR*, *CCaMK*, *MtCRE1*/*LjLHK1*, and *NF-YA2*. While many components were missing in the non-legumes. Among the three legumes, the orthologous genes of *MtNFP/LjNFR5*, *LjCASTOR* and *MtIPD3/LjCYCLOPS* were absent in *F. albida*. As previously reported, the expression of *NIN* is lower in the *ipd3*-mutant line [78~~75~~], and the analysis of the *M. truncatula* mutant C31 showed that the Nod Factor Perception (NFP) gene plays an essential role in Nod factor perception at early stages of the symbiotic interaction [79~~76~~]. Meanwhile, the function of *IPD3* was proved to be partly redundant, which means other proteins phosphorylated by CCaMK probably could partly do the job when *IPD3* is absent [78~~75~~]. The ~~reason~~ difference in the components within RNS pathway (Table 8) together with the relatively weak nitrogen-fixing ability [80~~77~~] of~~why~~ *F. albida* thus make itself a good reference in the research of RNS diversification~~showed a relatively lower ability to fix nitrogen [77] could be explained by the loss of *IPD3*, *NFP*, and some proteins with lower efficiency which would have taken its place in *F. albida* (Table 8)~~.

Formatted: Not Highlight

Formatted: Not Highlight

Formatted: Not Highlight

Formatted: Not Highlight

Formatted: Not Highlight

Conclusion

This comprehensive study reports the sequencing, assembly, and annotation of ~~five~~ five ~~African orphan crop's~~ genomes of underutilized plants in Africa along with details of their key evolutionary features. The draft genomes of these species will serve as an important complementary resource for the non-model food crops especially the leguminous plants, and will be valuable for both agroforestry and evolutionary research. Improvement in these former underutilized plants~~orphan crops~~ using genomics-assisted tools and methods could bring food security for millions of people.

Availability of supporting data

The raw data from our genome project was deposited in the SRA (Sequence Read Archive) database of National Center for Biotechnology Information with Bioproject ID PRJNA453822 and PRJNA474418. The assembly and annotation of the five genomes and other supporting data, including BUSCO results, are available in the *GigaScience* GigaDB repository [81~~78~~].

Abbreviations

AOCC: African Orphan Crops Consortium; BLAST: Basic Local Alignment Search Tool; BUSCO: Benchmarking Universal Single-Copy Orthologues; CDS: Coding DNA sequence; CFU: The Conservation Farming Unit; LTR: long terminal repeat; TF: transcription factors; MITE: miniature inverted repeat transposable elements; NCBI: National Center for Biotechnology Information; PBS: primer binding site; PPT: poly

purine tract.

Formatted: Line spacing: Double

Author contributions

XL, XX, HY, JW, PSH, RJ, AV and YC conceived the project. They supervised the respective components: AOCC-ICRAF: DNA extraction, sample logistics and collection; BGI: data generation and analyses of the study. YC supervised the analyses. RK and SM collected and extracted the DNA and RNA. SB and FY performed the genome assembly. ML, XZL, SBW and LZL performed the genome annotation, gene family analysis and identification of genes related to root growth and root nodule symbiosis. YC, ML, XZL performed the phylogenetic analysis. YC, HL, SKS, PSH and AV wrote the manuscript. HRL and SFP sequenced the samples. SM, WKH, AM, PSH, JW, HMY revised the manuscript. All authors read, edited and approved the final manuscript.

Acknowledgments

This work was supported by the Shenzhen Municipal Government of China, (No. JCYJ20150831201643396 and No. JCYJ20150529150409546), as well as the funding from the State Key Laboratory of Agricultural Genomics (No. 2011DQ782025), and Guangdong Provincial Key Laboratory of Genome Read and Write (No. 2017B030301011). This work is part of 10KP project led by BGI-Shenzhen and China National GeneBank.

Table 1: Statistics of the final *de novo* genome assembly in *V. subterranea*, *L. purpureus*, *F. albida*, *S. birrea* and *M. oleifera*.

		V. subterranea		*L. purpureus*		*F. albida*		*S. birrea*		*M. oleifera*	
		**Contig**	**Scaffold**	**Contig**	**Scaffold**	**Contig**	**Scaffold**	**Contig**	**Scaffold**	**Contig**	**Scaffold**
**Length (bp)**	N90	3,804	75,271	785	860	8,254	95,167	3,661	21,833	6,676	57,837
	N80	7,872	197,296	8,009	61,348	16,321	251,730	7,649	82,385	16,503	241,828
	N70	11,464	325,826	16,144	205,392	24,165	380,587	11,885	155,416	25,754	441,152
	N60	15,122	474,616	24,010	359,168	32,440	534,880	16,393	243,236	35,081	644,014
	N50	19,154	640,666	32,223	621,373	42,029	692,039	21,349	335,449	45,268	957,246
	N40	23,828	865,081	42,690	950,808	53,479	881,230	26,914	485,585	58,406	1,446,587
	N30	29,382	1,133,817	54,401	1,489,002	69,167	1,197,388	33,914	705,409	74,710	1,878,891
	N20	36,928	1,503,436	70,790	1,971,744	92,147	1,501,241	43,984	1,098,843	96,626	2,565,629
	N10	49,695	2,049,645	95,643	2,606,483	139,388	1,925,526	62,875	2,089,533	136,952	3,296,678
**Number**	N90	29,245	1,087	26,272	9,409	16,834	1,132	17,585	1,537	5,524	366
	N80	20,188	664	9,869	715	11,420	727	11,678	787	3,574	191
	N70	14,829	453	6,576	366	8,198	514	8,313	499	2,542	125
	N60	10,943	315	4,630	222	5,898	370	6,001	332	1,833	84
	N50	7,932	220	3,244	138	4,151	263	4,277	214	1,295	56
	N40	5,532	147	2,204	86	2,791	179	2,929	131	876	37
	N30	3,590	93	1,403	52	1,728	114	1,857	74	553	24
	N20	2,024	52	776	29	912	64	1,012	36	300	13
	N10	806	22	306	12	326	26	387	12	112	6
Maximum length		148,612	3,684,321	240,194	5,699,750	529,842	4,746,824	227,874	5,850,796	449,426	4,637,711
Total length		512,516,846	535,052,523	385,303,786	395,472,305	644,456,383	653,726,905	322,977,033	330,983,508	213,739,255	216,759,177
Total number>=100bp		104,575	65,586	135,039	118,976	75,572	51,470	64,158	40,280	29,972	22,329
Total number>=2000bp		35,465	2,920	15,984	4,265	26,459	5,758	22,172	4,852	8,300	2,166

Percentage of N content (%)	4.21	2.57	1.42	2.42	1.39

Formatted: Font: Not Bold

Formatted: Normal

Formatted: Tab stops: 3.72", Left

Formatted: Line spacing: single, Tab stops: 3.72", Left

Formatted: Font: Not Bold

Table 2: Completeness evaluation of genome assembly using BUSCO database in five species.

BUSCOs	*V. subterranea*		*L. purpureus*		*F. albida*		*S. birrea*		*M. oleifera*	
	**NO.**	**P,%**	**NO.**	**P,%**	**NO.**	**P,%**	**NO.**	**P,%**	**NO.**	**P,%**
Complete single copy	1,244	86.39	1,258	87.40	1,231	85.50	1352	93.90	1,278	88.80
Complete duplicated	82	5.69	83	5.80	84	5.80	32	2.20	19	1.30
Fragmented	28	1.94	20	1.40	34	2.40	21	1.50	23	1.60
Missing	86	5.97	79	5.40	91	6.30	35	2.40	120	8.30
Total	1440	/	1440	/	1440	/	1440	/	1440	/

Table 3: The gene coverage of the candidate species based on transcriptome data

Species	Dataset	Number	Total Length (bp)	Base Coverage by Assembly (%)	Sequence coverage by assembly (%)
V. subterranea	All	**116,223**	161,077,155	89.61	98.21
	>200bp	116,223	161,077,155	89.61	98.21
	>500bp	72,139	147,068,299	89.03	98.00
	>1000bp	47,952	129,884,929	88.33	97.52
L. purpureus	All	**86,867**	80,837,182	93.59	99.25
	>200bp	86,867	80,837,182	93.59	99.25
	>500bp	41,252	66,764,786	92.94	99.18
	>1000bp	24,627	55,074,989	92.32	99.02
F. albida	All	**50,294**	46,650,067	93.62	98.85
	>200bp	50,294	46,650,067	93.62	98.85
	>500bp	26,352	39,282,694	93.32	99.05
	>1000bp	15,569	31,560,858	92.78	98.95
M. oleifera	All	**60964**	57114636	88.98	92.16
	>200bp	60964	57114636	88.98	92.16
	>500bp	29581	47523018	88.85	92.69
	>1000bp	18322	39528310	88.70	92.99

Table 4: The proportion of different classes of repeats (%) in five species.

Repeat Type	*V. subterranea*		*L. purpureus*		*F. albida*		*S. birrea*		*M. oleifera*	
	**% in genome**	**Length (bp)**	**% in genome**	**Length(bp)**	**% in genome**	**Length (bp)**	**% in genome**	**Length (bp)**	**%in genome**	**Length (bp)**
SINE	0	313	0.005	19,444	< 0.01	1,966	0.02	69,836	0.11	248,569
LINE	0.25	1,387,567	0.45	1,784,785	0.91	6,003,271	0.19	647,579	1.83	3,970,802
LTR	19.77	105,828,735	23.78	94,062,428	44.65	291,901,514	38.78	128,362,381	22.69	49,200,625
DNA	7.15	38,294,871	4.76	18,851,402	4	26,164,519	1.76	5,829,982	5.81	12,599,607
Satellite	0.01	71,679	0.02	107,451	0.01	110,749	0	18,597	0.74	1,623,399
Simple repeat	0.35	1,922,719	0.2	821,773	0.04	308,481	0.04	153,135	0.29	630,662
Others	11.94	63,926,350	8.95	35,400,400	6.48	42,426,306	5.11	16,918,179	10.35	22,439,026
Total	**38.35**	**205,189,285**	**37.18**	**147,050,327**	**54.86**	**358,653,534**	**45.18**	**149,551,125**	**40.57**	**87,944,150**

Formatted: Font color: Red

Formatted: Font color: Red

Formatted: Font color: Red

Formatted: Font color: Red

Formatted: Font color: Red

Formatted: Font color: Red

Formatted: Font color: Red

Formatted: Font color: Red

Formatted: Font color: Red

Table 5. Various gene structure parameters of *V. subterranea*, *L. purpureus*, *F. albida*, *M. oleifera* and *S. birrea*.

	V. subterranea	*L. purpureus*	*F. albida*	*M. truncatula*	*G. max*
Protein-coding gene number	31,707	20,946	28,979	50,358	55,137
Mean gene length (bp)	3,287	3,696	3,396	2,334	3,144
Mean cds length (bp)	1,163	1,276	1,207	986	1,169
Mean exons per gene	5	5	5	4	5
Mean exon length (bp)	222	239	226	243	232
Mean intron length (bp)	501	557	504	440	488

	S. birrea	*M. oleifera*	*C. papaya*	*T. cacao*	*C. sinensis*
Protein-coding gene number	18,937	18,451	24,107	41,951	35,182
Mean gene length (bp)	3,561	3,308	2,531	3,684	3,797
Mean cds length (bp)	1,343	1,238	962	1,323	1,424
Mean exons per gene	6	5	4	6	6
Mean exon length (bp)	239	232	223	223	237
Mean intron length (bp)	479	478	473	479	475

Table 6. Annotation of non-coding RNA genes in *V. subterranea*, *L. purpureus*, *F. albida*, *S. birrea* and *M. oleifera* genome.

		miRNA	tRNA	rRNA					snRNA				Total
				Total rRNA	18S	28S	5.8S	5S	Total snRNA	CD-box	HACA-box	splicing	
V. subterranea	**Copy (w)**	102	756	1,080	55	62	17	946	523	327	47	149	2,461
	**Average length (bp)**	122	75	124	560	126	124	99	117	100	133	149	110
	**Total length (bp)**	12,466	56,639	134,185	30,798	7,793	2,110	93,484	61,006	32,643	6,236	22,127	264,296
	**% of genome**	0.0023%	0.0106%	0.0251%	0.0058%	0.0015%	0.0004%	0.0175%	0.0114%	0.0061%	0.0012%	0.0041%	0.0494%
L. purpureus	**Copy (w)**	109	611	633	213	283	53	84	457	278	48	131	1,810
	**Average length (bp)**	123	75	227	446	121	135	84	118	97	133	158	136
	**Total length (bp)**	13,398	45,748	143,466	95,074	34,186	7,177	7,029	54,029	26,915	6,371	20,743	256,641
	% of genome	0.0034%	0.0116%	0.0363%	0.0240%	0.0086%	0.0018%	0.0018%	0.0137%	0.0068%	0.0016%	0.0052%	0.0649%
F. albida	**Copy(w)**	126	458	1,008	25	26	6	951	1,996	1,836	42	118	3,588
	**Average length (bp)**	122	75	107	321	118	118	101	108	106	132	138	103
	**Total length (bp)**	15,364	34,388	107,518	8,034	3,063	710	95,711	216,482	194,676	5,548	16,258	373,752
	**% of genome**	0.0024%	0.0053%	0.0164%	0.0012%	0.0005%	0.0001%	0.0146%	0.0331%	0.0298%	0.0008%	0.0025%	0.0572%
S. birrea	**Copy (w)**	106	564	313	80	57	16	160	841	638	34	169	1,824
	**Average length (bp)**	122	75	142	240	113	103	106	115	105	124	148	113
	**Total length (bp)**	12,899	42,181	44,378	19,239	6,460	1,644	17,035	96,517	67,216	4,217	25,084	195,975
	**% of genome**	0.0039%	0.0127%	0.0134%	0.0058%	0.0020%	0.0005%	0.0051%	0.0292%	0.0203%	0.0013%	0.0076%	0.0592%
M. oleifera	**Copy (w)**	111	1,241	8,406	3,256	3,808	1,182	160	229	119	38	72	9,987

**Average length (bp)**	119	75	309	608	113	150	69	119	97	132	147	622
**Total length (bp)**	13,161	93,620	2,598,079	1,979,080	430,280	177,612	11,107	27,158	11,578	4,999	10,581	2,732,018
**% of genome**	0.0061%	0.0432%	1.1986%	0.9130%	0.1985%	0.0819%	0.0051%	0.0125%	0.0053%	0.0023%	0.0049%	1.2604%

Table 7. Statistics of functional annotation of protein-coding genes in the *V. subterranea*, *L. purpureus*, *F. albida*, *S. birrea* and *M. oleifera* genome.

	V. subterranea		***L. purpureus***		***F. albida***		***S. birrea***		***M. oleifera***	
	**Number of genes**	**Percentage (%)**	**Number of genes**	**Percentage (%)**	**Number of genes**	**Percentage (%)**	**Number of genes**	**Percentage (%)**	**Number of genes**	**Percentage (%)**
Nr-Annotated	31,013	97.81	20,540	98.06	27,021	93.24	18,547	97.94	18,203	98.65
Swissprot-Annotated	22,496	70.95	15,905	75.93	21,247	73.32	15,513	81.92	15,109	81.88
KEGG-Annotated	22,141	69.83	14,699	70.18	20,184	69.65	14,623	77.22	14,044	76.11
COG-Annotated	10,814	34.11	7,854	37.50	10,526	36.32	7,715	40.74	7,662	41.52
TrEMBL-Annotated	30,964	97.66	20,489	97.82	26,828	92.58	18,477	97.57	18,193	98.60
Interpro-Annotated	22,744	71.73	18,911	90.28	25,401	87.65	15,537	82.05	15,134	82.02
GO-Annotated	18,894	59.59	13,811	65.94	15,182	52.39	11,505	60.75	11,877	64.37
Overall	31,074	98.00	20,574	98.22	27,118	93.58	18,573	98.08	18,236	98.83
Unannotated	633	2.00	372	1.78	1,861	6.86	364	1.92	216	1.17

Table 8: The nitrogen fixation orthologous in *V. subterranea, L. purpureus, F. albida, M. oleifera and S. birrea*.

Gene	*V. subterranea*	*L. purpureus*	*F. albida*	*M. oleifera*	*S. birrea*
MtLYK3/LjNFR1	Vigsu176S22567_VIGSU	Labpu216S12485_LABPU	Faial2789S13350_FAIAL	——	——
MtNFP/LjNFR5	Vigsu1898S04417_VIGSU	Labpu54S03611_LABPU	——	——	Sclbi409S02347_SCLBI
MtDMI2/LjSYMRK	Vigsu107959S16599_VIGSU	Labpu4785S15752_LABPU	Faial1833S08172_FAIAL	Morol36160S02362_MOROL	Sclbi59955S15146_SCLBI
LjCASTOR	Vigsu108012S17109_VIGSU	Labpu27S13484_LABPU	——	——	———
MtHMGR1	——	——	——	——	———
MtDMI1/LjPOLLUX	Vigsu108496S19983_VIGSU	Labpu4332S15101_LABPU	Faial363S16033_FAIAL	Morol36085S07630_MOROL	———
NSP1	Vigsu2922S08781_VIGSU	Labpu723S04373_LABPU	Faial1104S01086_FAIAL	Morol36102S01150_MOROL	Sclbi5005S02593_SCLBI
NSP2	Vigsu107793S01507_VIGSU	Labpu887S08157_LABPU	Faial757S23006_FAIAL	Morol36224S03158_MOROL	Sclbi2944S01716_SCLBI
CCaMK	Vigsu91S05737_VIGSU	——	Faial752S22546_FAIAL	——	———
MtIPD3/LjCYCLOPS	Vigsu104856S09608_VIGSU	Labpu701S17462_LABPU	——	——	Sclbi2578S10386_SCLBI
NIN	Vigsu273S23676_VIGSU	Labpu165S10337_LABPU	Faial788S23538_FAIAL	Morol36195S02810_MOROL	Sclbi2838S04948_SCLBI
MtCRE1/LjLHK1	——	Labpu2293S02028_LABPU	Faial1226S02883_FAIAL	——	——
NF-YA1	Vigsu107799S13964_VIGSU	Labpu193775S11413_LABPU	Faial246S12019_FAIAL	Morol36154S02289_MOROL	Sclbi406S12278_SCLBI
NF-YA2	——	——	Faial858S26716_FAIAL	——	———
MtERN1	Vigsu107612S00570_VIGSU	Labpu210S01798_LABPU	Faial719S21851_FAIAL	Morol36040S00658_MOROL	Sclbi1920S01196_SCLBI
MtERN2	Vigsu108137S07511_VIGSU	Labpu448S03276_LABPU	Faial4604S17896_FAIAL	——	———

Additional files

Figure S1: K-mer (K=17) analysis of five genomes.

Figure S2: Distribution of sequencing depth of the assembly data.

Figure S3: The GC content.

Figure S4: Comparison of GC content across closely related species.

Figure S5: Statistics of gene models in *V. subterranea*, *L. purpureus*, *F. albida*, *M. oleifera*, *S.birrea*.

Figure S6: Expansion and contraction of gene families.

Formatted: Not Highlight

Table S1. Statistics of the raw and clean data of DNA sequencing.

Table S2. Summary statistics of the transcriptome data in four species.

Table S3. Estimation of genome size based on K-mer statistics in five species.

Table S4. BUSCO evaluation of the annotated protein-coding genes in five species.

Table S5. Analysis of gene families of different species.

Table S6. Enriched pathways of unique paralogs genes in families.

Formatted: Font: Not Bold

Table S7. Enriched GO terms (level 3) of unique paralogs genes in families.

Formatted: Font: Not Bold

Table S8. Enriched GO terms (level 3) of genes in families with expansion.

Formatted: Font: Bold

Table S9. Enriched pathways of genes in families with expansion.

Formatted: Font: Bold

Table S10~~8~~. The copy numbers of protein biosynthesis related genes in each species.

Table S11~~9~~. The copy numbers of starch biosynthesis genes in each species.

Table S12~~0~~. The copy numbers of fatty acid synthesis and storage related genes in each species.

Table S13~~1~~. The copy number of fatty acid degradation related genes in each species.

Table S14~~2~~. The numbers of Transcription factor in the studied species.

References

1. United Nations, Department of Economic and Social Affairs, Population Division. World population prospects: the 2017 revision, Key Findings and Advance Tables. 2017. Working Paper No. ESA/P/WP/248.

2. Development Initiatives. Global nutrition report 2017: nourishing the SDGs. Bristol, UK: Development Initiatives. 2017.

3. Mouillé, B., Charrondière, U. R., & Burlingame. The contribution of plant genetic resources to health and dietary diversity. Thematic Background Study. 2010.

4. Varshney RK, Chen W, Li Y, Bharti AK, Saxena RK, Schlueter JA, et al. Draft genome sequence of pigeonpea (*Cajanus cajan*), an orphan legume crop of resource-poor farmers. Nat Biotechnol. 2011;30:83-89. doi:10.1038/nbt.2022.

5. Foyer CH, Lam H-M, Nguyen HT, Siddique KHM, Varshney RK, Colmer TD, et al. Neglecting legumes has compromised human health and sustainable food production. Nat Plants. 2016;2:16112. doi:10.1038/nplants.2016.112.

6. Borget M. Food legumes. In: The Tropical Agriculturalist, CTA Macmillan. 1992.

7. Linnemann A.R, Azam–Ali S.N. Bambara groundnut (*Vigna subterranea*) literature review: A revised and updated bibliography. Tropical Crops Communication No. 7. 1993.

8. Gbaguidi AA, Dansi A, Dossou-Aminon I, Gbemavo DSJC, Orobiyi A, Sanoussi F, et al. Agromorphological diversity of local Bambara groundnut (*Vigna subterranea* (L.) Verdc.) collected in Benin. Genet Resour Crop Evol. 2018;65(4):1159-1171. doi:10.1007/s10722-017-0603-4.

9. Kang YJ, Kim SK, Kim MY, Lestari P, Kim KH, Ha B-K, et al. Genome

Formatted: Font color: Red

sequence of mungbean and insights into evolution within *Vigna* species. Nat Commun. 2014;5:5443. doi:10.1038/ncomms6443.

10. Yang K, Tian Z, Chen C, Luo L, Zhao B, Wang Z, et al. Genome sequencing of adzuki bean (*Vigna angularis*) provides insight into high starch and low fat accumulation and domestication. Proc Natl Acad Sci U S A. 2015;112(43):13213-13218. doi:10.1073/pnas.1420949112.

11. Jung IL. Soluble extract from *Moringa oleifera* leaves with a new anticancer activity. PLoS One. 2014;9(4):e95492. doi:10.1371/journal.pone.0095492.

12. Leone A, Spada A, Battezzati A, Schiraldi A, Aristil J and Bertoli S. Cultivation, genetic, ethnopharmacology, phytochemistry and pharmacology of *Moringa oleifera* Leaves: An Overview. Int J Mol Sci. 2015;16(6):12791-12835. doi:10.3390/ijms160612791.

13. Lea M. Bioremediation of turbid surface water using seed extract from *Moringa oleifera* Lam. (drumstick) tree. Curr Protoc Microbiol. 2014;33:1G.2.1-G.2.8. doi:10.1002/9780471729259.mc01g02s16.

14. Mabapa MP, Ayisi KK, Mariga IK, Mohlabi RC and Chuene RS. Production and utilization of moringa by farmers in Limpopo Province, South Africa. International Journal of Agricultural Research. 1962;12(4):160-171. doi:10.3923/ijar.2017.160.171.

15. Tian Y, Zeng Y, Zhang J, Yang CG, Yan L, Wang XJ, et al. High quality reference genome of drumstick tree (*Moringa oleifera* Lam.), a potential perennial crop. Science China Life Sciences. 2015;58(7):627-638. doi:10.1007/s11427-015-4872-x.

16. Maass BL, Knox MR, Venkatesha SC, Angessa TT, Ramme S and Pengelly BC. *Lablab purpureus*-a crop lost for Africa? Trop Plant Biol. 2010;3(3):123-135.

Formatted: Font: Italic, Font color: Red

Formatted: Font color: Red

Formatted: Font color: Red

doi:10.1007/s12042-010-9046-1.

17. Robotham O and Chapman M. Population genetic analysis of hyacinth bean (*Lablab purpureus* (L.) Sweet, Leguminosae) indicates an East African origin and variation in drought tolerance. Genet Resour Crop Evol. 2017;64(1):139-148. doi:10.1007/s10722-015-0339-y.

18. Kamotho GN. Evaluation of adaptability potential and genetic diversity of Kenyan Dolichos bean germplasm. PhD thesis. 2015.

19. Vankatesha S.C. Molecular characterization and development of mapping populatuions for construction of genetic map in dolichos bean. PhD thesis. 2012.

20. Mokgolodi NC, Setshogo MP, Shi L-l, Liu Y-j and Ma C. Achieving food and nutritional security through agroforestry: a case of *Faidherbia albida* in sub-Saharan Africa. For. Stud. China. 2011;13(2):123-131. doi:10.1007/s11632-011-0202-y.

21. Garrity DP, Akinnifesi FK, Ajayi OC, Weldesemayat SG, Mowo JG, Kalinganire A, et al. Evergreen agriculture: a robust approach to sustainable food security in Africa. Food Sec. 2010;2(3):197-214. doi:10.1007/s12571-010-0070-7.

22. DUNHAM KM. Biomass dynamics of herbaceous vegetation in Zambezi riverine woodlands. African Journal of Ecology. 1990;28(3):200-212. doi:10.1111/j.1365-2028.1990.tb01153.x.

23. Barnes RD and Fagg CW. *Faidherbia albida* monograph and annotated bibliography. Oxford Forestry Inst. 2003;41-267

24. Nerd A, Mizrahi Y, Janick J and Simon JE. Domestication and introduction of marula (*Sclerocarya birrea* subsp. *caffra*) as a new crop for the Negev Desert of Israel. New crops. 1993;496-499.

25. Mng'Omba SA, Sileshi GW, Jamnadass R, Akinnifesi FK and Mhango J. Scion and stock diameter size effect on growth and fruit production of *Sclerocarya birrea* (Marula) trees. J Hortic For. 2012;4(9):153-60.

26. Gouwakinnou GN, Lykke AM, Assogbadjo AE and Sinsin B. Local knowledge, pattern and diversity of use of *Sclerocarya birrea*. J Ethnobiol Ethnomed. 2011;7 (1):1-9. doi:10.1186/1746-4269-7-8.

27. Yang T and Wu C. DNA Extraction for plant samples by CTAB. protocols.io. 2018; dx.doi.org/10.17504/protocols.io.pzqdp5w

28. Luo R, Liu B, Xie Y, Li Z, Huang W, Yuan J, et al. SOAPdenovo2: an empirically improved memory-efficient short-read de novo assembler. GigaScience. 2012;1(1):1-6. doi:10.1186/2047-217X-1-18.

29. Teh BT, Lim K, Yong CH, Ng CCY, Rao SR, Rajasegaran V, et al. The draft genome of tropical fruit durian (*Durio zibethinus*). Nat Genet. 2017;49:1633-1641. doi:10.1038/ng.3972.

30. Simao FA, Waterhouse RM, Ioannidis P, Kriventseva EV and Zdobnov EM. BUSCO: assessing genome assembly and annotation completeness with single-copy orthologs. Bioinformatics. 2015;31(19):3210-3212. doi:10.1093/bioinformatics/btv351.

31. Chang Z, Li G, Liu J, Zhang Y, Ashby C, Liu D, et al. Bridger: a new framework for de novo transcriptome assembly using RNA-seq data. Genome Biol. 2015;16:30. doi:10.1186/s13059-015-0596-2.

32. Kent WJ. BLAT--the BLAST-like alignment tool. Genome Res. 2002;12(4):656-664. doi:10.1101/gr.229202.

33. Li R, Yu C, Li Y, Lam TW, Yiu SM, Kristiansen K, et al. SOAP2: an improved ultrafast tool for short read alignment. Bioinformatics. 2009;25(15):1966-1967.

doi:10.1093/bioinformatics/btp336.

34. Tarailo-Graovac M and Chen N. Using RepeatMasker to identify repetitive elements in genomic sequences. Curr Protoc Bioinformatics. 2009;25(1) 4.10.1-4.10.14. doi:10.1002/0471250953.bi0410s25.

35. Han Y and Wessler SR. MITE-Hunter: a program for discovering miniature inverted-repeat transposable elements from genomic sequences. Nucleic Acids Res. 2010;38(22):e199-e199. doi:10.1093/nar/gkq862.

36. Ellinghaus D, Kurtz S and Willhoeft U. LTRharvest, an efficient and flexible software for de novo detection of LTR retrotransposons. BMC Bioinformatics. 2008;9:18. doi:10.1186/1471-2105-9-18.

37. Gremme G, Steinbiss S and Kurtz S. GenomeTools: a comprehensive software library for efficient processing of structured genome annotations. IEEE/ACM Trans Comput Biol Bioinform. 2013;10(3):645-656. doi:10.1109/tcbb.2013.68.

38. Steinbiss S, Willhoeft U, Gremme G and Kurtz S. Fine-grained annotation and classification of de novo predicted LTR retrotransposons. Nucleic Acids Res. 2009;37(21):7002-7013. doi:10.1093/nar/gkp759.

39. Edgar RC. MUSCLE: multiple sequence alignment with high accuracy and high throughput. Nucleic Acids Res. 2004;32(5):1792-1797. doi:10.1093/nar/gkh340.

40. Campbell MS, Holt C, Moore B and Yandell M. Genome annotation and curation using MAKER and MAKER-P. Curr Protoc Bioinformatics. 2014;48(1): 4.11.1-4.11.39. doi:10.1002/0471250953.bi0411s48.

41. Haas BJ, Papanicolaou A, Yassour M, Grabherr M, Blood PD, Bowden J, et al. De novo transcript sequence reconstruction from RNA-seq using the Trinity platform for reference generation and analysis. Nat Protoc. 2013;8:1494–1512. doi:10.1038/nprot.2013.084.

42. Haas BJ, Salzberg SL, Zhu W, Pertea M, Allen JE, Orvis J, et al. Automated eukaryotic gene structure annotation using EVidenceModeler and the program to assemble spliced alignments. Genome Biol. 2008;9(1):R7. doi:10.1186/gb-2008-9-1-r7.

43. Stanke M, Schoffmann O, Morgenstern B and Waack S. Gene prediction in eukaryotes with a generalized hidden Markov model that uses hints from external sources. BMC Bioinformatics. 2006;7:62. doi:10.1186/1471-2105-7-62.

44. Lomsadze A, Ter-Hovhannisyan V, Chernoff YO and Borodovsky M. Gene identification in novel eukaryotic genomes by self-training algorithm. Nucleic Acids Res. 2005;33(20):6494-6506. doi:10.1093/nar/gki937.

45. Korf I. Gene finding in novel genomes. BMC Bioinformatics. 2004;5:59. doi:10.1186/1471-2105-5-59.

46. Nawrocki EP, Burge SW, Bateman A, Daub J, Eberhardt RY, Eddy SR, et al. Rfam 12.0: updates to the RNA families database. Nucleic Acids Res. 2015;43(D1):D130-D137. doi:10.1093/nar/gku1063.

47. Lowe TM and Chan PP. tRNAscan-SE On-line: integrating search and context for analysis of transfer RNA genes. Nucleic Acids Res. 2016;44(W1):W54-W57. doi:10.1093/nar/gkw413.

48. Tanabe M and Kanehisa M. Using the KEGG database resource. Curr Protoc Bioinformatics. 2012; 38(1):1.12.1-1.12.43. doi:10.1002/0471250953.bi0112s38.

49. Tatusov RL, Koonin EV and Lipman DJ. A genomic perspective on protein families. Science. 1997;278(5338):631-637.

50. Boeckmann B, Bairoch A, Apweiler R, Blatter MC, Estreicher A, Gasteiger E,

et al. The SWISS-PROT protein knowledgebase and its supplement TrEMBL in 2003. Nucleic Acids Res. 2003;31(1):365-370.

51. Jones P, Binns D, Chang HY, Fraser M, Li W, McAnulla C, et al. InterProScan 5: genome-scale protein function classification. Bioinformatics. 2014;30(9):1236-1240. doi:10.1093/bioinformatics/btu031.

52. Finn RD, Mistry J, Tate J, Coggill P, Heger A, Pollington JE, et al. The Pfam protein families database. Nucleic Acids Res. 2010;38 suppl 1:D211-D222. doi:10.1093/nar/gkp985.

53. Letunic I, Doerks T and Bork P. SMART 6: recent updates and new developments. Nucleic Acids Res. 2009;37 suppl 1:D229-D232. doi:10.1093/nar/gkn808.

54. Mi H, Muruganujan A, Casagrande JT and Thomas PD. Large-scale gene function analysis with the PANTHER classification system. Nat Protoc. 2013;8:1551-1566. doi:10.1038/nprot.2013.092

55. Attwood TK, Bradley P, Flower DR, Gaulton A, Maudling N, Mitchell AL, et al. PRINTS and its automatic supplement, prePRINTS. Nucleic Acids Res. 2003;31(1):400-402.

56. Corpet F, Servant F, Gouzy J and Kahn D. ProDom and ProDom-CG: tools for protein domain analysis and whole genome comparisons. Nucleic Acids Res. 2000;28(1):267-269.

57. Stichting C, Centrum M and Dongen SV. A Cluster Algorithm for Graphs. Information Systems [INS]. 2000:1-40.

58. Guindon S, Dufayard JF, Lefort V, Anisimova M, Hordijk W and Gascuel O. New algorithms and methods to estimate maximum-likelihood phylogenies: assessing the performance of PhyML 3.0. Syst Biol. 2010;59(3):307-321.

doi:10.1093/sysbio/syq010.

59. Yang Z. PAML 4: Phylogenetic analysis by maximum likelihood. Mol Biol Evol. 2007;24(8):1586-1591. doi:10.1093/molbev/msm088.

60. He N, Zhang C, Qi X, Zhao S, Tao Y, Yang G, et al. Draft genome sequence of the mulberry tree *Morus notabilis*. Nat Commun. 2013;4:2445. doi:10.1038/ncomms3445.

61. Lavin M, Herendeen PS, Wojciechowski MF and Linder P. Evolutionary rates analysis of leguminosae implicates a rapid diversification of lineages during the Tertiary. Syst Biol. 2005;54(4):575-594. doi:10.1080/10635150590947131.

62. De Bie T, Cristianini N, Demuth JP and Hahn MW. CAFE: a computational tool for the study of gene family evolution. Bioinformatics. 2006;22(10):1269-1271. doi:10.1093/bioinformatics/btl097.

63. Bernhardt C, Lee MM, Gonzalez A, Zhang F, Lloyd A and Schiefelbein J. The bHLH genes GLABRA3 (GL3) and ENHANCER OF GLABRA3 (EGL3) specify epidermal cell fate in the Arabidopsis root. Development. 2003;130(26):6431-6439. doi:10.1242/dev.00880.

64. Paponov IA, Paponov M, Teale W, Menges M, Chakrabortee S, Murray JA, et al. Comprehensive transcriptome analysis of auxin responses in Arabidopsis. Mol Plant. 2008;1(2):321-337. doi:10.1093/mp/ssm021.

65. Vanneste S, Rybel BD, Beemster GTS, Ljung K, Smet ID, Isterdael GV, et al. Cell cycle progression in the pericycle is not sufficient for SOLITARY ROOT/IAA14-mediated lateral root initiation in *Arabidopsis thaliana*. Plant Cell. 2005;17(11):3035-3050. doi:10.1105/tpc.105.035493.

66. Vandenbeldt RJ. Faidherbia albida in the West African semi-arid tropics.

Formatted: Indent: Left: 0", First line: 0"

Formatted: Font color: Red

ICRISAT. 1992. p. 107-110.

67. Jang YE, Kim MY, Shim S, Lee J and Lee S-H. Gene expression profiling for seed protein and oil synthesis during early seed development in soybean. Genes Genom. 2015;37(4):409-418. doi:10.1007/s13258-015-0269-2.

68. Bamshaiye OM, Adegbola JA and Bamishaiye EI. Bambara groundnut : an under-utilized nut in Africa. Adv Agric Biotechnol. 2011;1:60-72.

69. Raigond P, Ezekiel R and Raigond B. Resistant starch in food: a review. J Sci Food Agric. 2015;95(10):1968-1978.

70. Zhou H, Wang L, Liu G, Meng X, Jing Y, Shu X, et al. Critical roles of soluble starch synthase SSIIIa and granule-bound starch synthase Waxy in synthesizing resistant starch in rice. Proc Natl Acad Sci U S A. 2016;113(45):12844-12849. doi:10.1073/pnas.1615104113.

71. Bird AR, Flory C, Davies DA, Usher S and Topping DL. A novel barley cultivar (*Himalaya 292*) with a specific gene mutation in starch synthase IIa raises large bowel starch and short-chain fatty acids in rats. J Nutr. 2004;134(4):831-835. doi:10.1093/jn/134.4.831.

72. Morre DJ, Nyquist S and Rivera E. Lecithin biosynthetic enzymes of onion stem and the distribution of phosphorylcholine-cytidyl transferase among cell fractions. Plant Physiol. 1970;45(6):800-804.

73. Johnson KD and Kende H. Hormonal control of lecithin synthesis in barley aleurone cells: regulation of the CDP-choline pathway by gibberellin. Proc Natl Acad Sci U S A. 1971;68(11):2674-2677.

74. Soltis DE, Soltis PS, Morgan DR, Swensen SM, Mullin BC, Dowd JM, et al. Chloroplast gene sequence data suggest a single origin of the predisposition for symbiotic nitrogen fixation in angiosperms. Proc Natl Acad Sci U S A.

1995;92(7):2647-2651.

75. Doyle JJ. Phylogenetic perspectives on the origins of nodulation. Mol Plant Microbe Interact. 2011;24(11):1289-1295. doi:10.1094/MPMI-05-11-0114.

76. Geurts R, Xiao TT and Reinhold-Hurek B. What does it take to evolve a nitrogen-fixing endosymbiosis? Trends Plant Sci. 2016;21 (3):199–208. doi:10.1016/j.tplants.2016.01.012.

77. Wang Y, Tang H, Debarry JD, Tan X, Li J, Wang X, et al. MCScanX: A toolkit for detection and evolutionary analysis of gene synteny and collinearity. Nucleic Acids Res. 2012;40(7):e49. doi:10.1093/nar/gkr1293.

78. Horváth B, Li HY, Domonkos Á, Halász G, Gobbato E, Ayaydin F, et al. *Medicago truncatula* IPD3 is a member of the common symbiotic signaling pathway required for rhizobial and mycorrhizal symbioses. Mol Plant Microbe Interact. 2011;24(11):1345-1358. doi:10.1094/MPMI-01-11-0015.

79. Amor BB, Shaw SL, Oldroyd GED, Maillet F, Penmetsa RV, Cook D, et al. The NFP locus of *Medicago truncatula* controls an early step of Nod factor signal transduction upstream of a rapid calcium flux and root hair deformation. Plant J. 2003;34(4):495-506.

80. Ndoye I, Gueye M, Danso SKA and Dreyfus B. Nitrogen fixation in *Faidherbia albida*, *Acacia raddiana*, *Acacia senegal* and *Acacia seyal* estimated using the ^{15}N isotope dilution technique. Plant Soil. 1995;172(2):175-180. doi:10.1007/BF00011319.

81. Chang Y, Liu H, Liu M, Liao X, Sahu SK, Fu Y, Song B; Cheng S, Kariba R, Muthemba S, Hendre PS, Mayes S, Ho WK, Kendabie P, Wang S, Li L, Muchugi A, Jamnadass R, Lu H, Peng S, Deynze AV, Simons A, Yana-Shapiro H, Xu X, Yang H, Wang J, Liu X. Supporting data for "The draft genomes of

Formatted: Font color: Red

five agriculturally important African orphan crops". GigaScience Database 2018. http://dx.doi.org/10.5524/100504

Figure 1. Phylogenetic and evolutionary analysis. The scale bar indicates 10 million years. The values at the branch points indicate the estimates of divergence time (mya), while the blue numbers show the divergence time (million years ago, Mya), and the red nodes indicate the previously published calibration times. *V.sub* showed the seeds of *Vigna subterranea*, *L.pur* showed the flowers of *Lablab purpureus*, *F.alb* showed the seed pods of *Faidherbia albida*, *S.bir* showed the fruit of *Sclerocarya birrea*, *M.ole* showed the flowers of *Moringa oleifera*.

Figure 2. (A) The groups of orthologues shared among the *Lablab purpureus* (LABPU), *Faidherbia albida* (FAIAL), *Glycine max* (GLYMA), *Medicago truncatula* (MEDTR), *Vigna subterranea* (VIGSU). (B) The groups of orthologues shared among the *Sclerocarya birrea* (SCLBI), *Moringa oleifera* (MOROL), *Carica papaya* (CARPA), *Citrus sinensis* (CITSI), *Theobroma cacao* (THECA). Venn diagram generated by http://bioinformatics.psb.ugent.be/webtools/Venn/.

Figure 3. The common symbiosis signaling pathway. A total of 16 root nodulation symbiosis signal (Sym) pathway genes were identified in three legumes (*V. subterranea*, *L. purpureus*, and *F. albida*) and two non-legumes (*S. birrea* and *M. oleifera*). Lj : *L. japonicas*; Mt: *Medicago truncatula*, and LCOs: Lipochitooligosaccharides.

Figure 4. The percentage of transcription factors in five orphan species. Blastp tools was utilized to search against 58 plant transcription factor families obtained from PlantTFDB (http://planttfdb.cbi.pku.edu.cn/) (Additional file 2: Table S142). In this figure, MADS include M-type_MADS and MIKC_MADS. MYB include MYB and

MYB_related. NF-YA/B/C include NF-YA, NF-YB and NT-YC. "Others" comprises 31 types of transcription factors (E2F/DP, Nin-like, TALE, YABBY, GeBP, BES1, DBB, CO-like, CPP, SBP, STAT, WOX, BBR-BPC, CAMTA, AP2, ZF-HD, S1Fa-like, ARR-B, SRS, GRF, LSD, NF-X1, EIL, RAV, HRT-like, HB-PHD, VOZ, Whirly, SAP, LFY, NZZ/SPL) whose percentage was less than 1%.

Figure 5: The identification of the genes involved in the starch biosynthesis pathway. The identified genes involving in starch synthesis are shown in red. The number of homolog genes are presented in the additional file 2 Table S11~~4~~. (AGP: ADP-glucose pyrophosphorylase; AGPL: AGP large subunit; AGPS: AGP small subunit; PHOH: Starch phosphorylase H (Cytosolic type); GBSS: granule-bound starch synthase; SS: soluble starch synthase; BE: starch branching enzyme; ISA: isoamylase DPE: starch debranching enzyme).

Figure 1

Click here to access/download;Figure;Figure1.pdf

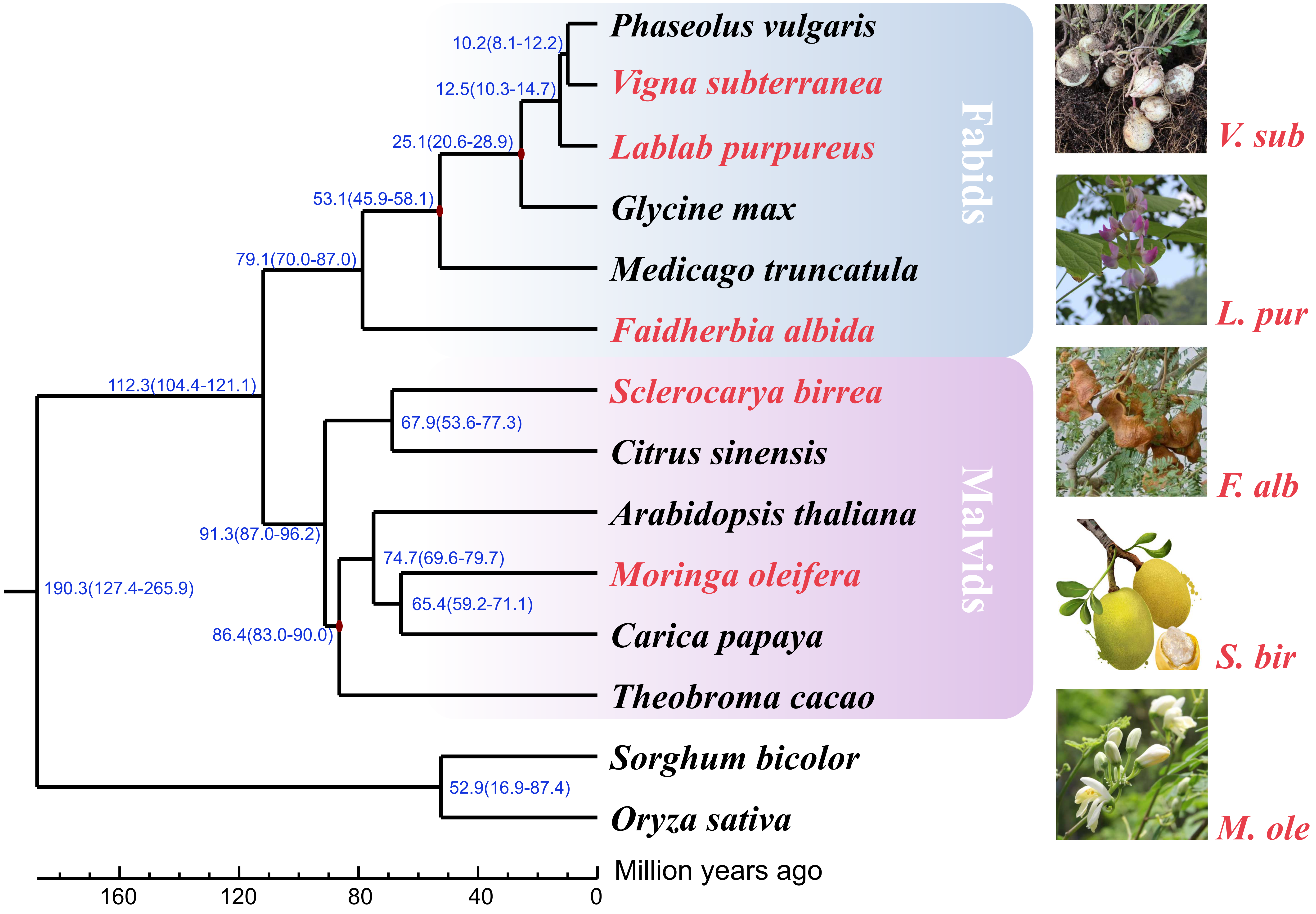

Figure 2

Click here to access/download;Figure;Figure2.pdf

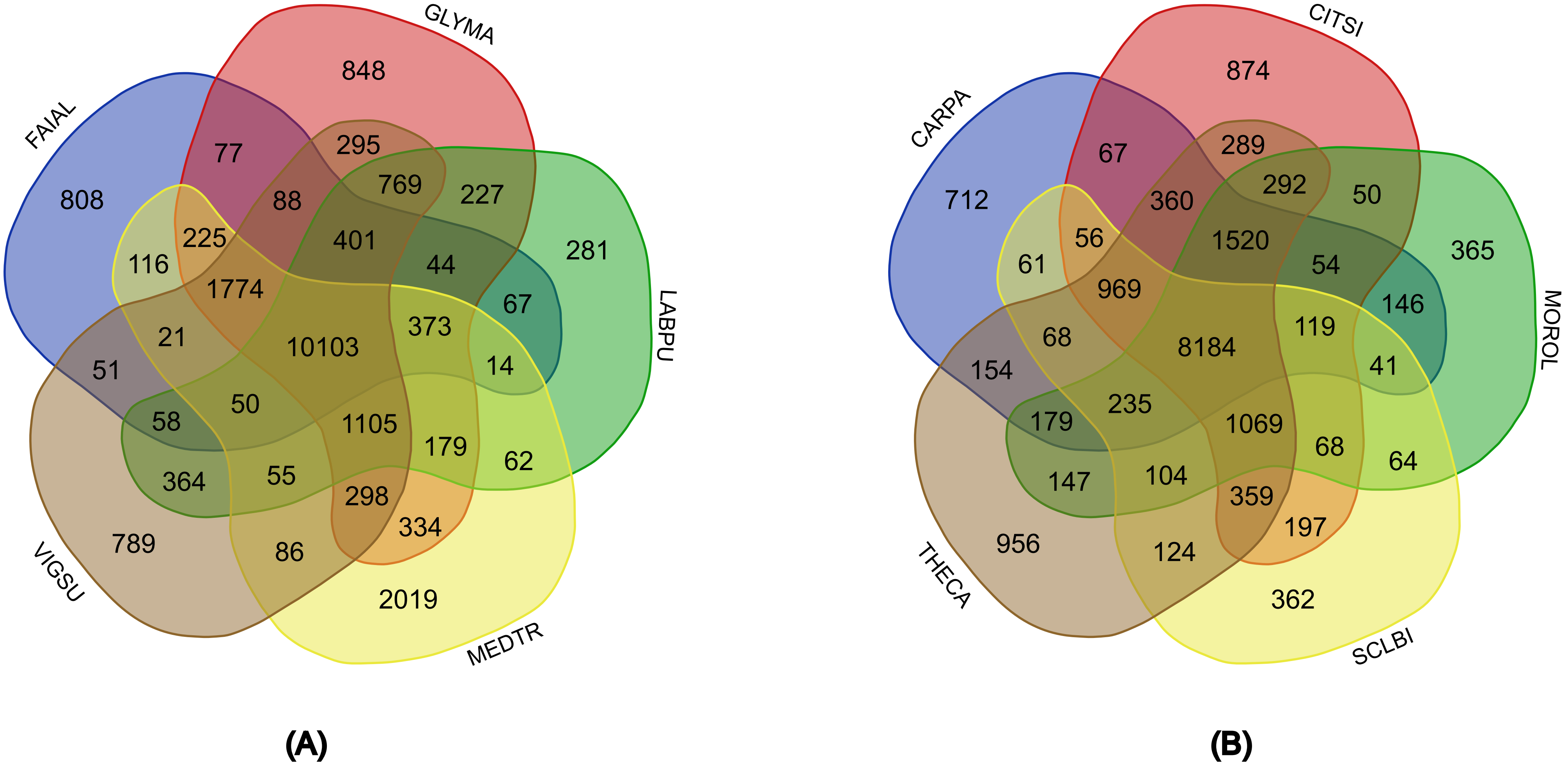

Figure 3

Click here to access/download;Figure;Figure3.pdf

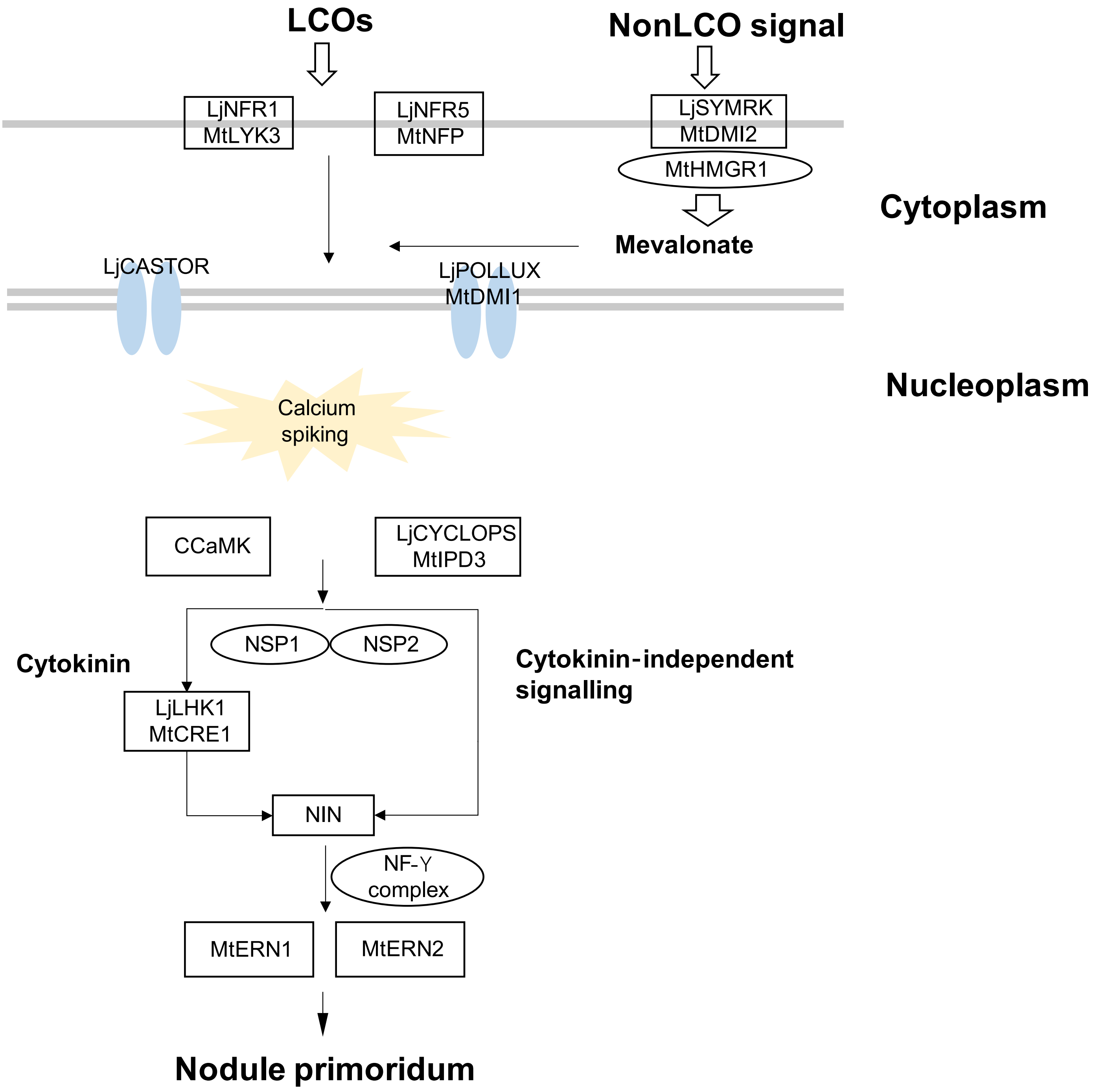

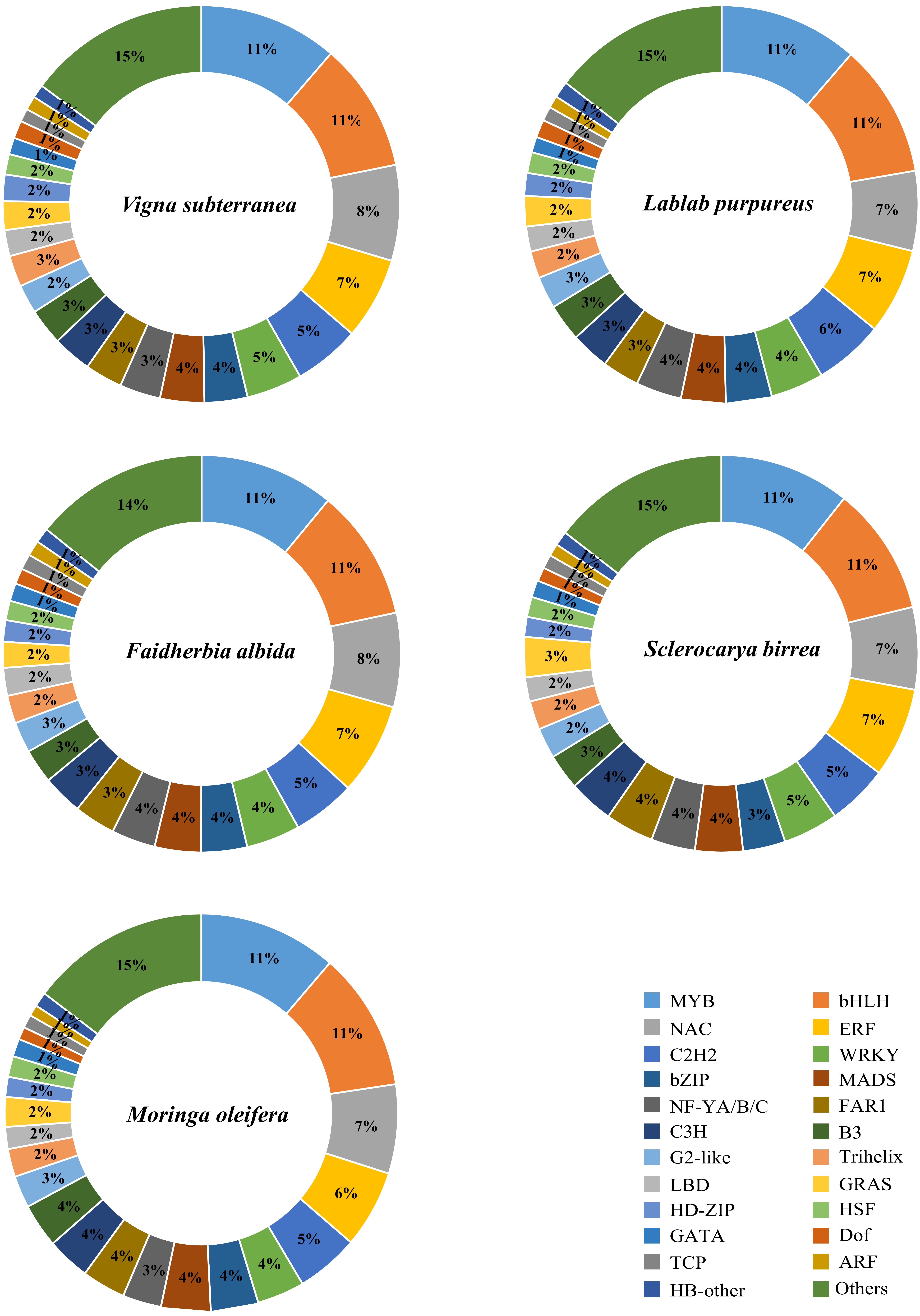
Vigna subterranea
Lablab purpureus
Faidherbia albida
Sclerocarya birrea
Moringa oleifera
MYB
bHLH
NAC
ERF
C2H2
WRKY
bZIP
MADS
NF-YA/B/C
FAR1
C3H
B3
G2-like
Trihelix
LBD
GRAS
HD-ZIP
HSF
GATA
Dof
TCP
ARF
HB-other
Others

Figure 5

Click here to access/download;Figure;Figure5.pdf

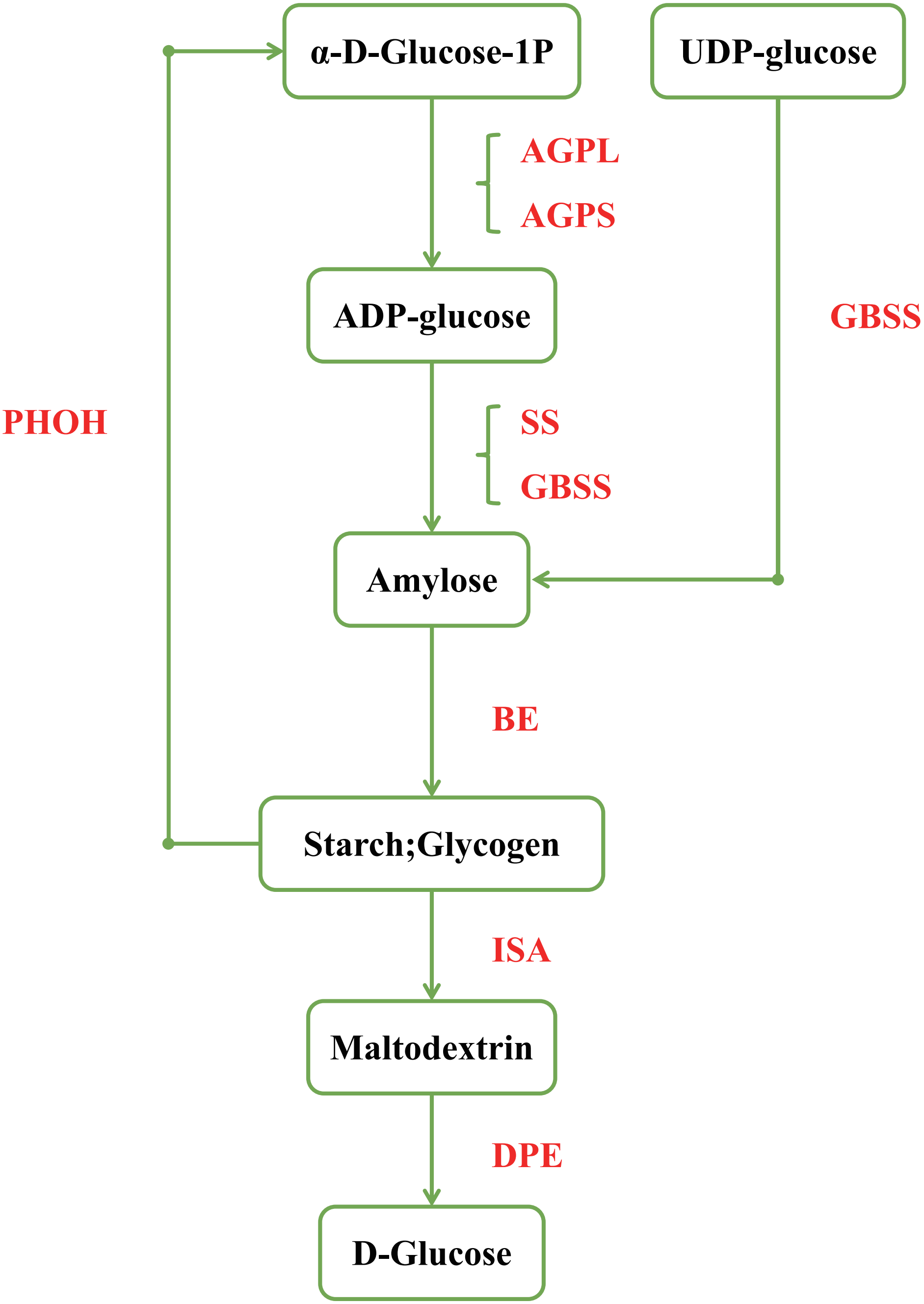

Click here to access/download
Supplementary Material
Additional file 1.docx

Click here to access/download
Supplementary Material
Additional file 1_clean.docx

Supplementary Material 2

Click here to access/download
Supplementary Material
Additional file 2.xlsx

Personal Cover
Click here to access/download;Personal Cover;Response_to_Reviewer_comments.docx

Dear Dr. Scott,

Sub: **Submission of the revised manuscript GIGA-D-18-00275**

We are glad to submit the thoroughly revised version of our manuscript entitled "The draft genomes of five agriculturally important African orphan crops", for possible publication in GigaScience as "Data Note".

The comments of the reviewers were highly insightful and enabled us to greatly improve the quality of our manuscript. The following were the major revision made in the manuscript:

1. The figure 1 in the earlier version of the manuscript was only a hand-drawn tree and was used to display the taxonomy of our sequenced species. The taxonomic position of *Populus trichocarpa* was according to the NCBI taxonomy. The actual phylogenetic tree based on 141-gene was constructed without *Populus trichocarpa* (Figure 3 & 4). Therefore, to avoid the confusion between different phylogenetic trees in the manuscript, we have merged the previous figure 1 and 3 into one, and moved figure 4 to the additional file1.
2. For the analyses related to gene families, we enriched the unique paralogs genes in KEGG pathway and GO analysis (Table S6 and 7).
3. We made an additional comparison of our *Vigna* genome data with two other sequenced genomes of *Vigna* species.
4. We re-classified the Repeat Type in Table 4 for better understanding.
5. We have revised the additional file 1: tableS1 and tableS2, and we used "bp" instead of "Gb", and also added "Reads number (bp)" data.

In the following pages, we present the point-by-point responses to each of the comments and suggestions of the reviewer. Revision 2 in the text is shown using the track changes. We strongly believe that these revisions in the manuscript and our accompanying responses are sufficient to make our manuscript suitable for publication in GigaScience.

We look forward to hearing from you at your earliest convenience.

Yours sincerely,

Xin Liu

Responses to comments of Reviewer #1

The topic of nitrogen fixation is complex and well studied. The brief section in this paper begins to ask some good question (about presence of genes that play important roles in nodulation) - but the presentation is insufficient to conclude "The reason why F. albida showed a relatively lower ability to fix nitrogen [77] could be explained by the loss of IPD3, NFP, and some proteins with lower efficiency which would have taken its place in F. albida." See the recent papers by Greismann et al., 10.1126/science.aat1743 and van Velzen et al., https://doi.org/10.1073/pnas.1721395115, for state-of-the-art work in this area.

Response: Thank you for the suggestion. The suggested reference manuscript on the "Phylogenomics studies of nitrogen-fixing root nodule symbiosis" which is recently published in Science (Greismann et al.) is the outcome of our BGI-Research team along with our collaborators. We do referred the suggested papers, and removed the confused conclusion, and revised the description, as follows:

"The difference in the components within RNS pathway (Table 8) together with the relatively weak nitrogen-fixing ability [80] of *F. albida* thus make itself a good reference in the research of RNS diversification".

1. Abstract: In the first sentence, the initial article, "A", is unnecessary ("A continued growth ...").

Response: According to your suggestion, we have revised the sentence, as follows:

“Continuous growth in the world population is expected to double the worldwide demand for food by 2050.”

2. Abstract, third sentence: typically, a sentence isn't started with a number ("30 species").

Response: According to your suggestion, we have revised the sentence, as follows:
“About 95% of the present food energy needs of humans are fulfilled by 30 species, within which wheat, maize and rice provide the majority of calories.”

3. Introduction: a minor point, but I am skeptical that the "World Population Prospects" from the U.N. (reference 1) is suitably paraphrased this way: "ensuring a sustainable food supply to meet the energy and nutritional needs of the expanding population is the greatest global challenge ahead of us." That is: scanning the report, I don't see that the report makes a claim about the "greatest global challenge" in an absolute sense (putting this need among others such as climate change, international conflict, etc.).

Response: Thank you for raising this question. According to your suggestion, we have revised the sentence, as follows:
“The world’s population is expected to reach 9.8 billion by 2050, thus ensuring a sustainable food supply to meet the energy and nutritional needs of the expanding population is one of the greatest global challenge.”

4. Introduction: "the utilization of crops plants appear to be the best choice" -- There is no other choice, right? We predominantly use crop plants (the only others being wild-harvested, non-crop foods).

Response: Thank you for the suggestion. According to your suggestion, we have revised the sentence, as follows:
“the utilization of potential crops (both model and non-model) plants appears to be a

better choice.”

5. "which originated in West Africa, and cultivated in Sub-Saharan" --> "which originated in West Africa, and IS cultivated in Sub-Saharan" (for parallel construction)

Response: According to your suggestion, we have revised the sentence, as follows: “which originated in West Africa, and is cultivated in Sub-Saharan areas, particularly Nigeria.”

6. "thereby highly making bambara groundnut a complete food" -- nonstandard word usage (omit "highly" to make it standard).

Response: According to your suggestion, we have omited “highly”, as follows: “thereby making bambara groundnut a complete food.”

7. Section on Lablab: "South West" should be one word, and should probably lower-case unless it names a particular place, e.g. "the Southwest": "In southwestern parts of Bangladesh ..."

Response: According to your suggestion, we have revised the sentence, as follows: “In southwestern parts of Bangladesh, lablab is reported to have a total production area of approximately 48000 ha.”

8. Extra period: "Kenya, approx.. 10,000"

Response: The suggested correction was implemented as follows: “Kenya, approx. 10,000 ha”

9. Section on phylogenetic analysis: "divergence time between M. truncatula and legumes" -- what other legumes? (since Medicago is itself a legume)

Response: The suggested correction was implemented as follows:

"39-59 Mya between *M. truncatula* and the main branch of legumes, 15-30 Mya between *G. max* and *P. vulgaris*, and 83-90 Mya between *T. cacao* and *A. thaliana*."

10. "In the present study, the divergence time between F. albida and Papilionoideae was predicted to be 79.1" - This is way outside the expected ranges, because the legume family itself is estimated to have originated around 60-64 Mya. Also, the value would depend on the particular species selected within the Papilionoideae - because rates are species-specific. See rates in Lavin et al. (2005), DOI: 10.1080/10635150590947131

Response: Thank you for raising this important point. We have removed the confused description, as follows:

"Based on the tree constructed by single-copy-family genes, the divergence time between *F. albida* and Papilionoideae was predicted to be 79.1 (70.0-87.0) Mya, which is a little different from the previous predicted origin of legumes based on two gene markers (matk and rbcL) (Lavin et al., 2005)."

11. Section "Identification of protein, starch, and fatty acid biosynthesis related genes" "Based on these observations we inferred that the ability to synthesize lecithin in V. subterranea is higher than that of soybeans" -- biosynthetic ability can't be inferred solely by the presence of gene sequences. All that can be said is that a necessary factor is present.

Response: Thank you for the suggestion. We do agree with your point, and removed the hypothetical description, and revised the sentence as follows:

"Based on these observations we inferred that the all the necessary factor to synthesize lecithin are present in *V. subterranea*."

12. "... and in comparison with other orphan crops it has higher potential to be a new

food crop." -- on what basis? Certainly not on the basis of gene composition, or on the ability to synthesize lecithin (which is itself of questionable nutritional value).

Response: Thank you for the suggestion. We do agree with your point, and removed the hypothetical description.

13. Sentence beginning "Therefore, this fine reference genomes together" needs to be rewritten. I don't think that "fine" is the intended word.

Response: Thank you for the suggestion. We have deleted this sentence.

14. Section "Identification of root nodule symbiosis pathway": "it has a major impact" --> "they have a major impact"

Response: According to your suggestion, we have revised the sentence, as follows: “They have a major impact on global nitrogen cycle.”

15. Data availability: I see that PRJNA453822 points to Faidherbia (good), but I don't find PRJNA474418 in GenBank. Should the bioproject IDs be given for the other species in the study?

Response: Thank you for pointing this out. Actually, we have now released the data (PRJNA474418) in NCBI.

16. Data availability: "The assembly and annotation of the B. ceiba genome and other supporting data, including BUSCO results, are available in the GigaScience database" -- is this an error? I assume this refers to Bombax ceiba - which is not described in the paper.

Response: Thank you for pointing out the typing error. According to your suggestion,

we have

revised the sentence, as follows:

"The assembly and annotation of the five genomes and other supporting data, including BUSCO results, are available in the GigaScience GigaDB repository."

Responses to comments of Reviewer #2

1. The premises of the study talks about orphan crops which are important for Africa: to qualify this statement, the crops chosen should be either consumed or grown by Africans in large quantity: Based on the introduction and the statistics given therein M. oleifera and L. purpureus do not qualify.

Response: The improper description is replaced with "underutilized local plants". For example, in the abstract" ..enhance agricultural productivity and tackle malnutrition in these countries, a greater utilization of neglected or underutilized local plants (generally so-called orphan crops, but also a few plants with special contribution to agriculture, such agroforestry and nutrient) could be a partial solution".

2. M. oleifera genome is already sequenced and published (Tian et al., 2015; Sci China Life Sci. 2015 Jul; 58(7):627-38. doi: 10.1007/s11427-015-4872-x.). The manuscript neither mentions this fact nor compares their results with this.

Response: Thank you for the suggestion. We add the description in Page 5, L16-18, as follows:

"Prior to this study, a draft genome of *Moringa oleifera* from Yunnan (China) was also reported with similar genome assembly size and gene numbers compared to our version".

3. The results of RNA-seq have been used only for checking the genome completion suggesting gross underutilization of data. The materials and methods says just different

parts of the plant has been subjected to RNA-seq. RNA-seq data of S. birrea is completely missing and there is no explanation of the same in the manuscript. The information provided in the supplementary file shows that there is no common denominator followed for the choice of tissue for RNA-seq. Further from table 5, it could be seen that only one among these various tissues have been used for checking the completeness of the WGS assembly. Overall, this gives a very hazy picture though a lot of work has been done and huge data-sets have been generated. I would recommend culling the data which is in no way utilized for obtaining the results provided in this manuscript.

Response: Thank you for raising this important point. We have actually compiled all the transcriptome data from different tissues, and used the combined version to check the completeness of the WGS assembly again. The results are shown in the Table 3 (not Table 5).

4. Genome and RNA-seq statistics are given only in Gb and Mb. This should be accompanied by number of reads and nucleotides.

Response: Thank you for the suggestion. According to your suggestion, we have revised the additional file 1: tableS1 and tableS2, and we used "bp" instead of "Gb", and also added "Reads number (bp)" data.

5. The difference between raw data and clean data seem to be too high ((30 to 43 %) except for S. birrea with respect to WGS data. Any specific reasons? This is even after keeping the cut off for quality score pretty low (< 16). Even for Sanger this kept as 20 while for NGS, this score is 30 to have high quality data.

Response: Thank you for pointing this out. Actually, the difference between raw and clean data is caused due to the filtering of the duplicated reads from the mate-pair libraries. However, for the pair-end data, the clean rate percentage were more than 80%.

Therefore, we strongly believe that the cut off (<16) is suitable and reliable for our data.

Kindly refer the below table for your kind perusal.

Species	Library insert size (bp)	Raw base num (bp)	Duplicated filter (%)	Clean base num (bp)	**Clean rate (%)**	Filter rate (%)
V. subterranea	250	49,086,002,100	4.85	42,847,600,630	**87.29**	12.71
	500	12,031,550,800	4.26	10,277,685,010	**85.42**	14.58
	2000	24,173,576,400	10.44	10,453,947,630	**43.25**	56.75
	6000	28,009,055,200	39.04	10,980,195,000	**39.20**	60.80
	20000	24,204,456,300	55.01	7,454,810,440	**30.80**	69.20
	Total	137,504,640,800		82,014,238,710	**59.64**	40.36
L. purpureus	250	60,287,284,200	12.31	42,480,817,860	**70.46**	29.54
	500	16,485,233,200	6.33	13,338,245,290	**80.91**	19.09
	2000	16,558,154,200	19.92	7,821,848,560	**47.24**	52.76
	6000	29,323,124,600	27.26	11,841,757,220	**40.38**	59.62
	10000	19,274,553,172	64.92	1,980,366,256	**10.27**	89.73
	Total	141,928,349,372		77,463,035,186	**54.58**	45.42
F. albida	170	33,121,301,800	4.67	28,703,837,700	**86.66**	13.34
	250	54,564,056,100	4.66	45,860,616,240	**84.05**	15.95
	350	26,516,538,200	3.97	23,136,560,300	**87.25**	12.75
	500	37,470,276,400	10.23	29,615,711,160	**79.04**	20.96
	800	26,368,901,400	13.01	20,376,553,800	**77.27**	22.73
	2000	38,010,750,800	42.20	13,971,852,350	**36.76**	63.24
	10000	21,485,522,000	69.52	2,813,237,470	**13.09**	86.91
	Total	237,537,346,700		164,478,369,020	**69.24**	30.76
M. oleifera	250	57,094,362,000	9.81	43,801,961,630	**76.72**	23.28
	500	47,503,842,900	9.28	36,852,547,210	**77.58**	22.42
	2000	59,470,238,400	25.04	24,641,039,770	**41.43**	58.57
	10000	27,070,800,800	59.47	5,461,131,620	**20.17**	79.83
	Total	191,139,244,100		110,756,680,230	**57.95**	42.05
S. birrea	170	33,105,136,400	11.94	26,550,586,320	**80.20**	19.80
	250	52,779,049,800	6.79	43,837,202,720	**83.06**	16.94
	350	40,081,173,400	25.71	32,814,989,680	**81.87**	18.13
	500	32,177,818,400	7.69	26,521,782,700	**82.42**	17.58
	800	28,800,733,400	7.06	23,567,139,060	**81.83**	18.17
	2000	33,418,800,000	53.19	8,543,561,280	**25.57**	74.43
	10000	22,862,981,800	57.16	5,613,268,420	**24.55**	75.45
	Total	243,225,693,200		167,448,530,180	**68.84**	31.16

6. The comparison of orthologs within the five species does not seem to have a common

ground as they belong to different species with not much evolutionary relationships to call for orthologous comparison. It would have been worthwhile to have the orthologous comparison with the related species. The choice of species in Table 5 needs to be explained.

Response: Thank you for the nice suggestion. We made the changes according to your suggestion. The orthologs of all the 14 species were identified just to get the single-copy-family genes for the construction of the tree. The comparison was made within fabids (for *F. albida*, *L. purpureus* and *V. subterranea*) and malvids (for *M. oleifera* and *S. birre*a) respectively. The species details in the Table 5 is now updated according to Figure 2.

7. In continuation of the previous point, the Vigna mungo genome and V. anguicularis genome should have been used along with other more complete legume genome (species) and mentioned in the manuscript while discussing the V subterranea.

Response: Thank you for the suggestion. We have now added the description in Page 5 L3-L4, as follows:

"The genomes of mung bean and adzuki bean have been published [9, 10], which also belongs to the *Vigna* genus"

8. The introduction does not talk about the previous genomic resources available in these five crops.

Response: Thank you for the suggestion. We admit our negligence. We have now added the relevant description regarding the previous genomic resources in the introduction section as well as in the data description, wherever necessary.

9. Table 4 formatting is confusing. Is it really required?

Response: Yes, the information on different classes of repeats (%) in five species is

important. According to your suggestion, we have revised the table 4 for more better understanding. We have now classified the Repeat Type in a more detailed manner (Table 4)

10. A lot of analysis has been mentioned in Supplementary data - however there is no major point emerging out of it - such data may be removed from the manuscript altogether. It just increases the bulk of the paper without really contributing anything.

Response: Thank you for the suggestion. We have removed the previous

table S13. Comparative analysis of the protein biosynthesis related genes in each species., table S14. Comparative analysis of the starch biosynthesis related genes in each species.

table S15. Comparative analysis of the fatty acid-plastids biosynthesis related genes in each species.

table S16. Comparative analysis of the fatty acid synthesis and storage related genes in each species.,

table S17. Comparative analysis of the fatty acid degradation related genes in each species. in additional file 2.

And add new table in additional file 1, as follows:

Table S6. Enriched pathways of unique paralogs genes in families.

Table S7. Enriched GO terms (level 3) of unique paralogs genes in families.

What's more ,we renumber the table.

11. Overall, results and discussion section shows hardly any discussion and incomplete results

Response: As our manuscript is a "data note" we focused mainly on data and its analysis part. The detailed findings and discussion will be presented in our subsequent manuscript covering the genomic data of several orphan crop species. The overall goal of the African Orphan Crops Consortium (AOCC) and BGI is to sequence, assemble and annotate the genomes of 101 plants contributed to traditional African food supplies

by 2020 (www.africanorphancrops.org).

Minor shortcomings

1. Please read the manuscript carefully and check punctuation. Examples: Page 20: Line No: In other cereals .... in barley.

Page 22: LN: 48-50. Fragment owing to wrong punctuation.

2. The accession numbers of these data-sets are indicated as SSR in the respective supplementary tables.

Response: We have now rectified the above mentioned errors.

Responses to comments of Reviewer #3

1. The plants sequenced in this project have smaller genome size compared to many other sequenced crops, and repeat elements are also comparatively low. However none of the assemblies are complete and couldn’t assemble into the chromosome level. If the authors have used long insert libraries also, it would have been better

Response: Thank you for the suggestion, we do agree with your comments. The incomplete assembly could be due to large fragments of repetitive sequences. This is one of the reasons, why we have submitted the manuscript as “data note” rather than “full length article”. The experience gained from the sequencing of five orphan species, we plan to apply more sequencing strategies for the future African orphan project, like techniques generating longer reads.

2. “Various gene structure parameters were compared to the related species of each sequenced genome as summarized in table 5”- The number of protein coding genes in these sequenced genomes seems to be less compared to the related species. Can the authors provide an explanation for this?

Response: Thank you for the suggestion. The number of protein coding genes in *V. subterranean* and *F. albida* is similar to other legumes, except *G. max* and *M. truncatula*. These exceptionally large number is caused by their lineage-specific duplication. The lower numbers in other three species may be related to their smaller genome size. But, our BUSCO results showed a relative high completeness of core genes, compared to those of other published plant genomes, and the size of the assemblies is closer to the estimated sizes. For instance, the previously reported gene number in *M. oleifera* (Tian et al., 2015; Sci China Life Sci. 2015) is extremely close to our number. Therefore, the possibility of mis-annotation of genes is pretty low.

3. Figure S5 is not provided

Response: Thank you for the suggestion. It is provided but our previous layout was confusing. Thank you for reminding, and we have modified it in this version.

4. 633, 372, 861, 364 and 216 genes are unannotated in *V. subterranea L. purpureus F. albida S. birrea* and *M. oleifera* respectively. Are these genes specific to the respective genomes?

Response: We found that there are 400, 305, 1514, 293, 172 unannotated genes which does not cluster with other species in gene family of *V. subterranea L. purpureus F. albida S. birrea* and *M. oleifera* respectively. Hence, we speculated that these genes are specific to the respective genomes. Kindly refer the specific results in the below table.

Species	***V. subterranea***	***L. purpureus***	***F. albida***	***S. birrea***	***M. oleifera***
Unannotated gene number	633	372	1861	364	216
Unique family gene number	3118	538	1966	796	798

Overlap gene number	147	31	312	67	40
Unoverlap gene number	486	341	1549	297	176
Unoverlap gene cluster with other species	86	36	35	4	4
Unoverlap gene uncluster with other species	400	305	1514	293	172

5. "Furthermore, the 10,103 gene families of *V. subterranea*, *L. purpureus*, *F. albida*, *M. truncat*ula and G. max were clustered (Figure 2A). There were 1,105 orthologous families shared by the four Papilionoideae species, while 808 gene families containing 1,966 genes were specific to F. albida, 281 gene families containing 538 genes were specific to L. purpureus, 789 gene families containing 3,118 genes were specific to V. subterranea.

Moreover, 8,184 gene families of S. birrea, M. oleifera, C. papaya, C. sinensis and T. cacao were clustered (Figure 2B), of which 365 gene families containing 798 genes were specific to M. oleifera, 362 gene families containing 796 genes were specific to S. birrea, respectively". -To which class the specific genes mostly belong in the functional annotation?

Response: Thank you for raising the question. We additionally analyzed our data and updated the description as follows:

"The enrichment analysis on KEGG pathway of the paralogs genes were also calculated (Additional file1: Table S6, S7). The functional annotation revealed that they mainly correspond to the carbon fixation, zeatin biosynthesis, glyoxylate and dicarboxylate metabolism in *V. subterranea*. However, for *L. purpureus*, the fatty acid elongation pathway was enriched. While in *F. albida*, the pathways corresponding to the plant-pathogen interaction and cyanoamino acid metabolism were enriched. In *S. birrea*, the pathways of plant-pathogen interaction, starch and sucrose metabolism, fatty acid biosynthesis were enriched. In *M. oleifera*, the pathways related to fatty acid and diterpenoid biosynthesis, cyanoamino acid metabolism were enriched. The enrichment

analysis on GO of paralogs genes were ion binding, metabolic process, disease resistance, cell component, biological process in *V. subterranea*, *L. purpureus*, *F. albida*, *M. oleifera*, and *S. birrea* respectively."

6. In the phylogenetic analysis with 141 single-copy genes from 14 species, Populus trichocarpa clusters with other members in Fabids. But in some other phylogenetic analysis constructed using the same criteria, the group malpigiales, which includes Populus trichocarpa clusters with malvids or as a separate group. How do the authors explain this?

Response: Thank you for the nice suggestion. The figure 1 in the earlier version of manuscript was only a hand-drawn tree, and was used to display the taxonomy of our sequenced species. The taxonomic position of *Populus trichocarpa* was according to the NCBI taxonomy. The actual phylogenetic tree based on 141-gene was constructed without *Populus trichocarpa* (Figure 3 & 4). Therefore, to avoid the confusion between different phylogenetic trees in the manuscript, we have merged the previous figure 1 and 3, and moved figure 4 to the additional file1.

Chang et al 2018. Supporting data for "The draft genomes of five agriculturally important African orphan crops". GigaScience Database 2018. http://dx.doi.org/10.5524/100504.
