# Supplementary material for: The draft genomes of five agriculturally important African orphan crops
Source: Gigascience. 2018 Dec 7;8(3):giy152. doi: 10.1093/gigascience/giy152 (PMC6405277; doi:10.1093/gigascience/giy152)
Supplement: GIGA-D-18-00275_Revision_2.pdf [file gigascience_8_3_giy152_s8.pdf]

GigaScience

The draft genomes of five agriculturally important African orphan crops

--Manuscript Draft--

**Manuscript Number:**	GIGA-D-18-00275R2
**Full Title:**	The draft genomes of five agriculturally important African orphan crops
**Article Type:**	Data Note
**Funding Information:**	Shenzhen Municipal Government of China (JCYJ20150831201643396) — Dr. Yue Chang<br>Shenzhen Municipal Government of China (JCYJ20150529150409546) — Dr. Shifeng Cheng<br>Guangdong Provincial Key Laboratory of Genome Read and Write (2017B030301011) — Mr. Haorong Lu
**Abstract:**	Background: Continuous growth of the world population is expected to double the worldwide demand for food by 2050. Eighty-eight percent of countries current face a serious burden of malnutrition, especially in Africa and South and South-East Asia. About 95% of the food energy needs of humans are fulfilled by just 30 species, of which wheat, maize and rice provide the majority of calories. Therefore, to diversify and stabilize global food supply, enhance agricultural productivity and tackle malnutrition, greater use of neglected or underutilized local plants (so-called 'orphan crops', but also including a few plants of special significance to agriculture, agroforestry and nutrition) could be a partial solution.<br><br>Results: Here, we present draft genome information from five agriculturally, biologically, medicinally and economically important underutilized plants native to Africa; Vigna subterranea, Lablab purpureus, Faidherbia albida, Sclerocarya birrea, and Moringa oleifera. Assembled genomes range in size from 217 to 654 Mb. In V. subterranea, L. purpureus, F. albida, S. birrea and M. oleifera we have predicted 31707, 20946, 28979, 18937, 18451 protein-coding genes, respectively. By further analysing the expansion and contraction of selected gene families, we have characterized root nodule symbiosis genes, transcription factors and starch biosynthesis-related genes in these genomes.<br><br>Conclusions: These genome data will be useful to identify and characterize agronomically important genes and understand their modes of action, enabling genomics-based, evolutionary studies, and breeding strategies to design faster, more focused and predictable crop improvement programs.
**Corresponding Author:**	Xin Liu, Ph.D.<br>BGI<br>CHINA
**Corresponding Author Secondary Information:**	
**Corresponding Author's Institution:**	BGI
**Corresponding Author's Secondary Institution:**	
**First Author:**	Yue Chang
**First Author Secondary Information:**	
**Order of Authors:**	Yue Chang
	Huan Liu
	Min Liu
	Xuezhu Liao
	Sunil Kumar Sahu

Powered by Editorial Manager® and ProduXion Manager® from Aries Systems Corporation

	Yuan Fu
	Bo Song
	Shifeng Cheng
	Robert Kariba
	Samuel Muthemba
	Prasad S. Hendre
	Sean Mayes
	Wai Kuan Ho
	Presidor Kendabie
	Sibo Wang
	Linzhou Li
	Alice Muchugi
	Ramni Jamnadass
	Haorong Lu
	Shufeng Peng
	Allen Van Deynze
	Anthony Simons
	Howard Yana-Shapiro
	Xun Xu
	Huanming Yang
	Jian Wang
	Xin Liu, Ph.D.
**Order of Authors Secondary Information:**	
**Response to Reviewers:**	Dear Dr. Scott,<br><br>We are glad to resubmit the thoroughly revised and cleaned version of our manuscript entitled “The draft genomes of five agriculturally important African orphan crops”, for possible publication in GigaScience as “Data Note”.<br>The corrections made and suggested by Dr. Lisa Martin were highly useful to significantly improve the quality of our manuscript. We have carefully implemented all the corrections suggested by her. Now we strongly believe that the revised manuscript is now ready for publication in GigaScience.<br><br>We look forward to hearing from you at your earliest convenience.<br>Yours sincerely,<br>Xin Liu<br><br>Reviewer comments:<br>Reviewer #1: The revisions in the last round satisfactorily addressed my concerns.<br>Reviewer #2: The revised version is well written and can be accepted for publication.<br>Response: We thank all the reviewers for their valuable suggestions in the earlier version of the manuscript, and for giving their kind acceptance to our manuscript.
**Additional Information:**	
**Question**	**Response**
Are you submitting this manuscript to a special series or article collection?	No

Powered by Editorial Manager® and ProduXion Manager® from Aries Systems Corporation

**Experimental design and statistics**<br><br>Full details of the experimental design and statistical methods used should be given in the Methods section, as detailed in our Minimum Standards Reporting Checklist. Information essential to interpreting the data presented should be made available in the figure legends.<br><br>Have you included all the information requested in your manuscript?	Yes
**Resources**<br><br>A description of all resources used, including antibodies, cell lines, animals and software tools, with enough information to allow them to be uniquely identified, should be included in the Methods section. Authors are strongly encouraged to cite Research Resource Identifiers (RRIDs) for antibodies, model organisms and tools, where possible.<br><br>Have you included the information requested as detailed in our Minimum Standards Reporting Checklist?	Yes
**Availability of data and materials**<br><br>All datasets and code on which the conclusions of the paper rely must be either included in your submission or deposited in publicly available repositories (where available and ethically appropriate), referencing such data using a unique identifier in the references and in the "Availability of Data and Materials" section of your manuscript.<br><br>Have you have met the above requirement as detailed in our Minimum Standards Reporting Checklist?	Yes

Powered by Editorial Manager® and ProduXion Manager® from Aries Systems Corporation

Manuscript
Click here to access/download;Manuscript;GIGA-D-18-00275_clean.docx
Click here to view linked References

The draft genomes of five agriculturally important African orphan crops

Yue Chang[1,2#], Huan Liu[1,2,8#], Min Liu[1,2,8#], Xuezhu Liao[1,2,8], Sunil Kumar Sahu[1,2,8], Yuan Fu[1,2], Bo Song[1,2], Shifeng Cheng[1,2], Robert Kariba[3], Samuel Muthemba[3], Prasad S. Hendre[3], Sean Mayes[5,6,7], Wai Kuan Ho[6,7], Presidor Kendabie[5], Sibo Wang[1,2], Linzhou Li[1,2], Alice Muchugi[3], Ramni Jamnadass[3], Haorong Lu[1,2], Shufeng Peng[1,2], Allen Van Deynze[3,4], Anthony Simons[3], Howard Yana-Shapiro[3,4], Xun Xu[1,2], Huanming Yang[1,2], Jian Wang[1,2] and Xin Liu[1,2,8,9*]

[1]BGI-Shenzhen, Beishan Industrial Zone, Yantian District, Shenzhen 518083, China; [2]China National GeneBank, BGI-Shenzhen, Jinsha Road, Shenzhen 518120, China; [3]African Orphan Crops Consortium, World Agroforestry Centre (ICRAF), Nairobi, Kenya; [4]University of California, 1 Shields Ave, Davis, CA 95616, USA; [5]Plant and Crop Sciences, Biosciences, University of Nottingham, Sutton Bonington Campus, Loughborough, Leicestershire LE12 5RD, UK; [6]Biosciences, University of Nottingham Malaysia Campus, Jalan Broga 43500 Semenyih, Selangor, Malaysia; [7]Crops For the Future, Jalan Broga, 43500 Semenyih, Selangor, Malaysia; [8]State Key Laboratory of Agricultural Genomics, BGI-Shenzhen, Shenzhen 518083, China; [9]BGI-Fuyang, BGI-Shenzhen, Fuyang 236009, China

*Correspondence address. Xin Liu, BGI-Shenzhen, Beishan Industrial Zone, Yantian District, Shenzhen 518083, China;

Tel: +86 18025460332; E-mail: liuxin@genomics.cn

ORCID:

Xin Liu: 0000-0003-3256-2940;

Yue Chang: 0000-0003-3909-0931;

Huan Liu: 0000-0002-6902-9931;

Liu Min: 0000-0002-8876-7534;

Sunil Kumar Sahu: 0000-0002-4742-9870

#Equal contribution

ABSTRACT

Background: Continuous growth of the world population is expected to double the worldwide demand for food by 2050. Eighty-eight percent of countries current face a serious burden of malnutrition, especially in Africa and South and South-East Asia. About 95% of the food energy needs of humans are fulfilled by just 30 species, of which wheat, maize and rice provide the majority of calories. Therefore, to diversify and stabilize global food supply, enhance agricultural productivity and tackle malnutrition, greater use of neglected or underutilized local plants (so-called 'orphan crops', but also including a few plants of special significance to agriculture, agroforestry and nutrition)

could be a partial solution.

Results: Here, we present draft genome information from five agriculturally, biologically, medicinally and economically important underutilized plants native to Africa; *Vigna subterranea*, *Lablab purpureus*, *Faidherbia albida*, *Sclerocarya birrea*, and *Moringa oleifera*. Assembled genomes range in size from 217 to 654 Mb. In *V. subterranea*, *L. purpureus*, *F. albida*, *S. birrea* and *M. oleifera* we have predicted 31707, 20946, 28979, 18937, 18451 protein-coding genes, respectively. By further analysing the expansion and contraction of selected gene families, we have characterized root nodule symbiosis genes, transcription factors and starch biosynthesis-related genes in these genomes.

Conclusions: These genome data will be useful to identify and characterize agronomically important genes and understand their modes of action, enabling genomics-based, evolutionary studies, and breeding strategies to design faster, more focused and predictable crop improvement programs.

Keywords: Orphan crops, food security, whole-genome sequencing, transcriptome, root nodule symbiosis, transcription factor.

Background

The world's population is expected to reach 9.8 billion people by 2050. Ensuring a sustainable food supply to meet the energy and nutritional needs of the expanding population is one of the greatest global challenges [1]. Approximately 88% of countries currently face a serious burden of malnutrition [2]. To overcome this burgeoning food

and nutritional challenge, the use of potential crop plants (both model and non-model) appears to be a better choice. Throughout history, humans have relied on an astonishing variety of plants for energy and nutrition: from 390,000 known plant species, around 5,000–7,000 plant species have been cultivated or collected for food [1, 2]. However, in the present century, fewer than 150 species are commercially cultivated for food purposes, and just 30 species provide 95% of human food energy needs. More than half of the protein and calories we obtain from plants are acquired from just three 'megacrops': rice, wheat and maize [3]. This narrow range of dietary diversity is partly a result of decades of intensive research, focused on just a few species, which has successfully led to the production of high-yielding varieties of these major crops, usually cultivated under high-input agricultural systems. However, in some regions, we are now witnessing a drastic decrease in their yields and the question has been raised as to whether rice and wheat (in particular) are currently making enough breeding progress to meet the challenge. All three megacrops are high-energy carbohydrate sources, but are limited in protein content. Even if these crops can meet the energy requirement of the increasing world population, they cannot meet the nutritional requirement for active health by themselves [2].

To diversify the global food supply, enhance agricultural productivity and tackle malnutrition, it is necessary to diversify and focus more on crop plants that are utilized in rural societies as a local source of nutrition and sustenance, but have so far received little attention for crop improvement. These landraces tend to be locally adapted, and can often provide a rich source of nutrition, yet they have largely been ignored by

modern interventions. The goal of the African Orphan Crops Consortium [4] (AOCC), an international public–private partnership, is to sequence, assemble and annotate the genomes of 101 plants that contribute to traditional African food supplies by 2020. These neglected or orphan plants have been seldom studied by scientists, but are of major importance in many African countries. They are usually grown by smallholder farmers, either for consumption or local sale, and are a major food source for 600 million rural Africans [5, 6]. In this study, we sequenced and assembled draft genomes of five African orphan plant species (Figure 1), which are highly important to augment food and nutritional security in Africa.

Vigna subterranea (Bambara groundnut; NCBI: txid115715) belonging to the Fabacaeae family, is a leguminoceous plant species that originated in West Africa, and is cultivated in sub-Saharan areas, particularly Nigeria [7, 8]. With good nitrogen-fixing ability and drought tolerance, on average the seeds contain 63% carbohydrate, 19% protein and 6.5% fat, thereby making bambara groundnut a complete food. Approximately 165,000 tons of this species is produced in Africa each year, but yields are low because efforts to improve Bambara have been neglected for many years [9]. The genomes of mung bean and adzuki bean, which also belong to the *Vigna* genus, have been published [10, 11].

Moringa oleifera (Moringa; NCBI: txid3735) is a highly nutritious, fast growing and drought-tolerant tree, which is indigenous to northern India, Pakistan and Nepal [12]. Presently, this species is ubiquitously distributed throughout tropical and subtropical countries, and in particular covers the major agro-ecological region in

Nigeria. The leaves are rich in protein, minerals, beta-carotene and antioxidant compounds, which are generally used as nutrition supplements and in traditional medicine. The seeds are used to extract oil, and seed powder can be used for water purification [13, 14]. There are varying reports of *Moringa* production: India is the largest producer of *Moringa* with an annual production of 1.1–1.3 million tonnes of tender fruits from an area of 38,000 ha. In Limpopo province, *Moringa* is cultivated in relatively small areas (0.25–1 ha), with seed yields of 50–100 kg/ha^{-1} [15]. Prior to this study, a draft genome of *Moringa oleifera* from Yunnan (China) was reported [16], which estimated a similar genome assembly size and gene numbers to our version.

Lablab purpureus (Dolichos bean or hyacinth bean; NCBI: txid35936), a member of the Fabaceae family, is one of the most ancient (>3500 years) domesticated and multipurpose legume species, which is used as an intercrop in livestock systems. Although it has large agromorphological diversity in South Asia, its origin appears to be African [17]. It is rich in protein, has good nitrogen-fixing ability, and is highly adaptable to diverse environmental conditions [18]. Limited production data are available, suggesting that yields are low. In south-western parts of Bangladesh, *Lablab* is reported to have a total production area of approximately 48,000 ha [17]. In other areas, it has a similarly relatively low production area; for example, Kenya, approx. 10,000 ha [19], and Karnataka, India, 79,000 ha [20].

Faidherbia albida (apple-ring acacia; NCBI: txid138055) is the only tree species in the *Faidherbia* genus (Fabaceae). Its distinctive key features, such as reverse phenology (leaves grow in the long dry season and shed during the rainy season) and

nitrogen-fixing ability, mean that *F. albida* has been planted as a key agroforestry species in traditional African farming systems for hundreds of years [21]. It originated in the Sahara or eastern and southern Africa, then spread across semi-arid tropical Africa, and later to the Middle East and Arabia. Estimates suggest that, during the last decade, the tree was cultivated over an area of 300,000 ha [22]. Average pod production ranges from 6–135 kg per tree per year in the Sudanian zone. In Mana Pools, Zimbabwe, two trees averaged 161 kg per tree in one year [23]. This yield per unit area is about 2,000–3,000kg/ha, assuming a density of ~20 mature trees per hectare [24].

Sclerocarya birrea (Marula; NCBI: txid289766) belongs to the Anacardiaceae family, and is a traditional fruit tree found in southern Africa – mostly south of the Zambezi river [25]. Fruits are eaten fresh, or are used to produce juices and wine, which has substantial socioeconomic and commercialization importance. The seeds of the fruits are rich in nutrition and oil content (56%), and are often consumed raw. It is estimated that the total value of the commercial marula trade is worth USD $160,000 per year to rural communities [26], with values per tree ranging from 315 kg (17,500 fruits) to 1,643 kg (91,300 fruits) [26, 27]. A survey in north-central Namibia showed that, on average, there are 5.33 farms per household, with a total of 13,278 fruiting trees.

Considering the limited systematic efforts to improve the breeding of these understudied tropical crops so far, making their genomic data available will provide much-needed impetus to conduct basic and applied translational research to improve and develop them as important, sustainably cultivated food crops. These efforts will be vital for directly or indirectly improving nutrition for the increasing urban populations

in the regions where these crops are grown.

Data description

Sample collection, library construction, and sequencing

Genomic DNA was extracted either from a tree (*F. albida*, *M. oleifera*) or from nursery plantlets (*V. subtarranea*, *L. purpureus*, *S. birrea*) grown at the World AgroForestry Center campus in Kenya using a modified CTAB method [28].

Extracted DNA was used to construct paired-end libraries (insert size ranging from 170–800 bp) and mate-pair libraries (insert size >2 kb) following Illumina (San Diego, USA) protocols. Subsequently, sequencing was performed on a HiSeq 2000 platform (Illumina, San Diego, CA, USA) using a shotgun sequencing strategy to generate more than 100 Gb raw data for each species (see Additional file 1: Table S1). Data were filtered using SOAPfilter (v2.2) [29] as follows: (1) small insert size reads were discarded; (2) PCR duplicates and adapter contamination were discarded; (3) reads with ≥30% low quality bases (quality score ≤15) were removed; (4) bases with low quality were trimmed from each end of the reads; (5) reads with ≥10% uncalled ("N") bases were removed. At the end, more than 100× high-quality reads were obtained for each species, according to their estimated genome size (see Additional file 1: Table S1).

RNA for transcriptome sequencing was extracted from different tissues of *V. subterranea*, *L. purpureus*, *F. albida*, and *M. oleifera*. The RNA was extracted using the

PureLink RNA Mini Kit (Thermo Fisher Scientific, Carlsbad, CA, USA) according to the manufacturer's instructions. For each sample, RNA libraries were constructed by following the TruSeq RNA Sample Preparation Kit (Illumina, San Diego, CA, USA) manual, and were then sequenced on the Illumina HiSeq 2500 platform (paired-end, 100-bp reads), generating ~36 Gb of sequence data for each species. Data were then filtered using a similar method to that used in DNA filtration, with a slight modification: (1) reads with ≥10% low quality bases (quality score ≤15) were removed; and (2) reads with ≥5% uncalled ("N") bases were removed (see Additional file 1: Table S2). All the transcriptome data from different tissues were compiled, and the combined version was used to check the completeness of the whole genome sequence assembly.

Evaluation of genome size

Clean reads of the paired-end libraries were used to estimate genome sizes (insert size 250 bp and 500 bp). k-mer frequency distribution analysis was performed using the following formula:

$$Gen = Num*(Len - 17 + 1) / K\_Dep$$

Where: *Num* represents the read number of reads used. *Len* represents the read length, *K* represents the k-mer length, and *K_Dep* refers to where the main peak is located in the distribution curve [30].

k-mer distributions of *F. albida, S. birrea,* and *M. oleifera* showed two distinct peaks (see Additional file 1: Figure S1), where the second peak was confirmed as the main one for each of the species. The genome sizes of *V. subterranea, L. purpureus, F.*

albida, *S. birrea* and *M. oleifera* were predicted as 550, 423, 661, 356 and 278 Mb, respectively (see Additional file 1: Table S3).

De novo genome assembly

For *de novo* genome assembly, SOAPdenovo2 (SOAPdenovo2, RRID:SCR_014986) [29] was used for constructing contigs, followed by scaffolding, and finally gap filling. To build contigs, libraries ranging from 170–800 bp were used to construct de Bruijn graphs with the parameters "pregraph –d 2 –K 55", and contigs were subsequently formed with the parameters "contig –g –D 1" to delete links with low coverage. In the scaffolding step, paired-end and mate-pair information was used to order the contigs with parameters "scaff –g –F" and "map –g –k 55". Finally, to fill the gaps within scaffolds, GapCloser version 1.12 (GapCloser, RRID:SCR_015026) [29] was used with the parameters "–l 150 –t 32" using the pair-end libraries. Finally, total assembled lengths of 535.05, 395.47, 653.73, 330.98, and 216.76 Mb were obtained for *V. subterranea*, *L. purpureus*, *F. albida*, *S. birrea* and *M. oleifera* genomes, respectively (Table 1). This accounted for approximately 97.3%, 93.5%, 98.9%, 92.9% and 77.9% of their respective estimated genome sizes.

Genome evaluation

Genome assembly completeness was assessed with BUSCO (Benchmarking Universal Single-Copy Orthologs) version 3.0.1, (BUSCO, RRID:SCR_015008) [31]. From the 1,440 core embryophyta genes, 1,326 (92.1%), 1,341 (93.2%), 1,315 (91.3%), 1,384

(96.1%) and 1,297 (90.1%) were identified in the *V. subterranea*, *L. purpureus*, *F. albida*, *S. birrea* and *M. oleifera* assemblies, respectively, with 1,244 (86.4%), 1,258 (87.4%), 1,231 (85.5%), 1,352 (93.9%) and 1.278 (88.8%) genes, respectively, being complete (Table 2).

To evaluate the completeness of genes in the assemblies, unigenes were generated from the transcript data of each species using Bridger software with the parameters "–kmer_length 25 –min_kmer_coverage 2" [32], and then aligned to the corresponding assembly using BLAT (BLAT, RRID:SCR_011919) [33]. The results indicated that each of the assemblies covered about 90% of the expressed unigenes, suggesting that the assembled genomes contained a high percentage of expressed genes (Table 3).

To confirm the accuracy of the assemblies, some of the paired-end libraries were mapped to the genome assemblies, and the sequencing coverage was calculated using SOAPaligner, version 2.21 (SOAPaligner/soap2 , RRID:SCR_005503) [34]. Sequencing coverage showed that >99% of the bases had a sequencing depth of more than 10×, and confirmed the accuracy at the base level (see Additional file 1: Figure S2). GC content and average depth were also calculated with 10 kb non-overlapping windows. The distribution of GC content indicated a relatively pure single genome without contamination or GC bias (see Additional file 1: Figure S3). The GC content of each sequenced genome was also compared with that of a related species. As expected, close peak positions showed that the related species were similar in GC content (see Additional file 1: Figure S4).

Repeat annotation

Repetitive sequences were identified using RepeatMasker (version 4-0-5) [35], with a combined Repbase and a custom library obtained through careful self-training. The custom library comprised three parts: MITEs (miniature inverted repeat transposable elements), LTRs (long terminal repeats), and an extensive library that was constructed as follows. First, the annotated MITE library was created using MITE-hunter [36] with default parameters. Then, a library of LTR elements with lengths of 1.5–25 kb, and two libraries of terminal repeats ranging from 100–6000 bp with ≥85% similarity were constructed using LTRharvest [37] integrated in Genometools (version 1.5.8) [38] with parameters "–minlenltr 100, –maxlenltr 6000, –mindistltr 1500, –maxdistltr 25000, –mintsd 5, –maxtsd 5, –similar 90, –vic 10". Subsequently, we used several strategies to filter the candidates, i.e. 1) presence of intact poly purine tracts or primer binding sites [39] using the eukaryotic tRNA library [40]; 2) removal of contamination from local gene clusters and tandem local repeats by inspecting 50 bases of the upstream and downstream LTR flanks using MUSCLE (MUSCLE, RRID:SCR_011812) [41] for a minimum of 60% identity; and 3) removal of nested LTR candidates from other types of the elements. Exemplars for the LTR library were extracted from the filtered candidates using a cutoff of 80% identity in 90% of the sequence. Regions of the genome annotated as LTRs and MITEs were masked, and then put into RepeatModeler (version 1-0-8; RepeatModeler, RRID:SCR_015027) to predict other repetitive sequences for the extensive library. Finally, the MITE, LTR and extensive libraries were integrated into the custom library, which was combined with the Repbase library and

taken as an input for RepeatMasker to identify and classify genome-wide repetitive elements. The pipeline identified 205,189,285 (38.35% of the genome length), 147,050,327 (37.18%), 358,653,534 (54.86%), 149,551,125 (45.18%), and 87,944,150 (40.57%) bases of non-redundant repetitive sequences in *V. subterranea*, *L. purpureus*, *F. albida*, *S. birrea* and *M. oleifera*, respectively. LTR elements were predominant, taking up 19.8%, 23.8%, 44.6%, 38.8%, 22.7% of each genome, respectively (Table 4).

Gene prediction

Repetitive regions of the genome were masked before gene prediction. Structures of protein-coding genes were predicted using the MAKER-P pipeline (version 2.31) [42] based on RNA, homologous and *de novo* prediction evidence. For RNA evidence, the clean transcriptome reads were assembled into inchworms using Trinity (version 2.0.6) [43], and then provided to MAKER-P as expressed sequence tag evidence. For homologous comparison, protein sequences from the model plant *Arabidopsis thaliana*, and related species of each sequenced species, were downloaded and provided as protein evidence. Related species used for homologous evidence were *Arachis duranensis*, *A. ipaensis*, *Glycine max*, *Lotus japonicus*, *Medicago truncatula*, and *Vigna angularis* for *V. subterranea*; *A. duranensis*, *Cajanus cajan*, *G. max*, *M. truncatula*, *Phaseolus vulgaris*, and *V. angularis* for *L. purpureus*; *C. cajan*, *V. angularis*, *L. japonicus*, *P. vulgaris*, *M. truncatula*, and *G. max* for *F. albida*; *Actinidia chinensis*, and *Musa acuminate* for *S. birrea*; and *G. max*, *Oryza sativa*, *Populus trichocarpa*, and *Sorghum bicolor* for *M. oleifera*.

For *de novo* prediction evidence, a series of training sets was made to optimize different *ab initio* gene predictors. Initially, a set of transcripts was generated by a genome-guided approach using Trinity with the parameters "--full_cleanup, --jaccard_clip, --genome_guided_max_intron 10000, --min_contig_length 200". The transcripts were then mapped back to the genome using PASA (version 2.0.2) [44] and a set of gene models with real gene characteristics (e.g., size and number of exons/introns per gene, features of splicing sites) was generated. Complete gene models were picked for training Augustus [45]. Genemark-ES (version 4.21) [46] was self-trained with default parameters. The first round of MAKER-P was run based on the evidence as above, with default parameters except "est2genome" and "protein2genome" being set to "1", yielding only RNA and protein-supported gene models. SNAP [47] was then trained with these gene models. Default parameters were used to run the second and final rounds of MAKER-P, producing the final gene models.

The number of protein-coding genes identified in each species was 31,707 in *V. subterranea*, 20,946 in *L. purpureus*, 28,979 in *F. albida*, 18,937 in *S. birrea*, and 18,451 in *M. oleifera*. Compared to the other sequenced species in the same genus [10, 11], *V. subterranea* has a more genes than mung bean (22,427) but less than adzuki bean (34,183). Various gene structure parameters were compared to the related species of each sequenced genome, as summarized in Table 5 and Additional file 1: Figure S5. BUSCO evaluation showed that at least 85% of 1,440 core genes could be identified across all the species, suggesting an acceptable quality of gene annotation for the five sequenced genomes (see Additional file 1: Table S4).

Non-coding RNA genes in the sequenced genomes were also annotated. Using BLAST, ribosomal RNA (rRNA) genes were searched against the *A. thaliana* rRNA database, or by searching for microRNAs (miRNA) and small nuclear RNA (snRNA) against the Rfam database (Rfam, RRID:SCR_004276; release 12.0) [48]. tRNAscan-SE (tRNAscan-SE, RRID:SCR_010835) was also used to scan for tRNAs [49]. The results are summarized in Table 6.

Functional annotation of protein-coding genes

Functional annotation of protein-coding genes was based on sequence similarity and domain conservation by aligning predicted amino acid sequences to public databases. Protein-coding genes were first searched against protein sequence databases for best matches, such as the Kyoto Encyclopedia of Genes and Genomes (KEGG, RRID:SCR_012773) [50], the National Center for Biotechnology Information (NCBI) non-redundant (NR) and COG databases [51], SwissProt and TrEMBL [52] using BLASTP with an E-value cut-off of 1e-5. Then, InterProScan 55.0 (InterProScan, RRID:SCR_005829) [53] was used to identify domains and motifs based on Pfam (Pfam, RRID:SCR_004726) [54], SMART (SMART, RRID:SCR_005026) [55], PANTHER (PANTHER, RRID:SCR_004869) [56], PRINTS (PRINTS, RRID:SCR_003412) [57], and ProDom (ProDom, RRID:SCR_006969) [58]. In total, 98.0%, 98.2%, 93.6%, 98.1% and 98.8% of genes in *V. subterranea*, *L. purpureus*, *F. albida*, *S.birrea*, and *M. oleifera*, respectively, were functionally annotated. Of the unannotated genes, 400, 305, 1,514, 293 and 172 were specific to *V. subterranea*, *L.*

purpureus, *F. albida*, *S. birrea*, and *M. oleifera*, respectively (Table 7).

Gene family construction

Protein and nucleotide sequences from the five sequenced species and nine other species (*A. thaliana*, *Carica papaya*, *Citrus sinensis*, *G. max*, *M. truncatula*, *O. sativa*, *P. vulgaris*, *S. bicolor*, and *Theobroma cacao*) were retrieved to construct gene families using OrthoMCL software [59] based on an all-versus-all BLASTP alignments with an E-value cutoff of 1e-5. A total of 609, 104, 499, 205 and 150 gene families were found specific to *V. subterranea*, *L. purpureus*, *F. albida*, *S. birrea*, and *M. oleifera*, respectively (see Additional file 1: Table S5).

Furthermore, the 10,103 gene families of *V. subterranea*, *L. purpureus*, *F. albida*, *M. truncatula*, and *G. max* were clustered (Figure 2A). There were 1,105 orthologous families shared by the four Papilionoideae species, while 808 gene families containing 1,966 genes were specific to *F. albida*, 281 gene families containing 538 genes were specific to *L. purpureus*, and 789 gene families containing 3,118 genes were specific to *V. subterranea.*

Moreover, 8,184 gene families of *S. birrea*, *M. oleifera*, *C. papaya*, *C. sinensis* and *T. cacao* were clustered (Figure 2B), of which 365 gene families containing 798 genes were specific to *M. oleifera*, and 362 gene families containing 796 genes were specific to *S. birrea*. KEGG pathway enrichment analysis of paralog genes was also conducted (Additional file 1: Table S6, S7). Functional annotation revealed that, in *V. subterranea*, these paralogs corresponded mainly with carbon fixation, zeatin biosynthesis, and

glyoxylate and dicarboxylate metabolism. However, for *L. purpureus*, the fatty acid elongation pathway was enriched, while in *F. albida*, pathways corresponding to plant–pathogen interactions and cyanoamino acid metabolism were enriched. In *S. birrea*, enrichment occurred in plant–pathogen interaction, starch and sucrose metabolism, and fatty acid biosynthesis pathways. In *M. oleifera*, pathways related to fatty acid and diterpenoid biosynthesis, and cyanoamino acid metabolism were enriched. Using Gene Ontology (GO) analysis, paralog genes in *V. subterranea*, *L. purpureus*, *F. albida*, *M. oleifera*, and *S. birrea* were enriched in ion binding, metabolic processes, disease resistance, cell components, and biological processes, respectively.

Phylogenetic analysis and estimation of divergence time

We identified 141 single-copy genes in the 14 species used for the above analysis, and subsequently used them to build a phylogenetic tree. Coding DNA sequence alignments of each single-copy family were generated following protein sequence alignment with MUSCLE (MUSCLE, RRID:SCR_011812) [41]. The aligned coding DNA sequences of each species were then concatenated to a supergene sequence. The phylogenetic tree was constructed with PhyML-3.0 (PhyML, RRID:SCR_014629) [60], with the HKY85+gamma substitution model on extracted four-fold degenerate sites. Divergence time was calculated using the Bayesian relaxed molecular clock method with MCMCTREE in PAML (PAML, RRID:SCR_014932) [61], based on published calibration times (39–59 Mya between *M. truncatula* and the main branch of legumes, 15–30 Mya between *G. max* and *P. vulgaris*, and 83–90 Mya between *T. cacao* and *A.*

thaliana) [11, 62].

Based on the tree constructed using single-copy-family genes, the divergence time between *F. albida* and Papilionoideae was predicted to be 79.1 (70.0–87.0) Mya. This is a little different from a previous prediction of the origin of legumes based on two gene markers (matk and rbcL) [63]. The divergence time between *M. oleifera* and *C. papaya* was predicted to be 65.4 (59.2–71.1) Mya, and 67.9 (53.6–77.3) Mya between *S. birrea* and *C. sinensis* (Figure 1).

Subsequently, to evaluate gene gain and loss, CAFE (CAFE, RRID:SCR_005983) [64] was employed to estimate the universal gene birth and death rate, λ, under a random birth and death model using the maximum likelihood method. Results for each branch of the phylogenetic tree were estimated and represented in Figure 1.

GO enrichment analysis was also conducted on gene pathways in expanded families in the lineage of each sequenced species (Additional file 1: Table S8, S9). Terms related to energy and nutrient metabolism were commonly distributed in the enrichment output of *V. subterranea, L. purpureus, M. oleifera and S. birrea*; for example, proton-transporting two-sector ATPase complex, cyclase activity, nutrient reservoir activity and carbohydrate derivative binding.

In *F. albida*, expanded gene families were related to signal transfer or regulation; e.g., signaling receptor activity, phosphatase regulator activity, and regulation of response to stimulus. Furthermore, the regulatory factors *GLABRA3*, *ENHANCER OF GLABRA 3*, *AUX1*, *LAX2*, and *LAX3* [65–67], which are related to the formation of root hairs and lateral roots, were identified in these families. As a traditional agroforestry

tree in Africa, *F. albida* was previously reported to have a root system architecture that displays wide variation under different environmental factors (soil depth, nutrient amount, or water reservoirs) [68]. This suggests its adaptability to the complex environment, which requires signal transferring and regulation. The results obtained from the GO enrichment analysis were consistent with the biological characteristics of *F. albida*.

Mining of transcription factors

Transcription factors (TFs) in the sequenced species were identified using protein sequences of plant TFs from the plant transcription factor database [69] by BLASTP search with an e-value cutoff of 10E−10, a minimum identity of 40% and a minimum query coverage of 50%. About 59 TF families were revealed across the genes in *M. truncatula*, *G. max*, *P. vulgaris*, *C. papaya*, *C. sinensis*, and the five sequenced species (see Additional file 2: Table S14). Among these TFs, bHLH, NAC, ERF, MYB-related, C2H2, MYB, WRKY, bZIP, FAR1, C3H, B3, G2-like, Trihelix, LBD, GRAS, M-type MADS, HD-ZIP, MIKC_MADS, HSF, GATA were found in abundance (Figure 4).

Identification of protein, starch, and fatty acid biosynthesis-related genes

Using the amino acid, starch and fatty acid synthesis genes in soybean [11, 70] as bait, we performed an ortholog search in *V. subterranea*, *L. purpureus*, *F. albida*, *S. birrea*, *M. oleifera*, *G. max*, *Triticum aestivum*, *Zea mays*, and *O. sativa* (Additional file 1: Tables S10–13). *V. subterranea* is a good source of resistant starch (RS) [71], which has

the potential to protect against diabetes and reduce the incidence of diarrhea and other inflammatory bowel diseases [72]. High amylose levels can contribute to RS. Previously, studies have shown that deficiency in *SSIIIa* (soluble starch synthase gene) decreases amylopectin biosynthesis and increases amylose biosynthesis by a granule-bound starch synthase (GBSSI) encoded by the *Wx* gene in *O. sativa indica* [73]. Down-regulation of the soluble starch synthase *SSII*, and of *SBE*, leads to higher levels of RS in barley [74]. Interestingly, in *V. subterranea*, two out of four GBSSs underwent expansion, suggesting their vital role in controlling starch synthesis (Figure 5) at the transcriptional and post-transcriptional level. No expansion in GBSS was observed in the genomes of *L. purpureus*, *F. albida*, *S. birrea* or *M. oleifera*, and in *V. subterranea*, soluble starch synthase was not expanded. Therefore, we speculate that the expansion of GBSS might be why *V. subterranea* is rich in RS.

Similarly, differences in the copy numbers of choline kinase, a key factor in fatty acid synthesis and storage, were found between the four legumes (*V. subterranea*, 7; *F. albida*, 4; *L. purpureus*, 2; and *G. max*, 5) and between two orphan species (*S. birrea*, 1, and *M. oleifera*, 3). Choline kinase is the first enzyme in the cytidine diphosphate–choline pathway, which is involved in lecithin biosynthesis [75, 76]. Based on these observations, we inferred that all the factors required to synthesize lecithin are present in *V. subterranea*. However, gene expression data remains lacking in terms of the GBSS and choline kinase genes in these the five species. More transcriptomic analysis and chemical tests are required to uncover the mechanisms of their nutrition metabolism.

Identification of the root nodule symbiosis pathway

Legumes (Fabaceae) are well known for their ability to fix nitrogen; an important trait to replenish nitrogen supplies in soil and agricultural systems. Being part of the human food production chain, legumes have a major impact on the global nitrogen cycle. Nitrogen-fixing plants can fix nitrogen through root nodule symbiosis (RNS) using symbiotic nitrogen-fixing bacteria. In a previous report, RNS was revealed to be restricted to Fabales, Fagales, Cucurbitales, and Rosales, which together form the monophyletic nitrogen-fixing clade. This suggests a predispositional event in their common ancestor, which enabled their subsequent evolution [77]. Despite this genetic predisposition, many leguminous members of the nitrogen-fixing clade are non-fixers [78]. This has raised the question as to whether the nodulation trait evolved independently in a convergent manner, or originated from a single evolutionary event followed by multiple losses. The answer to this question cannot be explained with current genomic approaches, because available genomic information of nodulating species is, at present, limited to a single subfamily, the Papilionoideae, in the Fabaceae. Although the Mimosoideae subfamily within the Fabaceae also contains nitrogen-fixing species, none of its members have been genome-sequenced.

In this analysis, we identified 16 root nodulation symbiosis signal (Sym) pathway genes in three legumes (*V. subterranea*, *L. purpureus,* and *F. albida*) and two non-legumes (*S. birrea* and *M. oleifera*). First, we collected the protein sequences of previously reported genes in the Sym pathways of *L. japonicus* and *M. truncatula* [79] (Figure 3). Using these sequences as bait, we predicted the Sym genes in *V. subterranea*,

L. purpureus, *F. albida*, *S. birrea*, and *M. oleifera* through reciprocal best hits generated by a BLASTP search with an E-value of 1e-5 (Table 8). To verify this prediction with syntenic analysis, 'all versus all' BLASTP results were subjected to MCSCANX [80] with default parameters to generate syntenic blocks. The result showed that, among the legumes, all of the components in the pathway were conserved except for *MtNFP*/*LjNFR5*, *LjCASTOR*, *CCaMK*, *MtCRE1*/*LjLHK1*, and *NF-YA2*, while many components were missing in the non-legumes. Among the three legumes, the orthologous genes *MtNFP/LjNFR5*, *LjCASTOR* and *MtIPD3/LjCYCLOPS* were absent in *F. albida*. As previously reported, the expression of *NIN* is lower in the *ipd3*-mutant line [81]; analysis of the *M. truncatula* mutant C31 showed that the Nod Factor Perception gene is essential in Nod factor perception at early stages of the symbiotic interaction [82]. Meanwhile, the function of *IPD3* was proved to be partly redundant, which means it is likely that other proteins phosphorylated by CCaMK can partially fulfill this role when *IPD3* is absent [81]. Differences in the components of the RNS pathway (Table 8), together with the relatively weak nitrogen-fixing ability [83] of *F. albida*, is thus a good reference for RNS diversification research.

Conclusion

This comprehensive study reports the sequencing, assembly, and annotation of five genomes of underutilized plants in Africa, along with details of their key evolutionary features. The draft genomes of these species will serve as an important complementary resource for non-model food crops, especially the leguminous plants, and will be

valuable for both agroforestry and evolutionary research. Improving these underutilized plants using genomics-assisted tools and methods could help to bring food security to millions of people.

Additional files

Figure S1: K-mer (K = 17) analysis of five genomes.

Figure S2: Distribution of sequencing depths of the assembly data.

Figure S3: GC content.

Figure S4: Comparison of GC content between closely related species.

Figure S5: Statistical analysis of gene models in *Vigna subterranea, Lablab purpureus, Faidherbia albida, Moringa oleifera and Sclerocarya birrea*.

Figure S6: Expansion and contraction of gene families.

Table S1: Statistical analysis of raw and clean DNA sequencing data

Table S2: Summary statistics of the transcriptome data of four species

Table S3: Estimation of genome size based on k-mer analysis of five species

Table S4: BUSCO evaluation of the annotated protein-coding genes of five species

Table S5: Analysis of gene families of different species

Table S6: Enriched pathways of unique paralog genes in families

Table S7: Enriched GO terms (level 3) of unique paralog genes in families

Table S8: Enriched GO terms (level 3) of genes in families with expansion

Table S9: Enriched pathways of genes in families with expansion

Table S10: Copy numbers of protein biosynthesis-related genes in each species

Table S11: Copy numbers of starch biosynthesis-related genes in each species

Table S12: Copy numbers of fatty acid synthesis and storage-related genes in each species

Table S13: Copy numbers of fatty acid degradation-related genes in each species

Table S14: Numbers of transcription factors in the studied species

Abbreviations

BUSCO: Benchmarking Universal Single-Copy Orthologs; LTR: long terminal repeat; TF: transcription factors; MITE: miniature inverted repeat transposable elements; NCBI: National Center for Biotechnology Information; RNS: root nodule symbiosis; RS.

Funding

This work was supported by funding from the Shenzhen Municipal Government of China (grant numbers JCYJ20150831201643396 and JCYJ20150529150409546), and the Guangdong Provincial Key Laboratory of Genome Read and Write (grant number 2017B030301011). This work is part of the 10KP project led by BGI-Shenzhen and China National GeneBank.

Availability of supporting data

The raw data from our genome project was deposited in the NCBI Sequence Read Archive database with Bioproject IDs PRJNA453822 and PRJNA474418. Assembly

and annotation of the five genomes and other supporting data, including BUSCO results, are available in the GigaDB repository [84], and the data reported in this study are also available in the CNGB Nucleotide Sequence Archive (CNSA: https://db.cngb.org/cnsa ; accession number CNP0000096).

Competing interests

The authors declare that they have no competing interests.

Author contributions

X.L., X.X., H.Y., J.W., P.S.H., R.J., A.V. and Y.C. conceived the project and supervised the respective components: DNA extraction, sample logistics and collection conducted by the African Orphan Crops Consortium of the World Agroforestry Centre; and data generation and analyses conducted by BGI. Y.C. supervised the analyses. R.K. and S.M. collected and extracted the DNA and RNA. S.B. and F.Y. performed the genome assembly. M.L., X.Z.L., S.B.W. and L.Z.L. performed the genome annotation, gene family analysis and identification of genes related to root growth and root nodule symbiosis. Y.C., M.L, X.Z.L. performed the phylogenetic analysis. Y.C., H.L., S.K.S., P.S.H. and A.V. wrote the manuscript. H.R.L. and S.F.P. sequenced the samples. S.M., W.K.H., A.M., P.S.H., J.W., H.M.Y. revised the manuscript. All authors read, edited and approved the final version of the manuscript.

References

1. United Nations, Department of Economic and Social Affairs, Population Division. World population prospects: the 2017 revision, Key Findings and Advance Tables. 2017. Working Paper No. ESA/P/WP/248.

2. Development Initiatives. Global nutrition report 2017: nourishing the SDGs. Bristol, UK: Development Initiatives. 2017.

3. Mouillé, B., Charrondière, U. R., & Burlingame. The contribution of plant genetic resources to health and dietary diversity. Thematic Background Study. 2010.

4. African Orphan Crops Consortium: http://www.africanorphancrops.org (2018). Accessed 20 Nov 2018.

5. Varshney RK, Chen W, Li Y, Bharti AK, Saxena RK, Schlueter JA, et al. Draft genome sequence of pigeonpea (*Cajanus cajan*), an orphan legume crop of resource-poor farmers. Nat Biotechnol. 2011;30:83-89. doi:10.1038/nbt.2022.

6. Foyer CH, Lam H-M, Nguyen HT, Siddique KHM, Varshney RK, Colmer TD, et al. Neglecting legumes has compromised human health and sustainable food production. Nat Plants. 2016;2:16112. doi:10.1038/nplants.2016.112.

7. Borget M. Food legumes. In: The Tropical Agriculturalist, CTA Macmillan. 1992.

8. Linnemann A.R, Azam–Ali S.N. Bambara groundnut (*Vigna subterranea*) literature review: A revised and updated bibliography. Tropical Crops Communication No. 7. 1993.

9. Gbaguidi AA, Dansi A, Dossou-Aminon I, Gbemavo DSJC, Orobiyi A,

Sanoussi F, et al. Agromorphological diversity of local Bambara groundnut (*Vigna subterranea* (L.) Verdc.) collected in Benin. Genet Resour Crop Evol. 2018;65(4):1159-1171. doi:10.1007/s10722-017-0603-4.

10. Kang YJ, Kim SK, Kim MY, Lestari P, Kim KH, Ha B-K, et al. Genome sequence of mungbean and insights into evolution within Vigna species. Nat Commun. 2014;5:5443. doi:10.1038/ncomms6443.

11. Yang K, Tian Z, Chen C, Luo L, Zhao B, Wang Z, et al. Genome sequencing of adzuki bean (*Vigna angularis*) provides insight into high starch and low fat accumulation and domestication. Proc Natl Acad Sci U S A. 2015;112(43):13213-13218. doi:10.1073/pnas.1420949112.

12. Jung IL. Soluble extract from *Moringa oleifera* leaves with a new anticancer activity. PLoS One. 2014;9(4):e95492. doi:10.1371/journal.pone.0095492.

13. Leone A, Spada A, Battezzati A, Schiraldi A, Aristil J and Bertoli S. Cultivation, genetic, ethnopharmacology, phytochemistry and pharmacology of *Moringa oleifera* Leaves: An Overview. Int J Mol Sci. 2015;16(6):12791-12835. doi:10.3390/ijms160612791.

14. Lea M. Bioremediation of turbid surface water using seed extract from *Moringa oleifera* Lam. (drumstick) tree. Curr Protoc Microbiol. 2014;33:1G.2.1-G.2.8. doi:10.1002/9780471729259.mc01g02s16.

15. Mabapa MP, Ayisi KK, Mariga IK, Mohlabi RC and Chuene RS. Production and utilization of moringa by farmers in Limpopo Province, South Africa. International Journal of Agricultural Research. 1962;12(4):160-171.

doi:10.3923/ijar.2017.160.171.

16. Tian Y, Zeng Y, Zhang J, Yang CG, Yan L, Wang XJ, et al. High quality reference genome of drumstick tree (*Moringa oleifera* Lam.), a potential perennial crop. Science China Life Sciences. 2015;58(7):627-638. doi:10.1007/s11427-015-4872-x.

17. Maass BL, Knox MR, Venkatesha SC, Angessa TT, Ramme S and Pengelly BC. *Lablab purpureus*-a crop lost for Africa? Trop Plant Biol. 2010;3(3):123-135. doi:10.1007/s12042-010-9046-1.

18. Robotham O and Chapman M. Population genetic analysis of hyacinth bean (*Lablab purpureus* (L.) Sweet, Leguminosae) indicates an East African origin and variation in drought tolerance. Genet Resour Crop Evol. 2017;64(1):139-148. doi:10.1007/s10722-015-0339-y.

19. Kamotho GN. Evaluation of adaptability potential and genetic diversity of Kenyan Dolichos bean germplasm. PhD thesis. 2015.

20. Vankatesha S.C. Molecular characterization and development of mapping populatuions for construction of genetic map in dolichos bean. PhD thesis. 2012.

21. Mokgolodi NC, Setshogo MP, Shi L-l, Liu Y-j and Ma C. Achieving food and nutritional security through agroforestry: a case of *Faidherbia albida* in sub-Saharan Africa. For. Stud. China. 2011;13(2):123-131. doi:10.1007/s11632-011-0202-y.

22. Garrity DP, Akinnifesi FK, Ajayi OC, Weldesemayat SG, Mowo JG, Kalinganire A, et al. Evergreen agriculture: a robust approach to sustainable

food security in Africa. Food Sec. 2010;2(3):197-214. doi:10.1007/s12571-010-0070-7.

23. DUNHAM KM. Biomass dynamics of herbaceous vegetation in Zambezi riverine woodlands. African Journal of Ecology. 1990;28(3):200-212. doi:10.1111/j.1365-2028.1990.tb01153.x.

24. Barnes RD and Fagg CW. *Faidherbia albida* monograph and annotated bibliography. Oxford Forestry Inst. 2003;41-267

25. Nerd A, Mizrahi Y, Janick J and Simon JE. Domestication and introduction of marula (*Sclerocarya birrea* subsp. *caffra*) as a new crop for the Negev Desert of Israel. New crops. 1993;496-499.

26. Mng’Omba SA, Sileshi GW, Jamnadass R, Akinnifesi FK and Mhango J. Scion and stock diameter size effect on growth and fruit production of *Sclerocarya birrea* (Marula) trees. J Hortic For. 2012;4(9):153-60.

27. Gouwakinnou GN, Lykke AM, Assogbadjo AE and Sinsin B. Local knowledge, pattern and diversity of use of *Sclerocarya birrea*. J Ethnobiol Ethnomed. 2011;7 (1):1-9. doi:10.1186/1746-4269-7-8.

28. Yang T and Wu C. DNA Extraction for plant samples by CTAB. protocols.io. 2018; dx.doi.org/10.17504/protocols.io.pzqdp5w

29. Luo R, Liu B, Xie Y, Li Z, Huang W, Yuan J, et al. SOAPdenovo2: an empirically improved memory-efficient short-read de novo assembler. GigaScience. 2012;1(1):1-6. doi:10.1186/2047-217X-1-18.

30. Teh BT, Lim K, Yong CH, Ng CCY, Rao SR, Rajasegaran V, et al. The draft

genome of tropical fruit durian (*Durio zibethinus*). Nat Genet. 2017;49:1633-1641. doi:10.1038/ng.3972.

31. Simao FA, Waterhouse RM, Ioannidis P, Kriventseva EV and Zdobnov EM. BUSCO: assessing genome assembly and annotation completeness with single-copy orthologs. Bioinformatics. 2015;31(19):3210-3212. doi:10.1093/bioinformatics/btv351.

32. Chang Z, Li G, Liu J, Zhang Y, Ashby C, Liu D, et al. Bridger: a new framework for de novo transcriptome assembly using RNA-seq data. Genome Biol. 2015;16:30. doi:10.1186/s13059-015-0596-2.

33. Kent WJ. BLAT--the BLAST-like alignment tool. Genome Res. 2002;12(4):656-664. doi:10.1101/gr.229202.

34. Li R, Yu C, Li Y, Lam TW, Yiu SM, Kristiansen K, et al. SOAP2: an improved ultrafast tool for short read alignment. Bioinformatics. 2009;25(15):1966-1967. doi:10.1093/bioinformatics/btp336.

35. Tarailo-Graovac M and Chen N. Using RepeatMasker to identify repetitive elements in genomic sequences. Curr Protoc Bioinformatics. 2009;25(1) 4.10.1-4.10.14. doi:10.1002/0471250953.bi0410s25.

36. Han Y and Wessler SR. MITE-Hunter: a program for discovering miniature inverted-repeat transposable elements from genomic sequences. Nucleic Acids Res. 2010;38(22):e199-e199. doi:10.1093/nar/gkq862.

37. Ellinghaus D, Kurtz S and Willhoeft U. LTRharvest, an efficient and flexible software for de novo detection of LTR retrotransposons. BMC Bioinformatics.

2008;9:18. doi:10.1186/1471-2105-9-18.

38. Gremme G, Steinbiss S and Kurtz S. GenomeTools: a comprehensive software library for efficient processing of structured genome annotations. IEEE/ACM Trans Comput Biol Bioinform. 2013;10(3):645-656. doi:10.1109/tcbb.2013.68.

39. Steinbiss S, Willhoeft U, Gremme G and Kurtz S. Fine-grained annotation and classification of de novo predicted LTR retrotransposons. Nucleic Acids Res. 2009;37(21):7002-7013. doi:10.1093/nar/gkp759.

40. Chan PP and Lowe TM. GtRNAdb 2.0: an expanded database of transfer RNA genes identified in complete and draft genomes. Nucleic Acids Res. 2016;44 (D1):D184-D9. doi:10.1093/nar/gkv1309.

41. Edgar RC. MUSCLE: multiple sequence alignment with high accuracy and high throughput. Nucleic Acids Res. 2004;32(5):1792-1797. doi:10.1093/nar/gkh340.

42. Campbell MS, Holt C, Moore B and Yandell M. Genome annotation and curation using MAKER and MAKER-P. Curr Protoc Bioinformatics. 2014;48(1): 4.11.1-4.11.39. doi:10.1002/0471250953.bi0411s48.

43. Haas BJ, Papanicolaou A, Yassour M, Grabherr M, Blood PD, Bowden J, et al. De novo transcript sequence reconstruction from RNA-seq using the Trinity platform for reference generation and analysis. Nat Protoc. 2013;8:1494–1512. doi:10.1038/nprot.2013.084.

44. Haas BJ, Salzberg SL, Zhu W, Pertea M, Allen JE, Orvis J, et al. Automated eukaryotic gene structure annotation using EVidenceModeler and the program to assemble spliced alignments. Genome Biol. 2008;9(1):R7. doi:10.1186/gb-

2008-9-1-r7.

45. Stanke M, Schoffmann O, Morgenstern B and Waack S. Gene prediction in eukaryotes with a generalized hidden Markov model that uses hints from external sources. BMC Bioinformatics. 2006;7:62. doi:10.1186/1471-2105-7-62.

46. Lomsadze A, Ter-Hovhannisyan V, Chernoff YO and Borodovsky M. Gene identification in novel eukaryotic genomes by self-training algorithm. Nucleic Acids Res. 2005;33(20):6494-6506. doi:10.1093/nar/gki937.

47. Korf I. Gene finding in novel genomes. BMC Bioinformatics. 2004;5:59. doi:10.1186/1471-2105-5-59.

48. Nawrocki EP, Burge SW, Bateman A, Daub J, Eberhardt RY, Eddy SR, et al. Rfam 12.0: updates to the RNA families database. Nucleic Acids Res. 2015;43(D1):D130-D137. doi:10.1093/nar/gku1063.

49. Lowe TM and Chan PP. tRNAscan-SE On-line: integrating search and context for analysis of transfer RNA genes. Nucleic Acids Res. 2016;44(W1):W54-W57. doi:10.1093/nar/gkw413.

50. Tanabe M and Kanehisa M. Using the KEGG database resource. Curr Protoc Bioinformatics. 2012; 38(1):1.12.1-1.12.43. doi:10.1002/0471250953.bi0112s38.

51. Tatusov RL, Koonin EV and Lipman DJ. A genomic perspective on protein families. Science. 1997;278(5338):631-637.

52. Boeckmann B, Bairoch A, Apweiler R, Blatter MC, Estreicher A, Gasteiger E,

et al. The SWISS-PROT protein knowledgebase and its supplement TrEMBL in 2003. Nucleic Acids Res. 2003;31(1):365-370.

53. Jones P, Binns D, Chang HY, Fraser M, Li W, McAnulla C, et al. InterProScan 5: genome-scale protein function classification. Bioinformatics. 2014;30(9):1236-1240. doi:10.1093/bioinformatics/btu031.

54. Finn RD, Mistry J, Tate J, Coggill P, Heger A, Pollington JE, et al. The Pfam protein families database. Nucleic Acids Res. 2010;38 suppl 1:D211-D222. doi:10.1093/nar/gkp985.

55. Letunic I, Doerks T and Bork P. SMART 6: recent updates and new developments. Nucleic Acids Res. 2009;37 suppl 1:D229-D232. doi:10.1093/nar/gkn808.

56. Mi H, Muruganujan A, Casagrande JT and Thomas PD. Large-scale gene function analysis with the PANTHER classification system. Nat Protoc. 2013;8:1551-1566. doi:10.1038/nprot.2013.092

57. Attwood TK, Bradley P, Flower DR, Gaulton A, Maudling N, Mitchell AL, et al. PRINTS and its automatic supplement, prePRINTS. Nucleic Acids Res. 2003;31(1):400-402.

58. Corpet F, Servant F, Gouzy J and Kahn D. ProDom and ProDom-CG: tools for protein domain analysis and whole genome comparisons. Nucleic Acids Res. 2000;28(1):267-269.

59. Stichting C, Centrum M and Dongen SV. A Cluster Algorithm for Graphs. Information Systems [INS]. 2000:1-40.

60. Guindon S, Dufayard JF, Lefort V, Anisimova M, Hordijk W and Gascuel O. New algorithms and methods to estimate maximum-likelihood phylogenies: assessing the performance of PhyML 3.0. Syst Biol. 2010;59(3):307-321. doi:10.1093/sysbio/syq010.

61. Yang Z. PAML 4: Phylogenetic analysis by maximum likelihood. Mol Biol Evol. 2007;24(8):1586-1591. doi:10.1093/molbev/msm088.

62. He N, Zhang C, Qi X, Zhao S, Tao Y, Yang G, et al. Draft genome sequence of the mulberry tree *Morus notabilis*. Nat Commun. 2013;4:2445. doi:10.1038/ncomms3445.

63. Lavin M, Herendeen PS, Wojciechowski MF and Linder P. Evolutionary rates analysis of leguminosae implicates a rapid diversification of lineages during the Tertiary. Syst Biol. 2005;54(4):575-594. doi:10.1080/10635150590947131.

64. De Bie T, Cristianini N, Demuth JP and Hahn MW. CAFE: a computational tool for the study of gene family evolution. Bioinformatics. 2006;22(10):1269-1271. doi:10.1093/bioinformatics/btl097.

65. Bernhardt C, Lee MM, Gonzalez A, Zhang F, Lloyd A and Schiefelbein J. The bHLH genes GLABRA3 (GL3) and ENHANCER OF GLABRA3 (EGL3) specify epidermal cell fate in the Arabidopsis root. Development. 2003;130(26):6431-6439. doi:10.1242/dev.00880.

66. Paponov IA, Paponov M, Teale W, Menges M, Chakrabortee S, Murray JA, et al. Comprehensive transcriptome analysis of auxin responses in Arabidopsis. Mol Plant. 2008;1(2):321-337. doi:10.1093/mp/ssm021.

67. Vanneste S, Rybel BD, Beemster GTS, Ljung K, Smet ID, Isterdael GV, et al. Cell cycle progression in the pericycle is not sufficient for SOLITARY ROOT/IAA14-mediated lateral root initiation in *Arabidopsis thaliana*. Plant Cell. 2005;17(11):3035-3050. doi:10.1105/tpc.105.035493.

68. Vandenbeldt RJ. *Faidherbia albida* in the West African semi-arid tropics. ICRISAT. 1992. p. 107-110.

69. Jin J, Tian F, Yang D-C, Meng Y-Q, Kong L, Luo J, et al. PlantTFDB 4.0: toward a central hub for transcription factors and regulatory interactions in plants. Nucleic Acids Res. 2017;45(D1):D1040-D5. doi:10.1093/nar/gkw982.

70. Jang YE, Kim MY, Shim S, Lee J and Lee S-H. Gene expression profiling for seed protein and oil synthesis during early seed development in soybean. Genes Genom. 2015;37(4):409-418. doi:10.1007/s13258-015-0269-2.

71. Bamshaiye OM, Adegbola JA and Bamishaiye EI. Bambara groundnut : an under-utilized nut in Africa. Adv Agric Biotechnol. 2011;1:60-72.

72. Raigond P, Ezekiel R and Raigond B. Resistant starch in food: a review. J Sci Food Agric. 2015;95(10):1968-1978.

73. Zhou H, Wang L, Liu G, Meng X, Jing Y, Shu X, et al. Critical roles of soluble starch synthase SSIIIa and granule-bound starch synthase Waxy in synthesizing resistant starch in rice. Proc Natl Acad Sci U S A. 2016;113(45):12844-12849. doi:10.1073/pnas.1615104113.

74. Bird AR, Flory C, Davies DA, Usher S and Topping DL. A novel barley cultivar (*Himalaya 292*) with a specific gene mutation in starch synthase IIa raises large

bowel starch and short-chain fatty acids in rats. J Nutr. 2004;134(4):831-835. doi:10.1093/jn/134.4.831.

75. Morre DJ, Nyquist S and Rivera E. Lecithin biosynthetic enzymes of onion stem and the distribution of phosphorylcholine-cytidyl transferase among cell fractions. Plant Physiol. 1970;45(6):800-804.

76. Johnson KD and Kende H. Hormonal control of lecithin synthesis in barley aleurone cells: regulation of the CDP-choline pathway by gibberellin. Proc Natl Acad Sci U S A. 1971;68(11):2674-2677.

77. Soltis DE, Soltis PS, Morgan DR, Swensen SM, Mullin BC, Dowd JM, et al. Chloroplast gene sequence data suggest a single origin of the predisposition for symbiotic nitrogen fixation in angiosperms. Proc Natl Acad Sci U S A. 1995;92(7):2647-2651.

78. Doyle JJ. Phylogenetic perspectives on the origins of nodulation. Mol Plant Microbe Interact. 2011;24(11):1289-1295. doi:10.1094/MPMI-05-11-0114.

79. Geurts R, Xiao TT and Reinhold-Hurek B. What does it take to evolve a nitrogen-fixing endosymbiosis? Trends Plant Sci. 2016;21 (3):199–208. doi:10.1016/j.tplants.2016.01.012.

80. Wang Y, Tang H, Debarry JD, Tan X, Li J, Wang X, et al. MCScanX: A toolkit for detection and evolutionary analysis of gene synteny and collinearity. Nucleic Acids Res. 2012;40(7):e49. doi:10.1093/nar/gkr1293.

81. Horváth B, Li HY, Domonkos Á, Halász G, Gobbato E, Ayaydin F, et al. *Medicago truncatula* IPD3 is a member of the common symbiotic signaling

pathway required for rhizobial and mycorrhizal symbioses. Mol Plant Microbe Interact. 2011;24(11):1345-1358. doi:10.1094/MPMI-01-11-0015.

82. Amor BB, Shaw SL, Oldroyd GED, Maillet F, Penmetsa RV, Cook D, et al. The NFP locus of *Medicago truncatula* controls an early step of Nod factor signal transduction upstream of a rapid calcium flux and root hair deformation. Plant J. 2003;34(4):495-506.

83. Ndoye I, Gueye M, Danso SKA and Dreyfus B. Nitrogen fixation in *Faidherbia albida*, *Acacia raddiana*, *Acacia senegal* and *Acacia seyal* estimated using the ^{15}N isotope dilution technique. Plant Soil. 1995;172(2):175-180. doi:10.1007/BF00011319.

84. Chang Y, Liu H, Liu M, Liao X, Sahu SK, Fu Y, Song B; Cheng S, Kariba R, Muthemba S, Hendre PS, Mayes S, Ho WK, Kendabie P, Wang S, Li L, Muchugi A, Jamnadass R, Lu H, Peng S, Deynze AV, Simons A, Yana-Shapiro H, Xu X, Yang H, Wang J, Liu X. Supporting data for "The draft genomes of five agriculturally important African orphan crops". GigaScience Database 2018. http://dx.doi.org/10.5524/100504.

85. Bioinformatics & Evolutionary Genomics: http://bioinformatics.psb.ugent.be/webtools/Venn/. Accessed 20 Nov 2018.

Table 1: Statistical analysis of the final *de novo* genome assembly of *Vigna subterranea*, *Lablab purpureus*, *Faidherbia albida*, *Sclerocarya birrea* and *Moringa oleifera*

**Parameters**		***V. subterranea***		***L. purpureus***		***F. albida***		***S. birrea***		***M. oleifera***	
		**Contig**	**Scaffold**	**Contig**	**Scaffold**	**Contig**	**Scaffold**	**Contig**	**Scaffold**	**Contig**	**Scaffold**
**Length (bp)**	N90	3,804	75,271	785	860	8,254	95,167	3,661	21,833	6,676	57,837
	N80	7,872	197,296	8,009	61,348	16,321	251,730	7,649	82,385	16,503	241,828
	N70	11,464	325,826	16,144	205,392	24,165	380,587	11,885	155,416	25,754	441,152
	N60	15,122	474,616	24,010	359,168	32,440	534,880	16,393	243,236	35,081	644,014
	N50	19,154	640,666	32,223	621,373	42,029	692,039	21,349	335,449	45,268	957,246
	N40	23,828	865,081	42,690	950,808	53,479	881,230	26,914	485,585	58,406	1,446,587
	N30	29,382	1,133,817	54,401	1,489,002	69,167	1,197,388	33,914	705,409	74,710	1,878,891
	N20	36,928	1,503,436	70,790	1,971,744	92,147	1,501,241	43,984	1,098,843	96,626	2,565,629
	N10	49,695	2,049,645	95,643	2,606,483	139,388	1,925,526	62,875	2,089,533	136,952	3,296,678

	N90	29,245	1,087	26,272	9,409	16,834	1,132	17,585	1,537	5,524	366
	N80	20,188	664	9,869	715	11,420	727	11,678	787	3,574	191
	N70	14,829	453	6,576	366	8,198	514	8,313	499	2,542	125
	N60	10,943	315	4,630	222	5,898	370	6,001	332	1,833	84
**Number**	N50	7,932	220	3,244	138	4,151	263	4,277	214	1,295	56
	N40	5,532	147	2,204	86	2,791	179	2,929	131	876	37
	N30	3,590	93	1,403	52	1,728	114	1,857	74	553	24
	N20	2,024	52	776	29	912	64	1,012	36	300	13
	N10	806	22	306	12	326	26	387	12	112	6
Maximum length		148,612	3,684,321	240,194	5,699,750	529,842	4,746,824	227,874	5,850,796	449,426	4,637,711
Total length		512,516,846	535,052,523	385,303,786	395,472,305	644,456,383	653,726,905	322,977,033	330,983,508	213,739,255	216,759,177
Total number ⩾ 100 bp		104,575	65,586	135,039	118,976	75,572	51,470	64,158	40,280	29,972	22,329

Total number ⩾ 2000 bp	35,465	2,920	15,984	4,265	26,459	5,758	22,172	4,852	8,300	2,166
N content (%)	4.21		2.57		1.42		2.42		1.39	

Table 2: Completeness evaluation of genome assembly using BUSCO database in five species

**BUSCOs**	***Vigna subterranea***		***Lablab purpureus***		***Faidherbia albida***		***Sclerocarya birrea***		***Moringa oleifera***	
	**N**	**%**	**N**	**%**	**N**	**%**	**N**	**%**	**N**	**%**
Complete single copy	1,244	86.39	1,258	87.40	1,231	85.50	1352	93.90	1,278	88.80
Complete duplicated	82	5.69	83	5.80	84	5.80	32	2.20	19	1.30
Fragmented	28	1.94	20	1.40	34	2.40	21	1.50	23	1.60
Missing	86	5.97	79	5.40	91	6.30	35	2.40	120	8.30
Total	1440	/	1440	/	1440	/	1440	/	1440	/

Abbreviation: BUSCO, Benchmarking universal single-copy orthologs; N, number

Table 3: Gene coverage of candidate species based on transcriptome data

**Species**	**Dataset**	**Number**	**Total length (bp)**	**Base coverage by assembly (%)**	**Sequence coverage by assembly (%)**
	All	116,223	161,077,155	89.61	98.21
Vigna	>200 bp	116,223	161,077,155	89.61	98.21
subterranea	>500 bp	72,139	147,068,299	89.03	98.00
	>1000 bp	47,952	129,884,929	88.33	97.52
	All	86,867	80,837,182	93.59	99.25
Lablab	>200 bp	86,867	80,837,182	93.59	99.25
purpureus	>500 bp	41,252	66,764,786	92.94	99.18
	>1000 bp	24,627	55,074,989	92.32	99.02
	All	50,294	46,650,067	93.62	98.85
Faidherbia	>200 bp	50,294	46,650,067	93.62	98.85
albida	>500 bp	26,352	39,282,694	93.32	99.05
	>1000 bp	15,569	31,560,858	92.78	98.95
	All	60,964	57,114,636	88.98	92.16
Moringa	>200 bp	60,964	57,114,636	88.98	92.16
oleifera	>500 bp	29,581	47,523,018	88.85	92.69
	>1000 bp	18,322	39,528,310	88.70	92.99

Table 4: Proportion of different classes of repeats (%) in five species

Repeat type	*Vigna subterranea*		*Lablab purpureus*		*Faidherbia albida*		*Sclerocarya birrea*		*Moringa oleifera*	
	**% in genome**	**Length (bp)**	**% in genome**	**Length (bp)**	**% in genome**	**Length (bp)**	**% in genome**	**Length (bp)**	**%in genome**	**Length (bp)**
SINE	0	313	0.005	19,444	< 0.01	1,966	0.02	69,836	0.11	248,569
LINE	0.25	1,387,567	0.45	1,784,785	0.91	6,003,271	0.19	647,579	1.83	3,970,802
LTR	19.77	105,828,735	23.78	94,062,428	44.65	291,901,514	38.78	128,362,381	22.69	49,200,625
DNA	7.15	38,294,871	4.76	18,851,402	4	26,164,519	1.76	5,829,982	5.81	12,599,607
Satellite	0.01	71,679	0.02	107,451	0.01	110,749	0	18,597	0.74	1,623,399
Simple repeat	0.35	1,922,719	0.2	821,773	0.04	308,481	0.04	153,135	0.29	630,662
Others	11.94	63,926,350	8.95	35,400,400	6.48	42,426,306	5.11	16,918,179	10.35	22,439,026
**Total**	**38.35**	**205,189,285**	**37.18**	**147,050,327**	**54.86**	**358,653,534**	**45.18**	**149,551,125**	**40.57**	**87,944,150**

Abbreviations: bp, base pairs; DNA, deoxyribonucleic acid; LINE, long interspersed nuclear element; LTR, long terminal repeats; SINE, short interspersed nuclear element.

Table 5: Gene structure parameters of *Vigna subterranea*, *Lablab purpureus*, *Faidhervia albida*, *Medicago truncatula*, *Glycine max*, *Sclerocarya birrea*, *Moringa oleifera*, *Carica papaya*, *Theobroma cacao* and *Citrus sinensis*

Species	Protein-coding gene number	Mean gene length (bp)	Mean coding sequence length (bp)	Mean exons per gene	Mean exon length (bp)	Mean intron length (bp)
V. subterranea	31,707	3,287	1,163	5	222	501
L. purpureus	20,946	3,696	1,276	5	239	557
F. albida	28,979	3,396	1,207	5	226	504
M. truncatula	50,358	2,334	986	4	243	440
G. max	55,137	3,144	1,169	5	232	488
S. birrea	18,937	3,561	1,343	6	239	479
M. oleifera	18,451	3,308	1,238	5	232	478
C. papaya	24,107	2,531	962	4	223	473
T. cacao	41,951	3,684	1,323	6	223	479
C. sinensis	35,182	3,797	1,424	6	237	475

Table 6: Annotation of non-coding RNA genes in the genomes of *Vigna subterranea*, *Lablab purpureus*, *Faidherbia albida*, *Sclerocarya birrea* and *Moringa oleifera*

Species		Type	Copy	Average length (bp)	Total length (bp)	% of genome
V. subterranea		miRNA	102	122	12,466	0.002330
		tRNA	756	75	56,639	0.010586
	rRNA	rRNA	1,080	124	134,185	0.025079
		18S	55	560	30,798	0.005756

		28S	62	126	7,793	0.001456
		5.8S	17	124	2,110	0.000394
		5S	946	99	93,484	0.017472
	snRNA	snRNA	523	117	61,006	0.011402
		CD-box	327	100	32,643	0.006101
		HACA-box	47	133	6,236	0.001165
		splicing	149	149	22,127	0.004135
L. purpureus		miRNA	109	123	13,398	0.003388
		tRNA	611	75	45,748	0.011568
	rRNA	rRNA	633	227	143,466	0.036277
		18S	213	446	95,074	0.024041
		28S	283	121	34,186	0.008644
		5.8S	53	135	7,177	0.001815
		5S	84	84	7,029	0.001777
	snRNA	snRNA	457	118	54,029	0.013662
		CD-box	278	97	26,915	0.006806
		HACA-box	48	133	6,371	0.001611
		splicing	131	158	20,743	0.005245
F. albida		miRNA	126	122	15,364	0.002350
		tRNA	458	75	34,388	0.005260
	rRNA	rRNA	1,008	107	107,518	0.016447
		18S	25	321	8,034	0.001229
		28S	26	118	3,063	0.000469
		5.8S	6	118	710	0.000109
		5S	951	101	95,711	0.014641
	snRNA	snRNA	1,996	108	216,482	0.033115
		CD-box	1,836	106	194,676	0.029779

		HACA-box	42	132	5,548	0.000849
		splicing	118	138	16,258	0.002487
S. birrea		miRNA	106	122	12,899	0.003897
		tRNA	564	75	42,181	0.012744
	rRNA	rRNA	313	142	44,378	0.013408
		18S	80	240	19,239	0.005813
		28S	57	113	6,460	0.001952
		5.8S	16	103	1,644	0.000497
		5S	160	106	17,035	0.005147
	snRNA	snRNA	841	115	96,517	0.029161
		CD-box	638	105	67,216	0.020308
		HACA-box	34	124	4,217	0.001274
		splicing	169	148	25,084	0.007579
M. oleifera		miRNA	111	119	13,161	0.006072
		tRNA	1,241	75	93,620	0.043191
	rRNA	rRNA	8,406	309	2,598,079	1.198602
		18S	3,256	608	1,979,080	0.913032
		28S	3,808	113	430,280	0.198506
		5.8S	1,182	150	177,612	0.08194
		5S	160	69	11,107	0.005124
	snRNA	snRNA	229	119	27,158	0.012529
		CD-box	119	97	11,578	0.005341
		HACA-box	38	132	4,999	0.002306
		splicing	72	147	10,581	0.004881

Table 7: Statistical analysis of the functional annotations of protein-coding genes in the genomes of *Vigna subterranea*, *Lablab purpureus*, *Faidherbia albida*, *Sclerocarya birrea* and *Moringa oleifera*

Database	***V. subterranea***		***L. purpureus***		***F. albida***		***S. birrea***		***M. oleifera***	
	**N**	**%**	**N**	**%**	**N**	**%**	**N**	**%**	**N**	**%**
Nr	31,013	97.81	20,540	98.06	27,021	93.24	18,547	97.94	18,203	98.65
Swissprot	22,496	70.95	15,905	75.93	21,247	73.32	15,513	81.92	15,109	81.88
KEGG	22,141	69.83	14,699	70.18	20,184	69.65	14,623	77.22	14,044	76.11
COG	10,814	34.11	7,854	37.50	10,526	36.32	7,715	40.74	7,662	41.52
TrEMBL	30,964	97.66	20,489	97.82	26,828	92.58	18,477	97.57	18,193	98.60
Interpro	22,744	71.73	18,911	90.28	25,401	87.65	15,537	82.05	15,134	82.02
GO	18,894	59.59	13,811	65.94	15,182	52.39	11,505	60.75	11,877	64.37
Overall	31,074	98.00	20,574	98.22	27,118	93.58	18,573	98.08	18,236	98.83

Unannotated	633	2.00	372	1.78	1,861	6.86	364	1.92	216	1.17

Table 8: Orthologs of nitrogen fixation genes in *Vigna subterranea, Lablab purpureus, Faidherbia albida, Moringa oleifera and Sclerocarya birrea*

Gene	*V. subterranea*	*L. purpureus*	*F. albida*	*M. oleifera*	*S. birrea*
MtLYK3/LjNFR1	Vigsu176S22567_VIGSU	Labpu216S12485_LABPU	Faial2789S13350_FAIAL	——	——
MtNFP/LjNFR5	Vigsu1898S04417_VIGSU	Labpu54S03611_LABPU	——	——	Sclbi409S02347_SCLBI
MtDMI2/LjSYMRK	Vigsu107959S16599_VIGSU	Labpu4785S15752_LABPU	Faial1833S08172_FAIAL	Morol36160S02362_MOROL	Sclbi59955S15146_SCLBI
LjCASTOR	Vigsu108012S17109_VIGSU	Labpu27S13484_LABPU	——	——	———
MtHMGR1	——	——	——	——	———
MtDMI1/LjPOLLUX	Vigsu108496S19983_VIGSU	Labpu4332S15101_LABPU	Faial363S16033_FAIAL	Morol36085S07630_MOROL	———
NSP1	Vigsu2922S08781_VIGSU	Labpu723S04373_LABPU	Faial1104S01086_FAIAL	Morol36102S01150_MOROL	Sclbi5005S02593_SCLBI

NSP2	Vigsu107793S01507_VIGSU	Labpu887S08157_LABPU	Faial757S23006_FAIAL	Morol36224S03158_MOROL	Sclbi2944S01716_SCLBI
CCaMK	Vigsu91S05737_VIGSU	——	Faial752S22546_FAIAL	——	———
MtIPD3/LjCYCLOPS	Vigsu104856S09608_VIGSU	Labpu701S17462_LABPU	——	——	Sclbi2578S10386_SCLBI
NIN	Vigsu273S23676_VIGSU	Labpu165S10337_LABPU	Faial788S23538_FAIAL	Morol36195S02810_MOROL	Sclbi2838S04948_SCLBI
MtCRE1/LjLHK1	——	Labpu2293S02028_LABPU	Faial1226S02883_FAIAL	——	——
NF-YA1	Vigsu107799S13964_VIGSU	Labpu193775S11413_LABPU	Faial246S12019_FAIAL	Morol36154S02289_MOROL	Sclbi406S12278_SCLBI
NF-YA2	——	——	Faial858S26716_FAIAL	——	———
MtERN1	Vigsu107612S00570_VIGSU	Labpu210S01798_LABPU	Faial719S21851_FAIAL	Morol36040S00658_MOROL	Sclbi1920S01196_SCLBI
MtERN2	Vigsu108137S07511_VIGSU	Labpu448S03276_LABPU	Faial4604S17896_FAIAL	——	———

Figure legends

Figure 1: Phylogenetic and evolutionary analysis.

Scale bar = 10 million years. Values at branch points indicate estimates of divergence time (million years ago, Mya); blue numbers show divergence time (Mya); red nodes indicate previously published calibration times. *V. sub* shows seeds of *Vigna subterranean*; *L. pur*, flowers of *Lablab purpureus*; *F. alb*, seed pods of *Faidherbia albida*; *S. bir*, fruit of *Sclerocarya birrea*; *M. ole*, flowers of *Moringa oleifera*.

Figure 2: The groups of orthologs shared by the orphan crops.

(A) Groups of orthologs shared between *Lablab purpureus* (*L. pur*), *Faidherbia albida* (*F. alb*), *Glycine max* (*G. max*), *Medicago truncatula* (*M. tru*) and *Vigna subterranea* (*V.sub*). (B) Groups of orthologs shared between *Sclerocarya birrea* (*S. bir*), *Moringa oleifera* (*M. ole*), *Carica papaya* (*C. pap*), *Citrus sinensis* (*C. sin*) and *Theobroma cacao* (*T. cac*). Venn diagram generated using [85].

Figure 3: The common symbiosis signaling pathway among the orphan crops.

Sixteen root nodulation symbiosis signal (Sym) pathway genes were identified in three legumes (*Vigna subterranea*, *Lablab purpureus* and *Faidherbia albida*) and two non-legumes (*Sclerocarya birrea* and *Moringa oleifera*). Lj, *Lotus japonicas*; Mt, *Medicago truncatula*; LCOs, Lipochitooligosaccharides.

Figure 4: Percentages of transcription factors in five orphan species.

Blastp was used to search against 58 plant transcription factor families obtained from PlantTFDB [69] (see Additional file 2: Table S14). In this figure, MADS includes M-type_MADS and MIKC_MADS. MYB includes MYB and MYB_related. NF-YA/B/C

includes NF-YA, NF-YB and NT-YC. “Others” comprises 31 types of transcription factors (E2F/DP, Nin-like, TALE, YABBY, GeBP, BES1, DBB, CO-like, CPP, SBP, STAT, WOX, BBR-BPC, CAMTA, AP2, ZF-HD, S1Fa-like, ARR-B, SRS, GRF, LSD, NF-X1, EIL, RAV, HRT-like, HB-PHD, VOZ, Whirly, SAP, LFY and NZZ/SPL) whose percentage was less than 1%.

Figure 5: Identification of genes involved in the starch biosynthesis pathway. Genes identified as being involved in starch synthesis are shown in red. Numbers of homolog genes are presented in Additional file 2: Table S11. AGP, ADP-glucose pyrophosphorylase; AGPL, AGP large subunit; AGPS, AGP small subunit; PHOH, starch phosphorylase H (cytosolic type); GBSS, granule-bound starch synthase; SS, soluble starch synthase; BE, starch branching enzyme; ISA, isoamylase; DPE, starch debranching enzyme.

Click here to access/download;Figure;Figure1.pdf

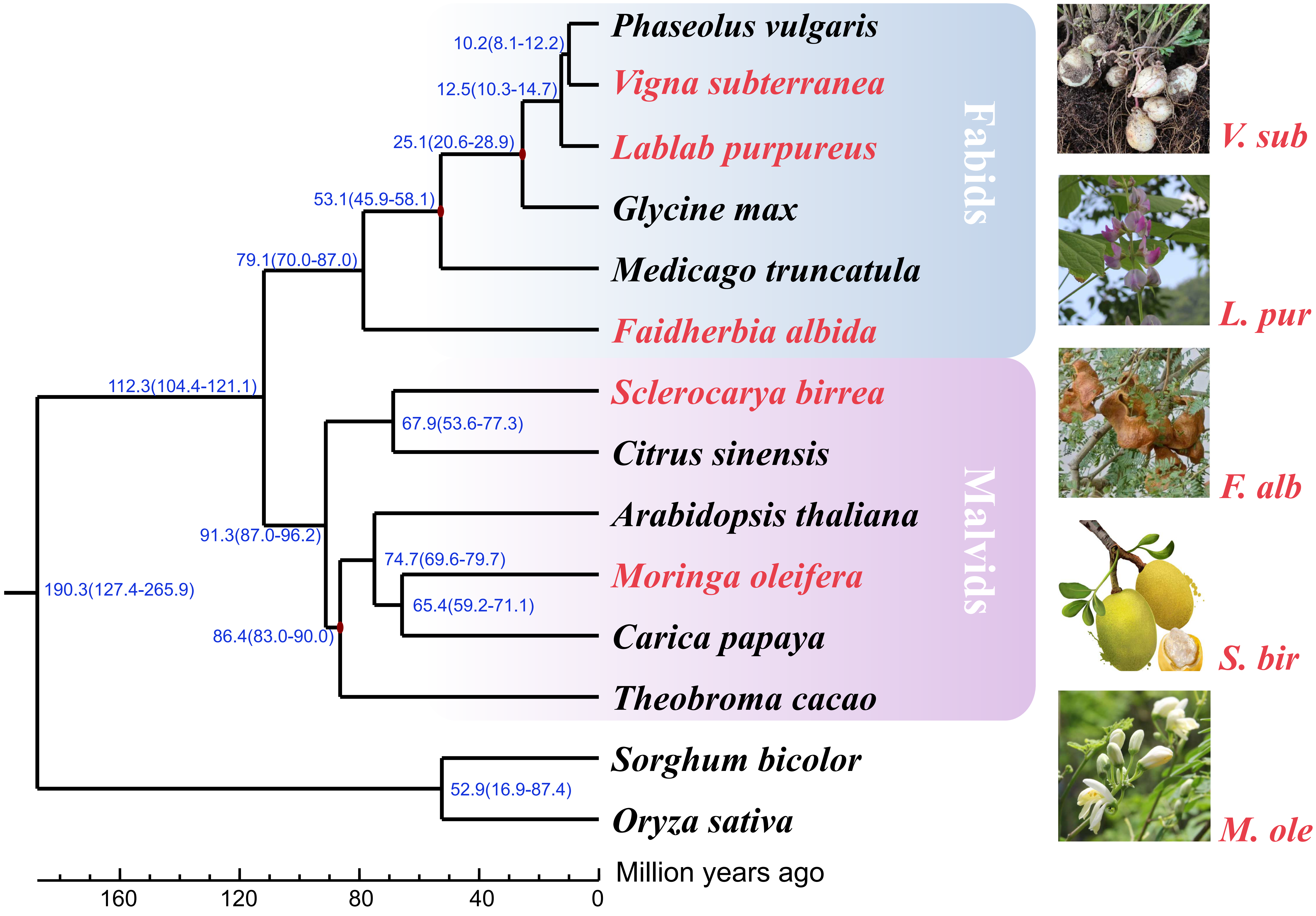

Figure 3 Click here to access/download;Figure;Figure3.pdf

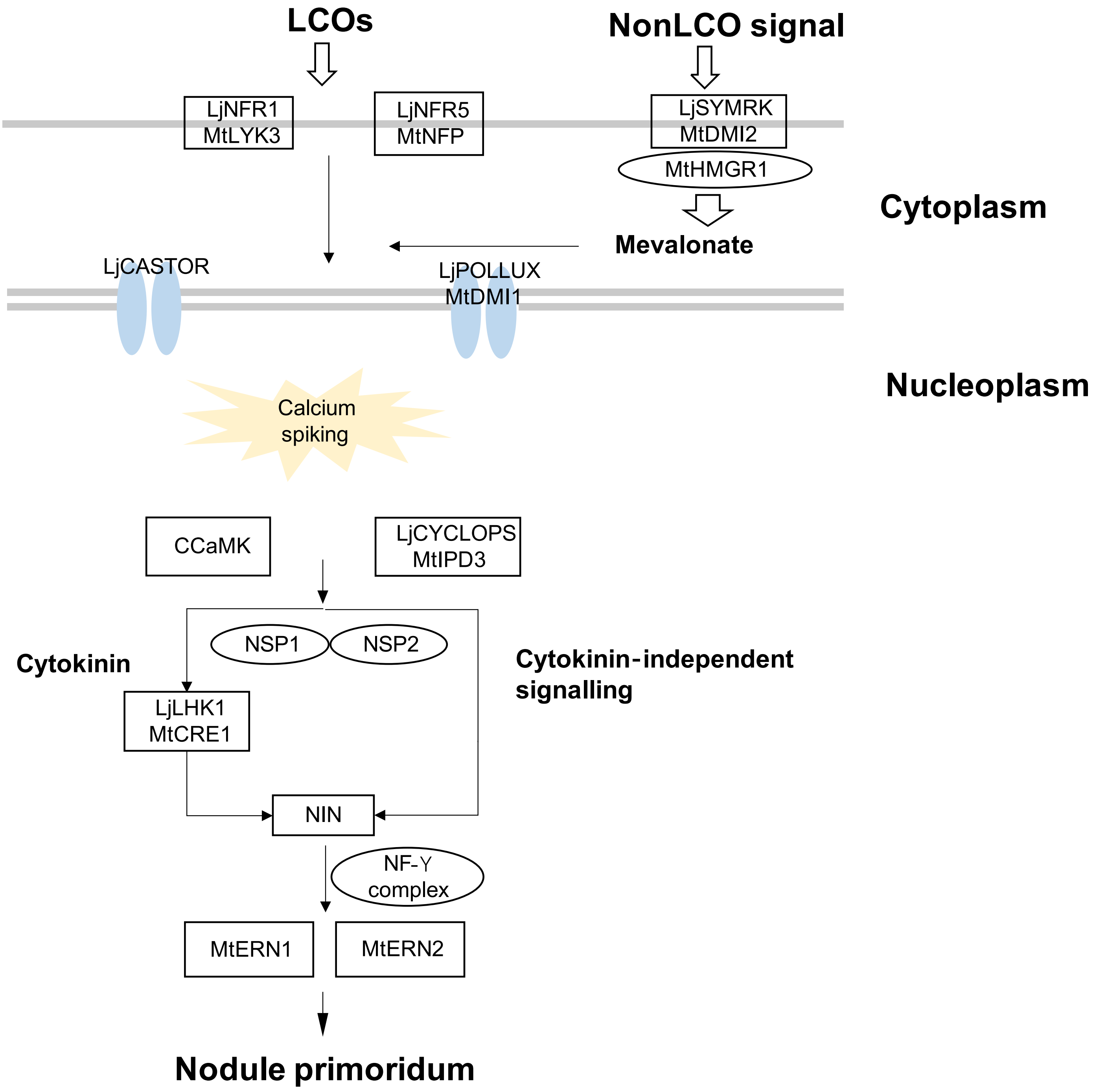

Figure 2

Click here to access/download;Figure;Figure2.pdf

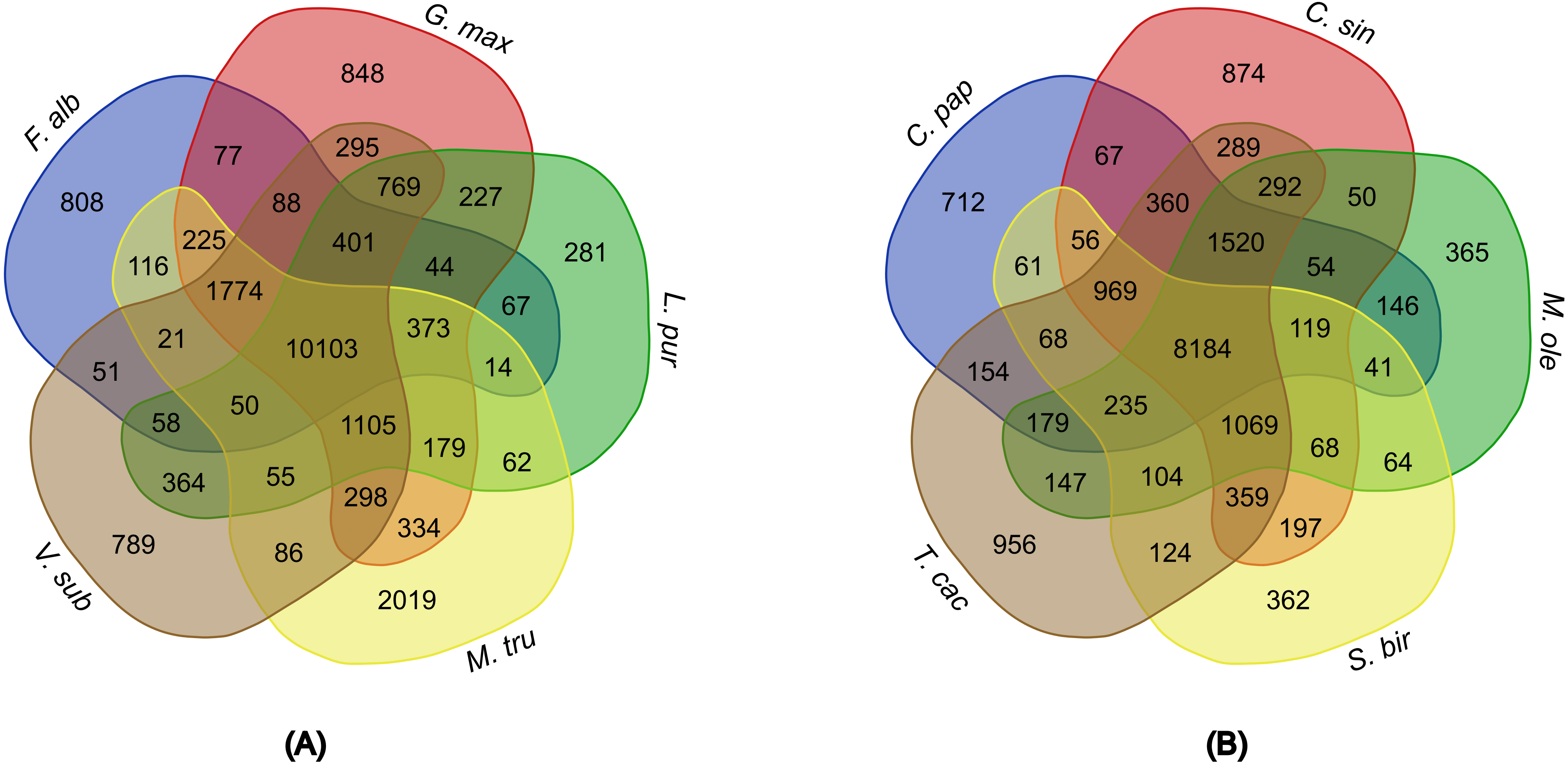

Figure 4
Click here to access/download;Figure;Figure4.pdf

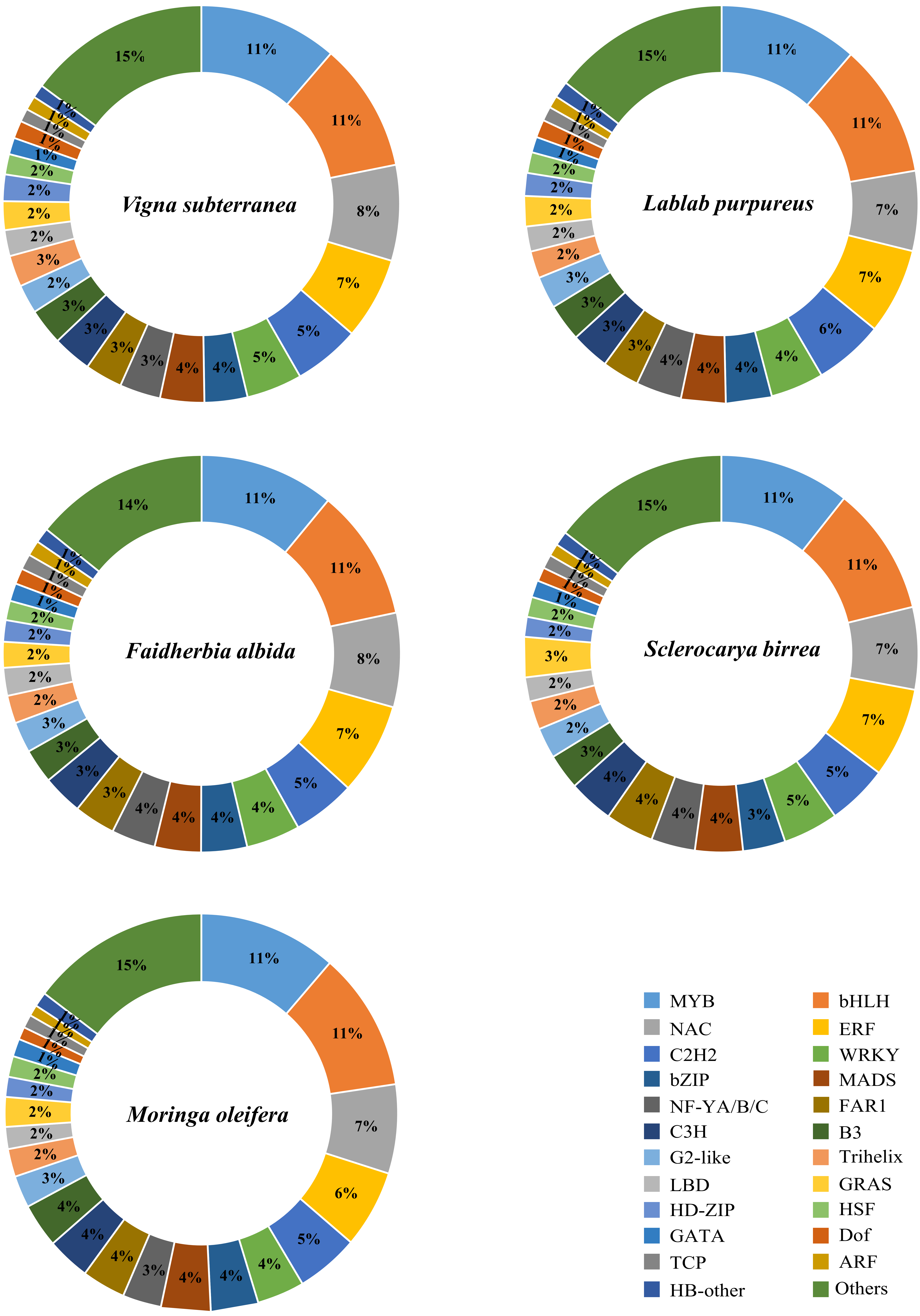

Figure 5

Click here to
access/download;Figure;Figure5.pdf

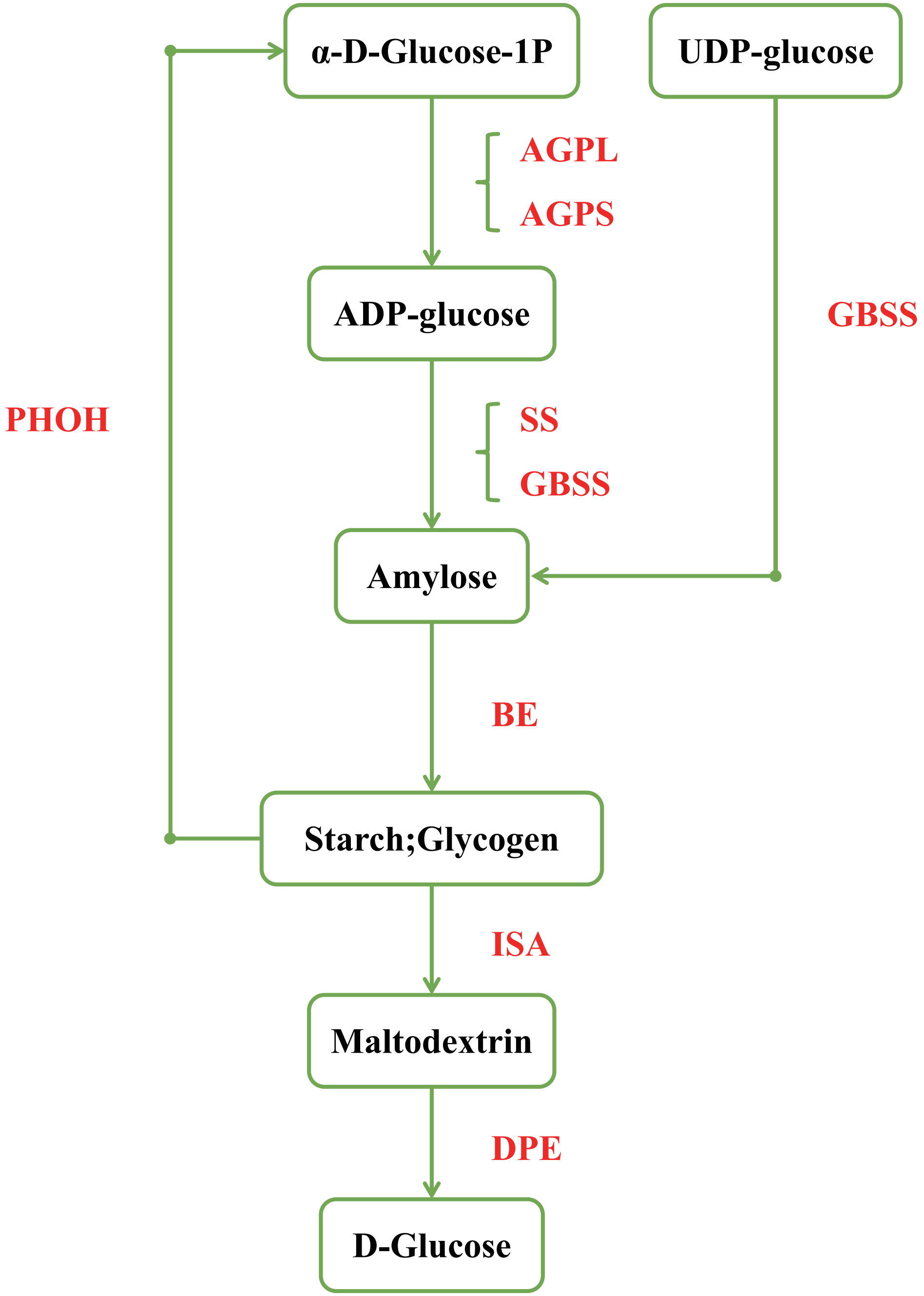

Click here to access/download
Supplementary Material
Additional file 1_clean.docx

Supplementary Material 2

Click here to access/download
Supplementary Material
Additional file 2.xlsx

Click here to access/download;Personal Cover;Response_to_Reviewer_comments.docx

Dear Dr. Scott,

Sub: **Submission of the re-revised manuscript GIGA-D-18-00275R1**

We are glad to resubmit the thoroughly revised and cleaned version of our manuscript entitled "The draft genomes of five agriculturally important African orphan crops", for possible publication in GigaScience as "Data Note".

The corrections made and suggested by Dr. Lisa Martin were highly useful to significantly improve the quality of our manuscript. We have carefully implemented all the corrections suggested by her. Now we strongly believe that the revised manuscript is now ready for publication in GigaScience.

We look forward to hearing from you at your earliest convenience.

Yours sincerely,

Xin Liu

Reviewer reports:

Reviewer #1: The revisions in the last round satisfactorily addressed my concerns.

Reviewer #2: The revised version is well written and can be accepted for publication.

Response: We thank all the reviewers for their valuable suggestions in the earlier version of the manuscript, and for giving their kind acceptance to our manuscript.
