# Supplementary material for: The draft genomes of five agriculturally important African orphan crops
Source: Gigascience. 2018 Dec 7;8(3):giy152. doi: 10.1093/gigascience/giy152 (PMC6405277; doi:10.1093/gigascience/giy152)
Supplement: Reviewer_3_Original_Submission_(Attachment).docx [file gigascience_8_3_giy152_s15.docx]

The manuscript entitled, “The draft genomes of five agriculturally important African orphan crops” is an excellent report on the exceptionally significant research work done by the authors. The authors have done an estimable job by sequencing the genome and several transcriptomes from five important crops. The project generated huge data; however the manuscript describes only the preliminary analysis. More deep analysis and further studies are needed to apply this knowledge in crop improvement programme. However, it establishes a strong platform for further research and breeding programmes. I strongly recommend the publication of this manuscript with some minor revisions. Please find the following comments.

1. The plants sequenced in this project have smaller genome size compared to many other sequenced crops, and repeat elements are also comparatively low. However none of the assemblies are complete and couldn’t assemble into the chromosome level. If the authors have used long insert libraries also, it would have been better
2. “Various gene structure parameters were compared to the related species of each sequenced genome as summarized in table 5”- The number of protein coding genes in these sequenced genome seems to be less compared to the related species. Can the authors provide an explanation for this?
3. Figure S5 is not provided
4. 633, 372, 861, 364 and 216 genes are unannotated in V. subterranea L. purpureus F. albida S. birrea and M. oleifera respectively. Are these genes specific to the respective genomes?
5. “Furthermore, the 10,103 gene families of *V. subterranea*, *L. purpureus*, *F. albida*,

*M. truncatula* and *G. max* were clustered (Figure 2A). There were 1,105 orthologous

families shared by the four Papilionoideae species, while 808 gene families containing

1,966 genes were specific to *F. albida*, 281 gene families containing 538 genes were

specific to *L. purpureus,* 789 gene families containing 3,118 genes were specific to *V.*

*subterranea.*

Moreover, 8,184 gene families of *S. birrea*, *M. oleifera*, *C. papaya*, *C. sinensis* and

*T. cacao* were clustered (Figure 2B), of which 365 gene families containing 798 genes

were specific to *M. oleifera*, 362 gene families containing 796 genes were specific to *S.*

*birrea,* respectively”*.*

-To which class the specific genes mostly belong in the functional annotation?

6. In the phylogenetic analysis with 141 single-copy genes from 14 species, Populus trichocarpa

clusters with other members in Fabids. But in some other phylogenetic analysis constructed

using the same criteria, the group malpigiales, which includes Populus trichocarpa clusters with

malvids or as a separate group. How do the authors explain this?

Ref:

Rahman, A. Y. A., Usharraj, A. O., Misra, B. B., Thottathil, G. P., Jayasekaran, K., Feng, Y., ... & Tan,

H. S. (2013). Draft genome sequence of the rubber tree Hevea brasiliensis. *BMC genomics*, *14*(1),

75.

Shulaev, V., Sargent, D. J., Crowhurst, R. N., Mockler, T. C., Folkerts, O., Delcher, A. L., ... & Burns,

P. (2011). The genome of woodland strawberry (Fragaria vesca). *Nature genetics*, *43*(2), 109.

https://genomevolution.org/wiki/index.php/Sequenced_plant_genomes
