# Supplementary material for: The draft genomes of five agriculturally important African orphan crops
Source: Gigascience. 2018 Dec 7;8(3):giy152. doi: 10.1093/gigascience/giy152 (PMC6405277; doi:10.1093/gigascience/giy152)
Supplement: giy152_Supplementary_Files [file giy152_supplementary_files.zip › Supplementary Material Figures S1-S6; Tables S1-S13.docx]

**
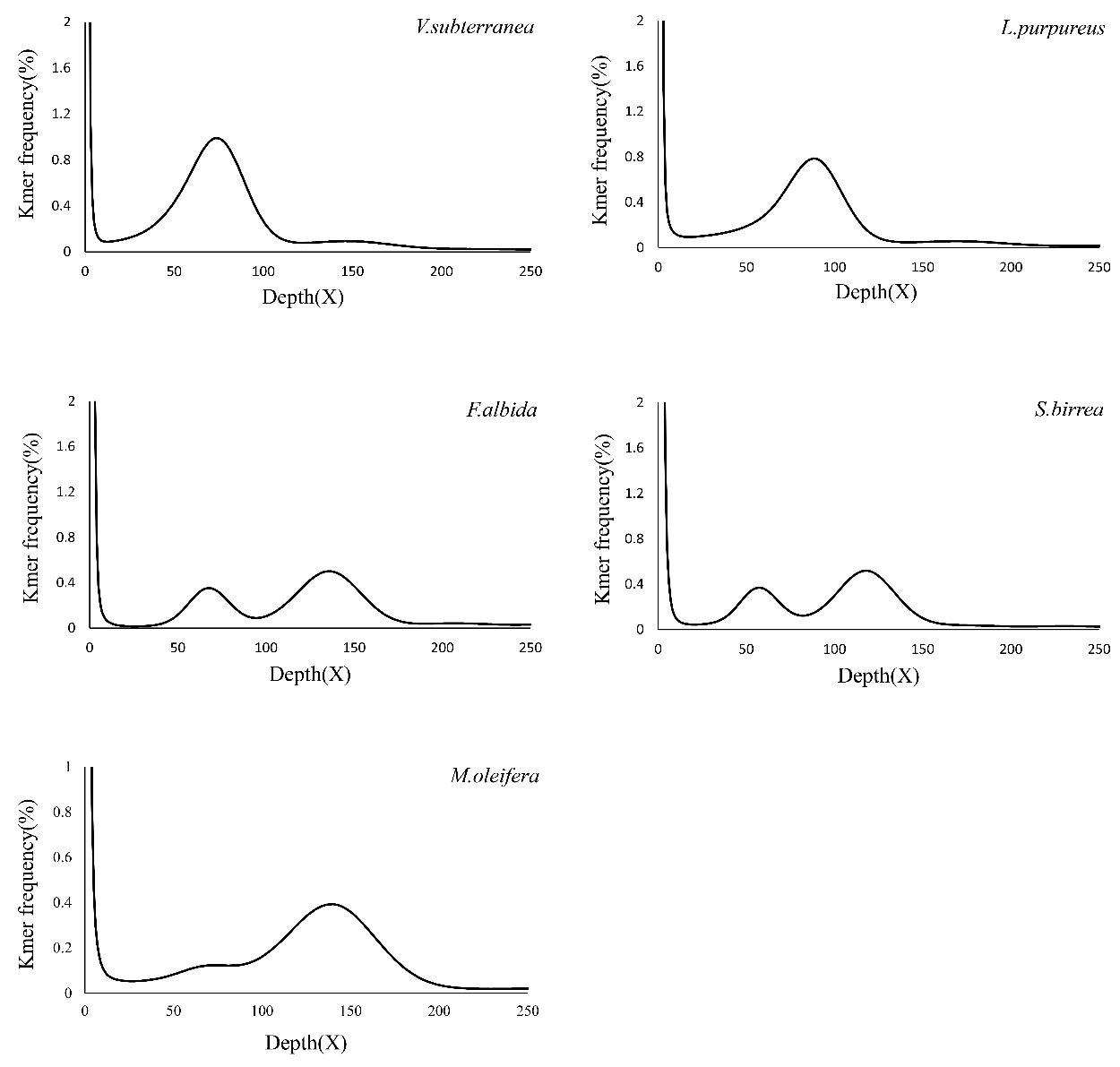
**

**Figure S1:** **K-mer (K=17) analysis of five genomes.** The X-axis is depth; the y-axis represents the frequency.


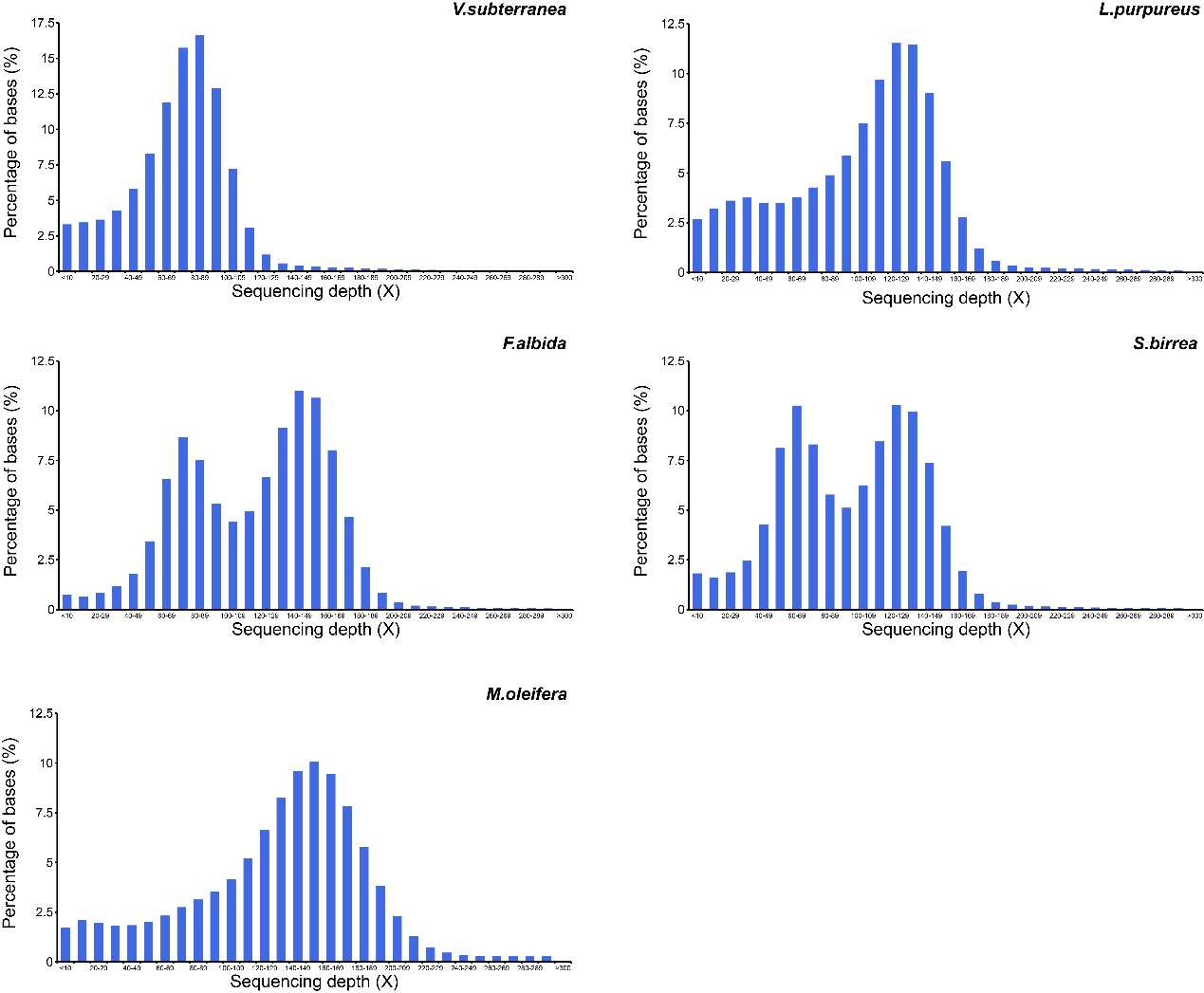


**Figure S2: Distribution of sequencing depth of the assembly data.** The X-axis is the depth, and the y-axis shows the corresponding percentage of bases. The result shows that <2.5% of bases had a sequencing depth less than 10X in *F.albida* and *S.birrea* and *M.oleifera*, >2.5% of bases had a sequencing depth less than 10X in *V. subterranea* *L. purpureus* and, and two peaks demonstrate the genome heterozygosity.

**
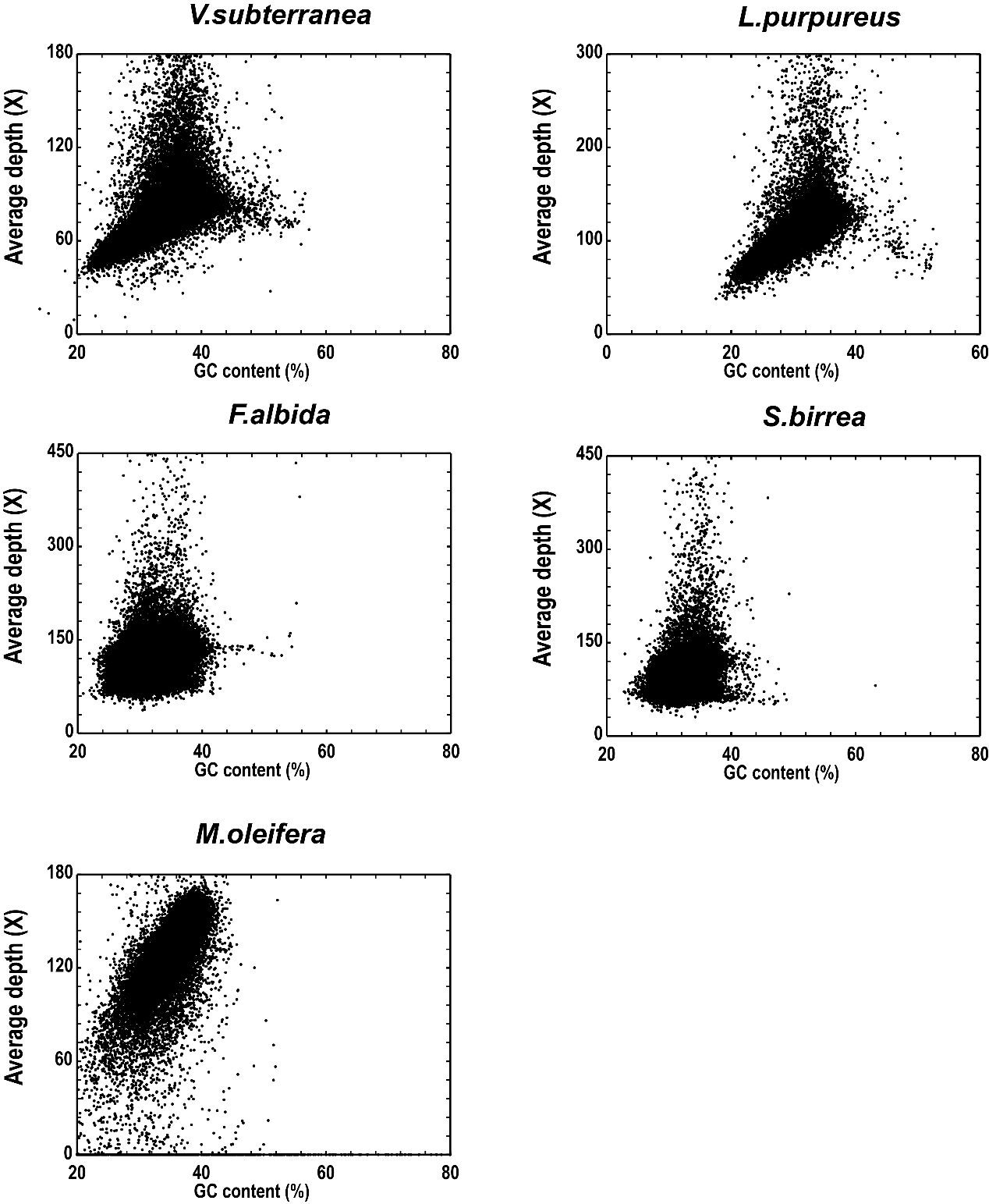
**

**Figure S3: The GC content.** The GC content and the average depth were calculated from 10 kb non-overlapping sliding windows. The distribution pattern of GC content indicates a relative pure single genomic sample without contamination and no GC bias.


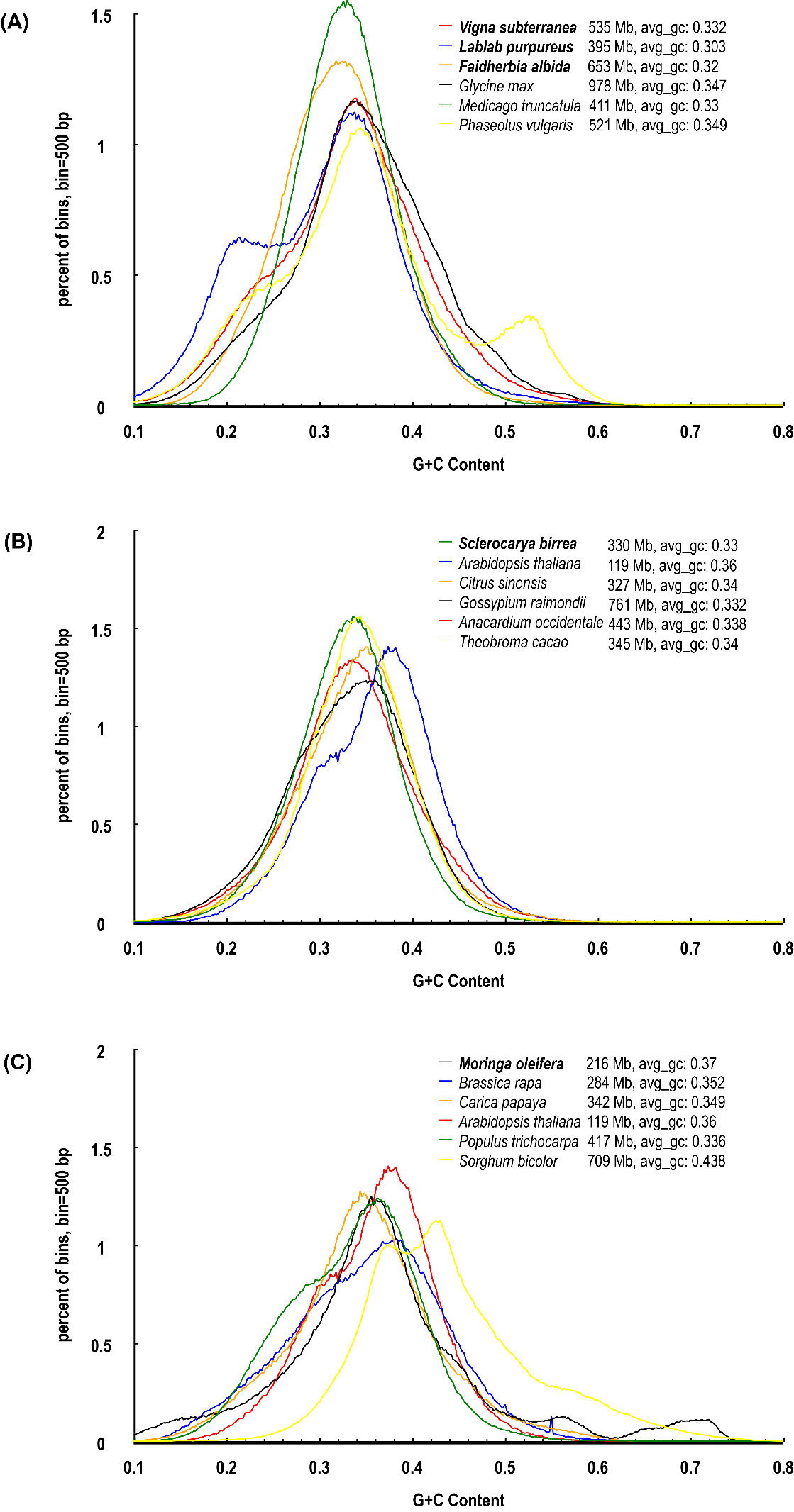


**Figure S4: Comparison of GC content across closely related species.** (A)showed the GC content between *Vigna subterranea*, *Lablab purpureus*, *Faidherbia albida* and related species. (B) showed the GC content of *Sclerocarya birrea* and related species. (C) showed the GC content of *Moringa oleifera* and related species.

**
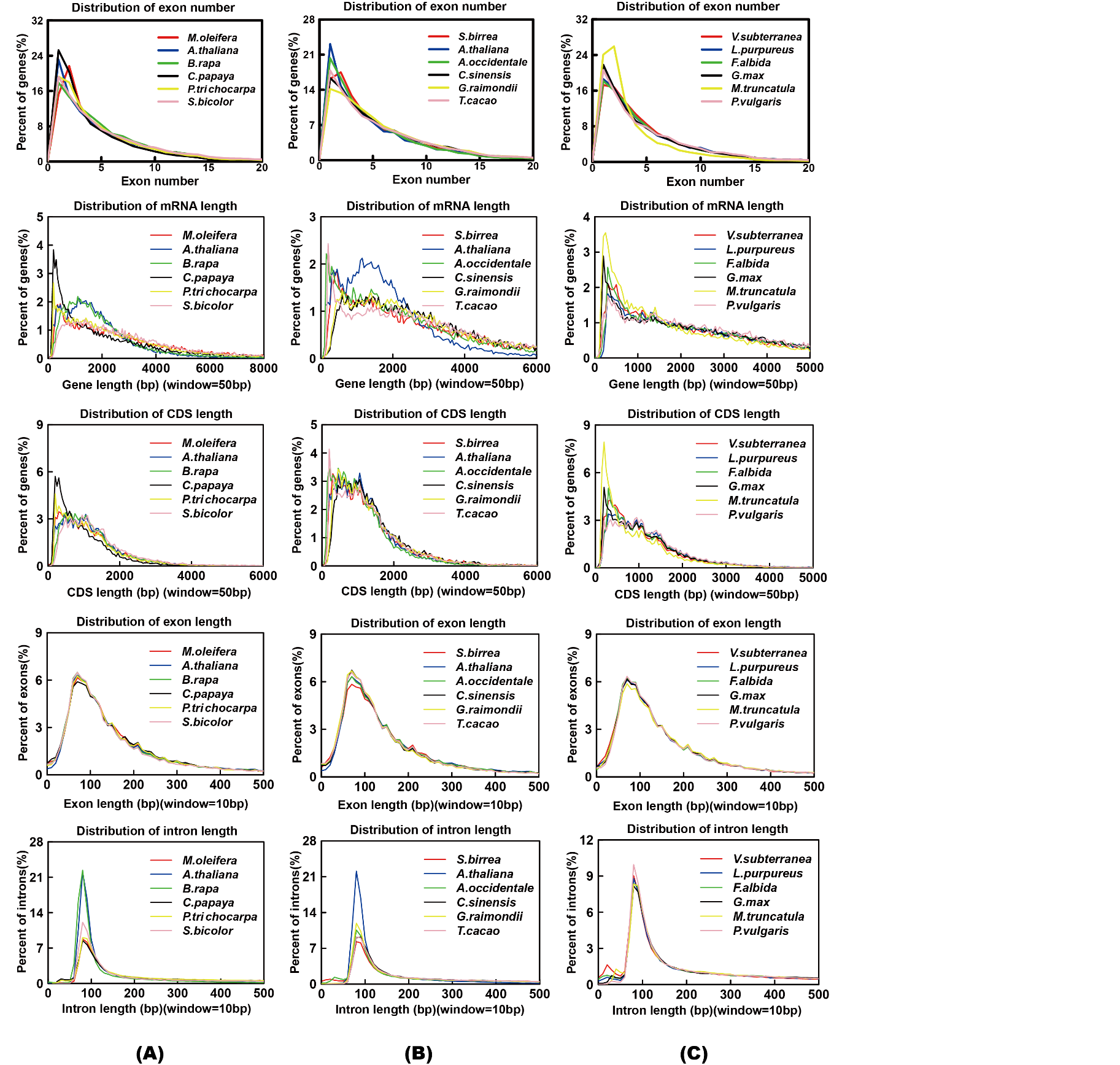
**

**Figure S5: Statistics of gene models in *V. subterranea, L. purpureus, F. albida, M. oleifera, S. birrea*.** (A) showed the gene models in *Moringa oleifera*, (B) showed the gene models in *Sclerocarya birrea*, (C) showed the gene models in *Vigna subterranea*, *Lablab purpureus*, *Faidherbia albida*.

**
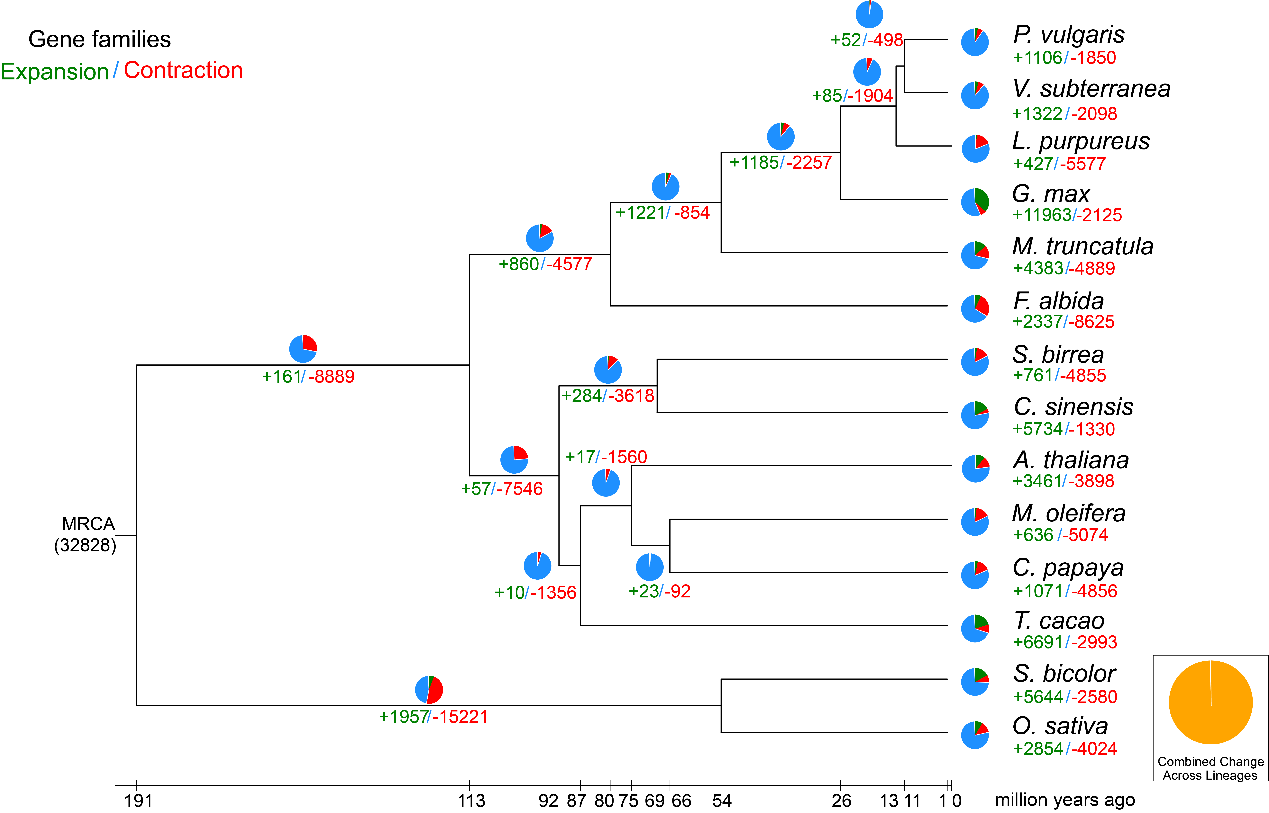
**

**Figure S6: Expansion and contraction of gene families.** Gene family with expansions are indicated in green, and gene family contractions are indicated in red; the proportions among total changes are shown using the same colors in the pie charts. The blue portions of the pie charts represent the conserved gene families. MRCA is the most recent common ancestor.

**Table S1. Statistics of the raw and clean data of DNA sequencing.** Clean data were obtained by filtering raw data as described in the article. The sequencing depth calculated based on a genome size (*V. subterranea* 550Mb, *L.purpureus* 423Mb, *F.albida* 661Mb, *S.birrea* 356Mb, *M.oleifera* 278Mb ).

| Species | Library insert size (bp) | | Read length (bp) | Raw data | | | | | | Clean data | | | | | | | | | Accession  number |
| --- | --- | --- | --- | --- | --- | --- | --- | --- | --- | --- | --- | --- | --- | --- | --- | --- | --- | --- | --- |
|  |  |  |  | **Base number (bp)** | | **Reads number (bp)** | | **Depth(X)** | | | **Base number (bp)** | | **Reads number (bp)** | | **Depth(X)** | | | |  |
| ***V.*** ***subterranea*** | 250 | | PE150 | 49,086,002,100 | | 327,240,014 | | 89.25 | | | 42,847,600,630 | | 295,500,694 | | 77.90 | | | | SRX4171803 |
|  | 500 | | PE100 | 12,031,550,800 | | 120,315,508 | | 21.88 | | | 10,277,685,010 | | 108,186,158 | | 18.69 | | | | SRX4171792 |
|  | 2000 | | MP100 | 24,173,576,400 | | 241,735,764 | | 43.95 | | | 10,453,947,630 | | 110,041,554 | | 19.01 | | | | SRX4171804 |
|  | 6000 | | MP100 | 28,009,055,200 | | 280,090,552 | | 50.93 | | | 10,980,195,000 | | 115,581,000 | | 19.96 | | | | SRX4171789 |
|  | 20000 | | MP75 | 24,204,456,300 | | 322,726,084 | | 44.01 | | | 7,454,810,440 | | 106,497,292 | | 13.55 | | | | SRX4171790 |
|  | **Total** | | **/** | **137,504,640,800** | | **1,292,107,922** | | **250.01** | | | **82,014,238,710** | | **735,806,698** | | **149.12** | | | |  |
| ***L.***  ***purpureus*** | 250 | | PE150 | 60,287,284,200 | | 401,915,228 | | 142.52 | | | 42,480,817,860 | | 326,775,522 | | 100.43 | | | | SRX4171774 |
|  | 500 | | PE100 | 16,485,233,200 | | 164,852,332 | | 38.97 | | | 13,338,245,290 | | 140,402,582 | | 31.53 | | | | SRX4171771 |
|  | 2000 | | MP100 | 16,558,154,200 | | 165,581,542 | | 39.14 | | | 7,821,848,560 | | 82,335,248 | | 18.49 | | | | SRX4171773 |
|  | 6000 | | MP100 | 29,323,124,600 | | 293,231,246 | | 69.32 | | | 11,841,757,220 | | 124,650,076 | | 27.99 | | | | SRX4171778 |
|  | 10000 | | MP49 | 19,274,553,172 | | 393,358,228 | | 45.57 | | | 1,980,366,256 | | 45,008,324 | | 4.68 | | | | SRX4171772 |
|  | **Total** | | **/** | **141,928,349,372** | | **1,418,938,576** | | **335.53** | | | **77,463,035,186** | | **719,171,752** | | **183.13** | | | |  |
| ***F.albida*** | 170 | PE100 | | | 33,121,301,800 | | 331,213,018 | | 50.11 | | | 28,703,837,700 | | 302,145,660 | | 43.42 | | SRX4003254 | |
|  | 250 | PE150 | | | 54,564,056,100 | | 363,760,374 | | 82.55 | | | 45,860,616,240 | | 316,280,112 | | 69.38 | | SRX4003257 | |
|  | 350 | PE100 | | | 26,516,538,200 | | 265,165,382 | | 40.12 | | | 23,136,560,300 | | 243,542,740 | | 35.00 | | SRX4003253 | |
|  | 500 | PE100 | | | 37,470,276,400 | | 374,702,764 | | 56.69 | | | 29,615,711,160 | | 311,744,328 | | 44.80 | | SRX4003252 | |
|  | 800 | PE100 | | | 26,368,901,400 | | 263,689,014 | | 39.89 | | | 20,376,553,800 | | 214,490,040 | | 30.83 | | SRX4003251 | |
|  | 2000 | MP100 | | | 38,010,750,800 | | 380,107,508 | | 57.50 | | | 13,971,852,350 | | 147,072,130 | | 21.14 | | SRX4003256 | |
|  | 10000 | MP100 | | | 21,485,522,000 | | 214,855,220 | | 32.50 | | | 2,813,237,470 | | 29,613,026 | | 4.26 | | SRX4003255 | |
|  | **Total** | **/** | | | **237,537,346,700** | | **2,193,493,280** | | **359.36** | | | **164,478,369,020** | | **1,564,888,036** | | **248.83** |  | | |
| ***S.birrea*** | 170 | | PE100 | 33,105,136,400 | | 331,051,364 | | 92.99 | | | 26,550,586,320 | | 279,479,856 | | 74.58 | | | | SRX4171798 |
|  | 250 | | PE150 | 52,779,049,800 | | 351,860,332 | | 148.26 | | | 43,837,202,720 | | 302,325,536 | | 123.14 | | | | SRX4171802 |
|  | 350 | | PE100 | 40,081,173,400 | | 400,811,734 | | 112.59 | | | 32,814,989,680 | | 345,420,944 | | 92.18 | | | | SRX4171795 |
|  | 500 | | PE100 | 32,177,818,400 | | 321,778,184 | | 90.39 | | | 26,521,782,700 | | 279,176,660 | | 74.50 | | | | SRX4171796 |
|  | 800 | | PE100 | 28,800,733,400 | | 288,007,334 | | 80.90 | | | 23,567,139,060 | | 248,075,148 | | 66.20 | | | | SRX4171801 |
|  | 2000 | | MP100 | 33,418,800,000 | | 334,188,000 | | 93.87 | | | 8,543,561,280 | | 89,932,224 | | 24.00 | | | | SRX4171800 |
|  | 10000 | | MP100 | 22,862,981,800 | | 228,629,818 | | 64.22 | | | 5,613,268,420 | | 59,087,036 | | 15.77 | | | | SRX4171799 |
|  | **Total** | | **/** | **243,225,693,200** | | **2,256,326,766** | | **683.22** | | | **167,448,530,180** | | **1,603,497,404** | | **470.36** | | | |  |
| ***M.oleifera*** | 250 | | PE150 | 57,094,362,000 | | 380,629,080 | | 205.38 | | | 43,801,961,630 | | 302,082,494 | | 157.56 | | | | SRX4171769 |
|  | 500 | | PE150 | 47,503,842,900 | | 316,692,286 | | 170.88 | | | 36,852,547,210 | | 254,155,498 | | 132.56 | | | | SRX4171788 |
|  | 2000 | | MP100 | 59,470,238,400 | | 594,702,384 | | 213.92 | | | 24,641,039,770 | | 259,379,366 | | 88.64 | | | | SRX4171787  SRX4171786 |
|  | 10000 | | MP100 | 27,070,800,800 | | 270,708,008 | | 97.38 | | | 5,461,131,620 | | 57,485,596 | | 19.64 | | | | SRX4171785 |
|  | **Total** | | **/** | **191,139,244,100** | | **1,562,731,758** | | **687.55** | | | **110,756,680,230** | | **873,102,954** | | **398.41** | | | |  |

**Table S2. Summary statistics of the transcriptome data in four species.**

| Species | Abbreviation | Raw data | | Clean data | | SSR  number | Sample |
| --- | --- | --- | --- | --- | --- | --- | --- |
|  |  | **Base number (bp)** | **Reads number (bp)** | **Base number (bp)** | **Reads number (bp)** |  |  |
| ***V.*** ***subterranea*** | VsSL | 15,342,299,786 | 126,795,866 | 4,666,457,080 | 44,023,180 | SRX4171791 | Semi mature leaf |
|  | VsWST | 11,833,035,764 | 97,793,684 | 3,280,636,400 | 30,949,400 | SRX4171794 | whole stem |
|  | VsYL | 8,936,443,868 | 73,854,908 | 2,558,726,156 | 24,138,926 | SRX4171793 | Young leaf |
|  | **Total** | **36,111,779,418** | **298,444,458** | **10,505,819,636** | **99,111,506** |  | **/** |
| ***L.***  ***purpureus*** | LpPt | 5,654,761,970 | 46,733,570 | 793,784,604 | 7,488,534 | SRX4171777 | petal |
|  | LpST | 12,103,473,184 | 100,028,704 | 1,672,179,680 | 15,775,280 | SRX4171776 | stem |
|  | LpSp | 8,928,599,438 | 73,790,078 | 1,060,903,968 | 10,008,528 | SRX4171775 | sepal |
|  | LpYL | 13,335,065,876 | 110,207,156 | 1,568,303,496 | 14,795,316 | SRX4171770 | Young leaf |
|  | **Total** | **40,021,900,468** | **330,759,508** | **5,095,171,748** | **48,067,658** |  | / |
| ***F.albida*** | FADS | 6,081,875,514 | 50,263,434 | 226,528,572 | 2,137,062 | SRX4003248 | Dry seed |
|  | FALB | 10,174,491,668 | 84,086,708 | 373,128,692 | 3,520,082 | SRX4003247 | Leaf bud |
|  | FAS | 5,910,837,658 | 48,849,898 | 247,319,412 | 2,333,202 | SRX4003250 | Stem |
|  | FAYL | 14,731,623,434 | 121,748,954 | 1,571,678,748 | 14,827,158 | SRX4003249 | Young leaf |
|  | **Total** | **36,898,828,274** | **304,948,994** | **2,418,655,424** | **22,817,504** |  | / |
| ***M.oleifera*** | MODS | 5,108,725,754 | 42,220,874 | 62,276,640 | 541,536 | SRX4171784 | Dry seed |
|  | MOFB | 155,602,612 | 1,285,972 | 655,960 | 5,704 | SRX4171783 | Flower bud |
|  | MOGS | 6,324,766,558 | 52,270,798 | 99,341,600 | 863,840 | SRX4171782 | Germinated seed |
|  | MOML | 11,289,800,456 | 93,304,136 | 1,012,977,960 | 8,808,504 | SRX4171781 | Mature leaf |
|  | MOSL | 12,495,191,566 | 103,266,046 | 202,225,430 | 1,758,482 | SRX4171780 | Semi mature leaf |
|  | MOST | 9,684,253,634 | 80,035,154 | 799,290,020 | 6,950,348 | SRX4171779 | stem |
|  | MOYB | 3,790,455,680 | 31,326,080 | 58,032,220 | 504,628 | SRX4171797 | Young bark |
|  | **Total** | **48,848,796,260** | **403,709,060** | **2,234,799,830** | **19,433,042** |  | / |

**Table S3. Estimation of genome size based on K-mer statistics in five species.**

| **Species** | **kmer value** | **kmer number** | **peak depth(X)** | **genome size  (Mb)** | **used bases  (Gb)** | **used reads  (Mb)** | **Depth  (X)** |
| --- | --- | --- | --- | --- | --- | --- | --- |
| *V.subterranea* | 17 | 40,688,936,015 | 74 | 549.85 | 45.66 | 310.60 | 83.04 |
| *L. purpureus* | 17 | 37,240,013,376 | 88 | 423.18 | 42.47 | 326.67 | 100.35 |
| *F. albida* | 17 | 89,254,911,375 | 135 | 661.15 | 104.13 | 929.74 | 79.33 |
| *S.birrea* | 17 | 42,056,676,105 | 118 | 356.41 | 47.27 | 326.02 | 64.07 |
| *M.oleifera* | 17 | 38,954,140,320 | 140 | 278.24 | 43.79 | 301.97 | 80.93 |

**Table S4. BUSCO evaluation of the annotated protein-coding genes in five species*.***

| **BUSCOs** | ***V. subterranea*** | | ***L. purpureus*** | | ***F. albida*** | | ***S. birrea*** | | M. oleifera | |
| --- | --- | --- | --- | --- | --- | --- | --- | --- | --- | --- |
|  | **NO.** | **P,%** | **NO.** | **P,%** | **NO.** | **P,%** | **NO.** | **P,%** | **NO.** | **P,%** |
| Complete single copy | 1,214 | 84.30 | 1,143 | 73.50 | 1,240 | 86.10 | 1153 | 80.10 | 1184 | 82.20 |
| Complete duplicated | 95 | 6.60 | 1,058 | 5.90 | 86 | 6.00 | 31 | 2.20 | 13 | 0.90 |
| Fragmented | 51 | 3.54 | 85 | 2.20 | 37 | 2.60 | 35 | 2.40 | 77 | 5.30 |
| Missing | 80 | 5.56 | 32 | 18.40 | 77 | 5.30 | 221 | 15.30 | 166 | 11.60 |
| Total | 1440 | / | 1440 | / | 1440 | / | 1440 | / | 1440 |  |

**Table S5. Analysis of gene families of different species.**

| Species | Genes number | Genes in families | Unclustered genes | Family number | Unique families | Average genes  per family |
| --- | --- | --- | --- | --- | --- | --- |
| *A. thaliana* | 26633 | 23282 | 3351 | 13191 | 644 | 1.76 |
| *C. papaya* | 24107 | 18320 | 5787 | 12925 | 421 | 1.42 |
| *C. sinensis* | 35182 | 33679 | 1503 | 14527 | 630 | 2.32 |
| ***F. albida*** | 28979 | 22241 | 6738 | 14270 | 499 | 1.56 |
| *G. max* | 55137 | 44516 | 10621 | 17140 | 560 | 2.6 |
| ***L. purpureus*** | 20946 | 19274 | 1672 | 14152 | 104 | 1.36 |
| *M. truncatula* | 50358 | 37895 | 12463 | 16814 | 1768 | 2.25 |
| ***M. oleifera*** | 18451 | 16196 | 2255 | 12637 | 150 | 1.28 |
| *O. sativa* | 37358 | 27452 | 9906 | 16554 | 1533 | 1.66 |
| *P. vulgaris* | 26226 | 24688 | 1538 | 16124 | 90 | 1.53 |
| ***S. birrea*** | 18937 | 16265 | 2672 | 12080 | 205 | 1.35 |
| *S. bicolor* | 38949 | 36157 | 2792 | 16110 | 1075 | 2.24 |
| *T. cacao* | 41951 | 36135 | 5816 | 15009 | 651 | 2.41 |
| ***V. subterranea*** | 31707 | 26718 | 4989 | 16307 | 609 | 1.64 |

**Table S6. Enriched pathways of unique paralogs genes in families.**

| Species | Pathway ID | KEGG description | Number of  genes | P-value  (<=0.05) |
| --- | --- | --- | --- | --- |
| *V.subterranea* | ko00908 | Zeatin biosynthesis | 36 | 1.38E-13 |
|  | ko00710 | Carbon fixation in photosynthetic organisms | 32 | 3.89E-09 |
|  | ko03420 | Nucleotide excision repair | 34 | 1.49E-07 |
|  | ko00270 | Cysteine and methionine metabolism | 33 | 4.08E-07 |
|  | ko03440 | Homologous recombination | 26 | 1.17E-06 |
|  | ko03030 | DNA replication | 31 | 1.66E-06 |
|  | ko00630 | Glyoxylate and dicarboxylate metabolism | 23 | 6.92E-06 |
|  | ko00591 | Linoleic acid metabolism | 11 | 0.000211 |
|  | ko00943 | Isoflavonoid biosynthesis | 9 | 0.000542 |
|  | ko01110 | Biosynthesis of secondary metabolites | 179 | 0.000575 |
|  | ko00620 | Pyruvate metabolism | 24 | 0.001328 |
|  | ko00900 | Terpenoid backbone biosynthesis | 16 | 0.001752 |
|  | ko00966 | Glucosinolate biosynthesis | 23 | 0.002362 |
|  | ko00130 | Ubiquinone and other terpenoid-quinone biosynthesis | 12 | 0.002831 |
|  | ko00920 | Sulfur metabolism | 12 | 0.002831 |
|  | ko00020 | Citrate cycle (TCA cycle) | 14 | 0.003039 |
|  | ko00030 | Pentose phosphate pathway | 17 | 0.003565 |
|  | ko00010 | Glycolysis / Gluconeogenesis | 27 | 0.006058 |
|  | ko00061 | Fatty acid biosynthesis | 10 | 0.007535 |
|  | ko01100 | Metabolic pathways | 278 | 0.019517 |
|  | ko00910 | Nitrogen metabolism | 9 | 0.021015 |
|  | ko03430 | Mismatch repair | 15 | 0.024229 |
|  | ko03410 | Base excision repair | 12 | 0.02518 |
|  | ko00240 | Pyrimidine metabolism | 32 | 0.031452 |
|  | ko00450 | Selenocompound metabolism | 7 | 0.031522 |
| *L. purpureus* | ko03030 | DNA replication | 28 | 2.04E-23 |
|  | ko03420 | Nucleotide excision repair | 28 | 1.19E-21 |
|  | ko03440 | Homologous recombination | 25 | 5.72E-20 |
|  | ko03430 | Mismatch repair | 24 | 5.73E-20 |
|  | ko00240 | Pyrimidine metabolism | 17 | 1.86E-07 |
|  | ko00230 | Purine metabolism | 17 | 5.61E-07 |
|  | ko03020 | RNA polymerase | 12 | 1.17E-06 |
|  | ko00062 | Fatty acid elongation | 7 | 3.74E-05 |
|  | ko03410 | Base excision repair | 6 | 0.0011 |
|  | ko04141 | Protein processing in endoplasmic reticulum | 12 | 0.017802 |
| *F. albida* | ko04626 | Plant-pathogen interaction | 103 | 2.06E-16 |
|  | ko04141 | Protein processing in endoplasmic reticulum | 76 | 7.31E-11 |
|  | ko04144 | Endocytosis | 51 | 1.77E-08 |
|  | ko00330 | Arginine and proline metabolism | 36 | 3.51E-08 |
|  | ko03040 | Spliceosome | 55 | 4.1E-07 |
|  | ko00940 | Phenylpropanoid biosynthesis | 49 | 1.51E-06 |
|  | ko03050 | Proteasome | 14 | 0.00056 |
|  | ko00460 | Cyanoamino acid metabolism | 22 | 0.00234 |
|  | ko01110 | Biosynthesis of secondary metabolites | 142 | 0.00709 |
|  | ko03430 | Mismatch repair | 13 | 0.007678 |
|  | ko02010 | ABC transporters | 15 | 0.009808 |
|  | ko03420 | Nucleotide excision repair | 16 | 0.010664 |
|  | ko03030 | DNA replication | 14 | 0.014452 |
|  | ko00360 | Phenylalanine metabolism | 18 | 0.02025 |
|  | ko03440 | Homologous recombination | 11 | 0.042941 |
| *S.birrea* | ko04626 | Plant-pathogen interaction | 59 | 2.33E-11 |
|  | ko00330 | Arginine and proline metabolism | 21 | 1.83E-09 |
|  | ko00620 | Pyruvate metabolism | 16 | 1.38E-06 |
|  | ko00500 | Starch and sucrose metabolism | 34 | 0.00015 |
|  | ko00052 | Galactose metabolism | 18 | 0.000154 |
|  | ko03020 | RNA polymerase | 13 | 0.000184 |
|  | ko00940 | Phenylpropanoid biosynthesis | 23 | 0.000516 |
|  | ko01100 | Metabolic pathways | 118 | 0.001871 |
|  | ko00061 | Fatty acid biosynthesis | 5 | 0.007009 |
|  | ko00250 | Alanine, aspartate and glutamate metabolism | 8 | 0.007712 |
|  | ko00360 | Phenylalanine metabolism | 11 | 0.019578 |
|  | ko00460 | Cyanoamino acid metabolism | 8 | 0.019911 |
|  | ko00230 | Purine metabolism | 13 | 0.045924 |
|  | ko02010 | ABC transporters | 8 | 0.046018 |
| *M.oleifera* | ko00195 | Photosynthesis | 54 | 2.49E-24 |
|  | ko01100 | Metabolic pathways | 151 | 6.1E-16 |
|  | ko00190 | Oxidative phosphorylation | 31 | 4.57E-10 |
|  | ko00061 | Fatty acid biosynthesis | 14 | 1.04E-08 |
|  | ko00640 | Propanoate metabolism | 15 | 2.02E-08 |
|  | ko00620 | Pyruvate metabolism | 16 | 2.03E-06 |
|  | ko04710 | Circadian rhythm - mammal | 10 | 8.71E-06 |
|  | ko00460 | Cyanoamino acid metabolism | 10 | 6.68E-05 |
|  | ko03020 | RNA polymerase | 15 | 0.000129 |
|  | ko03010 | Ribosome | 26 | 0.000235 |
|  | ko00904 | Diterpenoid biosynthesis | 6 | 0.001278 |
|  | ko04141 | Protein processing in endoplasmic reticulum | 20 | 0.002255 |
|  | ko00500 | Starch and sucrose metabolism | 21 | 0.006948 |
|  | ko00561 | Glycerolipid metabolism | 9 | 0.008008 |
|  | ko00562 | Inositol phosphate metabolism | 7 | 0.008678 |
|  | ko00040 | Pentose and glucuronate interconversions | 12 | 0.009397 |
|  | ko00230 | Purine metabolism | 16 | 0.010246 |
|  | ko00592 | alpha-Linolenic acid metabolism | 5 | 0.011694 |
|  | ko00240 | Pyrimidine metabolism | 15 | 0.012088 |
|  | ko04120 | Ubiquitin mediated proteolysis | 12 | 0.014412 |
|  | ko00520 | Amino sugar and nucleotide sugar metabolism | 9 | 0.014687 |
|  | ko00940 | Phenylpropanoid biosynthesis | 12 | 0.01608 |
|  | ko04070 | Phosphatidylinositol signaling system | 7 | 0.016208 |
|  | ko00380 | Tryptophan metabolism | 5 | 0.019185 |
|  | ko00710 | Carbon fixation in photosynthetic organisms | 8 | 0.034012 |
|  | ko00941 | Flavonoid biosynthesis | 6 | 0.041794 |

**Table S7. Enriched GO terms (level 3) of unique paralogs genes in families.**

| Species | GO ID | GO Term | Type | P-value | Number of  genes |
| --- | --- | --- | --- | --- | --- |
| *V.subterranea* | GO:0043167 | ion binding | MF | 1.37E-05 | 227 |
|  | GO:0009975 | cyclase activity | MF | 0.005437 | 2 |
|  | GO:0030312 | external encapsulating structure | CC | 2.42E-08 | 19 |
|  | GO:0071944 | cell periphery | CC | 0.000148 | 19 |
|  | GO:0071554 | cell wall organization or biogenesis | BP | 1.73E-06 | 19 |
| *L.purpureus* | GO:0016740 | transferase activity | MF | 1.46E-05 | 39 |
|  | GO:0061134 | peptidase regulator activity | MF | 0.000356 | 4 |
|  | GO:0004857 | enzyme inhibitor activity | MF | 0.000948 | 6 |
|  | GO:0044238 | primary metabolic process | BP | 0.001768 | 61 |
|  | GO:0071704 | organic substance metabolic process | BP | 0.002572 | 62 |
|  | GO:0009058 | biosynthetic process | BP | 0.003641 | 31 |
| *F.albida* | GO:0061134 | peptidase regulator activity | MF | 2.93E-12 | 19 |
|  | GO:0005515 | protein binding | MF | 6.94E-08 | 185 |
|  | GO:0004857 | enzyme inhibitor activity | MF | 7.54E-07 | 19 |
|  | GO:0038023 | signaling receptor activity | MF | 0.001539 | 9 |
|  | GO:0009607 | response to biotic stimulus | BP | 3.35E-05 | 10 |
|  | GO:0009405 | pathogenesis | BP | 0.001894 | 2 |
| *M.oleifera* | GO:0051184 | cofactor transporter activity | MF | 8.94E-17 | 12 |
|  | GO:0097367 | carbohydrate derivative binding | MF | 0.000706 | 5 |
|  | GO:0016787 | hydrolase activity | MF | 0.001538 | 62 |
|  | GO:0003735 | structural constituent of ribosome | MF | 0.002744 | 16 |
|  | GO:0061134 | peptidase regulator activity | MF | 0.004526 | 3 |
|  | GO:0045156 | electron transporter, transferring electrons  within the cyclic electron transport pathway of photosynthesis activity | MF | 0.007927 | 5 |
|  | GO:0031224 | intrinsic to membrane | CC | 6.14E-12 | 51 |
|  | GO:0044425 | membrane part | CC | 2.21E-11 | 59 |
|  | GO:0034357 | photosynthetic membrane | CC | 1.31E-09 | 20 |
|  | GO:0009521 | photosystem | CC | 3.47E-09 | 18 |
|  | GO:0005622 | intracellular | CC | 4.17E-05 | 21 |
|  | GO:0044424 | intracellular part | CC | 0.000101 | 63 |
|  | GO:0044464 | cell part | CC | 0.000683 | 66 |
|  | GO:0030529 | ribonucleoprotein complex | CC | 0.003146 | 17 |
|  | GO:0043234 | protein complex | CC | 0.006659 | 25 |
|  | GO:0033177 | proton-transporting two-sector ATPase complex,  proton-transporting domain | CC | 0.016826 | 4 |
|  | GO:0044085 | cellular component biogenesis | BP | 9.86E-06 | 16 |
|  | GO:0016043 | cellular component organization | BP | 0.00013 | 18 |
| *S.birrea* | GO:0016829 | lyase activity | MF | 0.002482 | 11 |
|  | GO:0044710 | single-organism metabolic process | BP | 9.19E-07 | 69 |
|  | GO:0071554 | cell wall organization or biogenesis | BP | 0.005961 | 8 |

**Table S8. Enriched GO terms (level 3) of genes in families with expansion.**

| **Species** | **GO ID** | **GO Term** | **Type** | **P-value** | **Number of genes** |
| --- | --- | --- | --- | --- | --- |
| *V.subterranea* | GO:0043167 | ion binding | MF | 3.19E-23 | 432 |
|  | GO:0009975 | cyclase activity | MF | 0.014373751 | 2 |
|  | GO:0030312 | external encapsulating structure | CC | 0.004508154 | 18 |
|  | GO:0016469 | proton-transporting two-sector ATPase complex | CC | 0.005028666 | 2 |
|  | GO:0033178 | proton-transporting two-sector ATPase complex, catalytic domain | CC | 0.013923474 | 5 |
| *L.purpureus* | GO:0004857 | enzyme inhibitor activity | MF | 2.56E-08 | 20 |
|  | GO:0045735 | nutrient reservoir activity | MF | 0.008956155 | 5 |
|  | GO:0032993 | protein-DNA complex | CC | 6.95E-07 | 12 |
|  | GO:0043228 | non-membrane-bounded organelle | CC | 0.000353963 | 34 |
|  | GO:0030312 | external encapsulating structure | CC | 0.00181643 | 10 |
|  | GO:0016043 | cellular component organization | BP | 1.35E-05 | 29 |
|  | GO:0071554 | cell wall organization or biogenesis | BP | 0.000200242 | 14 |
| *F.albida* | GO:0061134 | peptidase regulator activity | MF | 6.19E-20 | 46 |
|  | GO:0004857 | enzyme inhibitor activity | MF | 1.38E-19 | 85 |
|  | GO:0022857 | transmembrane transporter activity | MF | 3.53E-10 | 232 |
|  | GO:0005515 | protein binding | MF | 3.04E-09 | 888 |
|  | GO:0022892 | substrate-specific transporter activity | MF | 1.34E-05 | 150 |
|  | GO:0038023 | signaling receptor activity | MF | 2.64E-05 | 31 |
|  | GO:0019208 | phosphatase regulator activity | MF | 4.22E-05 | 14 |
|  | GO:0019825 | oxygen binding | MF | 0.004606648 | 6 |
|  | GO:0019239 | deaminase activity | MF | 0.005000086 | 5 |
|  | GO:0004871 | signal transducer activity | MF | 0.010089194 | 38 |
|  | GO:0016740 | transferase activity | MF | 0.013092711 | 730 |
|  | GO:0008287 | protein serine/threonine phosphatase complex | CC | 0.000117322 | 12 |
|  | GO:0005886 | plasma membrane | CC | 0.003774117 | 12 |
|  | GO:0051606 | detection of stimulus | BP | 0.000268293 | 6 |
|  | GO:0002252 | immune effector process | BP | 0.001056925 | 5 |
|  | GO:0002253 | activation of immune response | BP | 0.001056925 | 5 |
|  | GO:0002684 | positive regulation of immune system process | BP | 0.001056925 | 5 |
|  | GO:0002682 | regulation of immune system process | BP | 0.003862354 | 7 |
|  | GO:0048584 | positive regulation of response to stimulus | BP | 0.005000086 | 5 |
|  | GO:0044765 | single-organism transport | BP | 0.00529112 | 292 |
|  | GO:0048583 | regulation of response to stimulus | BP | 0.008847836 | 14 |
|  | GO:0042221 | response to chemical stimulus | BP | 0.014937374 | 63 |
|  | GO:0009607 | response to biotic stimulus | BP | 0.015291885 | 20 |
| *M.oleifera* | GO:0003735 | structural constituent of ribosome | MF | 1.23E-09 | 55 |
|  | GO:0051184 | cofactor transporter activity | MF | 7.63E-09 | 11 |
|  | GO:0016829 | lyase activity | MF | 8.94E-06 | 32 |
|  | GO:0097367 | carbohydrate derivative binding | MF | 7.73E-05 | 10 |
|  | GO:0045735 | nutrient reservoir activity | MF | 0.000548215 | 12 |
|  | GO:0061134 | peptidase regulator activity | MF | 0.004262883 | 5 |
|  | GO:0009521 | photosystem | CC | 1.05E-09 | 31 |
|  | GO:0034357 | photosynthetic membrane | CC | 3.08E-09 | 34 |
|  | GO:0030529 | ribonucleoprotein complex | CC | 1.22E-07 | 55 |
|  | GO:0005622 | intracellular | CC | 1.43E-06 | 55 |
|  | GO:0044424 | intracellular part | CC | 2.14E-05 | 181 |
|  | GO:0043228 | non-membrane-bounded organelle | CC | 2.59E-05 | 63 |
|  | GO:0044464 | cell part | CC | 0.000210526 | 194 |
|  | GO:0043229 | intracellular organelle | CC | 0.002182847 | 124 |
|  | GO:0033177 | proton-transporting two-sector ATPase complex, proton-transporting domain | CC | 0.005039457 | 9 |
|  | GO:0042445 | hormone metabolic process | BP | 1.14E-05 | 6 |
|  | GO:0044710 | single-organism metabolic process | BP | 1.35E-05 | 201 |
|  | GO:0044085 | cellular component biogenesis | BP | 0.000117777 | 30 |
|  | GO:0016049 | cell growth | BP | 0.002556299 | 5 |
|  | GO:0044707 | single-multicellular organism process | BP | 0.004478534 | 13 |
|  | GO:0044703 | multi-organism reproductive process | BP | 0.004504162 | 10 |
|  | GO:0044706 | multi-multicellular organism process | BP | 0.004504162 | 10 |
|  | GO:0022414 | reproductive process | BP | 0.008299012 | 10 |
|  | GO:0048610 | cellular process involved in reproduction | BP | 0.009996635 | 10 |
| *S.birrea* | GO:0097367 | carbohydrate derivative binding | MF | 1.22E-17 | 22 |
|  | GO:0005515 | protein binding | MF | 0.000242024 | 116 |
|  | GO:0036094 | small molecule binding | MF | 0.010869775 | 114 |
|  | GO:0004857 | enzyme inhibitor activity | MF | 0.013340899 | 9 |
|  | GO:0044710 | single-organism metabolic process | BP | 0.000658822 | 96 |
|  | GO:0009607 | response to biotic stimulus | BP | 0.003879699 | 6 |

**Table S9. Enriched pathways of genes in families with expansion.**

| Species | Pathway ID | KEGG description | Number of genes | P-value  (<=0.05) |
| --- | --- | --- | --- | --- |
| *V.subterranea* | ko00966 | Glucosinolate biosynthesis | 160 | 5.3E-124 |
|  | ko01110 | Biosynthesis of secondary metabolites | 435 | 1.18E-40 |
|  | ko03030 | DNA replication | 53 | 3.6E-11 |
|  | ko03440 | Homologous recombination | 43 | 1.31E-10 |
|  | ko03420 | Nucleotide excision repair | 52 | 5.63E-10 |
|  | ko00908 | Zeatin biosynthesis | 36 | 1.57E-07 |
|  | ko00520 | Amino sugar and nucleotide sugar metabolism | 65 | 1.89E-07 |
|  | ko00591 | Linoleic acid metabolism | 19 | 2.33E-07 |
|  | ko03430 | Mismatch repair | 33 | 2.91E-06 |
|  | ko00270 | Cysteine and methionine metabolism | 42 | 9.48E-06 |
|  | ko04070 | Phosphatidylinositol signaling system | 32 | 9.58E-06 |
|  | ko00564 | Glycerophospholipid metabolism | 39 | 2.35E-05 |
|  | ko00920 | Sulfur metabolism | 21 | 2.48E-05 |
|  | ko00130 | Ubiquinone and other terpenoid-quinone biosynthesis | 19 | 0.000252 |
|  | ko00360 | Phenylalanine metabolism | 40 | 0.000298 |
|  | ko00061 | Fatty acid biosynthesis | 17 | 0.000304 |
|  | ko00710 | Carbon fixation in photosynthetic organisms | 31 | 0.000448 |
|  | ko00940 | Phenylpropanoid biosynthesis | 62 | 0.000596 |
|  | ko00620 | Pyruvate metabolism | 35 | 0.001052 |
|  | ko00943 | Isoflavonoid biosynthesis | 11 | 0.001548 |
|  | ko01100 | Metabolic pathways | 465 | 0.00158 |
|  | ko03410 | Base excision repair | 21 | 0.001605 |
|  | ko00630 | Glyoxylate and dicarboxylate metabolism | 25 | 0.002112 |
|  | ko00020 | Citrate cycle (TCA cycle) | 20 | 0.002284 |
|  | ko00640 | Propanoate metabolism | 20 | 0.003303 |
|  | ko00030 | Pentose phosphate pathway | 24 | 0.004423 |
|  | ko00040 | Pentose and glucuronate interconversions | 49 | 0.006012 |
|  | ko00460 | Cyanoamino acid metabolism | 26 | 0.009262 |
|  | ko00500 | Starch and sucrose metabolism | 93 | 0.011727 |
|  | ko00900 | Terpenoid backbone biosynthesis | 20 | 0.011833 |
| *L. purpureus* | ko03440 | Homologous recombination | 49 | 5.08E-29 |
|  | ko03030 | DNA replication | 40 | 7.6E-20 |
|  | ko03420 | Nucleotide excision repair | 40 | 1.85E-17 |
|  | ko03430 | Mismatch repair | 34 | 1.28E-16 |
|  | ko00040 | Pentose and glucuronate interconversions | 29 | 3.61E-08 |
|  | ko00945 | Stilbenoid, diarylheptanoid and gingerol biosynthesis | 14 | 1.82E-06 |
|  | ko00500 | Starch and sucrose metabolism | 45 | 5.98E-06 |
|  | ko00950 | Isoquinoline alkaloid biosynthesis | 10 | 0.000974 |
|  | ko00073 | Cutin, suberine and wax biosynthesis | 8 | 0.001758 |
|  | ko03020 | RNA polymerase | 15 | 0.003137 |
|  | ko00901 | Indole alkaloid biosynthesis | 4 | 0.006322 |
|  | ko00965 | Betalain biosynthesis | 2 | 0.00686 |
|  | ko00062 | Fatty acid elongation | 8 | 0.009993 |
|  | ko00230 | Purine metabolism | 22 | 0.011178 |
|  | ko00350 | Tyrosine metabolism | 10 | 0.014795 |
|  | ko00960 | Tropane, piperidine and pyridine alkaloid biosynthesis | 7 | 0.016671 |
|  | ko00240 | Pyrimidine metabolism | 20 | 0.017911 |
|  | ko03040 | Spliceosome | 23 | 0.018301 |
|  | ko00941 | Flavonoid biosynthesis | 12 | 0.020864 |
|  | ko00640 | Propanoate metabolism | 6 | 0.023171 |
|  | ko03410 | Base excision repair | 8 | 0.035418 |
|  | ko00591 | Linoleic acid metabolism | 5 | 0.039401 |
|  | ko03050 | Proteasome | 7 | 0.04066 |
|  | ko00410 | beta-Alanine metabolism | 6 | 0.049893 |
| *F. albida* | ko04626 | Plant-pathogen interaction | 305 | 9.91E-17 |
|  | ko01110 | Biosynthesis of secondary metabolites | 685 | 2.78E-14 |
|  | ko00904 | Diterpenoid biosynthesis | 57 | 4.29E-11 |
|  | ko00450 | Selenocompound metabolism | 42 | 2.29E-10 |
|  | ko00945 | Stilbenoid, diarylheptanoid and gingerol biosynthesis | 47 | 6.82E-07 |
|  | ko00920 | Sulfur metabolism | 32 | 7.97E-06 |
|  | ko00941 | Flavonoid biosynthesis | 72 | 1.43E-05 |
|  | ko04141 | Protein processing in endoplasmic reticulum | 196 | 0.000477 |
|  | ko00270 | Cysteine and methionine metabolism | 56 | 0.001355 |
|  | ko04075 | Plant hormone signal transduction | 195 | 0.002284 |
|  | ko00020 | Citrate cycle (TCA cycle) | 39 | 0.003441 |
|  | ko00330 | Arginine and proline metabolism | 79 | 0.004352 |
|  | ko00944 | Flavone and flavonol biosynthesis | 24 | 0.006722 |
|  | ko00040 | Pentose and glucuronate interconversions | 73 | 0.009124 |
|  | ko00460 | Cyanoamino acid metabolism | 68 | 0.009177 |
|  | ko00640 | Propanoate metabolism | 25 | 0.015071 |
|  | ko04144 | Endocytosis | 120 | 0.018141 |
|  | ko03440 | Homologous recombination | 38 | 0.025177 |
|  | ko00290 | Valine, leucine and isoleucine biosynthesis | 20 | 0.025639 |
|  | ko00942 | Anthocyanin biosynthesis | 6 | 0.030386 |
|  | ko00340 | Histidine metabolism | 20 | 0.031301 |
|  | ko00750 | Vitamin B6 metabolism | 12 | 0.032321 |
|  | ko03430 | Mismatch repair | 37 | 0.035178 |
|  | ko03420 | Nucleotide excision repair | 49 | 0.041739 |
|  | ko00061 | Fatty acid biosynthesis | 17 | 0.047149 |
| *S.birrea* | ko00052 | Galactose metabolism | 54 | 2.77E-24 |
|  | ko00500 | Starch and sucrose metabolism | 75 | 6.45E-16 |
|  | ko04626 | Plant-pathogen interaction | 78 | 6.59E-11 |
|  | ko00330 | Arginine and proline metabolism | 23 | 2.3E-07 |
|  | ko03020 | RNA polymerase | 17 | 0.000164 |
|  | ko00620 | Pyruvate metabolism | 16 | 0.00033 |
|  | ko00940 | Phenylpropanoid biosynthesis | 32 | 0.00037 |
|  | ko00520 | Amino sugar and nucleotide sugar metabolism | 21 | 0.001325 |
|  | ko01100 | Metabolic pathways | 177 | 0.001449 |
|  | ko00460 | Cyanoamino acid metabolism | 12 | 0.006469 |
|  | ko00480 | Glutathione metabolism | 11 | 0.047416 |
| *M.oleifera* | ko00195 | Photosynthesis | 96 | 2.48E-21 |
|  | ko01100 | Metabolic pathways | 380 | 1.06E-12 |
|  | ko00190 | Oxidative phosphorylation | 65 | 1.96E-10 |
|  | ko00640 | Propanoate metabolism | 27 | 2.1E-08 |
|  | ko00061 | Fatty acid biosynthesis | 23 | 1.03E-07 |
|  | ko03010 | Ribosome | 75 | 1.95E-07 |
|  | ko00240 | Pyrimidine metabolism | 52 | 1.37E-06 |
|  | ko00450 | Selenocompound metabolism | 18 | 1.7E-06 |
|  | ko03020 | RNA polymerase | 35 | 1.77E-05 |
|  | ko00620 | Pyruvate metabolism | 28 | 0.000118 |
|  | ko00904 | Diterpenoid biosynthesis | 13 | 0.000132 |
|  | ko04710 | Circadian rhythm - mammal | 16 | 0.000184 |
|  | ko00040 | Pentose and glucuronate interconversions | 34 | 0.000395 |
|  | ko04141 | Protein processing in endoplasmic reticulum | 53 | 0.000482 |
|  | ko00908 | Zeatin biosynthesis | 12 | 0.000543 |
|  | ko01110 | Biosynthesis of secondary metabolites | 159 | 0.000661 |
|  | ko04626 | Plant-pathogen interaction | 58 | 0.001719 |
|  | ko00630 | Glyoxylate and dicarboxylate metabolism | 22 | 0.002512 |
|  | ko00966 | Glucosinolate biosynthesis | 7 | 0.006184 |
|  | ko00740 | Riboflavin metabolism | 4 | 0.00873 |
|  | ko00073 | Cutin, suberine and wax biosynthesis | 13 | 0.009551 |
|  | ko04120 | Ubiquitin mediated proteolysis | 30 | 0.013422 |
|  | ko00380 | Tryptophan metabolism | 11 | 0.01369 |
|  | ko00052 | Galactose metabolism | 20 | 0.020842 |
|  | ko00710 | Carbon fixation in photosynthetic organisms | 20 | 0.026452 |
|  | ko00902 | Monoterpenoid biosynthesis | 4 | 0.026786 |
|  | ko00950 | Isoquinoline alkaloid biosynthesis | 8 | 0.033978 |
|  | ko00230 | Purine metabolism | 38 | 0.038269 |
|  | ko00330 | Arginine and proline metabolism | 15 | 0.043208 |

**Table S10. The copy numbers of protein biosynthesis related genes in each species.**

| Category | *F. albida* | *L. purpureus* | *V. subterranea* | *S. birrea* | *M. oleifera* | *G.*  *max* | *T. aestivum* | *Z.*  *mays* | *O. sativa* |
| --- | --- | --- | --- | --- | --- | --- | --- | --- | --- |
| Ala biosynthesis | 3 | 3 | 6 | 4 | 3 | 8 | 12 | 10 | 2 |
| Arg, Gln, Glu, His and Pro biosynthesis | 54 | 42 | 59 | 39 | 35 | 119 | 195 | 174 | 62 |
| Asn and Asp biosynthesis | 19 | 11 | 16 | 11 | 12 | 163 | 74 | 22 | 18 |
| Cys, Gly and Ser biosynthesis | 37 | 20 | 43 | 37 | 23 | 63 | 94 | 53 | 33 |
| Ile, Leu and Val biosynthesis | 25 | 13 | 19 | 18 | 17 | 47 | 61 | 41 | 22 |
| Lys, Thr and Met biosynthesis | 39 | 19 | 21 | 17 | 13 | 49 | 100 | 70 | 33 |
| Phe, Trp and Tyr biosynthesis | 35 | 21 | 24 | 18 | 18 | 58 | 112 | 60 | 30 |
| Transport of amino acid and protein | 28 | 16 | 32 | 20 | 13 | 61 | 154 | 64 | 45 |
| Total | 240 | 145 | 220 | 164 | 134 | 568 | 802 | 494 | 245 |

**Table S11. The copy numbers of starch biosynthesis genes in each species.**

| Category | *F. albida* | *L. purpureus* | *V. subterranea* | *S. birrea* | *M. oleifera* | *G. max* | *O. sativa* | *T. aestivum* | *Z. mays* |
| --- | --- | --- | --- | --- | --- | --- | --- | --- | --- |
| AGPL | 5 | 4 | 4 | 3 | 5 | 8 | 5 | 9 | 15 |
| AGPS | 1 | 0 | 1 | 1 | 2 | 2 | 2 | 6 | 5 |
| BE | 2 | 1 | 2 | 1 | 2 | 2 | 3 | 22 | 3 |
| DPE | 2 | 2 | 2 | 1 | 2 | 4 | 2 | 6 | 4 |
| GBSS | 1 | 0 | 4 | 0 | 1 | 2 | 1 | 6 | 17 |
| ISA | 4 | 2 | 3 | 3 | 3 | 4 | 3 | 13 | 7 |
| PHOH | 2 | 3 | 4 | 3 | 4 | 6 | 2 | 18 | 2 |
| PUL | 1 | 1 | 1 | 1 | 1 | 1 | 1 | 5 | 4 |
| SS | 5 | 5 | 8 | 4 | 4 | 9 | 9 | 37 | 11 |
| Total | 23 | 18 | 29 | 17 | 24 | 38 | 28 | 122 | 68 |

Note: AGPL, AGPS, BE, DPE, GBSS, ISA, PHOH, PUL, and SS represent “ADP glucose pyrophosphorylase large subunit/glucose-1-phosphate

4 adenylyltransferase large subunit”, “glucose-1-phosphate adenylyltransferase small subunit”, “(starch) branching enzyme”, “putative

5 4-alpha-glucanotransferase”, “glucosyltransferase, starch synthase”, “putative isoamylase-type starch debranching enzyme”,

6 “alpha-glucanphosphorylase”, “starch debranching enzyme”, and “soluble starch synthase”.

**Table S12.** **The copy numbers of fatty acid synthesis and storage related genes in each species.**

| Category | *F. albida* | *L. purpureus* | *V. subterranea* | *S. birrea* | *M. oleifera* | *G. max* | *O. sativa* | *T. aestivum* | *Z. mays* |
| --- | --- | --- | --- | --- | --- | --- | --- | --- | --- |
| Acyltransferase | 12 | 6 | 13 | 8 | 5 | 18 | 11 | 35 | 33 |
| Base/Exchange Enzyme | 2 | 1 | 1 | 0 | 1 | 2 | 3 | 6 | 15 |
| Ceramidase / Ceramide Synthase (Acyl/CoA/independent) | 1 | 1 | 2 | 1 | 1 | 3 | 1 | 3 | 5 |
| Ceramide Fatty Acyl Amide alpha/Hydroxylase | 2 | 2 | 4 | 1 | 1 | 4 | 2 | 7 | 4 |
| Ceramide Sphingobase C4 Hydroxylase | 4 | 1 | 2 | 1 | 2 | 4 | 4 | 19 | 9 |
| Ceramide Sphingobase delta/4 Desaturase | 1 | 1 | 1 | 1 | 1 | 2 | 1 | 3 | 2 |
| Ceramide Sphingobase delta/8 Desaturase | 3 | 3 | 3 | 2 | 2 | 6 | 1 | 3 | 3 |
| Ceramide Synthase (Acyl/CoA : Sphingobase Acyltransferase) | 3 | 2 | 2 | 3 | 2 | 5 | 4 | 17 | 9 |
| Choline Kinase | 4 | 2 | 7 | 1 | 3 | 5 | 3 | 10 | 8 |
| CTP : Cholinephosphate Cytidylyltransferase | 3 | 0 | 2 | 1 | 1 | 5 | 3 | 8 | 6 |
| CTP : Ethanolaminephosphate Cytidylyltransferase | 3 | 2 | 2 | 1 | 2 | 4 | 4 | 10 | 3 |
| ER 2/Lysophosphatidate Acyltransferase (LPAAT) | 4 | 4 | 4 | 3 | 2 | 7 | 3 | 6 | 4 |
| ER CDP/Diacylglycerol Synthetase | 2 | 0 | 1 | 1 | 1 | 2 | 2 | 8 | 9 |
| ER Dihydroxyacetone/Phosphate Reductase | 3 | 4 | 5 | 3 | 2 | 7 | 4 | 16 | 6 |
| ER Glycerol/Phosphate Acyltransferase | 1 | 1 | 0 | 1 | 0 | 2 | 1 | 0 | 4 |
| ER OleateDesaturase | 2 | 4 | 2 | 2 | 1 | 7 | 4 | 11 | 9 |
| ER Phosphatidate Phosphatase | 4 | 1 | 2 | 0 | 1 | 4 | 6 | 19 | 17 |
| Ethanolamine Kinase | 1 | 1 | 1 | 1 | 2 | 2 | 1 | 3 | 7 |
| Glucosylceramide Synthase (UDP/Glucose: CeramideGlucosyltransferase) | 2 | 1 | 2 | 0 | 1 | 4 | 1 | 3 | 3 |
| Ketosphinganine Reductase | 1 | 1 | 2 | 1 | 1 | 2 | 1 | 4 | 5 |
| Phosphatidylinositol | 2 | 0 | 1 | 1 | 1 | 2 | 1 | 3 | 11 |
| Phosphoethanolamine N/Methyltransferase | 1 | 1 | 2 | 0 | 1 | 3 | 2 | 10 | 4 |
| Serine Palmitoyltransferase (LCB1) | 3 | 2 | 2 | 2 | 3 | 4 | 8 | 6 | 22 |
| Total | 64 | 41 | 63 | 35 | 37 | 104 | 71 | 210 | 198 |

**Table S13.** **The copy numbers of fatty acid degradation related genes in each species.**

| Category | *F. albida* | *L. purpureus* | *V. subterranea* | *S. birrea* | *M. oleifera* | *G. max* | *O. sativa* | *T. aestivum* | *Z. mays* |
| --- | --- | --- | --- | --- | --- | --- | --- | --- | --- |
| acetyl/CoA_acyltransferase_1 | 2 | 2 | 2 | 2 | 2 | 4 | 2 | 8 | 4 |
| acetyl/CoA_C/acetyltransferase | 2 | 0 | 3 | 2 | 2 | 4 | 2 | 11 | 8 |
| acyl/CoA_oxidase | 7 | 6 | 4 | 3 | 3 | 11 | 11 | 20 | 7 |
| aldehyde_dehydrogenase_(NAD+) | 13 | 9 | 15 | 4 | 9 | 27 | 13 | 48 | 12 |
| aldehyde_dehydrogenase_family_7_member_A1 | 1 | 0 | 1 | 1 | 1 | 2 | 1 | 3 | 5 |
| enoyl/CoA_hydratase/3/hydroxyacyl/CoA_dehydrogenase | 4 | 5 | 4 | 3 | 2 | 6 | 6 | 21 | 11 |
| long/chain_acyl/CoA_synthetase | 8 | 9 | 14 | 6 | 8 | 22 | 12 | 56 | 20 |
| Total | 37 | 31 | 43 | 21 | 27 | 76 | 47 | 167 | 67 |
