# Supplementary material for: The draft genomes of five agriculturally important African orphan crops
Source: Gigascience. 2018 Dec 7;8(3):giy152. doi: 10.1093/gigascience/giy152 (PMC6405277; doi:10.1093/gigascience/giy152)
Supplement: Response_to_Reviewer_Comments_Original_Submission.pdf [file gigascience_8_3_giy152_s9.pdf]

Author's Response To Reviewer Comments

Close

Responses to comments of Reviewer #1

The topic of nitrogen fixation is complex and well studied. The brief section in this paper begins to ask some good question (about presence of genes that play important roles in nodulation) - but the presentation is insufficient to conclude "The reason why F. albida showed a relatively lower ability to fix nitrogen [77] could be explained by the loss of IPD3, NFP, and some proteins with lower efficiency which would have taken its place in F. albida." See the recent papers by Greismann et al., 10.1126/science.aat1743 and van Velzen et al., https://doi.org/10.1073/pnas.1721395115, for state-of-the-art work in this area.

Response: Thank you for the suggestion. The suggested reference manuscript on the “Phylogenomics studies of nitrogen-fixing root nodule symbiosis” which is recently published in Science (Greismann et al.) is the outcome of our BGI-Research team along with our collaborators. We do referred the suggested papers, and removed the confused conclusion, and revised the description, as follows:
“The difference in the components within RNS pathway (Table 8) together with the relatively weak nitrogen-fixing ability [80] of F. albida thus make itself a good reference in the research of RNS diversification”.

1. Abstract: In the first sentence, the initial article, "A", is unnecessary ("A continued growth ...").

Response: According to your suggestion, we have revised the sentence, as follows:
“Continuous growth in the world population is expected to double the worldwide demand for food by 2050.”

2. Abstract, third sentence: typically, a sentence isn't started with a number ("30 species").

Response: According to your suggestion, we have revised the sentence, as follows:
“About 95% of the present food energy needs of humans are fulfilled by 30 species, within which wheat, maize and rice provide the majority of calories.”

3. Introduction: a minor point, but I am skeptical that the "World Population Prospects" from the U.N. (reference 1) is suitably paraphrased this way: "ensuring a sustainable food supply to meet the energy and nutritional needs of the expanding population is the greatest global challenge ahead of us." That is: scanning the report, I don't see that the report makes a claim about the "greatest global challenge" in an absolute sense (putting this need among others such as climate change, international conflict, etc.).

Response: Thank you for raising this question. According to your suggestion, we have revised the sentence, as follows:
“The world’s population is expected to reach 9.8 billion by 2050, thus ensuring a sustainable food supply to meet the energy and nutritional needs of the expanding population is one of the greatest global challenge.”

4. Introduction: "the utilization of crops plants appear to be the best choice" -- There is no other choice, right? We predominantly use crop plants (the only others being wild-harvested, non-crop foods).

Response: Thank you for the suggestion. According to your suggestion, we have revised the sentence, as follows:
“the utilization of potential crops (both model and non-model) plants appears to be a better choice.”

5. "which originated in West Africa, and cultivated in Sub-Saharan" --> "which originated in West Africa, and IS cultivated in Sub-Saharan" (for parallel construction)

Response: According to your suggestion, we have revised the sentence, as follows:
“which originated in West Africa, and is cultivated in Sub-Saharan areas, particularly Nigeria.”

6. "thereby highly making bambara groundnut a complete food" -- nonstandard word usage (omit "highly" to make it standard).

Response: According to your suggestion, we have omited “highly”, as follows:
“thereby making bambara groundnut a complete food.”

7. Section on Lablab: "South West" should be one word, and should probably lower-case unless it names a particular place, e.g. "the Southwest": "In southwestern parts of Bangladesh ..."

Response: According to your suggestion, we have revised the sentence, as follows:
“In southwestern parts of Bangladesh, lablab is reported to have a total production area of approximately 48000 ha.”

8. Extra period: "Kenya, approx.. 10,000"

Response: The suggested correction was implemented as follows:
“Kenya, approx. 10,000 ha”

9. Section on phylogenetic analysis: "divergence time between M. truncatula and legumes" -- what other legumes? (since Medicago is itself a legume)

Response: The suggested correction was implemented as follows:
“39-59 Mya between M. truncatula and the main branch of legumes, 15-30 Mya between G. max and P. vulgaris, and 83-90 Mya between T. cacao and A. thaliana.”

10. "In the present study, the divergence time between F. albida and Papilionoideae was predicted to be 79.1" - This is way outside the expected ranges, because the legume family itself is estimated to have originated around 60-64 Mya. Also, the value would depend on the particular species selected within the Papilionoideae - because rates are species-specific. See rates in Lavin et al. (2005), DOI: 10.1080/10635150590947131

Response: Thank you for raising this important point. We have removed the confused description, as follows:

“Based on the tree constructed by single-copy-family genes, the divergence time between F. albida and Papilionoideae was predicted to be 79.1 (70.0-87.0) Mya, which is a little different from the previous predicted origin of legumes based on two gene markers (matk and rbcL) (Lavin et al., 2005).”

11. Section "Identification of protein, starch, and fatty acid biosynthesis related genes" "Based on these observations we inferred that the ability to synthesize lecithin in V. subterranea is higher than that of soybeans" -- biosynthetic ability can't be inferred solely by the presence of gene sequences. All that can be said is that a necessary factor is present.

Response: Thank you for the suggestion. We do agree with your point, and removed the hypothetical description, and revised the sentence as follows:
“Based on these observations we inferred that the all the necessary factor to synthesize lecithin are present in V. subterranea.”

12. "... and in comparison with other orphan crops it has higher potential to be a new food crop." -- on what basis? Certainly not on the basis of gene composition, or on the ability to synthesize lecithin (which is itself of questionable nutritional value).

Response: Thank you for the suggestion. We do agree with your point, and removed the hypothetical description.

13. Sentence beginning "Therefore, this fine reference genomes together" needs to be rewritten. I don't think that "fine" is the intended word.

Response: Thank you for the suggestion. We have deleted this sentence.

14. Section "Identification of root nodule symbiosis pathway": "it has a major impact" --> "they have a major impact"

Response: According to your suggestion, we have revised the sentence, as follows:
“They have a major impact on global nitrogen cycle.”

15. Data availability: I see that PRJNA453822 points to Faidherbia (good), but I don't find PRJNA474418 in GenBank. Should the bioproject IDs be given for the other species in the study?

Response: Thank you for pointing this out. Actually, we have now released the data (PRJNA474418) in NCBI.

16. Data availability: "The assembly and annotation of the B. ceiba genome and other supporting data, including BUSCO results, are available in the GigaScience database" -- is this an error? I assume this refers to Bombax ceiba - which is not described in the paper.

Response: Thank you for pointing out the typing error. According to your suggestion, we have
revised the sentence, as follows:
“The assembly and annotation of the five genomes and other supporting data, including BUSCO results, are available in the GigaScience GigaDB repository.”

Responses to comments of Reviewer #2

1. The premises of the study talks about orphan crops which are important for Africa: to qualify this statement, the crops chosen should be either consumed or grown by Africans in large quantity: Based on the introduction and the statistics given therein M. oleifera and L. purpureus do not qualify.

Response: The improper description is replaced with "underutilized local plants". For example, in the abstract" ..enhance agricultural productivity and tackle malnutrition in these countries, a greater utilization of neglected or underutilized local plants (generally so-called orphan crops, but also a few plants with special contribution to agriculture, such agroforestry and nutrient) could be a partial solution".

2. M. oleifera genome is already sequenced and published (Tian et al., 2015; Sci China Life Sci. 2015 Jul; 58(7):627-38. doi: 10.1007/s11427-015-4872-x.). The manuscript neither mentions this fact nor compares their results with this.

Response: Thank you for the suggestion. We add the description in Page 5, L16-18, as follows:
"Prior to this study, a draft genome of Moringa oleifera from Yunnan (China) was also reported with similar genome assembly size and gene numbers compared to our version".

3. The results of RNA-seq have been used only for checking the genome completion suggesting gross underutilization of data. The materials and methods says just different parts of the plant has been subjected to RNA-seq. RNA-seq data of S. birrea is completely missing and there is no explanation of the same in the manuscript. The information provided in the supplementary file shows that there is no common denominator followed for the choice of tissue for RNA-seq. Further from table 5, it could be seen that only one among these various tissues have been used for checking the completeness of the WGS assembly. Overall, this gives a very hazy picture though a lot of work has been done and huge data-sets have been generated. I would recommend culling the data which is in no way utilized for obtaining the results provided in this manuscript.

Response: Thank you for raising this important point. We have actually compiled all the transcriptome data from different tissues, and used the combined version to check the completeness of the WGS assembly again. The results are shown in the Table 3 (not Table 5).

4. Genome and RNA-seq statistics are given only in Gb and Mb. This should be accompanied by number of reads and nucleotides.

Response: Thank you for the suggestion. According to your suggestion, we have revised the additional file 1: tableS1 and tableS2, and we used "bp" instead of "Gb", and also added "Reads number (bp)" data.

5. The difference between raw data and clean data seem to be too high ((30 to 43 %) except for S. birrea with respect to WGS data. Any specific reasons? This is even after keeping the cut off for quality score pretty low (< 16). Even for Sanger this kept as 20 while for NGS, this score is 30 to have high quality data.

Response: Thank you for pointing this out. Actually, the difference between raw and clean data is caused due to the filtering of the duplicated reads from the mate-pair libraries. However, for the pair-end data, the clean rate percentage were more than 80%. Therefore, we strongly believe that the cut off (<16) is suitable and reliable for our data. Kindly refer the below table for your kind perusal.

6. The comparison of orthologs within the five species does not seem to have a common ground as they belong to different species with not much evolutionary relationships to call for orthologous comparison. It would have been worthwhile to have the orthologous comparison with the related species. The choice of species in Table 5 needs to be explained.

Response: Thank you for the nice suggestion. We made the changes according to your suggestion. The orthologs of all the 14 species were identified just to get the single-copy-family genes for the construction of the tree. The comparison was made within fabids (for F. albida, L. purpureus and V. subterranea) and malvids (for M. oleifera and S. birrea) respectively. The species details in the Table 5 is now updated according to Figure 2.

7. In continuation of the previous point, the Vigna mungo genome and V. anguicularis genome should have been used along with other more complete legume genome (species) and mentioned in the manuscript while discussing the V subterranea.

Response: Thank you for the suggestion. We have now added the description in Page 5 L3-L4, as follows:
"The genomes of mung bean and adzuki bean have been published [9, 10], which also belongs to the Vigna genus"

8. The introduction does not talk about the previous genomic resources available in these five crops.
Response: Thank you for the suggestion. We admit our negligence. We have now added the relevant description regarding the previous genomic resources in the introduction section as well as in the data description, wherever necessary.

9. Table 4 formatting is confusing. Is it really required?

Response: Yes, the information on different classes of repeats (%) in five species is important. According to your suggestion, we have revised the table 4 for more better understanding. We have now classified the Repeat Type in a more detailed manner (Table 4)

10. A lot of analysis has been mentioned in Supplementary data - however there is no major point emerging out of it - such data may be removed from the manuscript altogether. It just increases the bulk of the paper without really contributing anything.

Response: Thank you for the suggestion. We have removed the previous
table S13. Comparative analysis of the protein biosynthesis related genes in each species.,
table S14. Comparative analysis of the starch biosynthesis related genes in each species.
table S15. Comparative analysis of the fatty acid-plastids biosynthesis related genes in each species.
table S16. Comparative analysis of the fatty acid synthesis and storage related genes in each

species.,
table S17. Comparative analysis of the fatty acid degradation related genes in each species. in additional file 2.
And add new table in additional file 1, as follows:
Table S6. Enriched pathways of unique paralogs genes in families.
Table S7. Enriched GO terms (level 3) of unique paralogs genes in families.
What's more ,we renumber the table.
11. Overall, results and discussion section shows hardly any discussion and incomplete results

Response: As our manuscript is a "data note" we focused mainly on data and its analysis part. The detailed findings and discussion will be presented in our subsequent manuscript covering the genomic data of several orphan crop species. The overall goal of the African Orphan Crops Consortium (AOCC) and BGI is to sequence, assemble and annotate the genomes of 101 plants contributed to traditional African food supplies by 2020 (www.africanorphancrops.org).

Minor shortcomings
1. Please read the manuscript carefully and check punctuation. Examples: Page 20: Line No: In other cereals .... in barley.
Page 22: LN: 48-50. Fragment owing to wrong punctuation.
2. The accession numbers of these data-sets are indicated as SSR in the respective supplementary tables.

Response: We have now rectified the above mentioned errors.

Responses to comments of Reviewer #3

1. The plants sequenced in this project have smaller genome size compared to many other sequenced crops, and repeat elements are also comparatively low. However none of the assemblies are complete and couldn't assemble into the chromosome level. If the authors have used long insert libraries also, it would have been better

Response: Thank you for the suggestion, we do agree with your comments. The incomplete assembly could be due to large fragments of repetitive sequences. This is one of the reasons, why we have submitted the manuscript as "data note" rather than "full length article". The experience gained from the sequencing of five orphan species, we plan to apply more sequencing strategies for the future African orphan project, like techniques generating longer reads.

2. "Various gene structure parameters were compared to the related species of each sequenced genome as summarized in table 5"- The number of protein coding genes in these sequenced genomes seems to be less compared to the related species. Can the authors provide an explanation for this?

Response: Thank you for the suggestion. The number of protein coding genes in V. subterranean and F. albida is similar to other legumes, except G. max and M. truncatula. These exceptionally large number is caused by their lineage-specific duplication. The lower numbers in other three species may be related to their smaller genome size. But, our BUSCO results showed a relative high completeness of core genes, compared to those of

other published plant genomes, and the size of the assemblies is closer to the estimated sizes. For instance, the previously reported gene number in M. oleifera (Tian et al., 2015; Sci China Life Sci. 2015) is extremely close to our number. Therefore, the possibility of mis-annotation of genes is pretty low.

3. Figure S5 is not provided

Response: Thank you for the suggestion. It is provided but our previous layout was confusing. Thank you for reminding, and we have modified it in this version.

4. 633, 372, 861, 364 and 216 genes are unannotated in V. subterranea L. purpureus F. albida S. birrea and M. oleifera respectively. Are these genes specific to the respective genomes?

Response: We found that there are 400, 305, 1514, 293, 172 unannotated genes which does not cluster with other species in gene family of V. subterranea L. purpureus F. albida S. birrea and M. oleifera respectively. Hence, we speculated that these genes are specific to the respective genomes. Kindly refer the specific results in the below table.

5. "Furthermore, the 10,103 gene families of V. subterranea, L. purpureus, F. albida, M. truncatula and G. max were clustered (Figure 2A). There were 1,105 orthologous families shared by the four Papilionoideae species, while 808 gene families containing 1,966 genes were specific to F. albida, 281 gene families containing 538 genes were specific to L. purpureus, 789 gene families containing 3,118 genes were specific to V. subterranea.
Moreover, 8,184 gene families of S. birrea, M. oleifera, C. papaya, C. sinensis and T. cacao were clustered (Figure 2B), of which 365 gene families containing 798 genes were specific to M. oleifera, 362 gene families containing 796 genes were specific to S. birrea, respectively". -To which class the specific genes mostly belong in the functional annotation?

Response: Thank you for raising the question. We additionally analyzed our data and updated the description as follows:
"The enrichment analysis on KEGG pathway of the paralogs genes were also calculated (Additional file1: Table S6, S7). The functional annotation revealed that they mainly correspond to the carbon fixation, zeatin biosynthesis, glyoxylate and dicarboxylate metabolism in V. subterranea. However, for L. purpureus, the fatty acid elongation pathway was enriched. While in F. albida, the pathways corresponding to the plant-pathogen interaction and cyanoamino acid metabolism were enriched. In S. birrea, the pathways of plant-pathogen interaction, starch and sucrose metabolism, fatty acid biosynthesis were enriched. In M. oleifera, the pathways related to fatty acid and diterpenoid biosynthesis, cyanoamino acid metabolism were enriched. The enrichment analysis on GO of paralogs genes were ion binding, metabolic process, disease resistance, cell component, biological process in V. subterranea, L. purpureus, F. albida, M. oleifera, and S. birrea respectively."

6. In the phylogenetic analysis with 141 single-copy genes from 14 species, Populus trichocarpa clusters with other members in Fabids. But in some other phylogenetic analysis

constructed using the same criteria, the group malpigiales, which includes Populus trichocarpa clusters with malvids or as a separate group. How do the authors explain this?

Response: Thank you for the nice suggestion. The figure 1 in the earlier version of manuscript was only a hand-drawn tree, and was used to display the taxonomy of our sequenced species. The taxonomic position of Populus trichocarpa was according to the NCBI taxonomy. The actual phylogenetic tree based on 141-gene was constructed without Populus trichocarpa (Figure 3 & 4). Therefore, to avoid the confusion between different phylogenetic trees in the manuscript, we have merged the previous figure 1 and 3, and moved figure 4 to the additional file1.

Chang et al 2018. Supporting data for "The draft genomes of five agriculturally important African orphan crops". GigaScience Database 2018. http://dx.doi.org/10.5524/100504.

Close
