# Supplementary material for: The draft genomes of five agriculturally important African orphan crops
Source: Gigascience. 2018 Dec 7;8(3):giy152. doi: 10.1093/gigascience/giy152 (PMC6405277; doi:10.1093/gigascience/giy152)
Supplement: Response_to_Reviewer_Comments_revision_1.pdf [file gigascience_8_3_giy152_s10.pdf]

Author's Response To Reviewer Comments

Close

Dear Dr. Scott,

We are glad to resubmit the thoroughly revised and cleaned version of our manuscript entitled “The draft genomes of five agriculturally important African orphan crops”, for possible publication in GigaScience as “Data Note”.
The corrections made and suggested by Dr. Lisa Martin were highly useful to significantly improve the quality of our manuscript. We have carefully implemented all the corrections suggested by her. Now we strongly believe that the revised manuscript is now ready for publication in GigaScience.

We look forward to hearing from you at your earliest convenience.
Yours sincerely,
Xin Liu

Reviewer comments:
Reviewer #1: The revisions in the last round satisfactorily addressed my concerns.
Reviewer #2: The revised version is well written and can be accepted for publication.
Response: We thank all the reviewers for their valuable suggestions in the earlier version of the manuscript, and for giving their kind acceptance to our manuscript.

Close
