# Supplementary material for: The draft genomes of five agriculturally important African orphan crops
Source: Gigascience. 2018 Dec 7;8(3):giy152. doi: 10.1093/gigascience/giy152 (PMC6405277; doi:10.1093/gigascience/giy152)
Supplement: Reviewer_1_Report_(Original_Submission) -- Steven Cannon — 9/10/2018 Reviewed [file gigascience_8_3_giy152_s11.pdf]

Reviewer Report

Title: The draft genomes of five agriculturally important African orphan crops

Version: Original Submission **Date:** 9/10/2018

Reviewer name: Steven Cannon

Reviewer Comments to Author:

General comments:

This paper describes the genome assemblies and annotations for several agronomically important "orphan crops," as part of the African Orphan Crops Consortium (AOCC).

The genome scaffold assemblies look to be of reasonable quality, for draft shotgun sequences. Likewise for the annotations. I am enthusiastic about the AOCC endeavor in general.

My own preference, as a reader of genome papers, is for simple, brief papers that sufficiently describe the methods and assembly characteristics and significance of the project. I would rather not plow through superficial analyses that are likely incorrect and incomplete. The sections on assembly and annotation methods look OK to me, as do the basic assessments of assembly and annotation quality.

On the other hand, the sections on gene family comparisons, gene function, evolutionary rate, and root nodule symbiosis, are generally shallow and sloppy. It is not possible, in general, to infer traits from crude gene family counts. The topic of nitrogen fixation is complex and well studied. The brief section in this paper begins to ask some good question (about presence of genes that play important roles in nodulation) - but the presentation is insufficient to conclude "The reason why F. albida showed a relatively lower ability to fix nitrogen [77] could be explained by the loss of IPD3, NFP, and some proteins with lower efficiency which would have taken its place in F. albida." See the recent papers by Greismann et al., 10.1126/science.aat1743 and van Velzen et al., https://doi.org/10.1073/pnas.1721395115, for state-of-the-art work in this area.

Also, the paper should be read and edited by a native English speaker. Construction and word usage is nonstandard in many parts of the paper.

Specific comments:

1. Abstract: In the first sentence, the initial article, "A", is unnecessary ("A continued growth ...").

2. Abstract, third sentence: typically, a sentence isn't started with a number ("30 species").

3. Introduction: a minor point, but I am skeptical that the "World Population Prospects" from the U.N. (reference 1) is suitably paraphrased this way: "ensuring a sustainable food supply to meet the energy and nutritional needs of the expanding population is the greatest global challenge ahead of us." That is: scanning the report, I don't see that the report makes a claim about the "greatest global challenge" in an absolute sense (putting this need among others such as climate change, international conflict, etc.).

4. Introduction: "the utilization of crops plants appear to be the best choice" -- There is no other choice, right? We predominantly use crop plants (the only others being wild-harvested, non-crop foods).

5. "which originated in West Africa, and cultivated in Sub-Saharan" --> "which originated in West Africa, and IS cultivated in Sub-Saharan" (for parallel construction)

6. "thereby highly making bambara groundnut a complete food" -- nonstandard word usage (omit "highly" to make it standard).

7. Section on Lablab: "South West" should be one word, and should probably lower-case unless it names a particular place, e.g. "the Southwest": "In southwestern parts of Bangladesh ..."

8. Extra period: "Kenya, approx.. 10,000"

9. Section on phylogenetic analysis: "divergence time between M. truncatula and legumes" -- what other legumes? (since Medicago is itself a legume)

10. "In the present study, the divergence time between F. albida and Papilionoideae was predicted to be 79.1" - This is way outside the expected ranges, because the legume family itself is estimated to have originated around 60-64 Mya. Also, the value would depend on the particular species selected within the Papilionoideae - because rates are species-specific. See rates in Lavin et al. (2005), DOI: 10.1080/10635150590947131.

11. Section "Identification of protein, starch, and fatty acid biosynthesis related genes"
"Based on these observations we inferred that the ability to synthesize lecithin in V. subterranea is higher than that of soybeans" -- biosynthetic ability can't be inferred solely by the presence of gene sequences. All that can be said is that a necessary factor is present.

12. "... and in comparison with other orphan crops it has higher potential to be a new food crop." -- on what basis? Certainly not on the basis of gene composition, or on the ability to synthesize lecithin (which is itself of questionable nutritional value).

13. Sentence beginning "Therefore, this fine reference genomes together" needs to be rewritten. I don't think that "fine" is the intended word.

14. Section "Identification of root nodule symbiosis pathway": "it has a major impact" --> "they have a major impact"

15. Data availability: I see that PRJNA453822 points to Faidherbia (good), but I don't find PRJNA474418 in GenBank. Should the bioproject IDs be given for the other species in the study?

16. Data availability: "The assembly and annotation of the B. ceiba genome and other supporting data, including BUSCO results, are available in the GigaScience database" -- is this an error? I assume this refers to Bombax ceiba - which is not described in the paper.

Methods

Are the methods appropriate to the aims of the study, are they well described, and are necessary controls included? Choose an item.

Conclusions

Are the conclusions adequately supported by the data shown? Choose an item.

Reporting Standards

Does the manuscript adhere to the journal’s guidelines on [minimum standards of reporting?]() Choose an item.

Choose an item.

Statistics

Are you able to assess all statistics in the manuscript, including the appropriateness of statistical tests used? Choose an item.

Quality of Written English

Please indicate the quality of language in the manuscript: Choose an item.

Declaration of Competing Interests

Please complete a declaration of competing interests, considering the following questions:

- Have you in the past five years received reimbursements, fees, funding, or salary from an organisation that may in any way gain or lose financially from the publication of this manuscript, either now or in the future?
- Do you hold any stocks or shares in an organisation that may in any way gain or lose financially from the publication of this manuscript, either now or in the future?
- Do you hold or are you currently applying for any patents relating to the content of the manuscript?
- Have you received reimbursements, fees, funding, or salary from an organization that holds or has applied for patents relating to the content of the manuscript?
- Do you have any other financial competing interests?
- Do you have any non-financial competing interests in relation to this paper?

If you can answer no to all of the above, write 'I declare that I have no competing interests' below. If your reply is yes to any, please give details below.

I declare that I have no competing interests

I agree to the open peer review policy of the journal. I understand that my name will be included on my report to the authors and, if the manuscript is accepted for publication, my named report including any attachments I upload will be posted on the website along with the authors' responses. I agree for my report to be made available under an Open Access Creative Commons CC-BY license (http://creativecommons.org/licenses/by/4.0/). I understand that any comments which I do not wish to be included in my named report can be included as confidential comments to the editors, which will not be published.

I agree to the open peer review policy of the journal

To further support our reviewers, we have joined with Publons, where you can gain additional credit to further highlight your hard work (see: https://publons.com/journal/530/gigascience). On publication of this paper, your review will be automatically added to Publons, you can then choose whether or not to claim your Publons credit. I understand this statement.

Yes Choose an item.
