# Supplementary material for: The draft genomes of five agriculturally important African orphan crops
Source: Gigascience. 2018 Dec 7;8(3):giy152. doi: 10.1093/gigascience/giy152 (PMC6405277; doi:10.1093/gigascience/giy152)
Supplement: Reviewer_2_Report_(Original_Submission) -- S. V. Amitha Mithra — 9/14/2018 Reviewed [file gigascience_8_3_giy152_s13.pdf]

Reviewer Report

Title: The draft genomes of five agriculturally important African orphan crops

Version: Original Submission **Date:** 9/14/2018

Reviewer name: S. V. Amitha Mithra

Reviewer Comments to Author:

The manuscript entitled "The draft genomes of five agriculturally important African orphan crops" submitted to Giga Science reports the draft whole genome sequencing of five crop species namely Vigna subterranea, Lablab purpureus, Faidherbia albida, Sclerocarya birrea, and Moringa oleifera. The authors have constructed libraries of various insert sizes and generated sufficient data to enable whole genome de novo assembly. They have identified various type of repeat sequences, predicted genes and annotated them using as many as tools and databases as possible. They have also identified transcription factors in these five species. Further they have compared the genomes the five species for completion using RNA-seq data and coverage of single copy genes. They have concentrated on root nodule related genes and starch synthesis related genes (especially with respect to resistance starch accumulation). The manuscript is written clearly and elaborately especially with respect to materials and methods. However it has the following shortcomings based on which it has been recommended for major revisions.

Major shortcomings:

1. The premises of the study talks about orphan crops which are important for Africa: to qualify this statement, the crops chosen should be either consumed or grown by Africans in large quantity: Based on the introduction and the statistics given therein M. oleifera and L. purpureus do not qualify.
2. M. oleifera genome is already sequenced and published (Tian et al., 2015; Sci China Life Sci. 2015 Jul; 58(7):627-38. doi: 10.1007/s11427-015-4872-x.). The manuscript neither mentions this fact nor compares their results with this.
3. The results of RNA-seq have been used only for checking the genome completion suggesting gross underutilization of data. The materials and methods says just different parts of the plant has been subjected to RNA-seq. RNA-seq data of S. birrea is completely missing and there is no explanation of the same in the manuscript. The information provided in the supplementary file shows that there is no common denominator followed for the choice of tissue for RNA-seq. Further from table 5, it could be seen that only one among these various tissues have been used for checking the completeness of the WGS assembly. Overall, this gives a very hazy picture though a lot of work has been done and huge data-sets have been generated. I would recommend culling the data which is in no way utilized for obtaining the results provided in this manuscript.
4. Genome and RNA-seq statistics are given only in Gb and Mb. This should be accompanied by number of reads and nucleotides.
5. The difference between raw data and clean data seem to be too high ((30 to 43 %) except for S. birrea with respect to WGS data. Any specific reasons? This is even after keeping the cut off for quality score pretty low (< 16). Even for Sanger this kept as 20 while for NGS, this score is 30 to have high quality data.
6. The comparison of orthologs within the five species does not seem to have a common ground as they belong to different species with not much evolutionary relationships to call for orthologous comparison. It would have been worthwhile to have the orthologous comparison with the related species. The choice of species in Table 5 needs to be explained.
7. In continuation of the previous point, the Vigna mungo genome and V. anguicularis genome should have been used along with other more complete legume genome (species) and mentioned in the manuscript while discussing the V subterranea.
8. The introduction does not talk about the previous genomic resources available in these five crops.

9. Table 4 formatting is confusing. Is it really required?
10. A lot of analysis has been mentioned din Supplementary data - however there is no major point emerging out of it - such data may be removed from the manuscript altogether. It just increases the bulk of the paper without really contributing anything.
11. Overall, results and discussion section shows hardly any discussion and incomplete results

Minor shortcomings:
1. Please read the manuscript carefully and check punctuation. Examples: Page 20: Line No: In other cereals .... in barley.
Page 22: LN: 48-50. Fragment owing to wrong punctuation.
2. The accession numbers of these data-sets are indicated as SSR in the respective supplementary tables.

Based on the above points, I recommend major revisions.

Methods

Are the methods appropriate to the aims of the study, are they well described, and are necessary controls included? Choose an item.

Conclusions

Are the conclusions adequately supported by the data shown? Choose an item.

Reporting Standards

Does the manuscript adhere to the journal's guidelines on [minimum standards of reporting?] Choose an item.

Choose an item.

Statistics

Are you able to assess all statistics in the manuscript, including the appropriateness of statistical tests used? Choose an item.

Quality of Written English

Please indicate the quality of language in the manuscript: Choose an item.

Declaration of Competing Interests

Please complete a declaration of competing interests, considering the following questions:

- Have you in the past five years received reimbursements, fees, funding, or salary from an organisation that may in any way gain or lose financially from the publication of this manuscript, either now or in the future?
- Do you hold any stocks or shares in an organisation that may in any way gain or lose financially from the publication of this manuscript, either now or in the future?

- Do you hold or are you currently applying for any patents relating to the content of the manuscript?
- Have you received reimbursements, fees, funding, or salary from an organization that holds or has applied for patents relating to the content of the manuscript?
- Do you have any other financial competing interests?
- Do you have any non-financial competing interests in relation to this paper?

If you can answer no to all of the above, write 'I declare that I have no competing interests' below. If your reply is yes to any, please give details below.

I declare that I have no competing interests

I agree to the open peer review policy of the journal. I understand that my name will be included on my report to the authors and, if the manuscript is accepted for publication, my named report including any attachments I upload will be posted on the website along with the authors' responses. I agree for my report to be made available under an Open Access Creative Commons CC-BY license (http://creativecommons.org/licenses/by/4.0/). I understand that any comments which I do not wish to be included in my named report can be included as confidential comments to the editors, which will not be published.

I agree to the open peer review policy of the journal

To further support our reviewers, we have joined with Publons, where you can gain additional credit to further highlight your hard work (see: https://publons.com/journal/530/gigascience). On publication of this paper, your review will be automatically added to Publons, you can then choose whether or not to claim your Publons credit. I understand this statement.

Yes Choose an item.
